# The incredible diversity of *Labiobaetis* Novikova & Kluge in New Guinea revealed by integrative taxonomy (Ephemeroptera, Baetidae)

**DOI:** 10.3897/zookeys.804.28988

**Published:** 2018-12-10

**Authors:** Thomas Kaltenbach, Jean-Luc Gattolliat

**Affiliations:** 1 Museum of Zoology, Palais de Rumine, Place Riponne 6, CH-1005 Lausanne, Switzerland Museum of Zoology Lausanne Switzerland; 2 University of Lausanne (UNIL), Department of Ecology and Evolution, CH-1015 Lausanne, Switzerland University of Lausanne Lausanne Switzerland

**Keywords:** COI, Indonesia, morphology, new species, Papua New Guinea, species delimitation

## Abstract

Material collected between 1999 and 2011 in Papua New Guinea and the Papua Province of Indonesia unveiled the enormous diversity of *Labiobaetis* on this island. Twenty-six new species were identified and delimited by integrative taxonomy using genetic distance (COI, Kimura-2-parameter) and morphology. These new species are described and illustrated based on larvae, augmenting the total number of *Labiobaetis* species on the island of New Guinea to 32. Seven morpho-groups of species are proposed based on morphological characters and a key to all New Guinea species is provided. The generic attributes of the larvae are summarised and slightly modified based on the examinations of the new species. Results on the genetics of most species (COI) are also provided. The interspecific K2P distances are between 13% and 32%, the intraspecific distances usually between 0% and 2%. Possible reasons for the remarkable richness of this genus in New Guinea are discussed.

## Introduction

The family Baetidae has the highest species diversity among mayflies, comprising more than 1000 species in 104 genera, which is approximately one quarter of all mayfly species worldwide (Gattolliat and Nieto 2009, Sartori and Brittain 2015). It has a cosmopolitan distribution with the exception of Antarctica and New Zealand. Investigations of the molecular phylogeny of the Order Ephemeroptera revealed the relatively primitive status of the family (Ogden and Whiting 2005, Ogden et al. 2009).

The genus *Labiobaetis* Novikova & Kluge, 1987 is one of the richest genera of Baetidae with 79 described species (Barber-James et al. 2013, Webb 2013, Shi and Tong 2014, Kubendran et al. 2014, 2015, Gattolliat et al. 2018). However, 19 species have been described based on adults only, which are providing too limited characteristics for a clear differentiation of species and may cause difficulties to associate larval and imaginal stages at a later point in time. The distribution of *Labiobaetis* is nearly worldwide, with the exception of the Neotropical realm and New Caledonia, and it is extremely diversified in the Afrotropical (28 species) and Oriental realms (23 species) (Gattolliat and Nieto 2009). The status and validity of the genus has often been the subject of controversy during the last two decades (Lugo-Ortiz and McCafferty 1997, Lugo-Ortiz et al. 1999, Gattolliat 2001, Fujitani et al. 2003, Fujitani 2008, Gattolliat and Staniczek 2011; Kluge 2012; Kubendran et al. 2015, Kluge and Novikova 2016). Furthermore, molecular reconstructions indicated that the concept of *Labiobaetis* is most probably at least diphyletic (Monaghan et al. 2005, Gattolliat et al. 2008).

Originally *Labiobaetis* was established as a subgenus of *Baetis* Leach (Novikova and Kluge 1987) and later elevated to generic rank by McCafferty and Waltz (1995). Some of the species were originally described as and for a long time remained classified in the genus *Baetis* (Agnew 1961, Gillies 1993, 1994, Müller-Liebenau and Soldán 1981, Müller-Liebenau 1982, Müller-Liebenau 1984a, b, Müller-Liebenau and Hubbard 1985). Some characters of *Labiobaetis*, especially a process at the outer apical margin of the antennal scape and a distomedial excavation at maxillary palp segment II of the larvae, were already recognised as diagnostic characters by Müller-Liebenau (1969) when she established the *atrebatinus* group of species within the genus *Baetis* from Europe. Later, Morihara and McCafferty (1979b) introduced the *propinquus* group of *Baetis* species from North America, based on the same characters and Müller-Liebenau (1984a) proposed the *molawinensis* group of *Baetis* species from the Oriental Region, based on the above and some additional characters. Müller-Liebenau (1984a) also proposed the *atrebatinus* complex comprising the *atrebatinus*, *propinquus* and *molawinensis* groups of species. The concept of *Labiobaetis* as proposed by McCafferty and Waltz (1995) encompassed the whole *atrebatinus* complex (and as a consequence the three species groups) and therefore had a distribution including North America, Europe and Asia. Lugo-Ortiz and McCafferty (1997) increased the distribution of the genus by describing six new species of *Labiobaetis* from the Afrotropics and transferring eight Afrotropical species to this genus. Later the genus was reorganised under the name *Pseudocloeon* with *Labiobaetis* as junior synonym by Lugo-Ortiz et al. (1999) based on adult morphology. However, Gattolliat (2001) contradicted this opinion by the reason that the larval stage of the type-species *P.kraepelini* Klapálek remained unknown and a complete analysis of the generic situation was therefore not possible, in the meantime, he also proposed to restrict the concept of *Pseudocloeon* to the type-species *P.kraepelini*, which is in line with a previous proposal of Waltz and McCafferty (1985,^1987^) and to wait with the final generic assignment of all the species until a global revision of these genera could be done based on material from their whole distribution range. This proposal is today generally followed by most authors (Fujitani et al. 2003, McCafferty et al. 2010, Kluge and Novikova 2011, 2014, 2016, Kubendran et al. 2014, Shi and Tong 2014). Recently, Kluge and Novikova (2016) established a new tribe Labiobaetini including the genera *Labiobaetis* and *Pseudopannota* Waltz & McCafferty, 1987, based on a unique combination of imaginal and larval characters. They also formally transferred all the species of *Pseudocloeon* to *Labiobaetis*, except the type species *Pseudocloeonkraepelini*.

Up to now there was only one study on *Labiobaetis* s. l. under the genus name *Pseudocloeon* Klapálek, 1905 from New Guinea (Lugo-Ortiz et al. 1999) with descriptions of six new species: *P.involutum* Lugo-Ortiz & McCafferty, *P.petersorum* Lugo-Ortiz & McCafferty, *P.tuberpalpus* Lugo-Ortiz & McCafferty, *P.vitile* Lugo-Ortiz & McCafferty, *P.vultuosum* Lugo-Ortiz & McCafferty and *P.xeniolum* Lugo-Ortiz & McCafferty. Here we are describing 26 new species of *Labiobaetis* based on recently collected larvae from different locations in New Guinea, including both Papua New Guinea and the Papua Province of Indonesia. Thereby we are considering *Labiobaetis* in a broad sense, even if we presume that the genus is probably polyphyletic. We are currently still missing morphological characters and especially genetic evidence to split the genus into monophyletic lineages. Genetic studies on species from all realms involving nuclear genes are necessary to unveil the generic delimitation of *Labiobaetis* at a later point in time.

## Materials and methods

The specimens were mainly collected by Michael Balke (Zoologische Staatssammlung München, ZSM, Germany) and members of his local team in Papua New Guinea and Indonesia (Papua Province). Further specimens were collected by L. Čížek (Institute of Entomology, Biology Centre CAS, České Budějovice) and Katayo Sagata (University of Papua New Guinea).

The specimens were preserved in 70%–96% ethanol. The dissection of larvae was done in Cellosolve (2-Ethoxyethanol) under Olympus SZX7 stereomicroscope and mounted on slides with Euparal liquid.

The DNA of part of the specimens was extracted by using non-destructive methods, which allows for subsequent morphological analysis (see Vuataz et al. 2011 for details). We amplified a 658 bp fragment of the mitochondrial gene cytochrome oxidase subunit 1 (COI) using the primers LCO 1490 (GGTCAACAAATCATAAAGATATTGG) and HCO 2198 (TAAACTTCAGGGTGACCAAAAAATCA) (Folmer et al. 1994). The polymerase chain reaction was conducted with an initial denaturation temperature of 98 °C for 30 sec followed by a total of 37 cycles with denaturation temperature of 98 °C for 10 sec, an annealing temperature of 50 °C for 30 sec and an extension at 72 °C for 30 sec, final extension at 72 °C for 2 min. Sequencing was done with Sanger’s method (Sanger et al. 1977). The genetic variability between specimens was estimated using Kimura-2-parameter distances (K2P, Kimura 1980) as well as *p*-distances, both of which were calculated with the program MEGA 7 (Kumar et al. 2016, http://www.megasoftware.net). The GenBank accession numbers are given in Table 1, nomenclature and ranking for genetic sequences was done according to Chakrabarty et al. 2013.

Drawings were made using an Olympus BX43 microscope. Photographs of larvae were taken with a Canon EOS 6D camera and the Visionary Digital Passport imaging system (http://www.duninc.com) and processed with the programs Adobe Photoshop Lightroom (http://www.adobe.com) and Helicon Focus version 5.3 (http://www.heliconsoft.com). Photographs were subsequently enhanced with Adobe Photoshop Elements 13.

The distribution map was generated with the program Simple Mapper (http://research.amnh.org/pbi/maps), the program GEOLocate (http://www.museum.tulane.edu/geolocate/web/WebGeoref.aspx) as well as Google Earth (http://www.google.com/earth/download/ge/) were used to attribute approximate GPS coordinates to sample locations of Lugo-Ortiz et al. (1999) and of *L.inopinatus* sp. n.

The taxonomic descriptions and the key presented herein were generated with a DELTA (Dallwitz 1980, Dallwitz et al. 1999) database containing the morphological states of characters of *Labiobaetis* species of New Guinea.

The new species described in this study were all compared to paratypes (on slides) of the six known species from New Guinea (deposited in Purdue University, West Lafayette, Indiana, USA).

For the terminology, we are referring to Hubbard (1995) and Morihara and McCafferty (1979a).

**Table 1.** Sequenced specimens.

Species	Locality	Specimen catalog #	GenBank # (COI)	GenSeq Nomenclature
*L.balkei* sp. n.	Papua New Guinea, Central Prov.	GBIFCH 00465156	MH619492	genseq-1 COI
		GBIFCH 00465157	MH619493	genseq-2 COI
*L.lobatus* sp. n.	Papua New Guinea, Central Prov.	GBIFCH 00508141	MH619503	genseq-1 COI
*L.michaeli* sp. n.	Papua New Guinea, Eastern Highlands	GBIFCH 00508129	MH619477	genseq-1 COI
		GBIFCH 00508130	MH619478	genseq-2 COI
		GBIFCH 00508134	MH619484	genseq-2 COI
*L.claudiae* sp. n.	Papua New Guinea, Madang Prov.	GBIFCH 00508144	MH619479	genseq-1 COI
*L.stagnum* sp. n.	Indonesia, Papua Prov.	GBIFCH 00465168	MH619491	genseq-2 COI
*L.orientis* sp. n.	Papua New Guinea, Eastern Highlands	GBIFCH 00465169	MH619496	genseq-1 COI
*L.papuaensis* sp. n.	Indonesia, Papua Prov.	GBIFCH 00465170	MH619502	genseq-1 COI
*L.gladius* sp. n.	Papua New Guinea, Western Highlands	GBIFCH 00465179	MH619486	genseq-4 COI
*L.janae* sp. n.	Indonesia, Papua Prov.	GBIFCH 00465181	MH619483	genseq-1 COI
		GBIFCH 00465182	MH619489	genseq-2 COI
*L.branchiaesetis* sp. n.	Papua New Guinea, Eastern Highlands	GBIFCH 00465183	MH619480	genseq-1 COI
*L.planus* sp. n.	Indonesia, Papua Prov.	GBIFCH 00508149	MH619485	genseq-2 COI
		GBIFCH 00508150	MH619487	genseq-2 COI
*L.podolakae* sp. n.	Papua New Guinea, Eastern Highlands	GBIFCH 00465194	MH619500	genseq-2 COI
*L.schwanderae* sp. n.	Papua New Guinea, Gulf Prov.	GBIFCH 00465197	MH619501	genseq-1 COI
*L.altus* sp. n.	Papua New Guinea, Enga Prov.	GBIFCH 00508131	MH619481	genseq-2 COI
*L.gindroi* sp. n.	Indonesia, Papua Prov.	GBIFCH 00465203	MH619490	genseq-2 COI
*L.paravitilis* sp. n.	Papua New Guinea, Madang Prov.	GBIFCH 00508148	MH619482	genseq-2 COI
*L.paravultuosus* sp. n.	Papua New Guinea, Enga Prov.	GBIFCH 00465213	MH619498	genseq-1 COI
		GBIFCH 00465214	MH619499	genseq-2 COI
*L.centralensis* sp. n.	Papua New Guinea, Central Prov.	GBIFCH 00465215	MH619495	genseq-1 COI
		GBIFCH 00465216	MH619494	genseq-2 COI
*L.elisae* sp. n.	Papua New Guinea, Western Highlands	GBIFCH 00465219	MH619497	genseq-1 COI
*L.vallus* sp. n.	Papua New Guinea, Madang Prov.	GBIFCH 00465226	MH619488	genseq-1 COI

## Results

### New species descriptions

Abbreviations:

**MZL**Museum of Zoology Lausanne (Switzerland)

**ZSM**Zoologische Staatssammlung München (Germany)

**MZB**Museum Zoologicum Bogoriense (Indonesia)

#### List of species

*balkei* group

1. *L.balkei* sp. n.

2. *L.lobatus* sp. n.

3. *L.michaeli* sp. n.

*claudiae* group

4. *L.claudiae* sp. n.

5. *L.stagnum* sp. n.

*orientis* group

6. *L.orientis* sp. n.

7. *L.papuaensis* sp. n.

*petersorum* group

8. *L.petersorum*

9. *L.gladius* sp. n.

10. *L.janae* sp. n.

*tuberpalpus* group

11. *L.tuberpalpus*

12. *L.branchiaesetis* sp. n.

13. *L.magnovaldus* sp. n.

14. *L.planus* sp. n.

15. *L.podolakae* sp. n.

16. *L.rutschmannae* sp. n.

17. *L.schwanderae* sp. n.

*vitilis* group

18. *L.vitilis*

19. *L.altus* sp. n.

20. *L.gindroi* sp. n.

21. *L.paravitilis* sp. n.

22. *L.wilhelmensis* sp. n.

*vultuosus* group

23. *L.vultuosus*

24. *L.paravultuosus* sp. n.

not assigned to a group

25. *L.centralensis* sp. n.

26. *L.dendrisetis* sp. n.

27. *L.elisae* sp. n.

28. *L.inopinatus* sp. n.

29. *L.involutus*

30. *L.pindaundensis* sp. n.

31. *L.vallus* sp. n.

32. *L.xeniolus*

**Figure 1.**
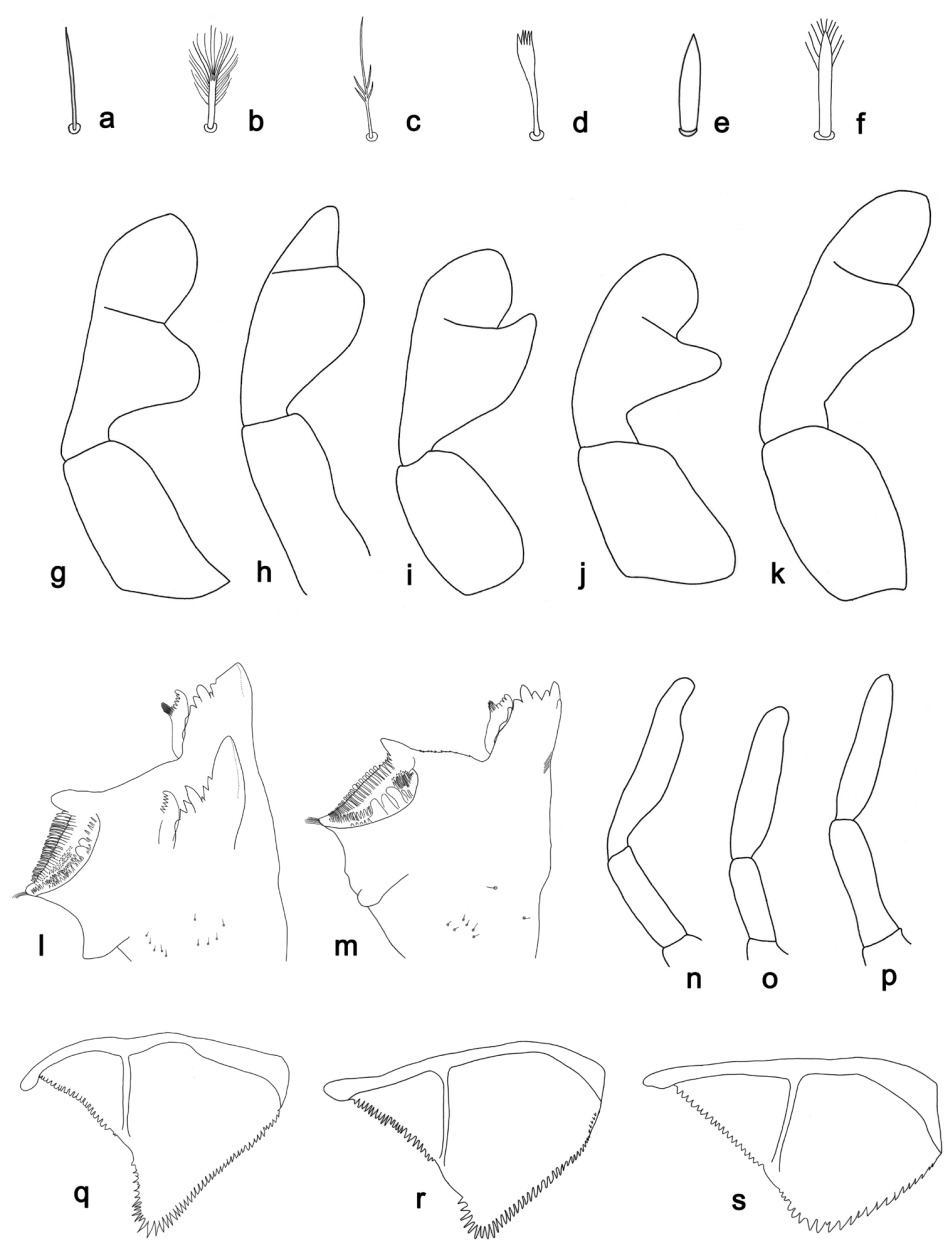
*Labiobaetis*, states of selected characters: **a-f** setae of the labrum dorsal, submarginal arc of setae **a** simple **b** feathered **c** dendritic **d** spatulate **e** lanceolate **f** lanceolate with apical pectination; **g–k** labial palp, distomedial protuberance of segment II and shape of segment III, **g** lobed and slightly pentagonal **h** compact, rounded and conical **i** hook-like and slightly pentagonal **j** thumb-like elongated and about semicircular **k** short thumb-like and oblong; **l-m** left mandible, **l** outermost denticle blade-like **m** denticles unmodified; **n–p** maxillary palp segment II, **n** with distolateral excavation **o** with slight distolateral excavation **p** without distolateral excavation; **q–s** paraproct, **q** distally expanded **r** distally slightly expanded **s** distally not expanded.

#### *L.balkei* group of species

The group is characterised by a large, lobed, distomedial protuberance of labial palp segment II and a dorsal, submarginal arc of setae of the labrum composed of spatulate, apically pectinate setae.

##### 1. Labiobaetis balkei sp. n.

Taxon classificationAnimaliaEphemeropteraBaetidae

http://zoobank.org/8F3BE8A6-4790-48A7-96DA-95527563DC87

**Figure 2.**
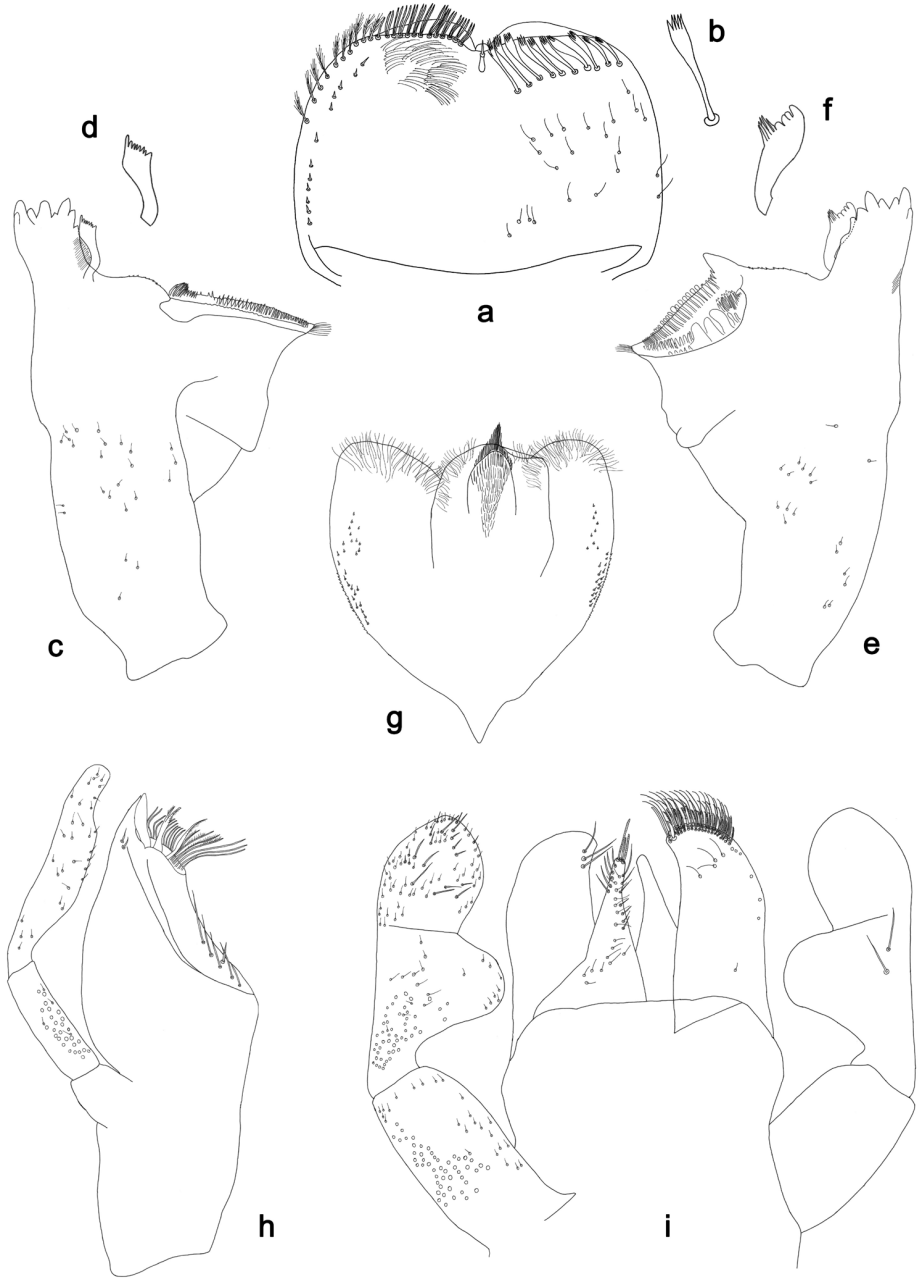
*Labiobaetisbalkei* sp. n., larva morphology: **a** Labrum **b** Labrum dorsal, submarginal seta **c** Right mandible **d** Right prostheca **e** Left mandible **f** Left prostheca **g**Hypopharynx**h** Maxilla **i** Labium.

**Figure 3.**
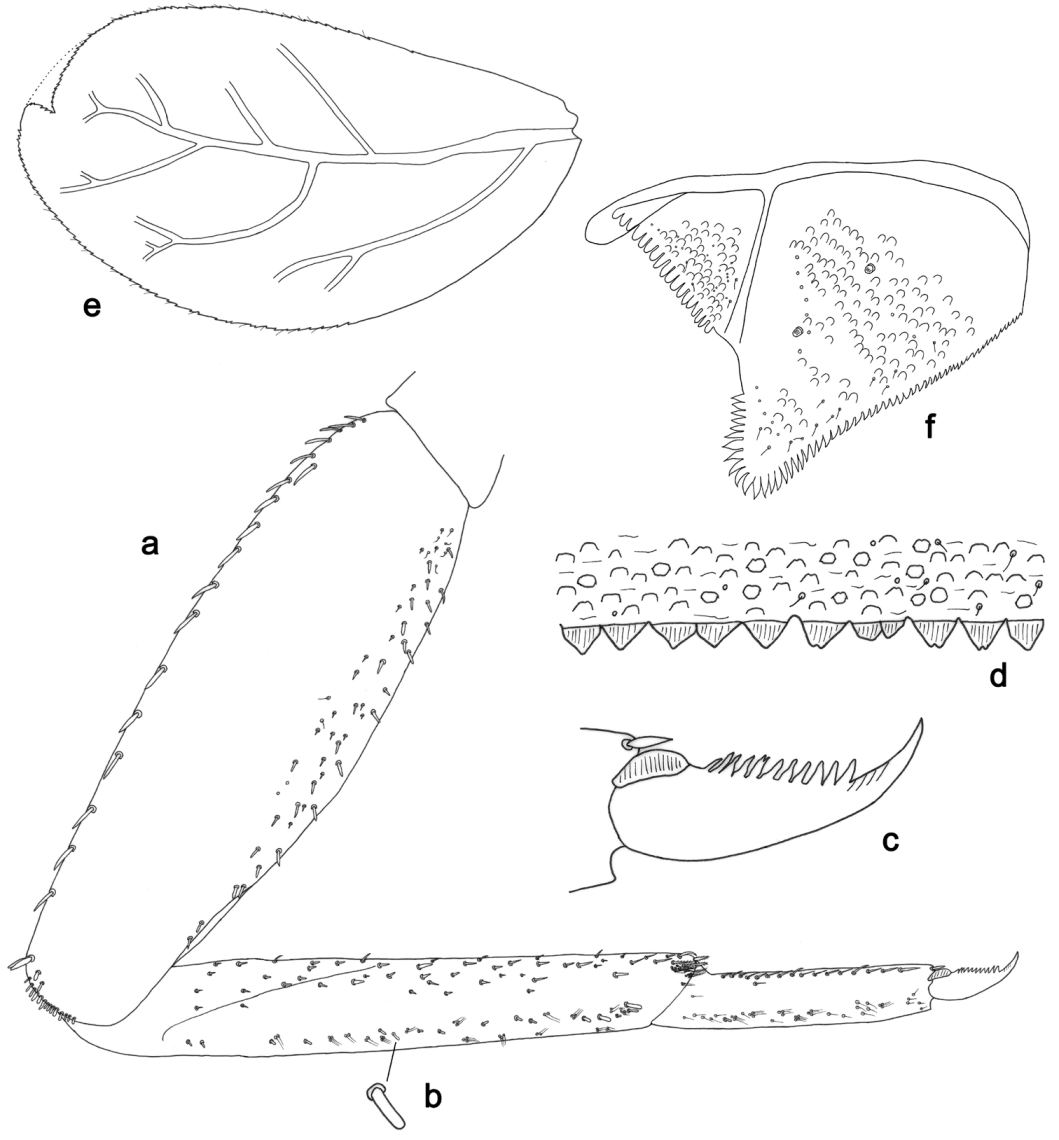
*Labiobaetisbalkei* sp. n., larva morphology: **a**Foreleg**b** Tibia dorsal seta **c** Fore claw **d**Tergum IV **e** Gill IV **f**Paraproct.

Figures 2 , 3 , 58a , 64a

###### Diagnosis.

**Larva.** Following combination of characters: A) labrum dorsal submarginal arc of setae composed of 10–11 spatulate, apically pectinate setae; B) labial palp segment II with a large, lobed distomedial protuberance; C) fore femur length ca. 3× maximum width, dorsally with a row of ca. 17 curved, spine-like setae on margin and a few curved, spine-like setae near proximal margin ; D) tibia dorsally with stout, spatulate, apically rounded setae along margin; E) claw with a row of 11–12 denticles; F) paraproct distally expanded.

###### Description.

**Larva** (Figs 2, 3, 58a). Body length 5.3 mm.

*Colouration.* Head, thorax and abdomen dorsally brown, thorax with brighter, faint pattern as in Fig. 58a. Head and thorax with bright median, dorsal suture. Thorax and abdomen ventrally colourless, femur with brown, distomedial spot, dorsal margin of femur brown, legs otherwise colourless, caudal filaments colourless.

*Antenna* with scape and pedicel sub-cylindrical, without distolateral process at scape; flagellum with lanceolate spines on apex of each segment.

*Labrum* (Fig. 2a, b). Rectangular, length 0.7× maximum width. Distal margin with medial emargination and a small process. Dorsally with medium, fine, simple setae scattered over surface; submarginal arc of setae composed of 10–11 long, spatulate, apically pectinate setae. Ventrally with marginal row of setae composed of lateral and anterolateral long, feathered setae and medial long, bifid setae; ventral surface with 12 short, spine-like setae near lateral and anterolateral margin.

*Right mandible* (Fig. 2c, d). Incisors fused. Outer and inner sets of denticles with 4 + 3 denticles plus one small intermediate denticle. Inner margin of innermost denticle with a row of thin setae. Prostheca robust, apically denticulate. Margin between prostheca and mola slightly convex, with minute denticles. Tuft of setae at apex of mola present.

*Left mandible* (Fig. 2e, f). Incisors fused. Outer and inner sets of denticles with 4 + 3 denticles. Prostheca robust, apically with small denticles and comb-shape structure. Margin between prostheca and mola straight, with minute denticles towards subtriangular process. Subtriangular process long and slender, above level of area between prostheca and mola. Denticles of mola apically constricted. Tuft of setae at apex of mola present.

Both mandibles with lateral margins almost straight. Basal half with fine, simple setae scattered over dorsal surface.

*Hypopharynx* (Fig. 2g). Lingua shorter than superlingua. Lingua longer than broad; medial tuft of stout setae present; distal half not expanded. Superlingua rounded; lateral margin rounded; fine, long, simple setae along distal margin.

*Maxilla* (Fig. 2h). Galea-lacinia with two simple, robust apical setae under crown. Inner dorsal row of setae with three denti-setae, distal denti-seta tooth-like, middle and proximal denti-setae slender, bifid and pectinate. Medially with one spine-like seta and five long, simple setae. Maxillary palp 1.2× as long as length of galea-lacinia; two segmented. Palp segment II 1.8× length of segment I. Setae on maxillary palp fine and simple, scattered over surface of segments I and II. Apex of last segment rounded, with excavation at inner distolateral margin.

*Labium* (Fig. 2i). Glossa basally broad, narrowing toward apex; shorter than paraglossa; inner margin with seven spine-like setae increasing in length distally; apex with three long, robust, pectinate setae; outer margin with five long, spine-like setae; ventral surface scattered with fine, simple setae. Paraglossa sub-rectangular, curved inward; apex rounded, ventrally with three rows of long, robust, apically pectinate setae; dorsally with a row of four medium, simple setae; ventrally with three long, spine-like setae near inner margin. Labial palp with segment I 0.6× length of segments II and III combined. Segment I covered with short, fine, simple setae ventrally and micropores dorsally. Segment II with a large, lobed distomedial protuberance; distomedial protuberance 0.9× width of base at segment III; inner and outer margin both with short, fine, simple setae; dorsally with two long, spine-like, simple setae. Segment III slightly pentagonal; apex slightly pointed; length 1.1× width; ventrally covered with long and medium spine-like, simple setae and short, fine, simple setae.

*Hind wing pads* absent.

*Foreleg* (Fig. 3a, b, c). Ratio of foreleg segments 1.1:1.0:0.4:0.2. *Femur*. Length ca. 3× maximum width. Dorsal marign with a row of 17–18 curved, spine-like setae and 1–4 curved, spine-like setae near proximal area, length of setae 0.15× maximum width of femur. Apex rounded with one pair of curved, spine-like setae and many short, stout, apically rounded setae. Many stout, lanceolate setae and a few fine, simple setae scattered along the ventral marign; femoral patch poorly developed. *Tibia.* Dorsal margin with stout, lanceolate, apically rounded setae and very fine, simple setae scattered. Ventral margin with a row of curved, spine-like setae and some longer, spine-like, bipectinate setae and a tuft of long, fine, simple setae on apex. Anterior surface scattered with many stout, lanceolate setae. Tibio-patellar suture present on basal 1/2. *Tarsus.* Dorsal margin with a row of fine, simple setae. Ventral marign with a row of curved, spine-like setae. Tarsal claw with one row of 11–12 denticles; distally pointed; with four stripes; subapical setae absent.

*Tergum* (Fig. 3d). Surface with irregular rows of slightly W-shaped scale bases and scattered fine, simple setae and micropores; scales short, apically rounded. Posterior margin of tergum IV with triangular spines, wider than long.

*Gills* (Fig. 3e). Present on segments II–VII. Margin with small denticles intercalating fine simple setae. Tracheae extending from main trunk to inner and outer margins. Gill IV as long as length of segments V and 2/3 VI combined. Gill VII as long as length of segments VIII and 1/2 IX combined.

*Paraproct* (Fig. 3f). Distally expanded, with many marginal, stout spines. Surface with U-shaped scale bases and scattered fine, simple setae and micropores. Postero-lateral extension (cercotractor) with small marginal spines.

###### Etymology.

Dedicated to Michael Balke (Zoologische Staatssammlung München, ZSM), who collected most of the fantastic material treated in this study.

###### Distribution.

New Guinea.

###### Biological aspects.

The specimens were collected at an altitude of 1400 m a.s.l.

###### Type-material.

**Holotype.** Nymph (on slide, GBIFCH 00465156), Papua New Guinea, Central, Kokoda Trek, 1400 m, Jan 2008, 09°01.95'S, 147°44.46'E, Posman (PNG 172). Deposited in ZSM. **Paratypes.** 37 nymphs (2 on slides, GBIFCH 00465157, GBIFCH 00465158, 20 in alcohol, GBIFCH 00515225, deposited in MZL; 15 in alcohol, GBIFCH 00515226, deposited in ZSM), same data as holotype.

##### 2. Labiobaetis lobatus sp. n.

Taxon classificationAnimaliaEphemeropteraBaetidae

http://zoobank.org/816C5845-98C9-427C-9B62-CED7A88A1FED

**Figure 4.**
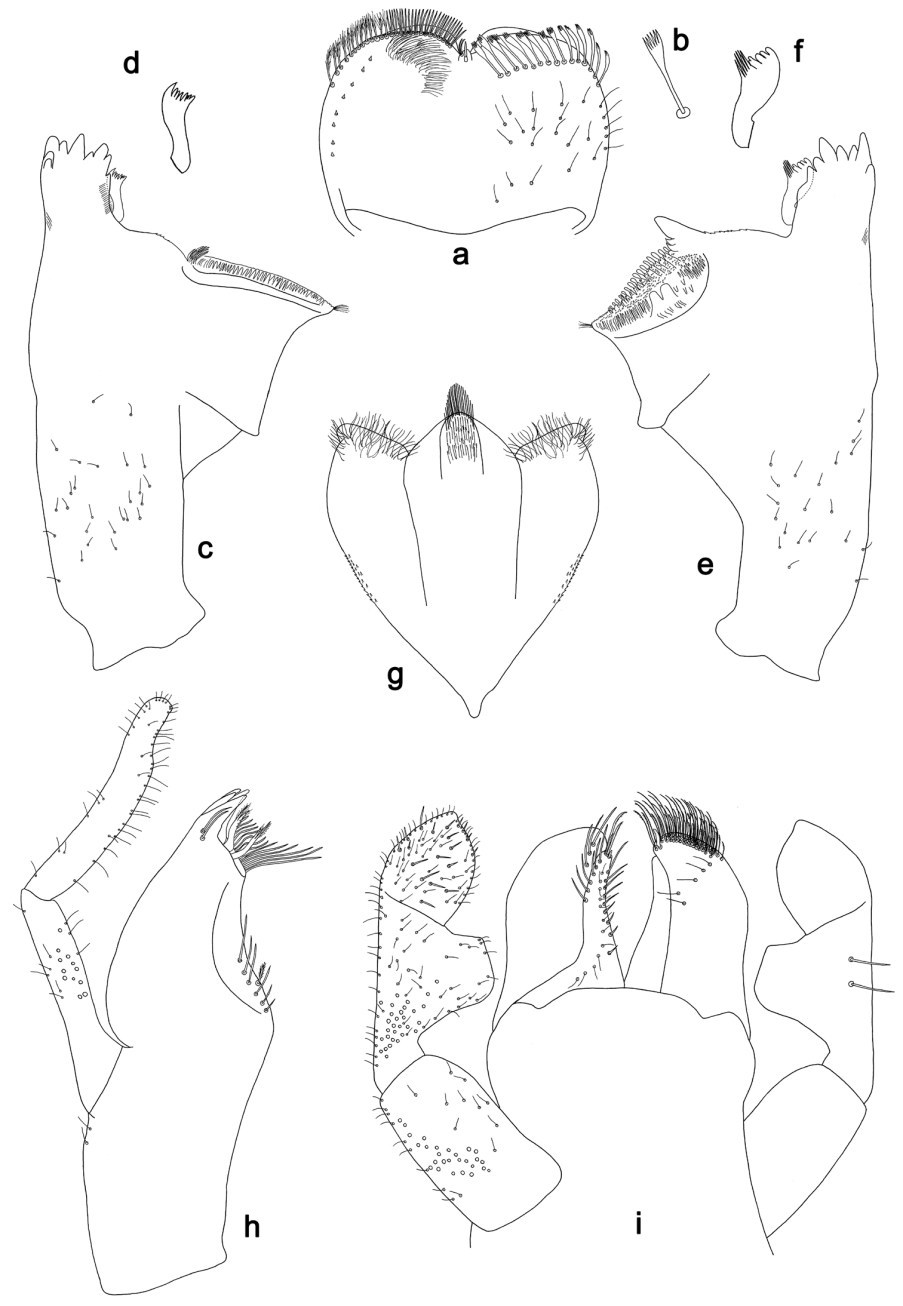
*Labiobaetislobatus* sp. n., larva morphology: **a** Labrum **b** Labrum dorsal, submarginal seta **c** Right mandible **d** Right prostheca **e** Left mandible **f** Left prostheca **g**Hypopharynx**h** Maxilla **i** Labium.

**Figure 5.**
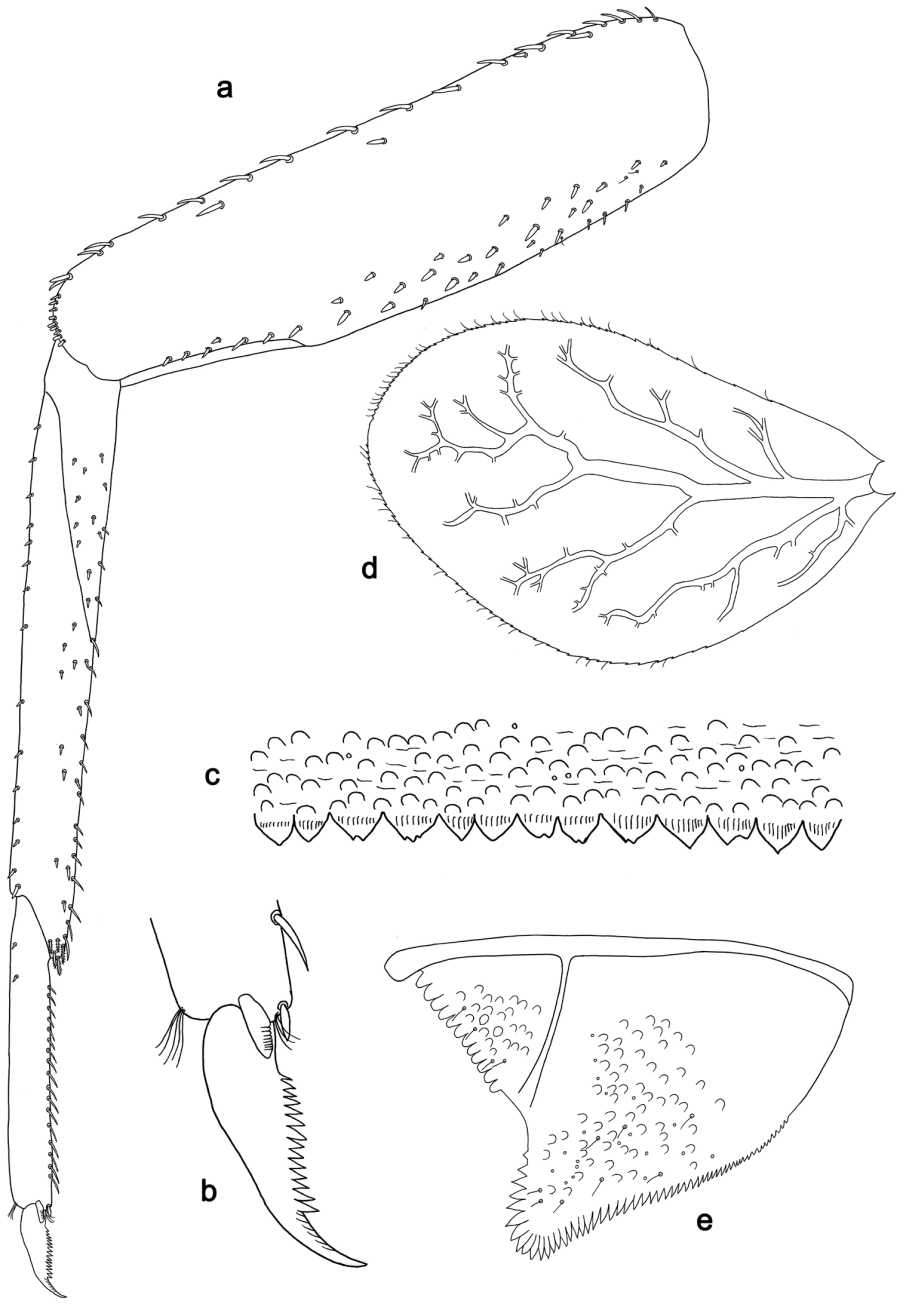
*Labiobaetislobatus* sp. n., larva morphology: **a**Foreleg**b** Fore claw **c**Tergum IV **d** Gill IV **e**Paraproct.

Figures 4 , 5 , 58b , 64a

###### Diagnosis.

**Larva.** Following combination of characters: A) labrum dorsal, submarginal arc of setae composed of 15 spatulate, apically pectinate setae; B) labial palp segment II with a large, lobed distomedial protuberance; C) fore femur length ca. 4× maximum width, dorsal margin with a row of ca. 16 curved, spine-like setae and a few spine-like setae near margin; D) fore claw with a row of 13–14 denticles; E) paraproct distally expanded.

###### Description.

**Larva** (Figs 4, 5, 58b). Body length 5.3 mm; antenna: approximately 2.5× as long as head length.

*Colouration.* Head, thorax and abdomen dorsally brown, thorax with bright, faint pattern as in Fig. 58b. Head and thorax with brighter median, dorsal suture, forewing pads with bright striation. Head, thorax and abdomen ventrally light brown, legs and caudal filaments light brown.

*Antenna* with scape and pedicel sub-cylindrical, without distolateral process at scape; flagellum with lanceolate spines and fine, simple setae on apex of each segment.

*Labrum* (Fig. 4a, b). Rectangular, length 0.7× maximum width. Distal margin with medial emargination and a small process. Dorsally with medium, fine, simple setae scattered over surface; submarginal arc of setae composed of 15 long, spatulate, apically pectinate setae. Ventrally with marginal row of setae composed of lateral and anterolateral long, feathered setae and medial long, bifid setae; ventral surface with eight short, spine-like setae near lateral and anterolateral margin.

*Right mandible* (Fig. 4c, d). Incisors fused. Outer and inner sets of denticles with 4 + 4 denticles. Inner margin of innermost denticle with a row of thin setae. Prostheca robust, apically denticulate. Margin between prostheca and mola slightly convex, with minute denticles. Tuft of setae at apex of mola present.

*Left mandible* (Fig. 4e, f). Incisors fused. Outer and inner sets of denticles with 4 + 3 denticles. Prostheca robust, apically with small denticles and comb-shape structure. Margin between prostheca and mola straight, with minute denticles towards subtriangular process. Subtriangular process long and slender, above level of area between prostheca and mola. Denticles of mola apically constricted. Tuft of setae at apex of mola present.

Both mandibles with lateral margins almost straight. Basal half with fine, simple setae scattered over dorsal surface.

*Hypopharynx* (Fig. 4g). Lingua about as long as superlingua. Lingua longer than broad; medial tuft of stout setae present; distal half not expanded. Superlingua straight; lateral margin rounded; fine, long, simple setae along distal margin.

*Maxilla* (Fig. 4h). Galea-lacinia with two simple, robust apical setae under crown. Inner dorsal row of setae with three denti-setae, distal denti-seta tooth-like, middle and proximal denti-setae slender, bifid and pectinate. Medially with one bipectinate, spine-like seta and six long, simple setae. Maxillary palp 1.5× as long as length of galea-lacinia; two segmented. Palp segment II about as long as segment I. Surface of maxillary palp scattered with fine and simple setae. Apex of last segment rounded, with excavation at inner distolateral margin.

*Labium* (Fig. 4i). Glossa basally broad, narrowing toward apex; shorter than paraglossa; inner margin with nine spine-like setae increasing in length distally; apex with two long, robust, pectinate setae, one medium and one short, robust seta; outer margin with six long spine-like setae increasing in length distally; ventral surface with fine, simple setae. Paraglossa sub-rectangular, curved inward; apex rounded; with three rows of long, robust, apically pectinate setae; dorsally with 4–5 medium, simple setae; ventrally with three long, spine-like setae near inner margin. Labial palp with segment I 0.7× length of segments II and III combined. Segment I covered with short, fine, simple setae ventrally and micropores dorsally. Segment II with a large, lobed distomedial protuberance; distomedial protuberance 0.9× width of base at segment III; inner and outer margin both with short, fine, simple setae; dorsally with two long, spine-like, simple setae. Segment III slightly pentagonal; apex slightly pointed; length 1.2× width; ventrally covered with medium spine-like, simple setae and short, fine, simple setae.

*Hind wing pads* absent.

*Foreleg* (Fig. 5a, b). Ratio of foreleg segments 1.2:1.0:0.5:0.2. *Femur*. Length ca. 3.5× maximum width. Dorsal margin with a row of ca. 16 curved, spine-like setae and a few stout, pointed setae near margin; length of setae 0.18× maximum width of femur. Apex rounded; with one pair of curved, spine-like setae and some short, stout, apically rounded setae. Many stout, lanceolate setae and a few fine, simple setae scattered along the ventral margin; femoral patch poorly developed. *Tibia.* Dorsal margin with a row of short, curved, spine-like setae. Ventral margin with a row of curved, spine-like setae and some bipectinate, spine-like setae on apex. Anterior surface scattered with stout, lanceolate setae. Tibio-patellar suture present on basal 1/2. *Tarsus.* Dorsal margin almost bare. Ventral margin with a row of curved, spine-like setae. Tarsal claw with one row of 13–14 denticles; distally pointed; with six stripes; subapical setae absent.

*Tergum* (Fig. 5c). Surface scattered with U-shaped scale bases and micropores. Posterior margin of tergum IV with triangular spines, wider than long.

*Gills* (Fig. 5d). Present on segments II–VII. Margin with small denticles intercalating fine simple setae. Tracheae extending from main trunk to inner and outer margins. Gill IV as long as length of segments V and 2/3 VI combined. Gill VII as long as length of segments VIII and 2/3 IX combined.

*Paraproct* (Fig. 5e). Distally expanded, with many marginal, stout spines. Surface with U-shaped scale bases and scattered fine, simple setae and micropores. Postero-lateral extension (cercotractor) with small marginal spines.

###### Etymology.

Refers to the large, lobed, distomedial protuberance of labial palp segment II.

###### Distribution.

New Guinea.

###### Biological aspects.

The specimens were collected at an altitude of 1390 m a.s.l.

###### Type-material.

**Holotype.** Nymph (on slide, GBIFCH 00508141), Papua New Guinea, Central, Kokoda Trek, 1390 m, Jan 2008, 09°00.34'S, 147°44.25'E, Posman (PNG 173). Deposited in ZSM. **Paratypes.** 34 nymphs (2 on slides, GBIFCH 00465161, GBIFCH 00465162, 20 in alcohol, GBIFCH 00515232, deposited in MZL; 12 in alcohol, GBIFCH 00515233, deposited in ZSM), same data as holotype.

##### 3. Labiobaetis michaeli sp. n.

Taxon classificationAnimaliaEphemeropteraBaetidae

http://zoobank.org/3162FB93-63BB-45D3-9DC6-8FF9B227F4C0

**Figure 6.**
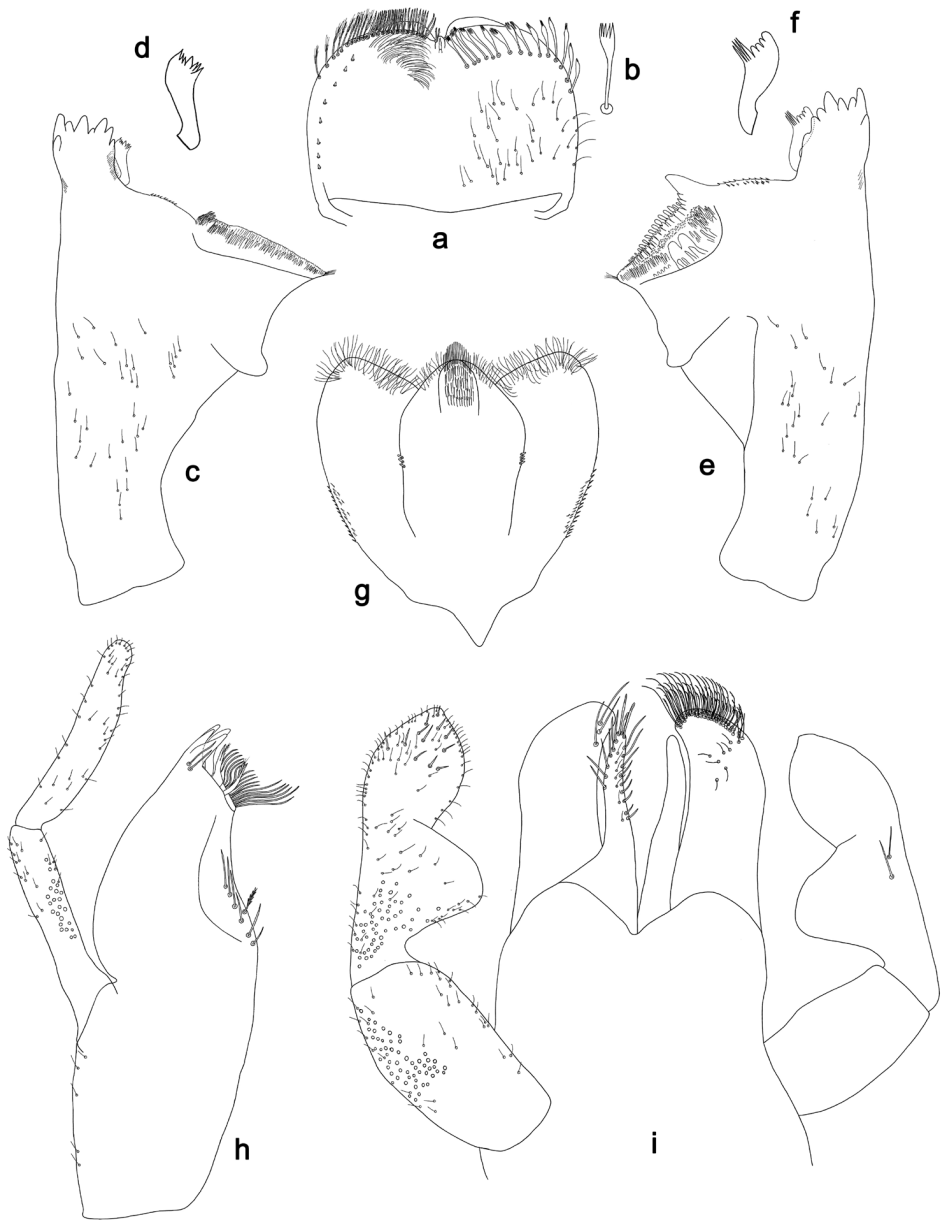
*Labiobaetismichaeli* sp. n., larva morphology: **a** Labrum **b** Seta of the labrum dorsal submarginal arc **c** Right mandible **d** Right prostheca **e** Left mandible **f** Left prostheca **g**Hypopharynx**h** Maxilla **i** Labium.

**Figure 7.**
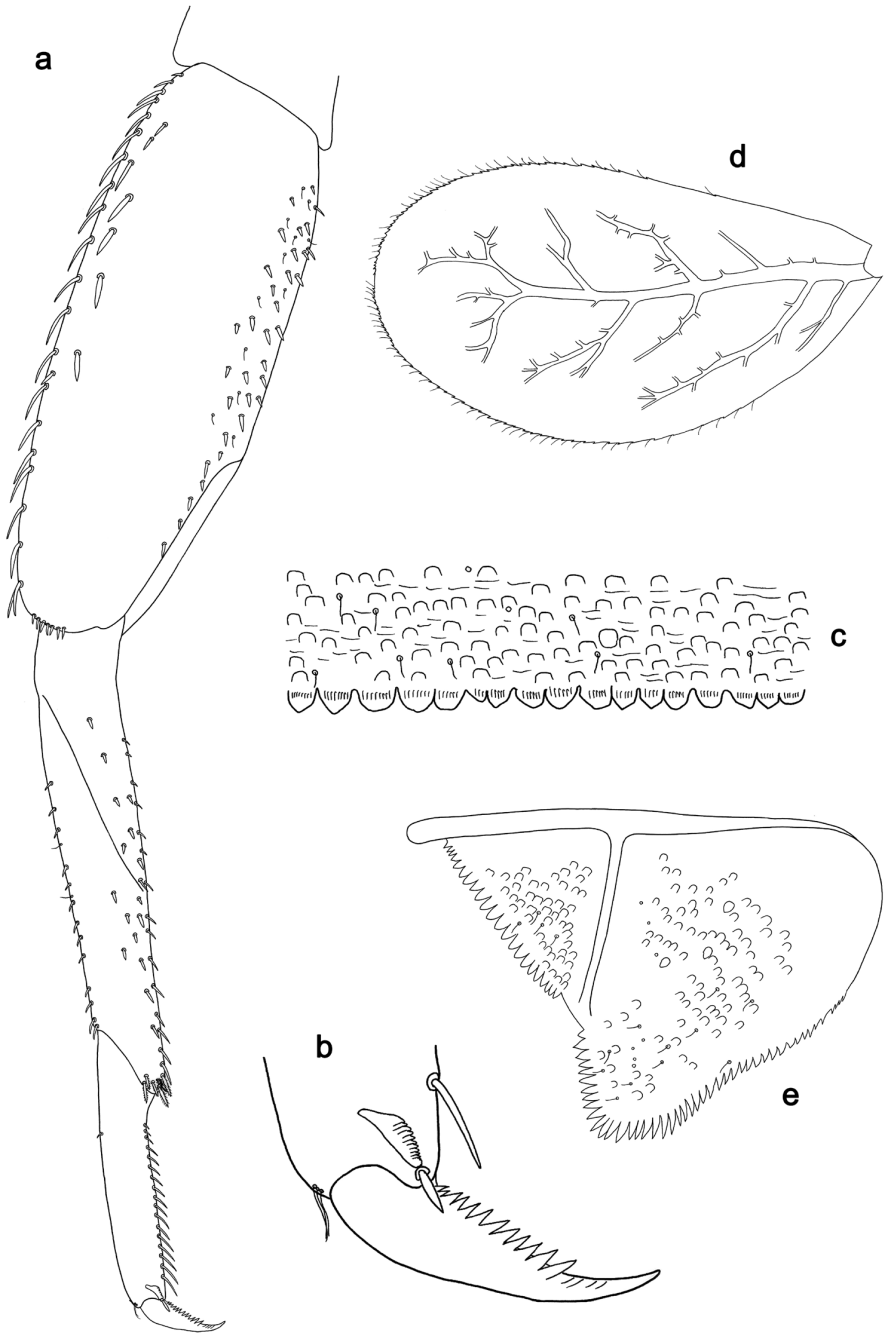
*Labiobaetismichaeli* sp. n., larva morphology: **a**Foreleg**b** Fore claw **c**Tergum IV **d** Gill IV **e**Paraproct.

Figures 6 , 7 , 58c , 64a

###### Diagnosis.

**Larva.** Following combination of characters: A) labrum dorsal, submarginal arc of setae composed of 14–16 spatulate, apically pectinate setae; B) labial palp segment II with a large, lobed distomedial protuberance; C) fore femur rather broad, length ca. 3× maximum width, dorsal margin with a row of ca. 19 curved, spine-like setae and a few spine-like setae near margin; D) fore claw with 11–12 denticles; E) paraproct distally slightly expanded.

###### Description.

**Larva** (Figs 6, 7, 58c). Body length 7.5 mm; antenna: approximately twice as long as head length.

*Colouration.* Head, thorax and abdomen dorsally brown, thorax with bright, faint pattern as in Fig. 58c. Head and thorax with bright median, dorsal suture, forewing pads with bright striation. Head, thorax and abdomen ventrally light brown, legs light brown with brown spots distomedially on femur and proximally on tibia, caudal filaments light brown.

*Antenna* with scape and pedicel sub-cylindrical, without distolateral process at scape; flagellum with lanceolate spines and fine, simple setae on apex of each segment.

*Labrum* (Fig. 6a, b). Rectangular, length 0.7× maximum width. Distal margin with medial emargination and a small process. Dorsally with medium, fine, simple setae scattered over surface; submarginal arc of setae composed of 14–16 long, spatulate, apically pectinate setae.. Ventrally with marginal row of setae composed of lateral and anterolateral long, feathered setae and medial long, bifid, pectinate setae; ventral surface with eight short, spine-like setae near lateral and anterolateral margin.

*Right mandible* (Fig. 6c, d). Incisors fused. Outer and inner sets of denticles with 4 + 4 denticles. Inner margin of innermost denticle with a row of thin setae. Prostheca robust, apically denticulate. Margin between prostheca and mola straight, with minute denticles. Tuft of setae at apex of mola present.

*Left mandible* (Fig. 6e, f). Incisors fused. Outer and inner sets of denticles with 4 + 4 denticles. Prostheca robust, apically with small denticles and comb-shape structure. Margin between prostheca and mola straight, with minute denticles towards subtriangular process. Subtriangular process long and slender, above level of area between prostheca and mola. Denticles of mola apically constricted. Tuft of setae at apex of mola present.

Both mandibles with lateral margins almost straight. Basal half with fine, simple setae scattered over dorsal surface.

*Hypopharynx* (Fig. 6g). Lingua about as long as superlingua. Lingua longer than broad; medial tuft of stout setae present; distal half not expanded. Superlingua straight; lateral margin rounded; fine, long, simple setae along distal margin.

*Maxilla* (Fig. 6h). Galea-lacinia with two simple, robust apical setae under crown. Inner dorsal row of setae with three denti-setae, distal denti-seta tooth-like, middle and proximal denti-setae slender, bifid and pectinate. Medially with one bipectinate, spine-like seta and 5–6 long, simple setae. Maxillary palp 1.4× as long as length of galea-lacinia; two segmented. Palp segment II about as long as segment I. Setae on maxillary palp fine and simple, scattered over surface of segments I and II. Apex of last segment rounded, with excavation at inner distolateral margin.

*Labium* (Fig. 6i). Glossa basally broad, narrowing toward apex; shorter than paraglossa; inner margin with nine spine-like setae increasing in length distally; apex with two long and one medium, robust, pectinate setae; outer margin with six long spine-like setae increasing in length distally; ventral surface with short, fine, simple setae. Paraglossa sub-rectangular, curved inward; apex rounded; with three rows of long, robust, apically pectinate setae; dorsally with 6–8 medium, simple setae; ventrally with three long, spine-like setae near inner margin. Labial palp with segment I 0.8× length of segments II and III combined. Segment I covered with short, fine, simple setae ventrally and micropores dorsally. Segment II with large, lobed distomedial protuberance; distomedial protuberance 0.9× width of base at segment III; inner and outer margin both with short, fine, simple setae; dorsally with two long, spine-like, simple setae. Segment III slightly pentagonal; apex slightly pointed; length 1.3× width; ventrally covered with medium spine-like, simple setae and short, fine, simple setae.

*Hind wing pads* absent.

*Foreleg* (Fig. 7a, b). Ratio of foreleg segments 1.3:1.0:0.6:0.1. *Femur*. Length ca. 3× maximum width. Dorsal margin with a row of ca. 19 curved, spine-like setae and with some stout, pointed setae near margin; length of setae 0.2× maximum width of femur. Apex rounded; with one pair of curved, spine-like setae and some short, stout, pointed setae. Many stout, lanceolate setae and a few fine, simple setae scattered along the ventral margin; femoral patch poorly developed. *Tibia.* Dorsal margin with a row of short, curved, spine-like setae and fine, simple setae. Ventral margin with a row of curved, spine-like setae and some longer, spine-like, bipectinate setae and a tuft of long, fine, simple setae on apex. Anterior surface scattered with stout, lanceolate setae. Tibio-patellar suture present on basal 1/2. *Tarsus.* Dorsal margin almost bare. Ventral margin with a row of curved, spine-like setae. Tarsal claw with one row of 11–12 denticles; distally pointed; with 4–5 stripes; subapical setae absent.

*Tergum* (Fig. 7c). Surface with irregular rows of U-shaped scale bases and scattered fine, simple setae and micropores, scales short, apically rounded. Posterior margin of tergum IV with rounded or pentagonal spines, wider than long.

*Gills* (Fig. 7d). Present on segments II - VII. Margin with small denticles intercalating fine simple setae. Tracheae extending from main trunk to inner and outer margins. Gill IV as long as length of segments V and 1/2 VI combined. Gill VII as long as length of segments VIII and 1/3 IX combined.

*Paraproct* (Fig. 7e). Distally slightly expanded, with many marginal, stout spines. Surface with U-shaped scale bases and scattered fine, simple setae and micropores. Postero-lateral extension (cercotractor) with small marginal spines.

###### Etymology.

Dedicated to Michael Balke (Zoologische Staatssammlung München, ZSM), who collected most of the fantastic material treated in this study.

###### Distribution.

New Guinea.

###### Biological aspects.

The specimens were collected at an altitude of 2200 m a.s.l.

###### Type-material.

**Holotype.** Nymph (on slide, GBIFCH 00508129), Papua New Guinea, Eastern Highlands, Akameku-Brahmin, Bismarck Range, 2200 m, 23 Nov 2006, 05°56.80'S, 145°22.24'E, Balke & Kinibel (PNG 106). Deposited in ZSM. **Paratypes.** 9 nymphs (3 on slides, GBIFCH 00465163, GBIFCH 00465164, GBIFCH 00508130, 4 in alcohol, GBIFCH 00515230, GBIFCH 00508134, deposited in MZL; 2 in alcohol, GBIFCH 00515231, deposited in ZSM), same data as holotype.

#### *L.claudiae* group of species

The group is characterised by an elongated, thumb-like protuberance of labial palp segment II and a dorsal, submarginal arc of setae composed of simple setae. Additionally, the gills have both longer and shorter fine, simple setae at the margin and the femoral patch is well developed.

##### 4. Labiobaetis claudiae sp. n.

Taxon classificationAnimaliaEphemeropteraBaetidae

http://zoobank.org/F8721E8C-74C6-4D77-BCB4-E78C166EB58D

**Figure 8.**
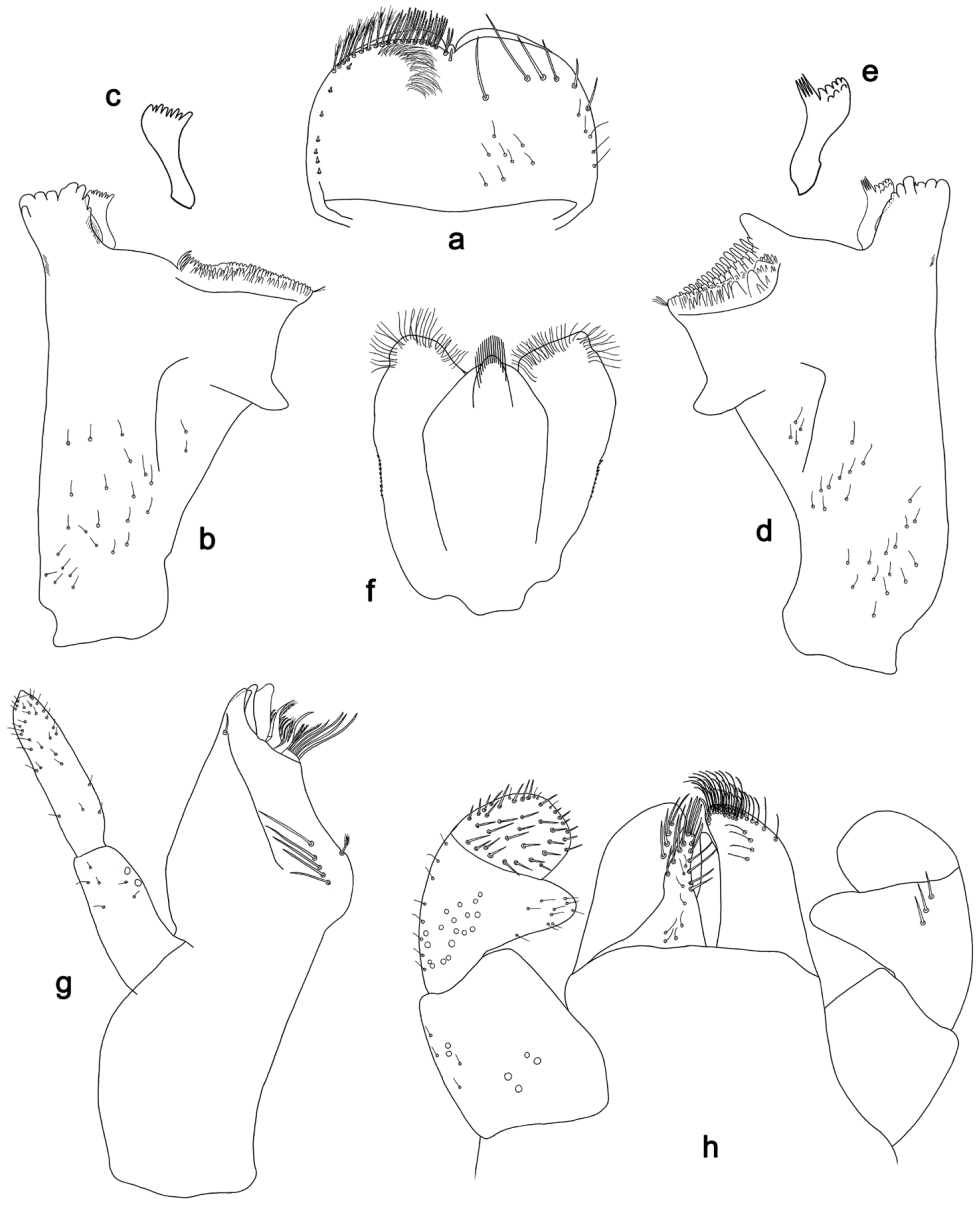
*Labiobaetisclaudiae* sp. n., larva morphology: **a** Labrum **b** Right mandible **c** Right prostheca **d** Left mandible **e** Left prostheca **f**Hypopharynx**g** Maxilla **h** Labium.

**Figure 9.**
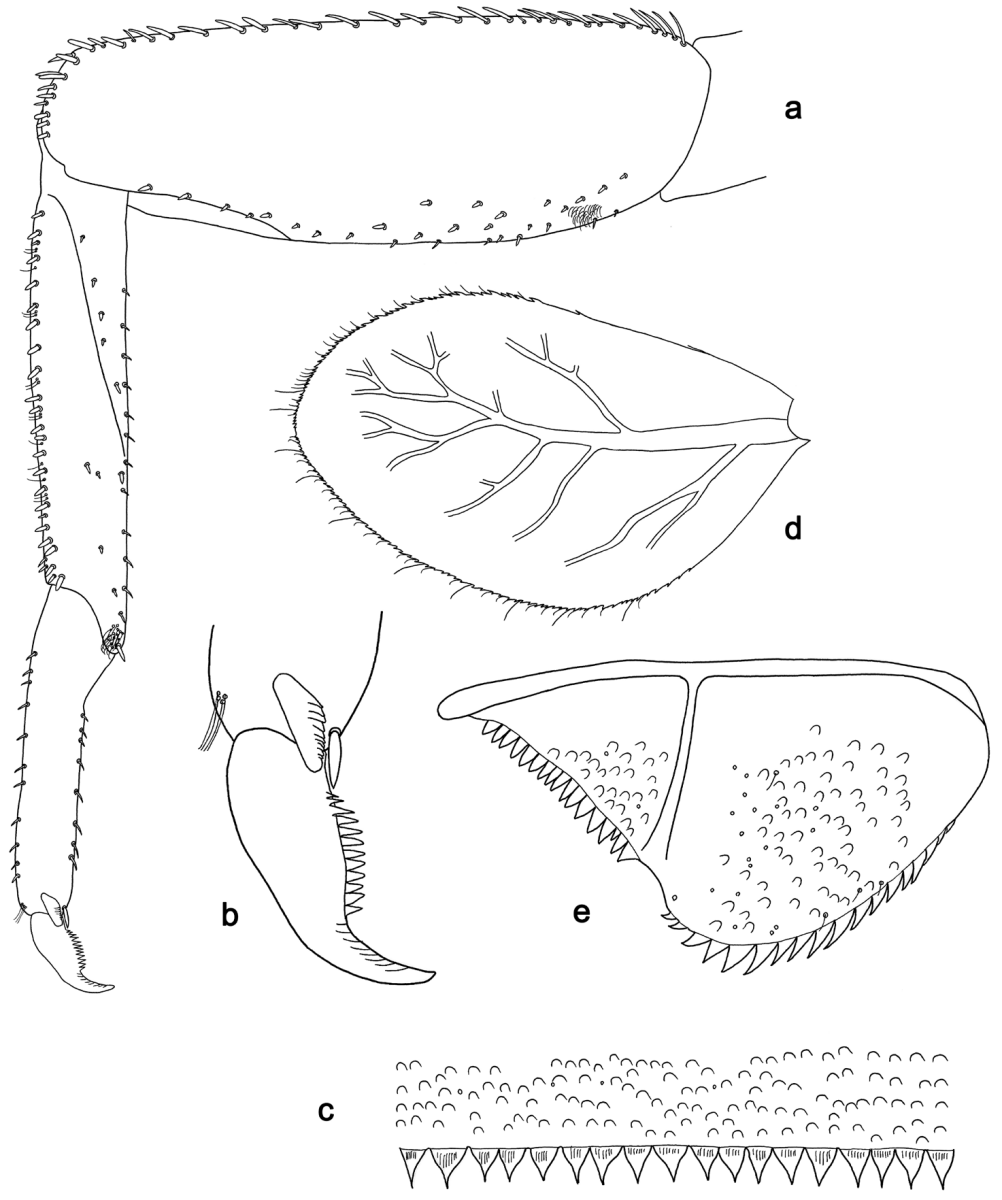
*Labiobaetisclaudiae* sp. n., larva morphology: **a**Foreleg**b** Fore claw **c**Tergum IV **d** Gill IV **e**Paraproct.

Figures 8 , 9 , 58d , 64a

###### Diagnosis.

**Larva.** Following combination of characters: A) labrum dorsal submarginal arc of setae composed of one plus five long, simple setae; B) labial palp segment II with an elongated thumb-like distomedial protuberance, segment III subrectangular; C) fore femur rather broad, length ca. 3× width, dorsal margin with a row of ca. 27 curved, spine-like setae; D) gills with small denticles and both medium and long, fine simple setae on margin; E) paraproct distally not expanded, with ca. 18 marginal, stout spines.

###### Description.

**Larva** (Figs 8, 9, 58d). Body length 5.3 mm; antenna: approximately twice as long as head length.

*Colouration.* Head, thorax and abdomen dorsally brown, head and thorax with bright median, dorsal suture, thorax with bright pattern as in Fig. 58d, abdominal segments I, V, VI, IX and X light brown, other segments dark brown. Head, thorax and abdomen ventrally light brown, legs transparent with brown spots distomedially on femur and proximally on tibia and tarsus, caudal filaments brown.

*Antenna* with scape and pedicel sub-cylindrical, without distolateral process at scape; flagellum with broad, lanceolate spines and fine, simple setae on apex of each segment.

*Labrum* (Fig. 8a). Rectangular, length 0.6× maximum width. Distal margin with medial emargination and a small process. Dorsally with medium, fine, simple setae scattered over surface; submarginal arc of setae composed of one plus five long, simple setae. Ventrally with marginal row of setae composed of anterolateral long, feathered setae and medial long, bifid, pectinate setae; ventral surface with seven short, spine-like setae near lateral and anterolateral margin.

*Right mandible* (Fig. 8b, c). Incisors fused. Outer and inner sets of denticles with 4 + 4 denticles. Inner margin of innermost denticle with a row of thin setae. Prostheca robust, apically denticulate. Margin between prostheca and mola straight. Tuft of setae at apex of mola present.

*Left mandible* (Fig. 8d, e). Incisors fused. Outer and inner sets of denticles with 3 + 3 denticles and one minute intermediate denticle. Prostheca robust, apically with small denticles and comb-shape structure. Margin between prostheca and mola straight. Subtriangular process long and slender, above level of area between prostheca and mola. Denticles of mola apically constricted. Tuft of setae at apex of mola present.

Both mandibles with lateral margins almost straight. Basal half with fine, simple setae scattered over dorsal surface.

*Hypopharynx* (Fig. 8f). Lingua shorter than superlingua. Lingua longer than broad; medial tuft of stout setae present; distal half laterally expanded. Superlingua straight; lateral margin straight; fine, long, simple setae along distal margin.

*Maxilla* (Fig. 8g). Galea-lacinia with one simple, robust apical seta under crown. Inner dorsal row of setae with three denti-setae, distal denti-seta tooth-like, middle and proximal denti-setae slender, bifid and pectinate. Medially with one bipectinate, spine-like seta and 5–6 long, simple setae. Maxillary palp slightly longer than length of galea-lacinia; two segmented. Palp segment II 1.3× length of segment I. Setae on maxillary palp fine and simple, scattered over surface of segments I and II. Apex of last segment constricted, without excavation at inner distolateral margin.

*Labium* (Fig. 8h). Glossa basally broad, narrowing toward apex; shorter than paraglossa; inner margin with six spine-like setae increasing in length distally; apex with three long, robust setae; outer margin with 4–5 long, spine-like setae increasing in length distally; ventral surface with fine, simple, scattered setae. Paraglossa sub-rectangular, curved inward, apex rounded; ventrally with three rows of long, robust, distally pectinate setae in apical area and a row of 3–4 medium, simple setae in anteromedial area; dorsally with a row of 3–4 long, spine-like setae near inner margin. Labial palp with segment I 0.8× length of segments II and III combined. Segment I covered with short, fine, simple setae ventrally and micropores dorsally. Segment II with an elongated, thumb-like distomedial protuberance; distomedial protuberance 0.4× width of base of segment III; inner and outer margin both with short, fine, simple setae; dorsally with row of three long, spine-like setae in anteromedial area. Segment III subrectangular; apex truncate; length 0.9× width; ventrally covered with medium spine-like, simple setae and short, fine, simple setae.

*Hind wing pads* absent.

*Foreleg* (Fig. 9a, b). Ratio of foreleg segments 1.3:1.0:0.6:0.2. *Femur*. Length ca. 3× maximum width. Dorsal margin with a row of ca. 27 curved, spine-like setae; length of setae 0.16× maximum width of femur. Apex rounded; with two pairs of curved, spine-like setae and some short, stout setae. Many stout, lanceolate setae scattered along the ventral margin; femoral patch well developed. *Tibia.* Dorsal margin with a row of stout, lanceolate setae and very fine, simple setae. Ventral margin with a row of curved, spine-like setae, on apex one stout, spine-like seta and a tuft of long, fine, simple setae. Anterior surface scattered with stout, lanceolate setae. Tibio-patellar suture present on basal 2/3 area. *Tarsus.* Dorsal margin with a row of short, curved, spine-like setae. Ventral margin with a row of curved, spine-like setae. Tarsal claw with one row of 12–13 denticles; distally pointed; with eight stripes; subapical setae absent.

*Tergum* (Fig. 9c). Surface with irregular rows of U-shaped scale bases and scattered micropores. Posterior margin of tergum IV with triangular spines, longer than wide.

*Gills* (Fig. 9d). Present on segments II–VII. Margin with small denticles intercalating both medium and long, fine, simple setae. Tracheae extending from main trunk to inner and outer margins. Gill IV as long as length of segments V and 2/3 VI combined. Gill VII as long as length of segments VIII and IX combined.

*Paraproct* (Fig. 9e). Distally not expanded, with ca. 18 marginal, stout spines. Surface scattered with U-shaped scale bases and fine, simple setae and micropores. Postero-lateral extension (cercotractor) with small marginal spines.

###### Etymology.

Dedicated to Claudia Kaltenbach, the wife of one of the authors (TK) for her constant support during the study.

###### Distribution.

New Guinea.

###### Biological aspects.

The specimens were collected in a ford at an altitude of 80 m a.s.l.

###### Type-material.

**Holotype.** Nymph (on slide, GBIFCH 00508144), Papua New Guinea, Madang, highway nr Madang, ford, 80 m, 26 Nov and 2–3 Dec 2006, 05°24.41'S, 145°38.21'E, Binatang Boys (PNG 117). Deposited in ZSM. **Paratypes.** 4 nymphs (2 on slide, GBIFCH 00508145, GBIFCH 00465165, 2 in alcohol, GBIFCH 00515229, deposited in MZL), same data as holotype.

##### 5. Labiobaetis stagnum sp. n.

Taxon classificationAnimaliaEphemeropteraBaetidae

http://zoobank.org/4908A8A5-CF14-4A0D-B4E1-50712943B186

**Figure 10.**
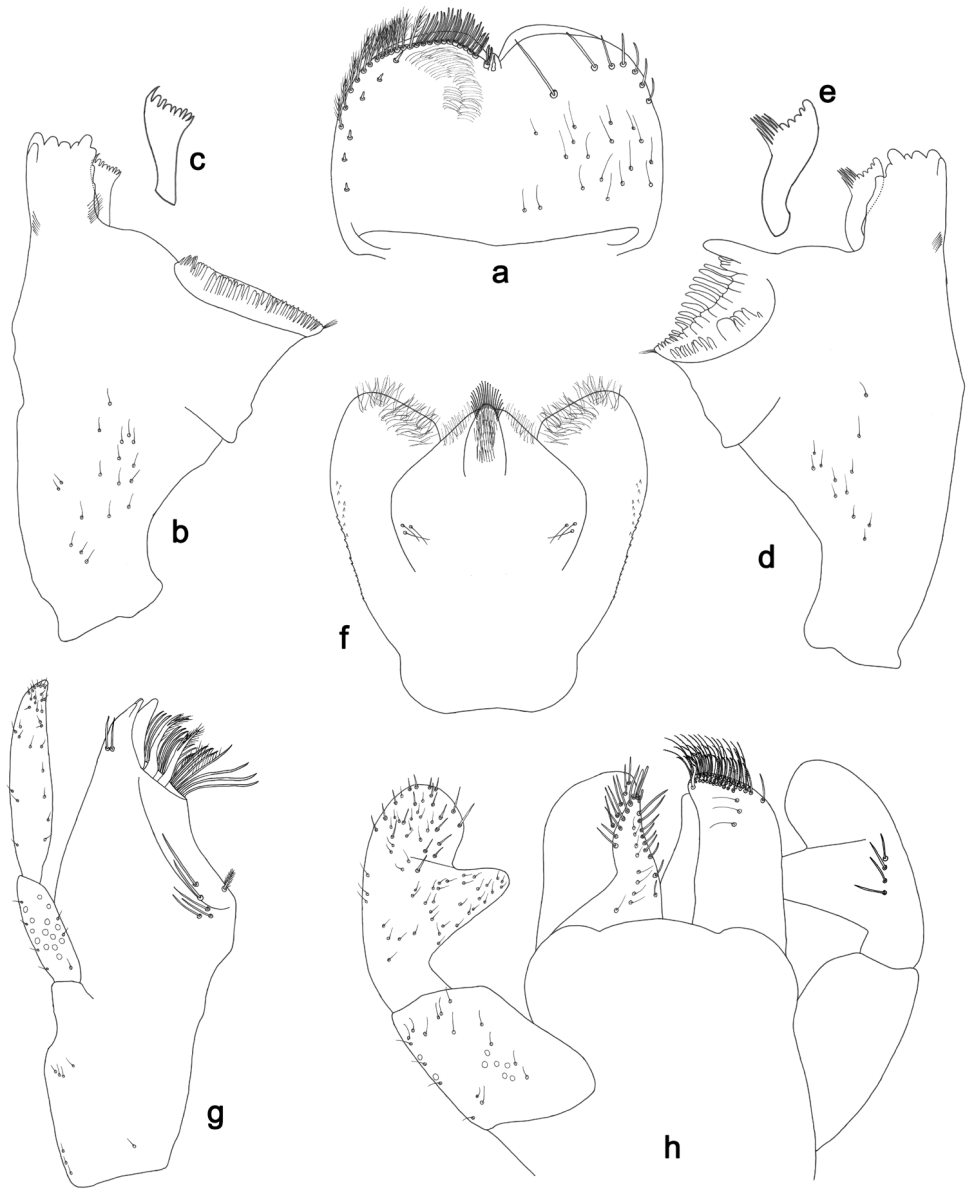
*Labiobaetisstagnum* sp. n., larva morphology: **a** Labrum **b** Right mandible **c** Right prostheca **d** Left mandible **e** Left prostheca **f**Hypopharynx**g** Maxilla **h** Labium.

**Figure 11.**
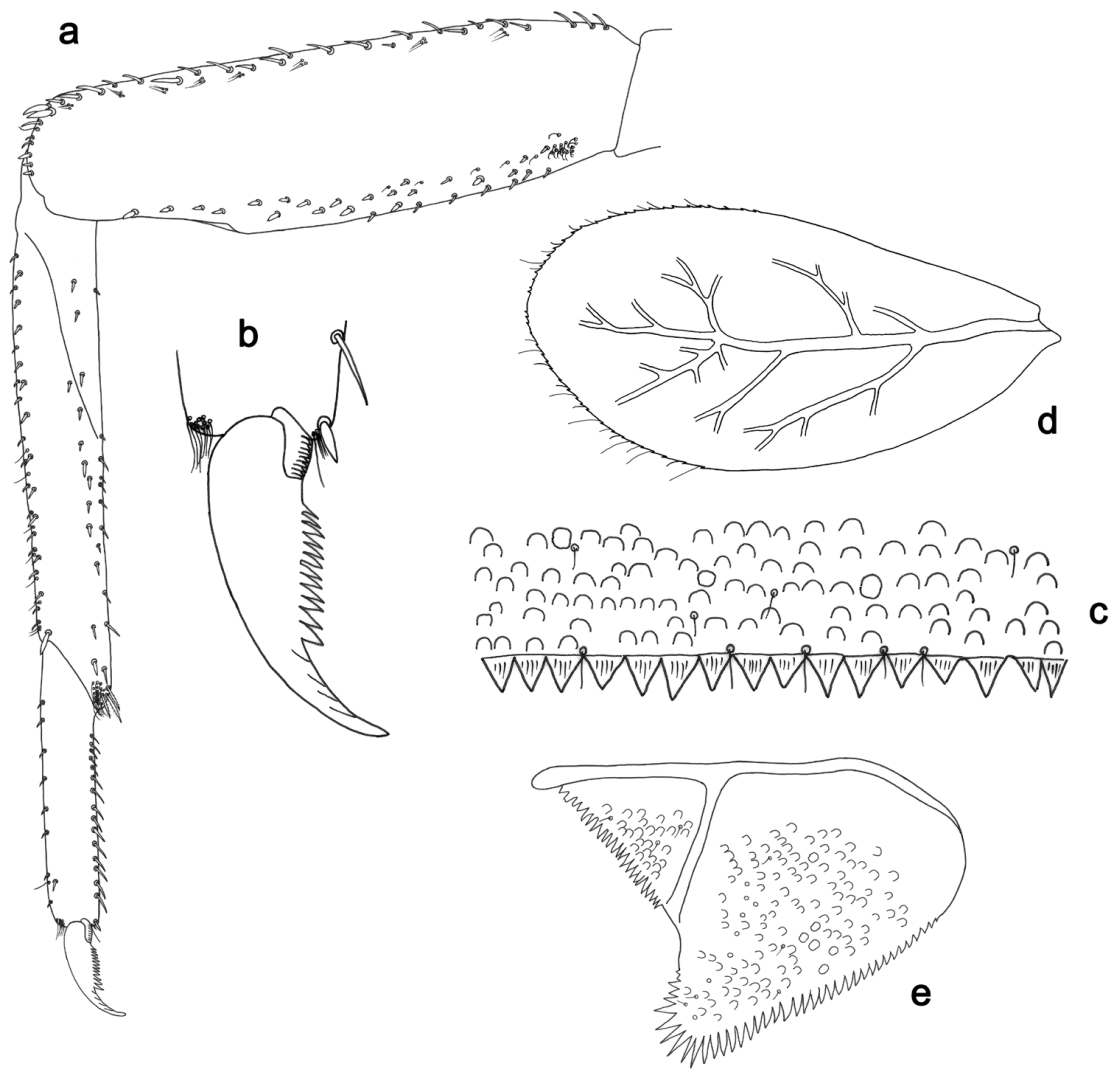
*Labiobaetisstagnum* sp. n., larva morphology: **a**Foreleg**b** Fore claw **c**Tergum IV **d** Gill IV **e**Paraproct.

Figures 10 , 11 , 59a , 64a

###### Diagnosis.

**Larva.** Following combination of characters: A) labrum dorsal submarginal arc of setae composed of one plus six long, simple setae; B) maxillary palp about as long as length of galea-lacinia; C) labial palp segment II with an elongated, thumb-like distomedial protuberance; D) fore femur rather broad, length ca. 3× maximum width, dorsal margin with a row of ca. 18 curved, spine-like setae on margin and a few stout, pointed setae near margin; E) fore claw with 10–12 denticles.

###### Description.

**Larva** (Figs 10, 11, 59a). Body length 4.5 mm; antenna: approximately twice as long as head length.

*Colouration.* Head, thorax and abdomen dorsally brown, head and thorax with bright median, dorsal suture, head, thorax and abdomen with bright pattern as in Fig. 59a, forewing pads with brown striation. Head, thorax and abdomen ventrally light brown, legs light brown with a brown spot distomedially on femur and medially on tibia, dorsal margin of femur brown, tarsus proximally brown. Caudal filaments light brown with a dark brown section at 3/4 of length of cerci, cerci with 2^nd^ dark brown section near the tip.

*Antenna* with scape and pedicel sub-cylindrical, without distolateral process at scape; flagellum with broad, lanceolate spines and fine, simple setae on apex of each segment.

*Labrum* (Fig. 10a). Rectangular, length 0.6× maximum width. Distal margin with medial emargination and a small process. Dorsally with many medium to long, fine, simple setae in posterior area; submarginal arc of setae composed of one plus 5–6 long, simple setae. Ventrally with marginal row of setae composed of lateral and anterolateral long, pectinate setae and medial long, bifid, pectinate setae; ventral surface with six short, spine-like setae near lateral and anterolateral margin.

*Right mandible* (Fig. 10b, c). Incisors fused. Outer and inner sets of denticles with 4 + 4 denticles. Inner margin of innermost denticle with a row of thin setae. Prostheca robust, apically denticulate. Margin between prostheca and mola slightly convex. Tuft of setae at apex of mola present.

*Left mandible* (Fig. 10d, e). Incisors fused. Outer and inner sets of denticles with 4 + 3 (sometimes four) denticles. Prostheca robust, apically with small denticles and comb-shape structure. Margin between prostheca and mola slightly convex. Subtriangular process long and slender, above level of area between prostheca and mola. Denticles of mola apically constricted. Tuft of setae at apex of mola present.

Both mandibles with lateral margins almost straight. Basal half with fine, simple setae scattered over dorsal surface.

*Hypopharynx* (Fig. 10f). Lingua shorter than superlingua. Lingua about as broad as long; medial tuft of stout setae present; distal half laterally expanded. Superlingua rounded; lateral margin straight; fine, long, simple setae along distal margin.

*Maxilla* (Fig. 10g). Galea-lacinia with two simple, robust apical setae under crown. Inner dorsal row of setae with three denti-setae, distal denti-seta tooth-like, middle and proximal denti-setae slender, bifid and pectinate. Medially with one bipectinate, spine-like seta and 5–6 long, simple setae. Maxillary palp slightly longer than length of galea-lacinia; two segmented. Palp segment II 1.9× length of segment I. Setae on maxillary palp fine and simple, scattered over surface of segments I and II. Apex of last segment constricted, without excavation at inner distolateral margin.

*Labium* (Fig. 10h). Glossa basally broad, narrowing toward apex; shorter than paraglossa; inner margin with 8–9 spine-like setae increasing in length distally; apex with two long and one short, robust, pectinate setae; outer margin with seven spine-like setae increasing in length distally; ventral surface with fine, simple, scattered setae. Paraglossa sub-rectangular, curved inward; apex rounded; with three rows of long, robust, apically pectinate setae: dorsally with three medium, simple setae; ventrally with five long, spine-like setae near inner margin. Labial palp with segment I 0.8× length of segments II and III combined. Segment I covered with short, fine, simple setae ventrally and micropores dorsally. Segment II with an elongated, thumb-like distomedial protuberance; distomedial protuberance 0.6× width of base of segment III; inner and outer margin both with short, fine, simple setae; dorsally with row of 4–5 long, spine-like, simple setae. Segment III semicircular; apex rounded; length 0.8× width; ventrally covered with short and medium spine-like, simple setae and short, fine, simple setae.

*Hind wing pads* absent.

*Foreleg* (Fig. 11a, b). Ratio of foreleg segments 1.2:1.0:0.5:0.2. *Femur*. Length ca. 3× maximum width. Dorsal margin with a row of ca. 17 curved, spine-like setae and a few stout, pointed setae near margin; length of setae 0.14× maximum width of femur. Apex rounded; with one pair of spine-like setae and some short, stout, pointed or apically rounded setae. Many stout, lanceolate setae and a few fine, simple setae along ventral margin; femoral patch well developed. *Tibia.* Dorsal margin with row of short, curved, spine-like setae and long, fine, simple setae and a row of stout, lanceolate setae near margin; one larger, robust, pointed seta on apex. Ventral margin with a row of curved, spine-like setae and some longer, spine-like, bipectinate setae and a tuft of long, fine, simple setae on apex. Anterior surface scattered with stout, lanceolate setae. Tibio-patellar suture present on basal 1/2. *Tarsus.* Dorsal margin with a row of short, curved, spine-like setae. Ventral margin with a row of curved, spine-like setae. Tarsal claw with one row of 10–12 denticles; distally pointed; with four stripes; subapical setae absent.

*Tergum* (Fig. 11c). Surface with irregular rows of U-shaped scale bases and scattered with fine, simple setae, scales short and apically rounded. Posterior margin of tergum IV with triangular spines, longer than wide.

*Gills* (Fig. 11d). Present on segments II–VII. Margin with small denticles intercalating both medium and long, fine, simple setae. Tracheae extending from main trunk to inner and outer margins. Gill IV as long as length of segments V and 2/3 VI combined. Gill VII as long as length of segments VIII and 1/2 IX combined.

*Paraproct* (Fig. 11e). Distally expanded, with many marginal, stout spines. Surface with U-shaped scale bases and scattered fine, simple setae and micropores. Postero-lateral extension (cercotractor) with small marginal spines.

###### Etymology.

Latin word for pool, refers to the appearance in stream pools.

###### Distribution.

New Guinea.

###### Biological aspects.

The specimens were collected in stream pools at an altitude of 115 m a.s.l.

###### Type-material.

**Holotype.** Nymph (on slide, GBIFCH 00465166), Indonesia, Papua, Setani-Maribu, stream pools, 115 m, 14 Oct 2011, 02°30.51'S, 140°22.83'E, Balke (PAP01). Temporary deposited in MZL before definitely housed in MZB. **Paratypes.** 9 nymphs (2 on slides, GBIFCH 00465167, GBIFCH 00465168, deposited in MZL, 5 in alcohol, GBIFCH 00515227, deposited in MZL; 2 in alcohol, GBIFCH 00515228, deposited in ZSM), same data as holotype.

#### *L.orientis* group of species

The group is characterised by a large, lobed, distomedial protuberance of labial palp segment II and a dorsal, submarginal arc of setae of the labrum composed of feathered setae.

##### 6. Labiobaetis orientis sp. n.

Taxon classificationAnimaliaEphemeropteraBaetidae

http://zoobank.org/25115C5B-2F6D-4B0A-92D3-00B6239FC9DA

**Figure 12.**
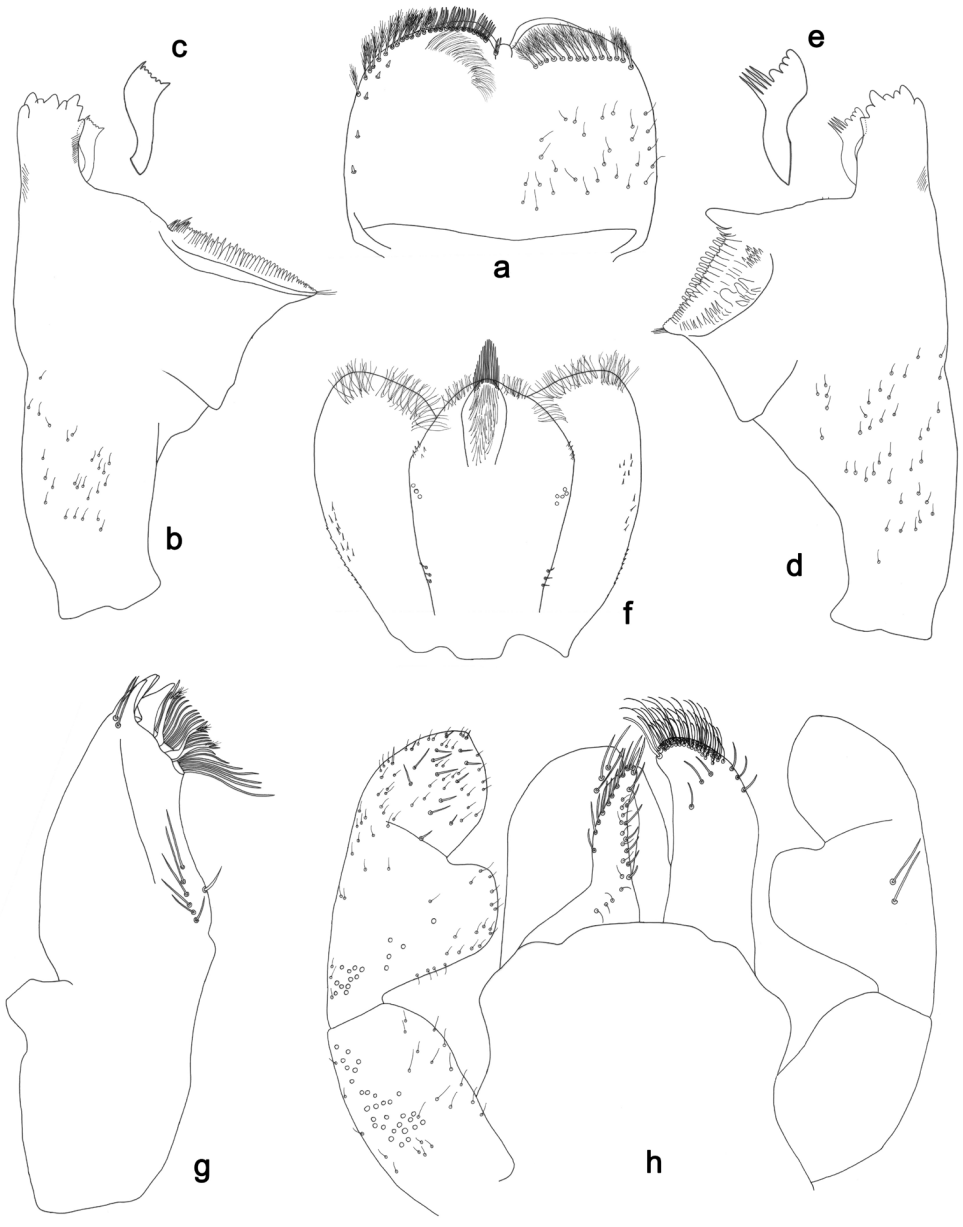
*Labiobaetisorientis* sp. n., larva morphology: **a** Labrum **b** Right mandible **c** Right prostheca **d** Left mandible **e** Left prostheca **f**Hypopharynx**g** Maxilla **h** Labium.

**Figure 13.**
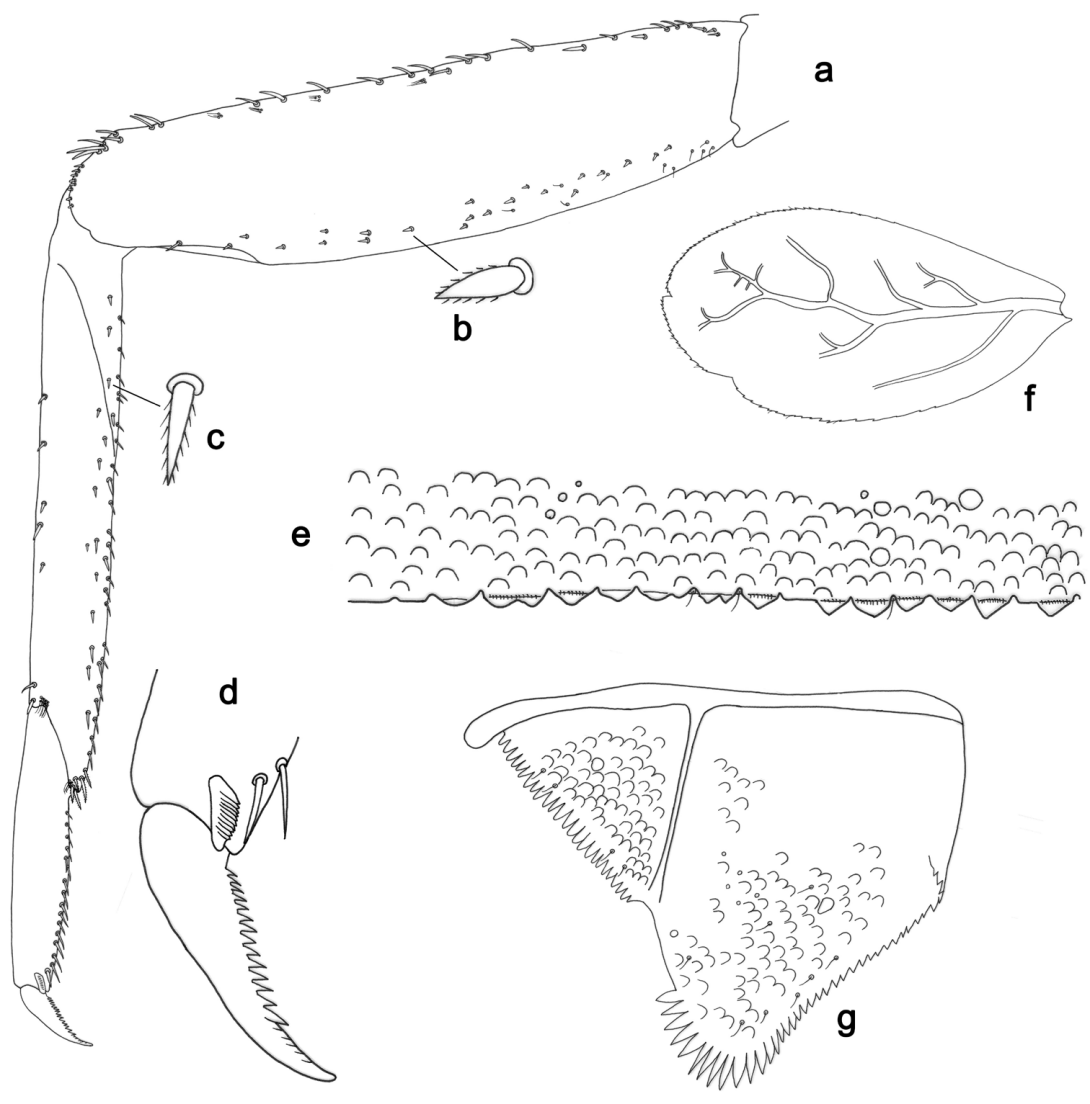
*Labiobaetisorientis* sp. n., larva morphology: **a** Hind leg **b** Femur ventral seta **c** Tibia ventral seta **d** Hind claw **e**Tergum IV **f** Gill V **g**Paraproct.

Figures 12 , 13 , 64a

###### Diagnosis.

**Larva.** Following combination of characters: A) labrum dorsal submarginal arc of setae composed of 14 long, feathered setae; B) labial palp segment II with a large, lobed distomedial protuberance and with segment III slightly pentagonal and slightly pointed on apex; C) hind femur slender, length ca. 4× maximum width, dorsal margin with a row of ca. 18 curved, spine-like setae and a few stout, pointed setae near margin; D) femur and tibia of hind leg ventrally with stout, bipectinate, pointed setae; E) hind tarsal claw with 14 denticles.

###### Description.

**Larva** (Figs 12, 13).

*Colouration.* Unknown.

*Antenna* with scape and pedicel sub-cylindrical, without distolateral process at scape; flagellum with lanceolate spines and fine, simple setae on apex of each segment.

*Labrum* (Fig. 12a). Rectangular, length 0.7× maximum width. Distal margin with medial emargination and a small process. Dorsally with many medium to long, fine, simple setae; submarginal arc of setae composed of 14 long, feathered setae. Ventrally with marginal row of setae composed of lateral and anterolateral long, feathered setae and medial long, bifid, pectinate setae; ventral surface with five short, spine-like setae near lateral and anterolateral margin.

*Right mandible* (Fig. 12b, c). Incisors fused. Outer and inner sets of denticles with 4 + 4 denticles. Inner margin of innermost denticle with a row of thin setae. Prostheca robust, apically denticulate. Margin between prostheca and mola slightly convex. Tuft of setae at apex of mola present.

*Left mandible* (Fig. 12d, e). Incisors fused. Outer and inner sets of denticles with 4 + 4 denticles. Prostheca robust, apically with small denticles and comb-shape structure. Margin between prostheca and mola straight, with few minute setae. Subtriangular process long and slender, above level of area between prostheca and mola. Denticles of mola apically constricted. Tuft of setae at apex of mola present.

Both mandibles with lateral margins almost straight. Basal half with fine, simple setae scattered over dorsal surface.

*Hypopharynx* (Fig. 12f). Lingua about as long as superlingua. Lingua longer than broad; medial tuft of stout setae present; distal half laterally expanded. Superlingua rounded; lateral margin rounded; fine, long, simple setae along distal margin.

*Maxilla* (Fig. 12g). Galea-lacinia with two simple, robust apical setae under crown. Inner dorsal row of setae with three denti-setae, distal denti-seta tooth-like, middle and proximal denti-setae slender, bifid and pectinate. Medially with one spine-like seta and six long, simple setae. Maxillary palp unknown.

*Labium* (Fig. 12h). Glossa basally broad, narrowing toward apex; shorter than paraglossa; inner margin with seven spine-like setae increasing in length distally; apex with two long and one medium, robust, pectinate setae; outer margin with eight long, spine-like setae; ventral surface with short, fine, simple setae. Paraglossa sub-rectangular, curved inward; apex rounded; with three rows of long, robust, apically pectinate setae; dorsally with three medium, simple setae; ventrally with three long, spine-like setae near inner margin. Labial palp with segment I 0.7× length of segments II and III combined. Segment I covered with short and medium, fine, simple setae ventrally and with micropores dorsally. Segment II with a large, lobed distomedial protuberance; distomedial protuberance 0.8× width of base of segment III; inner and outer margin both with short, fine, simple setae; dorsally with two long, spine-like, simple setae. Segment III slightly pentagonal; apex slightly pointed; length 1.1× width; ventrally covered with medium spine-like, simple setae and short, fine, simple setae.

*Hind wing pads* absent.

*Hind leg* (Fig. 13a, b, c, d). Ratio of hind leg segments 1.3:1.0:0.5:0.2. *Hind femur*. Length ca. 4× maximum width. Dorsal margin with a row of ca. 18 curved, spine-like setae and with a few stout, pointed setae near margin; length of setae 0.16× maximum width of femur. Apex rounded; with some curved spine-like setae and many short, stout setae. Many stout, lanceolate, bipectinate setae and a few fine, simple setae along ventral margin; femoral patch poorly developed. *Tibia.* Dorsal margin with a few curved, spine-like setae. Ventral margin with a row of curved, spine-like setae and some longer, spine-like, bipectinate setae and a tuft of long, fine, simple setae on apex. Anterior surface scattered with stout, lanceolate, bipectinate setae (pectination difficult to see). Tibio-patellar suture present on basal 1/3 area. *Tarsus.* Dorsal margin bare. Ventral margin with a row of curved, spine-like setae. Tarsal claw with one row of 14 denticles; tapering distally; with five stripes; subapical setae absent.

*Tergum* (Fig. 13e). Surface with irregular rows of U-shaped scale bases and scattered micropores. Posterior margin of tergum IV with triangular spines, wider than long.

*Gills* (Fig. 13f). Present on segments II–VII. Margin with small denticles and long, fine, simple setae. Tracheae extending from main trunk to inner and outer margins. Gill IV unknown, gill V as long as length of segments VI and 1/2 VII combined. Gill VII as long as length of segments VIII and 1/2 IX combined.

*Paraproct* (Fig. 13g). Distally expanded, with many marginal, stout spines. Surface with U-shaped scale bases and scattered fine, simple setae and micropores. Postero-lateral extension (cercotractor) with small marginal spines.

###### Etymology.

Refers to the type locality, which is in the Eastern Highlands of Papua New Guinea.

###### Distribution.

New Guinea.

###### Biological aspects.

The specimen was collected at an altitude of 1700–1800 m a.s.l.

###### Type-material.

**Holotype.** Nymph (on slide, GBIFCH 00465169), Papua New Guinea, Eastern Highlands, Marawaka, Ande, 1700–1800 m, 09 Nov 2006, nr 07°01.70'S, 145°49.81'E, Balke & Kinibel (PNG 87). Deposited in ZSM.

##### 7. Labiobaetis papuaensis sp. n.

Taxon classificationAnimaliaEphemeropteraBaetidae

http://zoobank.org/398A4E16-2A9F-42E2-B885-9E116B6A84C8

**Figure 14.**
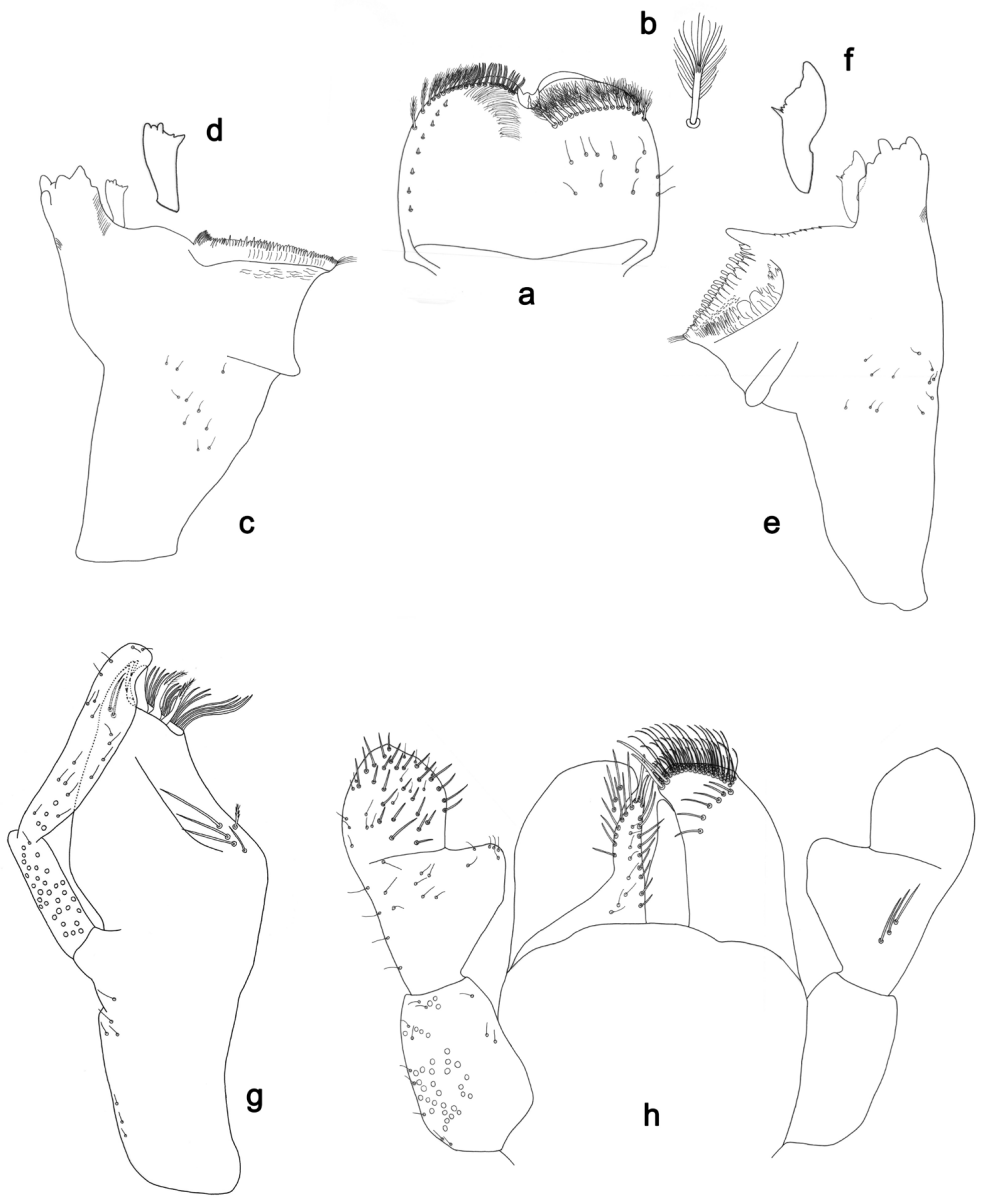
*Labiobaetispapuaensis* sp. n., larva morphology: **a** Labrum **b** Labrum dorsal, submarginal seta **c** Right mandible **d** Right prostheca **e** Left mandible **f** Left prostheca **g** Maxilla **h** Labium.

**Figure 15.**
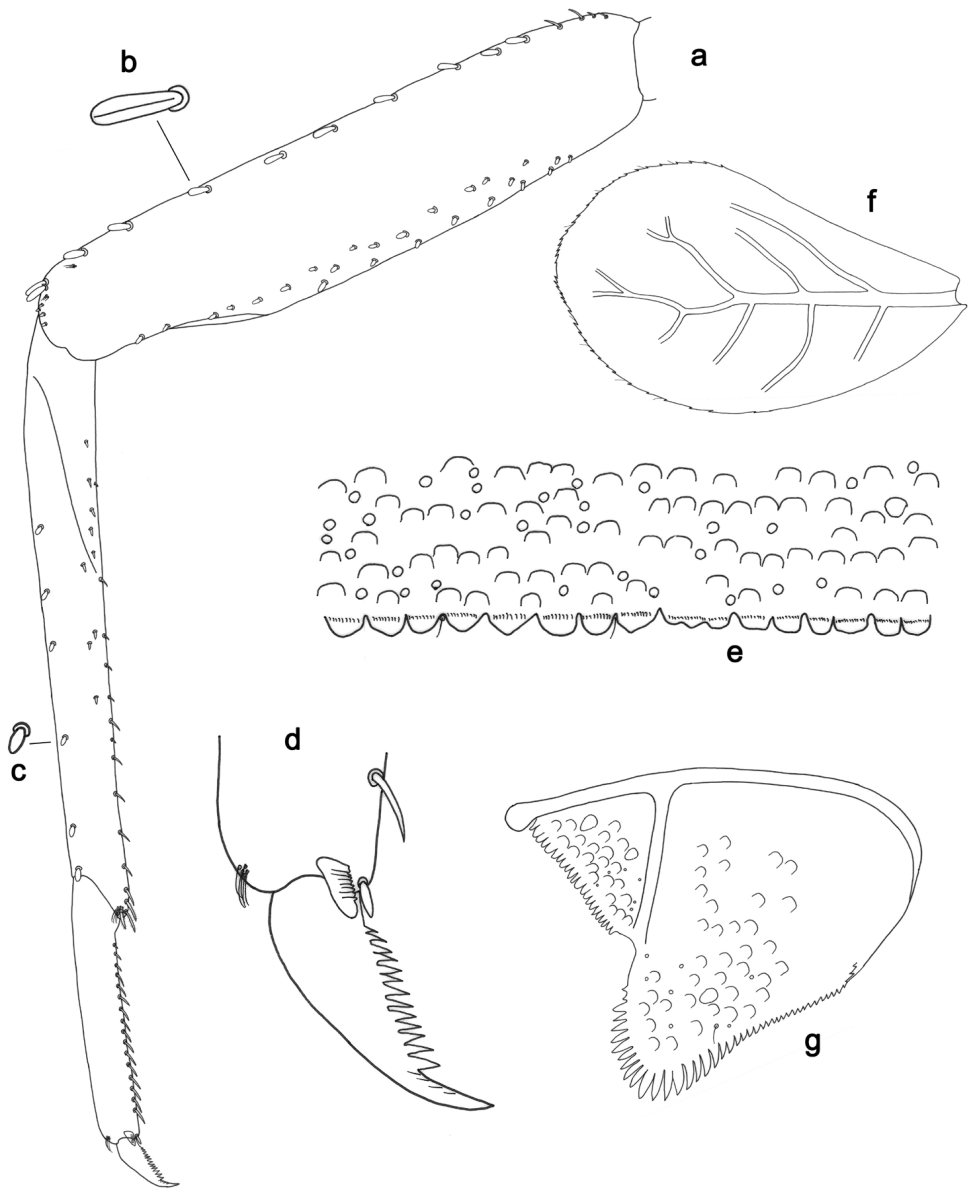
*Labiobaetispapuaensis* sp. n., larva morphology: **a**Foreleg**b** Femur dorsal seta **c** Tibia dorsal seta **d** Fore claw **e**Tergum IV **f** Gill IV **g**Paraproct.

Figures 14 , 15 , 59b , 64a

###### Diagnosis.

**Larva.** Following combination of characters: A) labrum dorsal submarginal arc of setae composed of 14–17 feathered setae, B) labial palp segment II with a large, lobed distomedial protuberance; C) labial palp segment III slightly pentagonal, apically slightly pointed; D) fore femur very slender, length ca. 5× maximum width, dorsal margin with a row of ca. 13 robust, slightly spatulate, apically blunt setae; E) tibia dorsal margin with a row of stout, lanceolate, apically rounded setae.

###### Description.

**Larva** (Figs 14, 15, 59b). Body length 5.4 mm.

*Colouration.* Head, thorax and abdomen dorsally brown, with bright pattern as in Fig. 59b. Head and thorax with bright median, dorsal suture, forewing pads with bright striation. Head, thorax and abdomen ventrally light brown, legs and caudal filaments light brown, femur dorsal margin brown.

*Antenna* with scape and pedicel sub-cylindrical, without distolateral process at scape; flagellum with lanceolate spines on apex of each segment.

*Labrum* (Fig. 14a, b). Rectangular, length 0.7× maximum width. Distal margin with medial emargination and a small process. Dorsally with medium, fine, simple setae scattered over surface; submarginal arc of setae composed of 14–17 long, feathered setae. Ventrally with marginal row of setae composed of lateral and anterolateral long, feathered setae and medial long, bifid, pectinate setae; ventral surface with eight short, spine-like setae near lateral and anterolateral margin.

*Right mandible* (Fig. 14c, d). Incisors fused. Outer and inner sets of denticles with 4 + 3 denticles. Inner margin of innermost denticle with a row of thin setae. Prostheca robust, apically denticulate. Margin between prostheca and mola slightly convex. Tuft of setae at apex of mola present.

*Left mandible* (Fig. 14e, f). Incisors fused. Outer and inner sets of denticles with 4 + 4 denticles. Prostheca robust, apically denticulate. Margin between prostheca and mola straight, with minute denticles towards subtriangular process. Subtriangular process long and slender, above level of area between prostheca and mola. Denticles of mola apically constricted. Tuft of setae at apex of mola present.

Both mandibles with lateral margins almost straight. Basal half with fine, simple setae scattered over dorsal surface.

*Hypopharynx*. Unknown.

*Maxilla* (Fig. 14g). Galea-lacinia with two simple, robust apical setae under crown. Inner dorsal row of setae with three denti-setae, distal denti-seta tooth-like, middle and proximal denti-setae slender, bifid and pectinate. Medially with one bipectinate, spine-like seta and four long, simple setae. Maxillary palp about as long as length of galea-lacinia; two segmented. Palp segment II 1.8× length of segment I. Setae on maxillary palp fine and simple, scattered over surface of segment II. Apex of last segment rounded, with an excavation at inner distolateral margin.

*Labium* (Fig. 14h). Glossa basally broad, narrowing toward apex; shorter than paraglossa; inner margin with nine spine-like setae increasing in length distally; apex with three long, robust, pectinate setae; outer margin with seven spine-like setae increasing in length distally; ventral surface with short, fine, simple setae. Paraglossa sub-rectangular, curved inward; apex rounded; with three rows of long, robust, apically pectinate setae; dorsally with row of six medium, simple setae; ventrally with three long, spine-like setae near inner margin. Labial palp with segment I 0.7× length of segments II and III combined. Segment I covered with micropores dorsally and ventrally with fine, simple setae along margins. Segment II with a large, lobed distomedial protuberance; distomedial protuberance 0.6× width of base of segment III; inner margin with few fine, simple setae; outer margin with few short, fine, simple setae; dorsally with a row of three long, spine-like setae. Segment III slightly pentagonal; apex slightly pointed; length 1.2× width; ventrally covered with medium spine-like, simple setae and short, fine, simple setae.

*Hind wing pads* absent.

*Foreleg* (Fig. 15a–d). Ratio of foreleg segments 1.1:1.0:0.4:0.1. *Femur*. Length ca. 5× maximum width. Dorsal margin with a row of ca. nine robust, slightly spatulate, apically rounded setae and four spine-like, apically pointed setae on basal area length of setae 0.17× maximum width of femur. Apex rounded; with one pair of robust, slightly spatulate, apically rounded setae and some short, stout, apically rounded setae. Many stout, broad lanceolate, apically rounded setae along ventral margin; femoral patch absent. *Tibia.* Dorsal margin with a row of stout, broad lanceolate, apically rounded setae. Ventral margin with a row of curved, spine-like setae and some longer, spine-like, bipectinate setae and a tuft of long, fine, simple setae on apex. Anterior surface scattered with stout, lanceolate setae. Tibio-patellar suture present on basal 1/3. *Tarsus.* Dorsal margin bare with a tuft of long, fine, simple setae on apex (Fig. 15d). Ventral margin with a row of curved, spine-like setae. Tarsal claw with one row of 12–13 denticles; distally pointed; with four stripes; subapical setae absent.

*Tergum* (Fig. 15e). Surface with irregular rows of U-shaped scale bases and scattered micropores. Posterior margin of tergum IV with rounded or triangular spines, wider than long.

*Gills* (Fig. 15f). Present on segments II–VII. Margin with small denticles and long, fine, simple setae. Tracheae extending from main trunk to inner and outer margins. Gill IV as long as length of segments V and VI combined. Gill VII as long as length of segments VIII, IX and 1/2 X combined.

*Paraproct* (Fig. 15g). Distally expanded, with many marginal, stout spines. Surface with U-shaped scale bases and scattered fine, simple setae and micropores. Postero-lateral extension (cercotractor) with small marginal spines.

###### Etymology.

Refers to the type locality in the Papua Province of Indonesia.

###### Distribution.

New Guinea.

###### Biological aspects.

The specimens were collected at an altitude of 774 m a.s.l.

###### Type-material.

**Holotype.** Nymph (on slide, GBIFCH 00465170), Indonesia, Papua, Road Nabire-Enarotali KM 55, 774 m, 22 Oct 2011, 03°29.80'S, 135°43.89'E, Balke (PAP09). Temporary deposited in MZL before definitely housed in MZB. **Paratype.** 1 nymph (on slide, GBIFCH 00465171, deposited in MZL), same data as holotype.

#### *L.petersorum* group of species

The group is characterised by mandibles with an outermost blade-like enlarged denticle, a short and rounded distomedial protuberance of labial palp segment II and a dorsal, submarginal arc of setae of the labrum composed of simple setae.

##### 8. Labiobaetis petersorum

Taxon classificationAnimaliaEphemeropteraBaetidae

(Lugo-Ortiz & McCafferty, 1999)

Figures 16 , 64b

###### Diagnosis.

**Figure 16.**
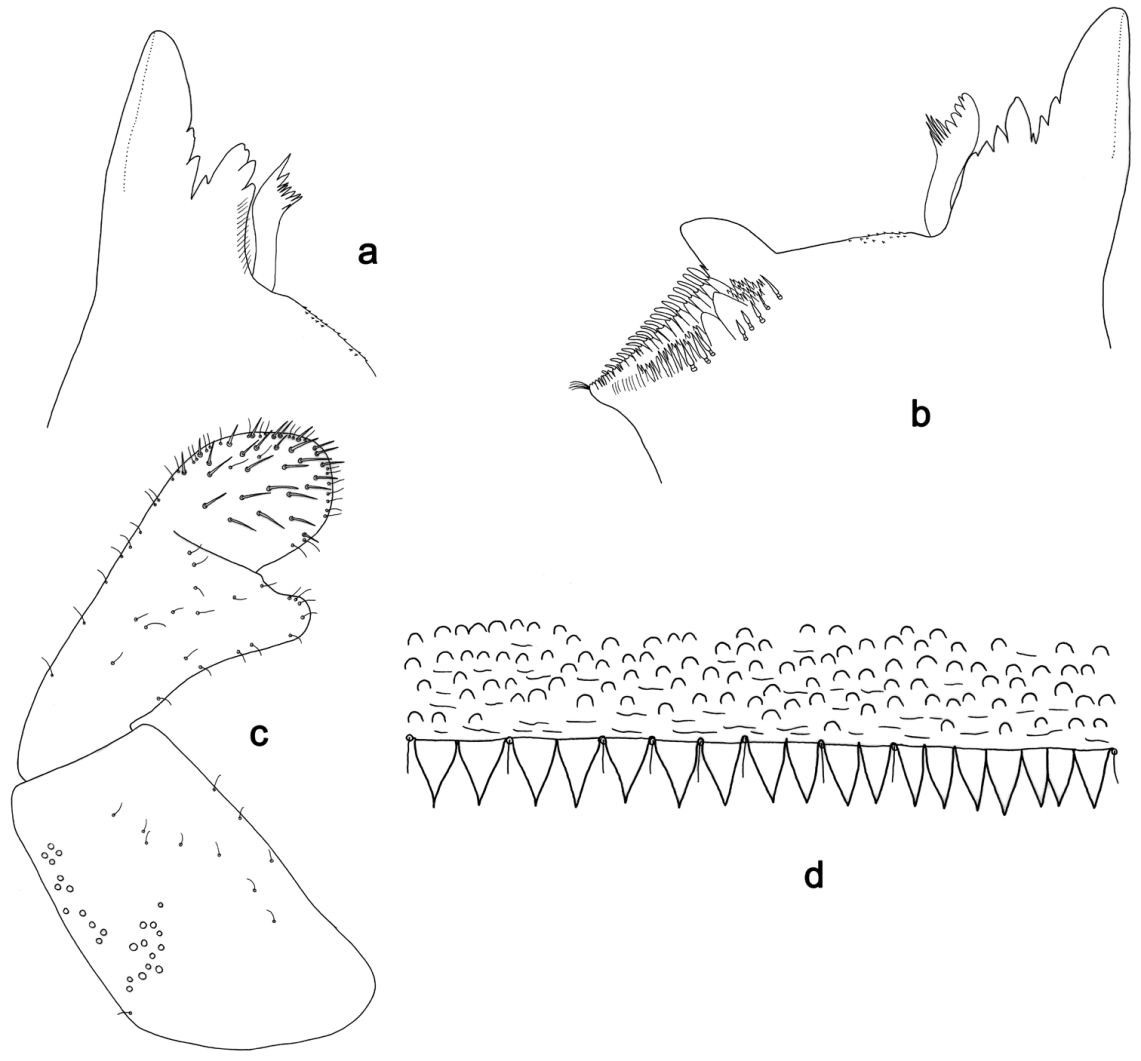
*Labiobaetispetersorum*, larva morphology: **a** Right mandible **b** Left mandible **c** Labial palp **d**Tergum IV.

**Larva.** Following combination of characters: A) labrum dorsal arc of setae composed of one plus 6–7 long, simple setae; B) both mandibles with outermost denticle blade-like; C) labial palp as Fig. 16c, segment II with short thumb-like distomedial protuberance; D) fore femur broad, length 2.6× maximum width, dorsal margin with a row of >40 curved, spine-like setae; E) spines at posterior margin of tergum IV triangular, pointed, longer than wide; F) paraproct distally not expanded.

###### Examined material.

**Paratypes.** 4 nymphs (on slides, PERC 0 012 564, PERC 0 012 565, PERC 0 012 566, PERC 0 012 567), Papua New Guinea, Bulolo R, 2950 ft, E of Wau, 15 Oct 1964, W.L. and J.G. Peters.

##### 9. Labiobaetis gladius sp. n.

Taxon classificationAnimaliaEphemeropteraBaetidae

http://zoobank.org/DE125588-E5E3-4DED-834B-848EABE2EDC5

**Figure 17.**
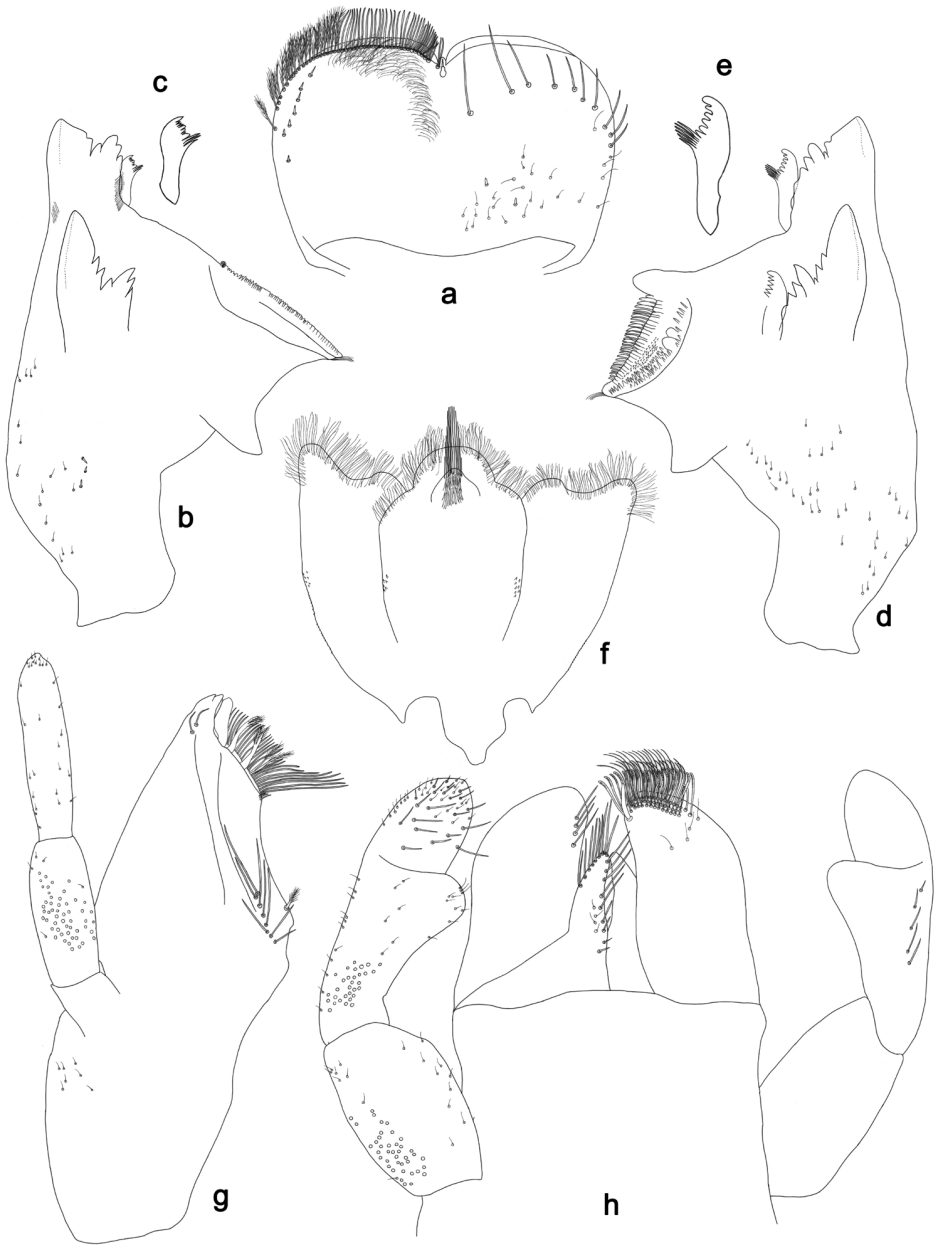
*Labiobaetisgladius* sp. n., larva morphology: **a** Labrum **b** Right mandible **c** Right prostheca **d** Left mandible **e** Left prostheca **f**Hypopharynx**g** Maxilla **h** Labium.

**Figure 18.**
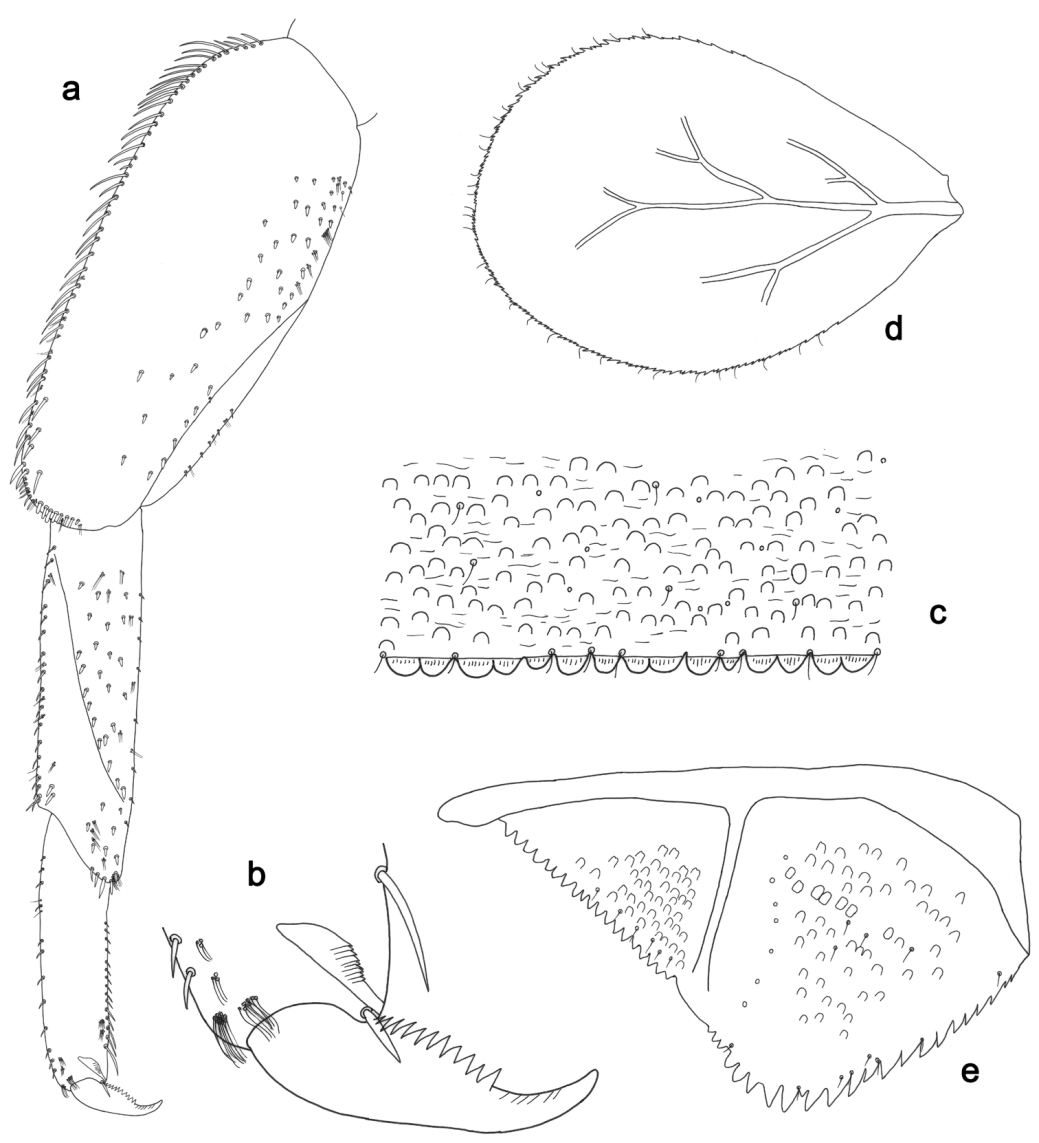
*Labiobaetisgladius* sp. n., larva morphology: **a**Foreleg**b** Fore claw **c**Tergum IV **d** Gill IV **e**Paraproct.

Figures 17 , 18 , 59c , 64b

###### Diagnosis.

**Larva.** Following combination of characters: A) labrum dorsal arc of setae composed of one plus 9–11 long, simple setae; B) both mandibles with outermost denticles blade-like; C) hypopharynx with concave distal margin of superlingua; D) maxillary palp somewhat longer than length of galea-lacinia; E) fore femur broad, length 2.5× maximum width, dorsal margin with a row of ca. 37 curved, spine-like setae and distally with some stout, pointed setae near margin; F) gills margin serrate with alternating smaller and bigger denticles intercalating long, fine, simple setae; G) spines at posterior margin of tergum IV rounded, wider than long; H) paraproct distally not expanded.

###### Description.

**Larva** (Figs 17, 18, 59c). Body length 6.7 mm; cerci: 3.7 mm; terminal filament: 2.8 mm.

*Colouration.* Head, thorax and abdomen dorsally brown, head and thorax with bright median, dorsal suture, forewing pads with slightly darker striation. Head, thorax and abdomen ventrally light brown, legs light brown and with brown marks as in Fig. 59c, caudal filaments light brown.

*Antenna* with scape and pedicel sub-cylindrical, without distolateral process at scape; flagellum with broad, apically blunt spines and fine, simple setae on apex of each segment.

*Labrum* (Fig. 17a). Rectangular, length 0.7× maximum width. Distal margin with medial emargination and a small process. Dorsally with short to medium, fine simple setae and some short, robust, simple setae on posterior area; submarginal arc of setae composed of one plus 9–11 long, simple setae. Ventrally with marginal row of setae composed of lateral and anterolateral long, feathered setae and medial long, pectinate setae, centrally bifid; ventral surface with seven short, spine-like setae near lateral and anterolateral margin.

*Right mandible* (Fig. 17b, c). Incisors fused. Outer and inner sets of denticles with 3 + 4 denticles, outermost denticle blade-like. Inner margin of innermost denticle with a row of thin setae. Prostheca robust, apically with small denticles and comb-shape structure. Margin between prostheca and mola straight. Tuft of setae at apex of mola present.

*Left mandible* (Fig. 17d, e). Incisors fused. Outer and inner sets of denticles with 3 + 4 denticles plus one minute intermediate denticle, outermost denticle blade-like. Prostheca robust, apically with small denticles and comb-shape structure. Margin between prostheca and mola straight. Subtriangular process long and slender, above level of area between prostheca and mola. Denticles of mola apically constricted. Tuft of setae at apex of mola present.

Both mandibles with lateral margins almost straight. Basal half with fine, simple setae scattered over dorsal surface.

*Hypopharynx* (Fig. 17f). Lingua longer than superlingua. Lingua longer than width; medial tuft of stout setae present; distal half laterally expanded. Superlingua concave; lateral margin straight; fine, long, simple setae along distal margin.

*Maxilla* (Fig. 17g). Galea-lacinia with two simple, robust apical setae under crown. Inner dorsal row of setae with three denti-setae, distal denti-seta tooth-like, middle and proximal denti-setae slender, bifid and pectinate. Medially with one bipectinate, spine-like seta and eight long, simple setae. Maxillary palp slightly longer than length of galea-lacinia; two segmented. Palp segment II 1.3× length of segment I. Setae on maxillary palp fine and simple, scattered over surface of segments I and II. Apex of last segment slightly pointed, without excavation at inner distolateral margin.

*Labium* (Fig. 17h). Glossa basally broad, narrowing toward apex; shorter than paraglossa; inner margin with nine spine-like setae increasing in length distally; apex with three long, robust setae; outer margin with six long spine-like setae increasing in length distally; ventral surface with few short, fine, simple setae. Paraglossa sub-rectangular, curved inward; apex rounded; with three rows of long, robust, apically pectinate setae; dorsally with row of four medium, simple setae; ventrally with four long, spine-like setae near inner margin. Labial palp with segment I 0.7× length of segments II and III combined. Segment I covered with micropores dorsally and ventrally with fine, simple setae along margins. Segment II with a short, thumb-like distomedial protuberance; distomedial protuberance 0.4× width of base of segment III; inner and outer margin both with fine, simple setae; dorsally with row of 5–6 spine-like, simple setae, decreasing in length distally. Segment III oblong; apex slightly pointed; length 1.3× width; ventrally covered with long and medium spine-like, simple setae and short, fine, simple setae.

*Hind wing pads* absent.

*Foreleg* (Fig. 18a, b). Ratio of foreleg segments 1.6:1.0:0.7:0.3. *Femur*. Length 2.5× maximum width. Dorsal margin with a row of ca. 37 curved, spine-like setae and distally with some stout, pointed setae near margin; length of setae 0.18× maximum width of femur. Apex rounded; with one pair of curved, spine-like setae and many short, stout, apically rounded setae. Many stout, lanceolate setae and a few fine, simple setae along ventral margin; femoral patch poorly developed. *Tibia.* Dorsal margin with a row of short, curved, spine-like setae and long, fine, simple setae, apically with two longer, curved, spine-like setae. Ventral margin with a row of short, spine-like setae and one stout, spine-like seta and a tuft of long, fine, simple setae on apex. Anterior surface scattered with many stout, lanceolate setae and fine, simple setae. Tibio-patellar suture present on basal 3/4. *Tarsus.* Dorsal margin with a row of short, spine-like setae and some long, fine, simple setae. Ventral margin with a row of curved, spine-like setae on margin. Tarsal claw with one row of eleven denticles; tapering distally; with six stripes; subapical setae absent.

*Tergum* (Fig. 18c). Surface with irregular rows of U-shaped scale bases and scattered fine, simple setae and micropores, scales oblong. Posterior margin of tergum IV with rounded spines, wider than long.

*Gills* (Fig. 18d). Present on segments II–VII. Margin with alternating smaller and bigger denticles intercalating long, fine, simple setae. Tracheae extending from main trunk to inner and outer margins, pigmentation limited to main trunk and extensions to inner margin. Gill IV as long as length of segments V and 1/2 VI combined. Gill VII as long as length of segments VIII and 1/3 IX combined.

*Paraproct* (Fig. 18e). Distally not expanded, with ca. 22 marginal, stout spines. Surface with U-shaped scale bases and scattered fine, simple setae and micropores. Postero-lateral extension (cercotractor) with small marginal spines.

###### Etymology.

Latin word for sword, refers to the blade-like outermost denticle of the mandibles.

###### Distribution.

New Guinea.

###### Biological aspects.

The specimens were collected at altitudes of 1800–3210 m a.s.l.

###### Type-material.

**Holotype.** Nymph (on slide, GBIFCH 00465172), Papua New Guinea, Simbu Prov., 05°49.96'S, 145°06.129'E, (GPS), Mt. Wilhelm, Pindaunde Creek, 2350 m a.s.l. (7915 ft), S5 (oria.6), 18 Aug 1999, leg. L. Čížek . Deposited in MZL. **Paratypes.** 7 nymphs (3 on slides, GBIFCH 00465173, GBIFCH 00465177, GBIFCH 00465178, 4 in alcohol, GBIFCH 00515241, deposited in MZL), same data as holotype; 44 nymphs (2 on slides, GBIFCH 00465174, GBIFCH 00465175, 27 in alcohol, GBIFCH 00515242, GBIFCH 00508132, deposited in MZL; 15 in alcohol, GBIFCH 00515243, deposited in ZSM), Papua New Guinea, Simbu Prov., 05°48.050'S, 145°04.150'E, (GPS), Mt. Wilhelm, Pindaunde Creek, 3210 m a.s.l., (10895 ft), S2 (oria.3), 17 Aug 1999, L. Čížek leg; 52 nymphs (1 on slide, GBIFCH 00465176, 32 in alcohol, GBIFCH 00515239, GBIFCH 00508124, GBIFCH 00508133, deposited in MZL; 20 in alcohol, GBIFCH 00515240, deposited in ZSM), Papua New Guinea, Simbu Prov., 05°49.033'S, 145°5.271'E, (GPS), Mt. Wilhelm, Pindaunde Creek, 2600 m a.s.l. (near fish farm), (9181 ft GPS), S4 (oria.5), 18 Aug 1999, L. Čížek leg.

###### Additional material.

5 nymphs (1 on slide, GBIFCH 00465179, 4 in alcohol, GBIFCH 00515275, GBIFCH 00508135, deposited in MZL), Papua New Guinea, Western Highlands, Simbai, 1800–2000 m, 26 Feb 2007, 05°15.87'S, 144°32.72'E, Kinibel (PNG 134); 2 nymphs (1 on slide, GBIFCH 00465180, 1 in alcohol, GBIFCH 00515261, deposited in MZL), Papua New Guinea, Eastern Highlands, Akameku-Brahmin, Bismarck Range, 2200 m, 23 Nov 2006, 05°56.80'S, 145°22.24'E, Balke & Kinibel (PNG 106).

##### 10. Labiobaetis janae sp. n.

Taxon classificationAnimaliaEphemeropteraBaetidae

http://zoobank.org/358E21B7-E2BD-446F-BA28-2F1DFEFCA3A4

**Figure 19.**
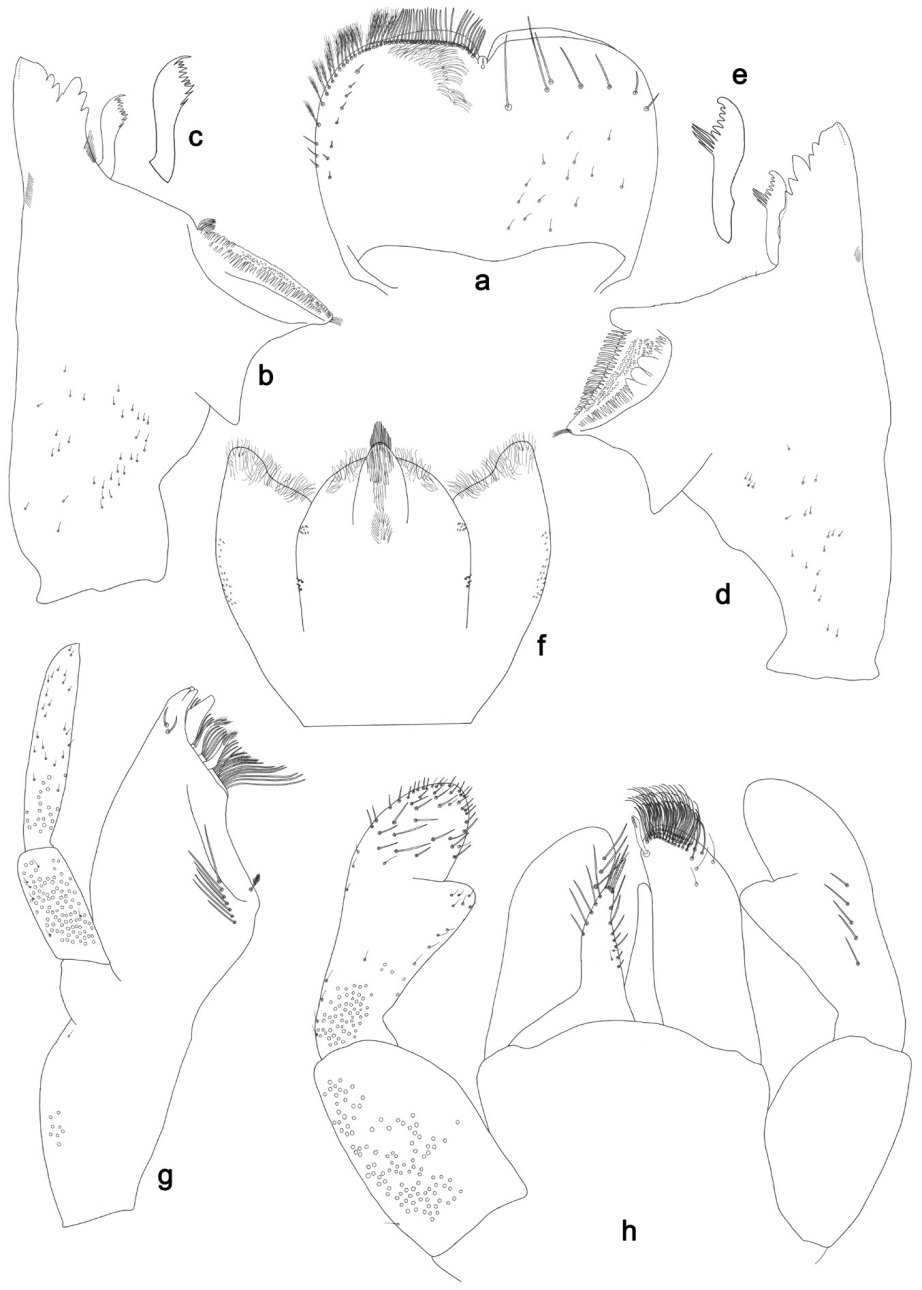
*Labiobaetisjanae* sp. n., larva morphology: **a** Labrum **b** Right mandible **c** Right prostheca **d** Left mandible **e** Left prostheca **f**Hypopharynx**g** Maxilla **h** Labium.

**Figure 20.**
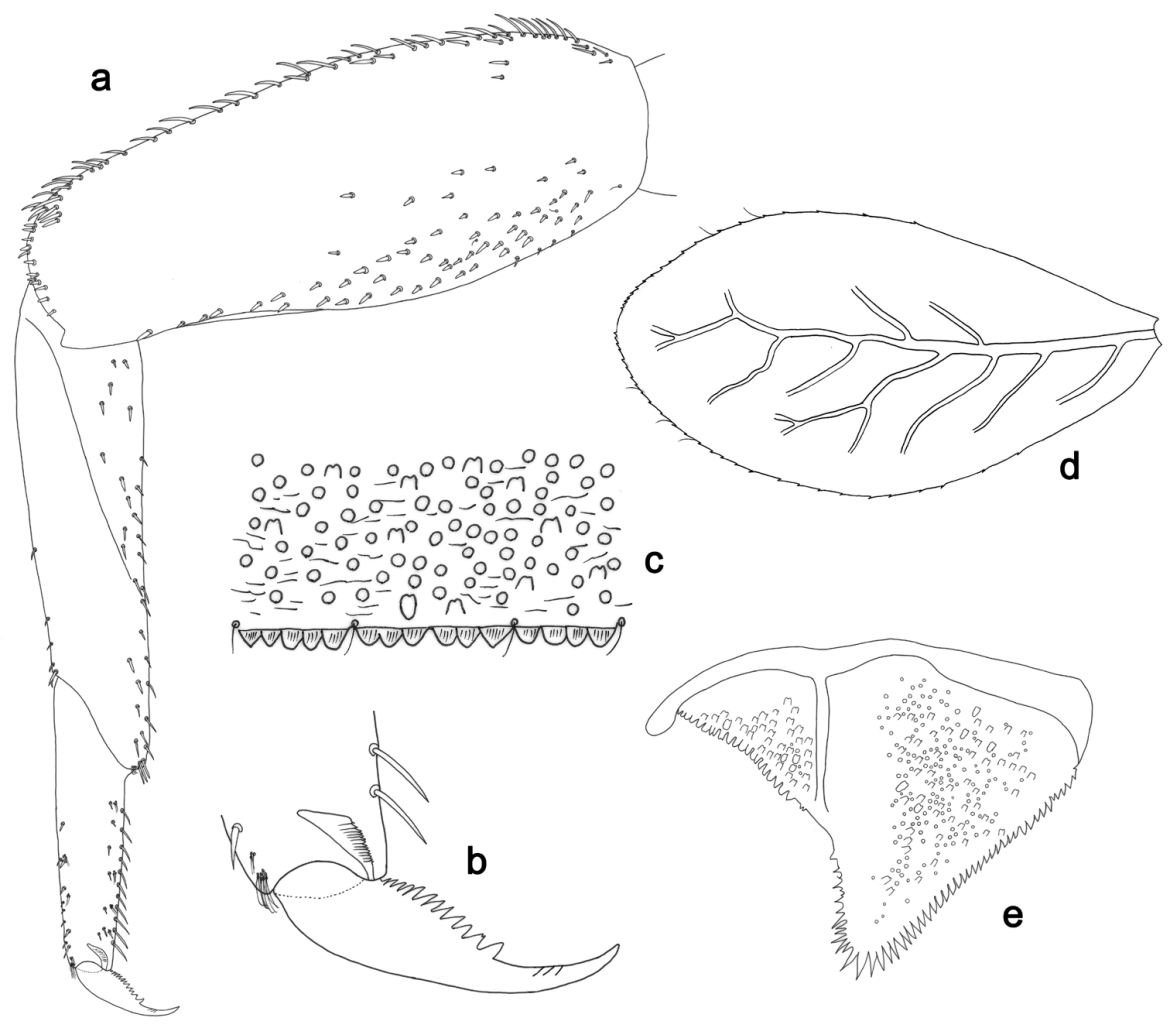
*Labiobaetisjanae* sp. n., larva morphology: **a**Foreleg**b** Fore claw **c**Tergum IV **d** Gill IV **e**Paraproct.

Figures 19 , 20 , 59d , 64b

###### Diagnosis.

**Larva.** Following combination of characters: A) labrum dorsal arc of setae with one plus 6–7 long, simple setae; B) both mandibles with outermost denticle blade-like; C) hypopharynx with slightly concave distal margin of superlingua; D) maxillary palp somewhat longer than length of galea-lacinia; E) fore femur rather broad, length 2.6× maximum width, dorsal margin with a row of ca. 34 curved, spine-like setae and with some stout, pointed setae near margin; F) gills long, gill IV as long as length of segments V to VII combined, gill VII somewhat longer than length of segments VIII to X combined.

###### Description.

**Larva** (Figs 19, 20, 59d). Body length 8–9 mm.

*Colouration.* Head, thorax and abdomen dorsally grey-brown, abdominal segments VI, IX and X brighter, head and thorax with bright median, dorsal suture, forewing pads with bright striation. Head, thorax and abdomen ventrally light grey-brown, legs colourless with brown medial spot and dorsal margin on femur, caudal filaments light brown.

*Antenna* with scape and pedicel sub-cylindrical, without distolateral process at scape; flagellum with lanceolate spines on apex of each segment.

*Labrum* (Fig. 19a). Rectangular, length 0.7× maximum width. Distal margin with medial emargination and small process. Dorsally with medium, fine, simple setae scattered over surface; submarginal arc of setae composed of one plus 6–7 long, simple setae. Ventrally with marginal row of setae composed of lateral and anterolateral long, feathered setae and medial long, pectinate setae, centrally bifid; ventral surface with eight short, spine-like setae near lateral and anterolateral margin.

*Right mandible* (Fig. 19b, c). Incisors fused. Outer and inner sets of denticles with 3 + 3 denticles plus one minute intermediate denticle, outermost denticle blade-like. Inner margin of innermost denticle with a row of thin setae. Prostheca robust, apically and distolaterally denticulate. Margin between prostheca and mola straight. Tuft of setae at apex of mola present.

*Left mandible* (Fig. 19d, e). Incisors fused. Outer and inner sets of denticles with 3 + 4 denticles, outermost denticle blade-like. Prostheca robust, apically with small denticles and comb-shape structure. Margin between prostheca and mola straight, with few minute setae, and with minute denticles toward subtriangular process. Subtriangular process long and slender, above level of area between prostheca and mola. Denticles of mola apically constricted. Tuft of setae at apex of mola present.

Both mandibles with lateral margins almost straight. Basal half with fine, simple setae scattered over dorsal surface.

*Hypopharynx* (Fig. 19f). Lingua about as long as superlingua. Lingua about as broad as long; medial tuft of stout setae present; distal half not expanded. Superlingua slightly concave; lateral margin rounded; fine, long, simple setae along distal margin.

*Maxilla* (Fig. 19g). Galea-lacinia with two simple, robust apical setae under crown. Inner dorsal row of setae with three denti-setae, distal denti-seta tooth-like, middle and proximal denti-setae slender, bifid and pectinate. Medially with one bipectinate, spine-like seta and seven long, simple setae. Maxillary palp slightly longer than length of galea-lacinia; two segmented. Palp segment II 2.2× length of segment I. Setae on maxillary palp fine and simple, scattered over surface of segments I and II. Apex of last segment slightly pointed, without excavation at inner distolateral margin.

*Labium* (Fig. 19h). Glossa basally broad, narrowing toward apex; shorter than paraglossa; inner margin with 7–8 spine-like setae increasing in length distally; apex with three long, robust, pectinate setae; outer margin with five long, spine-like setae; ventral surface with few short, fine, simple setae. Paraglossa sub-rectangular, curved inward; apex rounded; with three rows of long, robust, apically pectinate setae; dorsally with three medium, simple setae; ventrally with three long, spine-like setae near inner margin. Labial palp with segment I 0.8× length of segments II and III combined. Segment I covered with micropores dorsally. Segment II with a short, thumb-like distomedial protuberance; distomedial protuberance 0.4× width of base of segment III; inner and outer margin both with short, fine, simple setae; dorsally with row of five long, spine-like, simple setae. Segment III oblong; apex slightly pointed; length 1.1× width; ventrally covered with long and medium spine-like, simple setae and short, fine, simple setae.

*Hind wing pads* absent.

*Foreleg* (Fig. 20a, b). Ratio of foreleg segments 1.5:1.0:0.6:0.3. *Femur*. Length ca. 3× maximum width. Dorsal margin with a row of ca. 34 curved, spine-like setae and some stout, pointed setae near margin; length of setae 0.15× maximum width of femur. Apex rounded; with two pairs of curved, spine-like setae and many short, stout, pointed setae. Many stout, lanceolate setae and a few fine, simple setae along ventral margin; femoral patch absent. *Tibia.* Dorsal margin with few short, curved, spine-like setae and a pair of longer, curved, spine-like setae on apex. Ventral margin with a row of curved, spine-like setae and some longer, stout, pointed setae on apex. Anterior surface scattered with many stout, lanceolate setae. Tibio-patellar suture present on basal 2/3. *Tarsus.* Dorsal margin with some short, spine-like setae and fine simple setae. Ventral margin with a row of curved, spine-like setae. Tarsal claw with one row of 12 denticles; distally pointed; with three stripes; subapical setae absent.

*Tergum* (Fig. 20c). Surface with many micropores and scattered W-shaped scale bases, scales oblong. Posterior margin of tergum IV with rounded or triangular spines, wider than long.

*Gills* (Fig. 20d). Present on segments II - VII. Margin with small denticles and long, fine, simple setae. Tracheae extending from main trunk to inner margin and partly to outer margin. Gill IV as long as length of segments V, VI and VII combined. Gill VII somewhat longer than length of segments VIII and X combined.

*Paraproct* (Fig. 20e). Distally expanded, with many marginal, stout spines. Surface with W-shaped scale bases and many micropores. Postero-lateral extension (cercotractor) with small marginal spines.

###### Etymology.

Dedicated to Janice (“Jan”) Peters (Florida A & M University), who pioneered the collection of mayflies in New Guinea.

###### Distribution.

New Guinea.

###### Biological aspects.

The specimens were collected at an altitude of 3200 m a.s.l.

###### Type-material.

**Holotype.** Nymph (on slide, GBIFCH 00465181), Indonesia, Papua, Lake Habemma, stream, 3200 m, 19 Oct 2011, 04°07.77'S, 138°40.77'E, Balke (PAP07). Temporary deposited in MZL before definitely housed in MZB. **Paratypes.** 30 nymphs (1 on slide, GBIFCH 00465182, 19 in alcohol, GBIFCH 00515252, GBIFCH 00508126, deposited in MZL; 11 in alcohol, GBIFCH 00515253, deposited in ZSM), same data as holotype.

#### *L.tuberpalpus* group of species

The group is characterised by a compact, rounded distomedial protuberance of labial palp segment II and a dorsal, submarginal arc of setae of the labrum composed of simple setae. In this arc of setae the first and second setae after the central, submedian seta are standing closely together.

##### 11. Labiobaetis tuberpalpus

Taxon classificationAnimaliaEphemeropteraBaetidae

(Lugo-Ortiz & McCafferty, 1999)

Figures 21 , 65a

###### Diagnosis.

**Figure 21.**
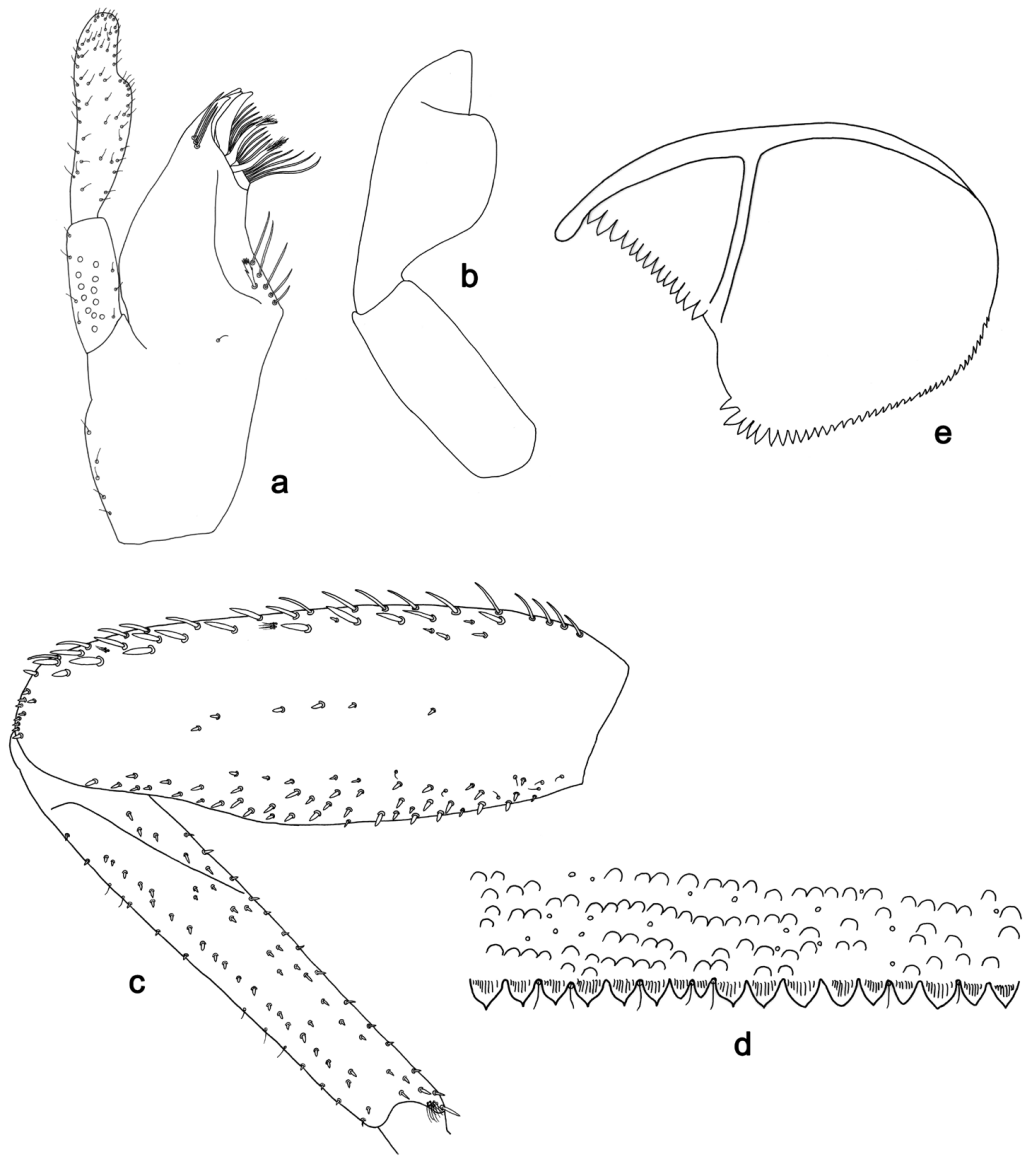
*Labiobaetistuberpalpus*, larva morphology: **a** Maxilla **b** Labial palp **c**Foreleg (femur, tibia) **d**Tergum IV.

**Larva.** Following combination of characters: A) labrum dorsal arc of setae composed of one plus 4–5 long, simple setae; B) maxillary palp longer than galwa-lacinia, with well-developed excavation at inner distolateral margin of segment II; C) fore femur rather broad, length 2.8× maximum width, foreleg setation as Fig. 21c; D) fore claw with a row of 9–10 denticles; E) spines at posterior margin of tergum IV mostly triangular and pointed, about as long as wide; F) paraproct with ca. 38 marginal spines.

###### Examined material.

**Paratypes.** 1 nymph (on slides, PERC 0 010 571), Papua New Guinea, Bulolo R, E of Wau, 2950 ft, 18 Oct 1964, W.L. and J.G. Peters; 1 nymph (on slide, PERC 0 010 573), Papua New Guinea, Morobe Prov., Clearwater Cr, nr Luau, 15 Sept 1983, J.T. and D.A. Polhemus.

##### 12. Labiobaetis branchiaesetis sp. n.

Taxon classificationAnimaliaEphemeropteraBaetidae

http://zoobank.org/E3BD391C-F1E3-461E-9DD9-C6F37EFA16B1

**Figure 22.**
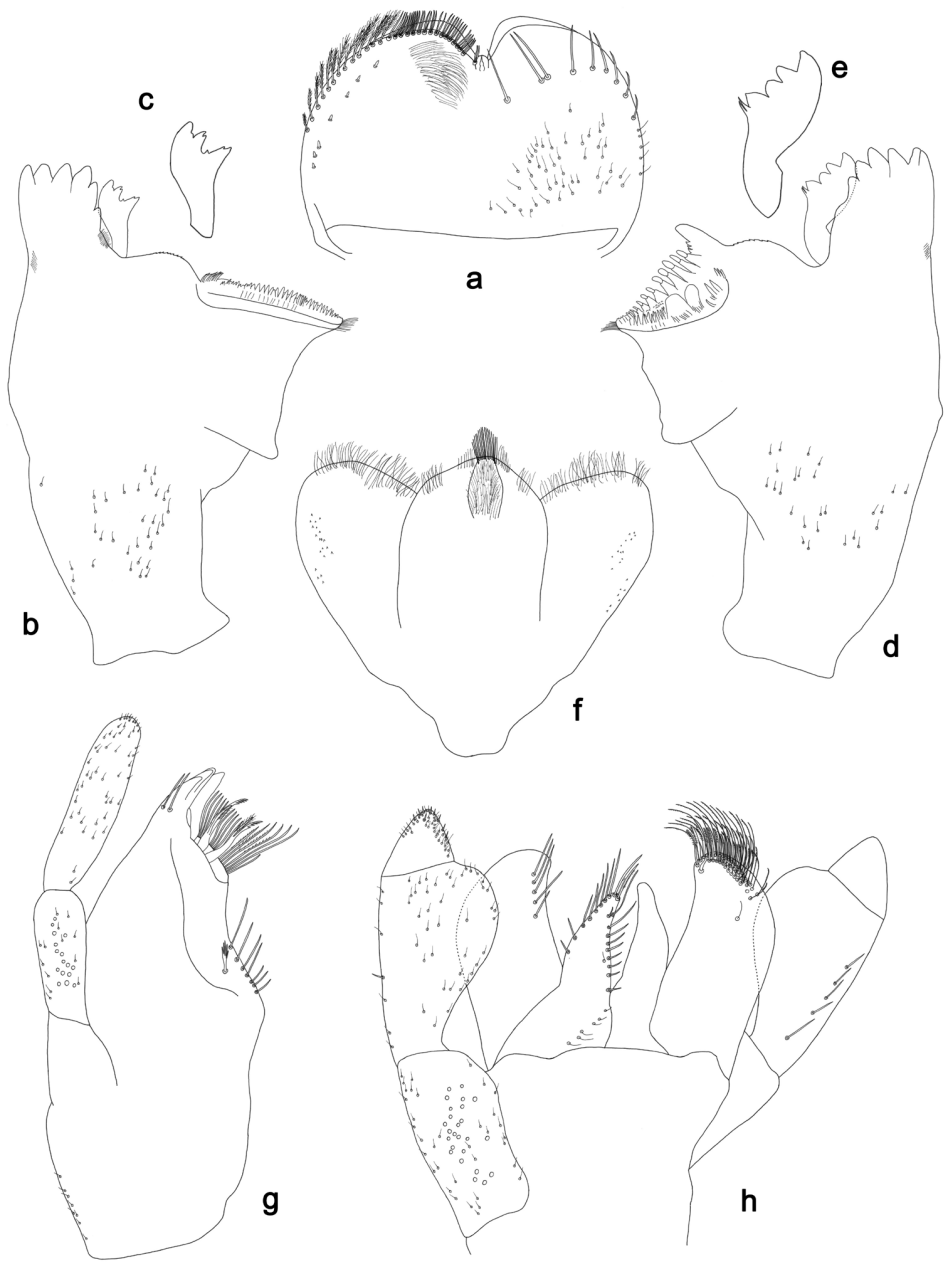
*Labiobaetisbranchiaesetis* sp. n., larva morphology: **a** Labrum **b** Right mandible **c** Right prostheca **d** Left mandible **e** Left prostheca **f**Hypopharynx**g** Maxilla **h** Labium.

**Figure 23.**
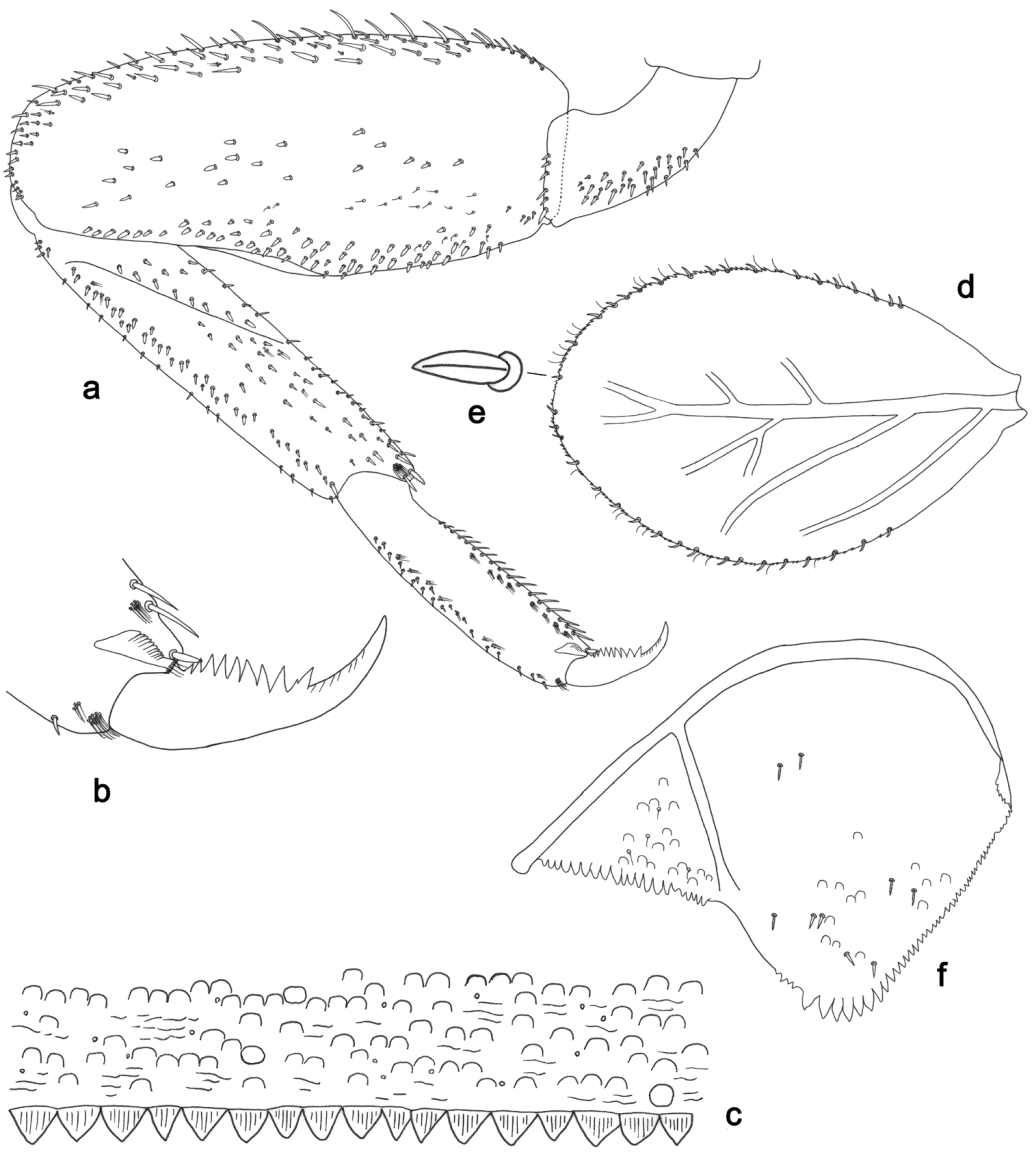
*Labiobaetisbranchiaesetis* sp. n., larva morphology: **a**Foreleg**b** Fore claw **c**Tergum IV **d** Gill IV **e** Seta on gill margin **f**Paraproct.

Figures 22 , 23 , 60a , 65a

###### Diagnosis.

**Larva.** Following combination of characters: A) labrum dorsal submarginal arc of setae composed of one plus eight or nine long, simple setae; B) labial palp segment II with a compact, rounded distomedial protuberance; C) fore femur broad, length ca. 2× maximum width, dorsal margin with a row of ca. 26 curved, spine-like setae and many stout, pointed setae near margin; D) gills margin serrate with small spines intercalating long, fine, simple setae, and with robust, lanceolate setae on margin; E) paraproct surface with scales or scale bases and fine, slightly lanceolate setae as well as fine, simple setae.

###### Description.

**Larva** (Figs 22, 23, 60a). Body length 7.5 mm; cerci: 7.5 mm; terminal filament: 3.2 mm; antenna: approximately twice as long as head length.

*Colouration.* Head, thorax and abdomen dorsally dark brown, abdominal segment X light brown, head and thorax with bright median, dorsal suture. Head, thorax and abdomen ventrally brown, femur dorsal and ventral margins brown, legs otherwise colourless, caudal filaments light brown.

*Antenna* with scape and pedicel sub-cylindrical, without distolateral process at scape; flagellum with lanceolate spines and fine, simple setae on apex of each segment.

*Labrum* (Fig. 22a). Rectangular, length 0.6× maximum width. Distal margin with medial emargination and a small process. Dorsally with many short to medium, fine, simple setae; submarginal arc of setae composed of one plus 8–9 long, simple setae. Ventrally with marginal row of setae composed of lateral and anterolateral long, feathered setae and medial long, bifid setae; ventral surface with seven short, spine-like setae near lateral and anterolateral margin.

*Right mandible* (Fig. 22b, c). Incisors fused. Outer and inner sets of denticles with 3 + 3 denticles. Inner margin of innermost denticle with a row of thin setae. Prostheca robust, apically denticulate. Margin between prostheca and mola slightly convex, with minute denticles. Tuft of setae at apex of mola present.

*Left mandible* (Fig. 22d, e). Incisors fused. Outer and inner sets of denticles with 3 + 4 denticles. Prostheca robust, apically denticulate. Margin between prostheca and mola slightly convex, with minute denticles toward subtriangular process. Subtriangular process long and slender, above level of area between prostheca and mola. Denticles of mola apically constricted. Tuft of setae at apex of mola present.

Both mandibles with lateral margins slightly convex. Basal half with fine, simple setae scattered over dorsal surface.

*Hypopharynx* (Fig. 22f). Lingua about as long as superlingua. Lingua longer than broad; medial tuft of stout setae present; distal half not expanded. Superlingua rounded; lateral margin rounded; fine, long, simple setae along distal margin.

*Maxilla* (Fig. 22g). Galea-lacinia with two simple, robust apical setae under crown. Inner dorsal row of setae with three denti-setae, distal denti-seta tooth-like, middle and proximal denti-setae slender, bifid and pectinate. Medially with one bipectinate, spine-like seta and seven long, simple setae. Maxillary palp 1.2× as long as length of galea-lacinia; two segmented. Palp segment II 1.5× length of segment I. Setae on maxillary palp fine and simple, scattered over surface of segments I and II. Apex of last segment rounded, with a slight excavation at inner distolateral margin.

*Labium* (Fig. 22h). Glossa basally broad, narrowing toward apex; shorter than paraglossa; inner margin with nine spine-like setae increasing in length distally; apex with two long and one medium, robust, pectinate setae; outer margin with eight long, spine-like setae increasing in length distally; ventral surface with few short, fine, simple setae. Paraglossa sub-rectangular, curved inward; apex rounded; with three rows of long, robust, apically pectinate setae; dorsally with one medium, simple seta; ventrally with five long, spine-like setae near inner margin. Labial palp with segment I 0.7× length of segments II and III combined. Segment I covered with short, fine, simple setae ventrally and micropores dorsally. Segment II with a compact, rounded distomedial protuberance; distomedial protuberance 0.6× width of base of segment III; inner and outer margin both with short, fine, simple setae; dorsally with a row of five long, spine-like, simple setae. Segment III conical; apex rounded; length 0.9× width; ventrally covered with short, fine, simple setae.

*Hind wing pads* absent.

*Foreleg* (Fig. 23a, b). Ratio of foreleg segments 1.3:1.0:0.7:0.2. *Femur*. Length ca. 2× maximum width. Dorsal margin with a row of ca. 26 curved, spine-like setae and with many stout, pointed setae near margin; length of setae 0.14× maximum width of femur. Apex rounded; with one curved, spine-like seta and many short, stout, pointed setae. Many stout, lanceolate setae and a few fine, simple setae along ventral margin; femoral patch poorly developed. *Tibia.* Dorsal margin with a row of short, spine-like setae and many stout, lanceolate setae along margin. Ventral margin with a row of curved, spine-like setae, on apex one bipectinate, spine-like seta and a tuft of long, fine, simple setae. Anterior surface scattered with many stout, lanceolate setae. Tibio-patellar suture present on basal 1/2. *Tarsus.* Dorsal margin with a row of short, spine-like setae and long, simple setae. Ventral margin with a row of curved, spine-like setae. Tarsal claw with one row of 9–10 denticles; distally pointed; with seven stripes; subapical setae absent.

*Tergum* (Fig. 23c). Surface with irregular rows of U-shaped scale bases and scattered micropores; scales short, apically rounded. Posterior margin of tergum IV with triangular spines, about as long as wide.

*Gills* (Fig. 23d, e). Present on segments II - VII. Margin with small denticles intercalating long, fine, simple setae, and with robust, lanceolate setae on margin. Tracheae extending from main trunk to inner margin and partly to outer margin. Gill IV as long as length of segments V and 1/2 VI combined. Gill VII as long as length of segments VIII and 1/3 IX combined.

*Paraproct* (Fig. 23f). Distally slightly expanded, with many marginal, stout spines. Surface with U-shaped scale bases and scattered fine, slightly lanceolate setae as well as fine, simple setae. Postero-lateral extension (cercotractor) with small marginal spines.

###### Etymology.

Latin words for gills and seta, refers to the robust, lanceolate setae on the margin of the gills.

###### Distribution.

New Guinea.

###### Biological aspects.

The specimens were collected in altitudes of 1000 m a.s.l. and 1700 m–1800 m a.s.l.

###### Type-material.

**Holotype.** Nymph (on slide, GBIFCH 00465183), Papua New Guinea, Eastern Highlands, Marawaka, Ande, 1700–1800 m, 09 Nov 2006, nr 07°01.70'S, 145°49.81'E, Balke & Kinibel (PNG 87). Deposited in ZSM. **Paratypes.** 24 nymphs (2 on slides, GBIFCH 00465184, GBIFCH 00465185, 14 in alcohol, GBIFCH 00515219, GBIFCH 00508123, deposited in MZL; 8 in alcohol, GBIFCH 00515220, deposited in ZSM), same data as holotype; 3 nymphs (1 on slide, GBIFCH 00465186, 2 in alcohol, GBIFCH 00515286, deposited in MZL), Papua New Guinea, Gulf, Marawaka, nr Ande, 1000 m, 10 Nov 2006, 07°03.60'S, 145°44.38'E, Balke & Kinibel (PNG 89).

##### 13. Labiobaetis magnovaldus sp. n.

Taxon classificationAnimaliaEphemeropteraBaetidae

http://zoobank.org/EE4A5136-ADD7-487F-AF3B-877B79D87BB1

**Figure 24.**
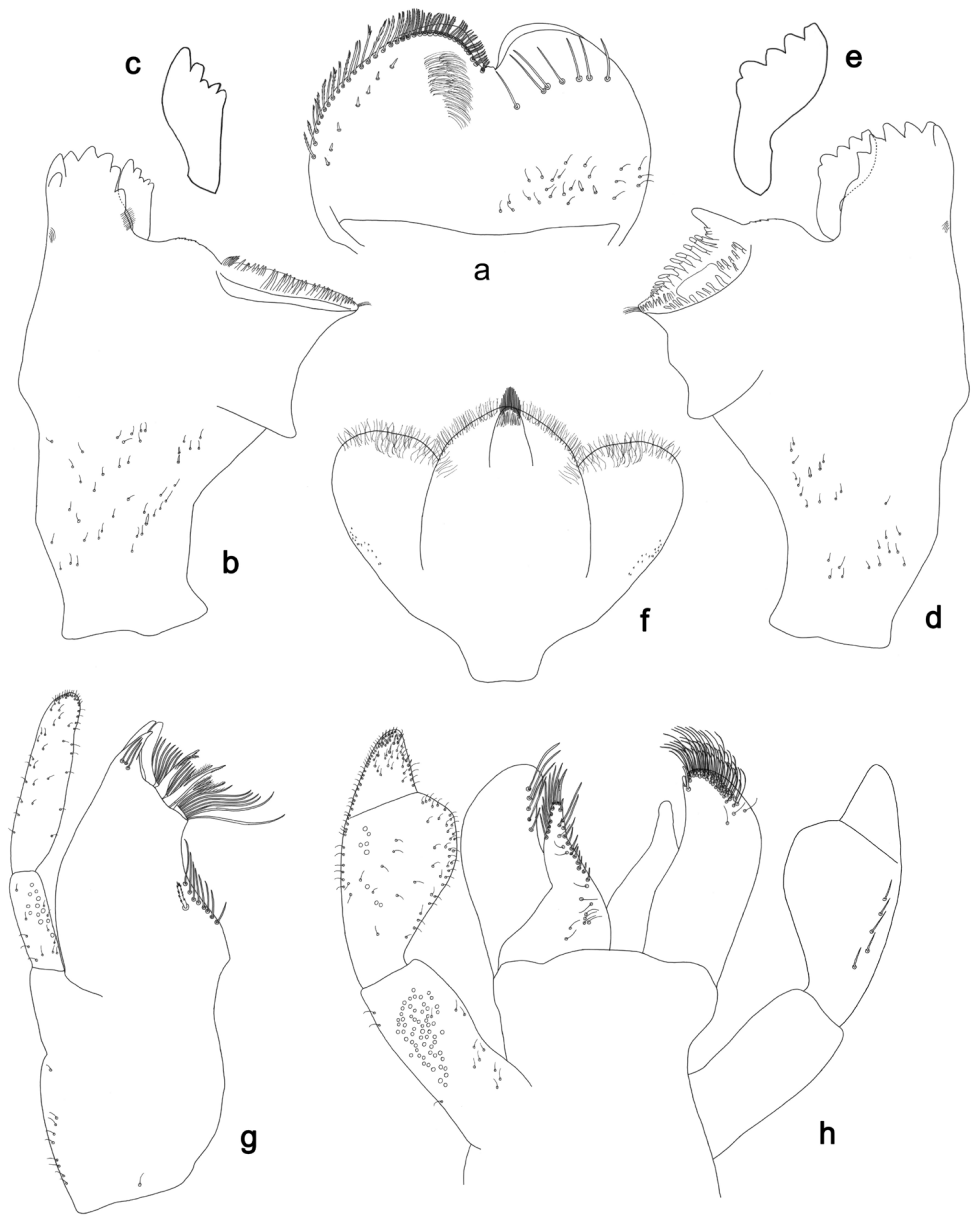
*Labiobaetismagnovaldus* sp. n., larva morphology: **a** Labrum **b** Right mandible **c** Right prostheca **d** Left mandible **e** Left prostheca **f**Hypopharynx**g** Maxilla **h** Labium.

**Figure 25.**
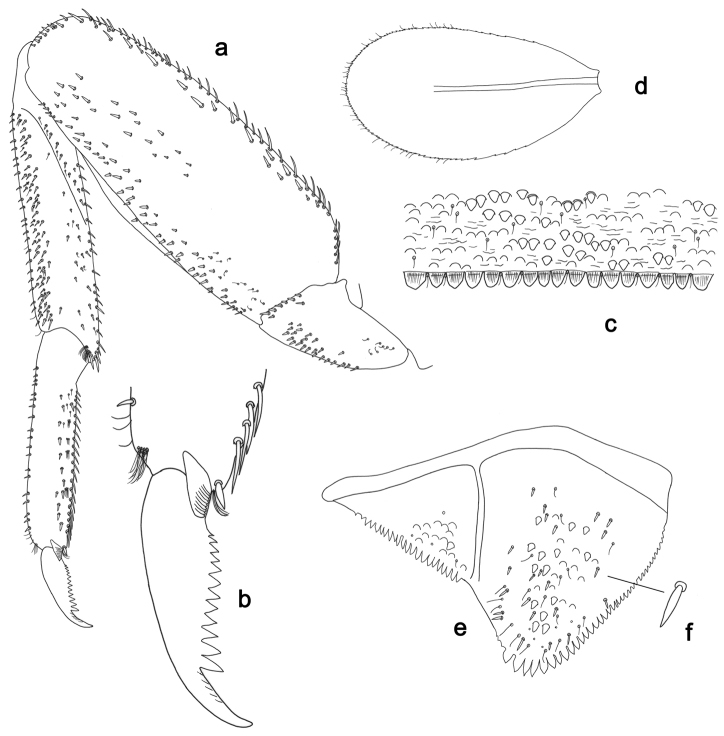
*Labiobaetismagnovaldus* sp. n., larva morphology: **a**Foreleg**b** Fore claw **c**Tergum IV **d** Gill IV **e**Paraproct**f** Seta on paraproct surface.

Figures 24 , 25 , 60b , 65a

###### Diagnosis.

**Larva.** Following combination of characters: A) labrum dorsal submarginal arc of setae composed of one plus six or seven long, simple setae; B) maxillary palp about as long as galea-lacinia, excavation on inner distal margin of segment II poorly developed; C) labial palp segment II with a compact, rounded distomedial protuberance; D) fore femur rather broad, length ca. 3× maximum width, dorsal margin with a row of ca. 34 curved, spine-like setae and many stout, pointed setae near margin; E) fore claw with one row of 11–12 denticles; F) tracheae of gills restricted to main trunk, without pigmentation; G) paraproct surface with slightly lanceolate setae and fine, simple setae.

###### Description.

**Larva** (Figs 24, 25, 60b). Body length 6.8 mm; antenna approximately twice as long as head length.

*Colouration.* Head, thorax and abdomen dorsally brown, head and thorax with bright median, dorsal suture, forewing pads with bright striation. Head, thorax and abdomen ventrally brown, legs light brown, dorsal magin of femur and tibia brown, caudal filaments brown.

*Antenna* with scape and pedicel sub-cylindrical, without distolateral process at scape; flagellum with lanceolate spines and fine, simple setae on apex of each segment.

*Labrum* (Fig. 24a). Rectangular, length 0.6× maximum width. Distal margin with medial emargination and a small process. Dorsally with medium, fine, simple setae and a few slightly lanceolate setae; submarginal arc of setae composed of one plus 6–8 long, simple setae. Ventrally with marginal row of setae composed of lateral and anterolateral long, pectinate setae and medial long, bifid, pectinate setae; ventral surface with seven short, spine-like setae near lateral and anterolateral margin.

*Right mandible* (Fig. 24b, c). Incisors fused. Outer and inner sets of denticles with 4 + 3 denticles. Inner margin of innermost denticle with a row of thin setae. Prostheca robust, apically denticulate. Margin between prostheca and mola slightly convex, with minute denticles. Tuft of setae at apex of mola present.

*Left mandible* (Fig. 24d, e). Incisors fused. Outer and inner sets of denticles with 3 + 3 denticles. Prostheca robust, apically denticulate. Margin between prostheca and mola slightly convex, with minute denticles toward subtriangular process. Subtriangular process long and slender, above level of area between prostheca and mola. Denticles of mola apically constricted. Tuft of setae at apex of mola present.

Both mandibles with lateral margins almost straight. Basal half with fine, simple setae scattered over dorsal surface.

*Hypopharynx* (Fig. 24f). Lingua longer than superlingua. Lingua about as broad as long; medial tuft of stout setae present; distal half not expanded. Superlingua rounded; lateral margin rounded; fine, long, simple setae along distal margin.

*Maxilla* (Fig. 24g). Galea-lacinia with three simple, robust apical setae under crown. Inner dorsal row of setae with three denti-setae, distal denti-seta tooth-like, middle and proximal denti-setae slender, bifid and pectinate. Medially with one bipectinate, spine-like seta and eight long, simple setae. Maxillary palp about as long as length of galea-lacinia; two segmented. Palp segment II 1.9× length of segment I. Setae on maxillary palp fine and simple, scattered over surface of segments I and II. Apex of last segment rounded, with slight excavation at inner distolateral margin.

*Labium* (Fig. 24h). Glossa basally broad, narrowing toward apex; shorter than paraglossa; inner margin with 11–12 spine-like setae increasing in length distally; apex with three long, robust, pectinate setae and one short, robust seta; outer margin with seven spine-like setae increasing in length distally; ventral surface with short, fine, simple setae. Paraglossa sub-rectangular, curved inward; apex rounded; with three rows of long, robust, apically pectinate setae; dorsally with 2–3 medium, simple setae; ventrally with five long, spine-like setae near inner margin. Labial palp with segment I 0.7× length of segments II and III combined. Segment I covered with short, fine, simple setae ventrally and micropores dorsally. Segment II with a compact, rounded distomedial protuberance; distomedial protuberance 0.5× width of base of segment III; inner and outer margin both with short, fine, simple setae; dorsally with row of five long, spine-like, simple setae. Segment III conical; apex rounded; length 1.0× width; ventrally covered with short, spine-like, simple setae and short, fine, simple setae.

*Hind wing pads* absent.

*Foreleg* (Fig. 25a, b). Ratio of foreleg segments 1.3:1.0:0.7:0.3. *Femur*. Length ca. 3× maximum width. Dorsal margin with row of ca. 34 curved, spine-like setae and with many stout, pointed setae near margin; length of setae 0.15× maximum width of femur. Apex rounded; with one curved, spine-like seta and many short, stout, pointed setae. Many stout, lanceolate setae and a few fine, simple setae along ventral margin; femoral patch poorly developed. *Tibia.* Dorsal margin with a row of short, curved, spine-like setae and long, fine, simple setae. Ventral margin with a row of curved, spine-like setae, apically longer and dense and with a tuft of long, fine, simple setae. Anterior surface scattered with many stout, lanceolate setae. Tibio-patellar suture present on basal 1/2. *Tarsus.* Dorsal margin with a row of short, spine-like setae and long, simple setae. Ventral margin with a row of curved, spine-like setae. Tarsal claw with one row of 11–14 denticles; tapering distally; with 5–7 stripes; subapical setae absent.

*Tergum* (Fig. 25c). Surface with irregular rows of U-shaped scale bases and scattered fine, simple setae, scales egg-shaped. Posterior margin of tergum IV with rounded spines, about as long as wide.

*Gills* (Fig. 25d). Present on segments II–VII. Margin with small denticles intercalating long, fine, simple setae. Tracheae restricted to main trunk. Gill IV as long as length of segments V and 1/2 VI combined. Gill VII as long as length of segment VIII.

*Paraproct* (Fig. 25e). Distally slightly expanded, with many marginal, stout spines. Surface with U-shaped scale bases and scattered fine, slightly lanceolate setae as well as fine, simple setae. Postero-lateral extension (cercotractor) with small marginal spines.

*Terminal filament* 0.6× length of cerci.

###### Etymology.

Latin for “very high”, refers to the high altitude (2900 m a.s.l.) of the type locality.

###### Distribution.

New Guinea.

###### Biological aspects.

The specimens were collected in altitudes of 2200 m a.s.l. and 2900 m a.s.l. (in forest).

###### Type-material.

**Holotype.** 1 Nymph (on slide, GBIFCH 00465187), Papua New Guinea, Simbu Prov., 05°49'S, 145°04.5'E, Mt. Wilhelm, Pindaunde Creek, 2900 m a.s.l. (in forest), S3 (oria.4), 18 Aug 1999, leg. L. Čížek. Deposited in MZL. **Paratypes.** 8 nymphs (2 on slides, GBIFCH 00465188, GBIFCH 00465189, 6 in alcohol, GBIFCH 00515266, GBIFCH 00508122, deposited in MZL), same data as holotype; 7 nymphs (1 on slide, GBIFCH 00465190, 3 in alcohol, GBIFCH 00515267, deposited in MZL; 3 in alcohol, GBIFCH 00515268, deposited in ZSM), Papua New Guinea, Eastern Highlands, Akameku-Brahmin, Bismarck Range, 2200 m, 23 Nov 2006, 05°56.80'S, 145°22.24'E, Balke & Kinibel (PNG 106).

##### 14. Labiobaetis planus sp. n.

Taxon classificationAnimaliaEphemeropteraBaetidae

http://zoobank.org/252F2D49-1C46-4F5E-9816-4D1554B3EF27

**Figure 26.**
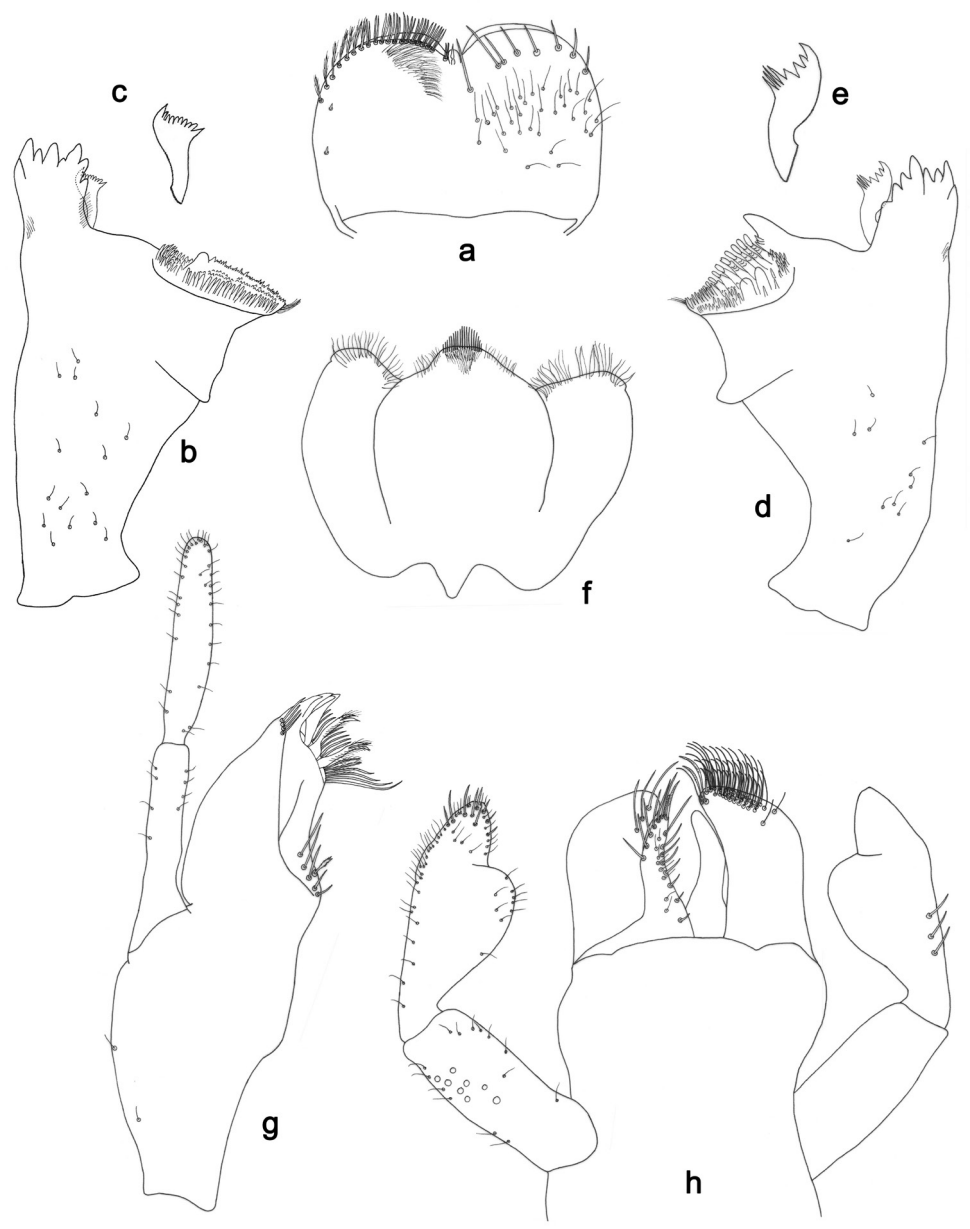
*Labiobaetisplanus* sp. n., larva morphology: **a** Labrum **b** Right mandible **c** Right prostheca **d** Left mandible **e** Left prostheca **f**Hypopharynx**g** Maxilla **h** Labium.

**Figure 27.**
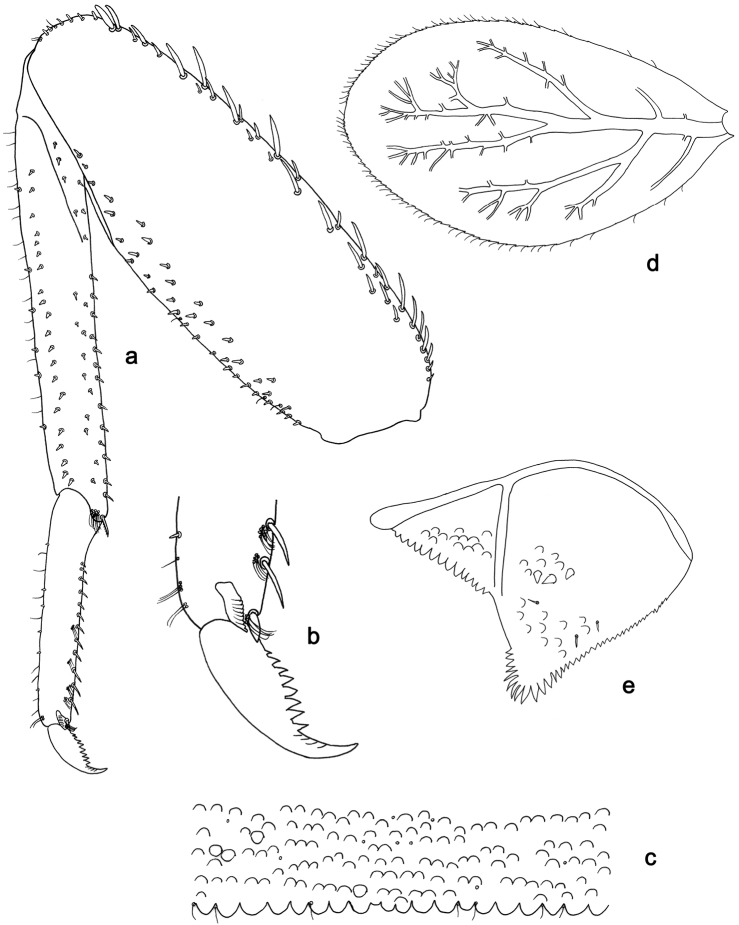
*Labiobaetisplanus* sp. n., larva morphology: **a**Foreleg**b** Fore claw **c**Tergum IV **d** Gill IV **e**Paraproct.

Figures 26 , 27 , 60c , 65a

###### Diagnosis.

**Larva.** Following combination of characters: A) labrum dorsal submarginal arc of setae composed of one plus 5–7 long, simple setae; B) maxillary palp much longer than length of galea-lacinia, apically rounded, with very poorly developed excavation at inner distolateral margin; C) labial palp segment II with a compact, rounded distomedial protuberance; D) fore femur rather broad, length ca. 3× maximum width, dorsal margin with a row of ca. 19 curved, spine-like setae and some stout, pointed setae near margin; E) fore claw with 7 – 8 denticles; F) paraproct distally expanded, surface with few slightly spatulate setae.

###### Description.

**Larva** (Figs 26, 27, 60c). Body length 3.3 mm; antenna: approximately twice as long as head length.

*Colouration.* Head, thorax and abdomen dorsally brown, head and thorax with bright median, dorsal suture, forewing pads with bright striation. Head, thorax and abdomen ventrally light brown. Legs colourless, femur with large, distomedial, brown spot and brown dorsal margin, caudal filaments light brown.

*Antenna* with scape and pedicel sub-cylindrical, without distolateral process at scape; flagellum without spines on apex of segments.

*Labrum* (Fig. 26a). Rectangular, length 0.7× maximum width. Distal margin with medial emargination and a small process. Dorsally with many medium to long, fine, simple setae; submarginal arc of setae composed of one plus 5–7 long, simple setae. Ventrally with marginal row of setae composed of lateral and anterolateral long, feathered setae and medial long, bifid setae; ventral surface with two short, spine-like setae near lateral margin.

*Right mandible* (Fig. 26b, c). Incisors fused. Outer and inner sets of denticles with 4 + 3 denticles plus one small intermediate denticle. Inner margin of innermost denticle with a row of thin setae. Prostheca robust, apically denticulate. Margin between prostheca and mola straight. Tuft of setae at apex of mola present.

*Left mandible* (Fig. 26d, e). Incisors fused. Outer and inner sets of denticles with 4 + 4 denticles. Prostheca robust, apically with small denticles and comb-shape structure. Margin between prostheca and mola straight. Subtriangular process long and slender, above level of area between prostheca and mola. Denticles of mola apically constricted. Tuft of setae at apex of mola present.

Both mandibles with lateral margins almost straight. Basal half with fine, simple setae scattered over dorsal surface.

*Hypopharynx* (Fig. 26f). Lingua about as long as superlingua. Lingua about as broad as long; medial tuft of stout setae present; distal half not expanded. Superlingua rounded; lateral margin rounded; fine, long, simple setae along distal margin.

*Maxilla* (Fig. 26g). Galea lacinia with four simple, robust apical setae under crown. Inner dorsal row of setae with three denti-setae, distal denti-seta tooth-like, middle and proximal denti-setae slender, bifid and pectinate. Medially with one bipectinate, spine-like seta and five long, simple setae. Maxillary palp 1.6× as long as length of galea-lacinia; two segmented. Palp segment II 1.2× length of segment I. Setae on maxillary palp fine and simple, scattered over surface of segments I and II. Apex of last segment rounded, with very slight excavation at inner distolateral margin.

*Labium* (Fig. 26h). Glossa basally broad, narrowing toward apex; shorter than paraglossa; inner margin with 7–8 spine-like setae increasing in length distally; apex with two long and one medium, robust, pectinate setae; outer margin with five long, spine-like setae; ventral surface with short, fine, simple, scattered setae. Paraglossa sub-rectangular, curved inward; apex rounded; with three rows of long, robust, apically pectinate setae; dorsally with 2–3 medium, simple setae; ventrally with 3–4 long, spine-like setae near inner margin. Labial palp with segment I 0.8× length of segments II and III combined. Segment I covered with short, fine, simple setae ventrally and micropores dorsally. Segment II with a compact, rounded distomedial protuberance; distomedial protuberance 0.4× width of base of segment III; inner and outer margin both with short, fine, simple setae; dorsally with a row of 3–4 long, spine-like, simple setae. Segment III conical; apex truncate; length 0.9× width; ventrally covered with short and medium spine-like, simple setae and short, fine, simple setae.

*Hind wing pads* absent.

*Foreleg* (Fig. 27a, b). Ratio of foreleg segments 1.2:1.0:0.5:0.2. *Femur*. Length ca. 3× maximum width. Dorsal margin with a row of ca. 19 curved, spine-like setae and with some stout, pointed setae near margin; length of setae 0.2× maximum width of femur. Apex rounded; with one pair of spine-like setae and some short, stout, pointed setae. Many stout, lanceolate setae and a few fine, simple setae along ventral margin; femoral patch poorly developed. *Tibia.* Dorsal margin with a row of short, curved, spine-like setae and a row of long, fine, simple setae and a row of stout, lanceolate setae near margin. Ventral margin with a row of curved, spine-like setae, on apex one bipectinate, spine-like seta and a tuft of long, fine, simple setae. Anterior surface scattered with stout, lanceolate setae. Tibio-patellar suture present on basal 1/3. *Tarsus.* Dorsal margin with a row of short, spine-like setae and long, simple setae. Ventral margin with a row of curved, spine-like setae. Tarsal claw with one row of 7–8 denticles; distally pointed; with four stripes; subapical setae absent.

*Tergum* (Fig. 27c). Surface with irregular rows of U-shaped scale bases and scattered micropores, scales short, apically rounded. Posterior margin of tergum IV with rounded spines, wider than long.

*Gills* (Fig. 27d). Present on segments II–VII. Margin with small denticles intercalating long, fine, simple setae. Tracheae extending from main trunk to inner and outer margins. Gill IV as long as length of segments V and VI combined. Gill VII as long as length of segments VIII and IX combined.

*Paraproct* (Fig. 27e). Distally expanded, with many marginal, stout spines. Surface with U-shaped scale bases and scattered fine, slightly lanceolate setae. Postero-lateral extension (cercotractor) with small, marginal spines.

###### Etymology.

Refers to the low altitude (95 m a.s.l.) of the type locality.

###### Distribution.

New Guinea.

###### Biological aspects.

The specimens were collected at an altitude of 95 m a.s.l.

###### Type-material.

**Holotype.** Nymph (on slide, GBIFCH 00465191), Indonesia, Papua, Sorong, inland, 95 m, 19 Feb 2006, 00°49.35'S, 131°24.20'E, Balke & Tindige (BH 20). Temporary deposited in MZL before definitly housed in MZB. **Paratypes.** 16 nymphs (2 on slides, GBIFCH 00465192, GBIFCH 00465193, 9 in alcohol, GBIFCH 00515269, GBIFCH 00508149, GBIFCH 00508150, deposited in MZL; 5 in alcohol, GBIFCH 00515270, deposited in ZSM), same data as holotype.

##### 15. Labiobaetis podolakae sp. n.

Taxon classificationAnimaliaEphemeropteraBaetidae

http://zoobank.org/61BDC588-005D-40ED-A206-90E0548A205B

**Figure 28.**
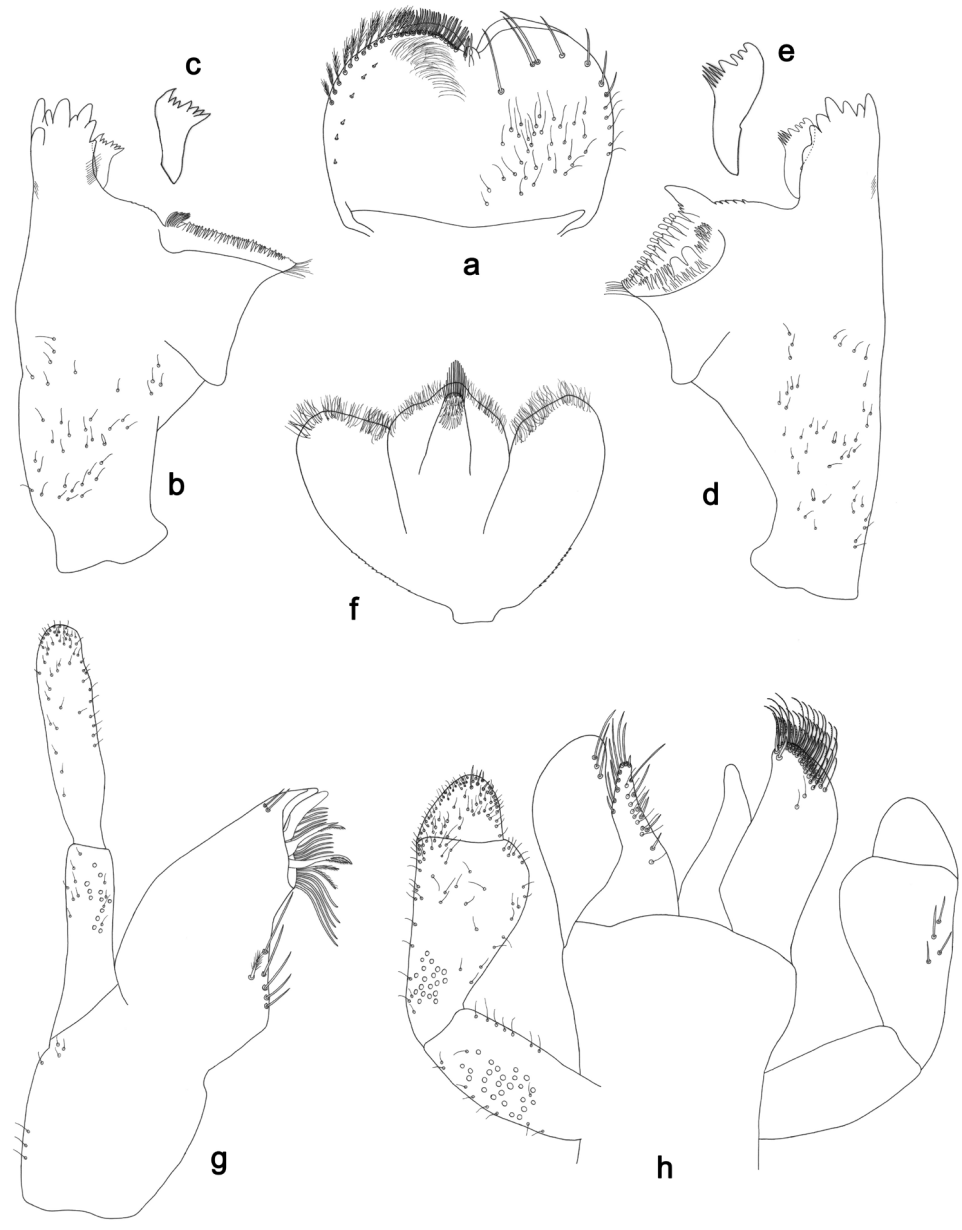
*Labiobaetispodolakae* sp. n., larva morphology: **a** Labrum **b** Right mandible **c** Right prostheca **d** Left mandible **e** Left prostheca **f**Hypopharynx**g** Maxilla **h** Labium.

**Figure 29.**
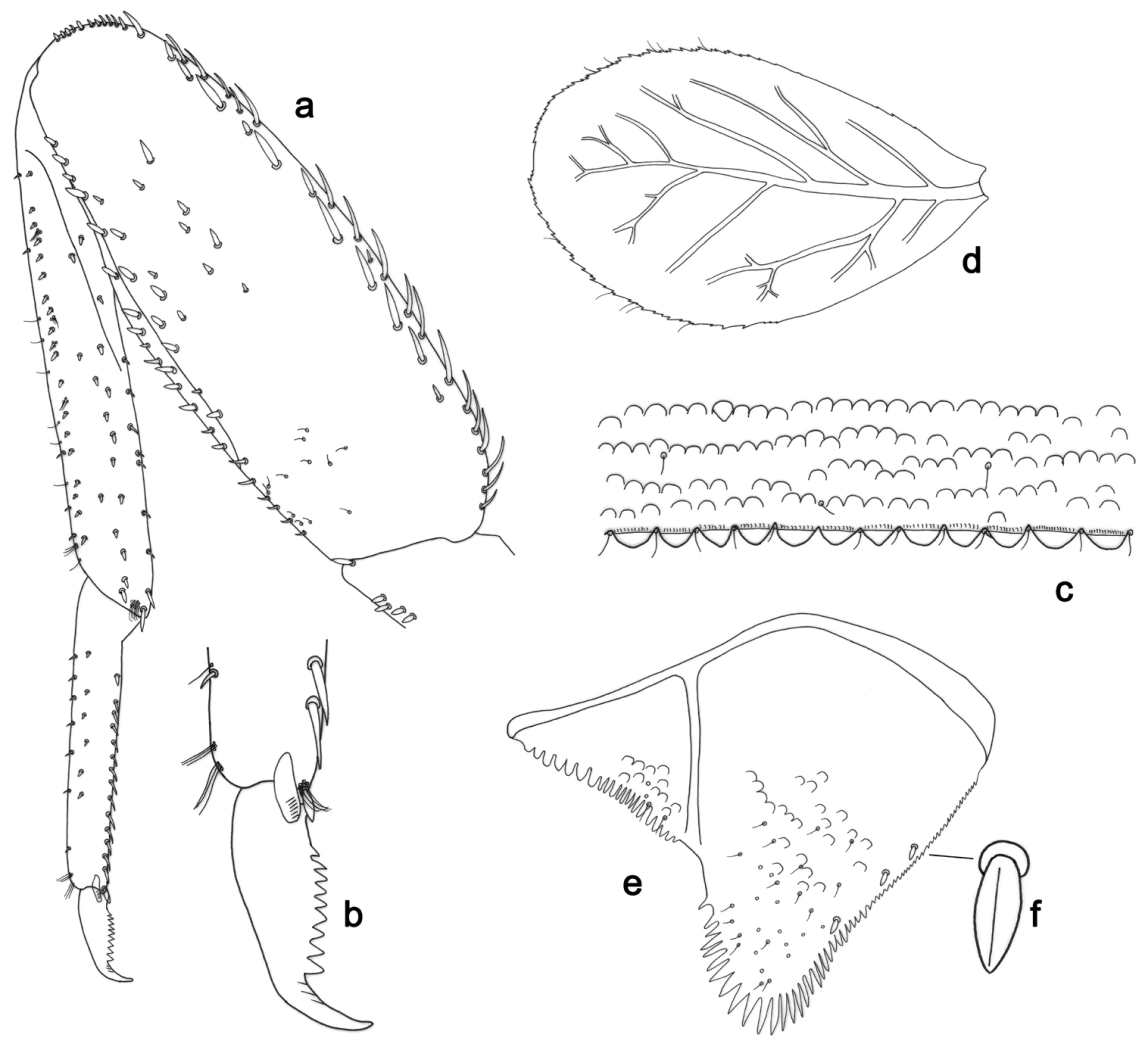
*Labiobaetispodolakae* sp. n., larva morphology: **a**Foreleg**b** Fore claw **c**Tergum IV **d** Gill IV **e**Paraproct**f** Seta near paraproct margin.

Figures 28 , 29 , 60d , 65a

###### Diagnosis.

**Larva.** Following combination of characters: A) labrum dorsal submarginal arc of setae composed of one plus six long, simple setae; B) maxillary palp 1.2× length of galea-lacinia; C) labial palp segment II with a compact, rounded distomedial protuberance; D) fore femur rather broad, length ca. 3× maximum width, dorsal margin with a row of ca. 16 curved, spine-like setae and a row of spine-like setae near margin; E) paraproct with three robust, lanceolate setae near distolateral margin.

###### Description.

**Larva** (Figs 28, 29, 60d). Body length 4.7 mm.

*Colouration.* Head, thorax and abdomen dorsally brown, head and thorax with bright median, dorsal suture, forewing pads with bright striation. Head, thorax and abdomen ventrally light brown, femur dorsal margin brown, legs otherwise colourless, caudal filaments light brown.

*Antenna* with scape and pedicel sub-cylindrical, without distolateral process at scape; flagellum with broad spines on apex of each segment.

*Labrum* (Fig. 28a). Rectangular, length 0.7× maximum width. Distal margin with medial emargination and a small process. Dorsally with many medium to long, fine, simple setae; submarginal arc of setae composed of one plus six long, simple setae. Ventrally with marginal row of setae composed of lateral and anterolateral long, feathered setae and medial long, bifid, pectinate setae; ventral surface with six short, spine-like setae near lateral and anterolateral margin.

*Right mandible* (Fig. 28b, c). Incisors fused. Outer and inner sets of denticles with 4 + 3 denticles plus one small intermediate denticle. Inner margin of innermost denticle with a row of thin setae. Prostheca robust, apically denticulate. Margin between prostheca and mola slightly convex, with minute denticles. Tuft of setae at apex of mola present.

*Left mandible* (Fig. 28d, e). Incisors fused. Outer and inner sets of denticles with 3 + 4 denticles. Prostheca robust, apically with small denticles and comb-shape structure. Margin between prostheca and mola straight, with minute denticles towards subtriangular process. Subtriangular process long and slender, above level of area between prostheca and mola. Denticles of mola apically constricted. Tuft of setae at apex of mola present.

Both mandibles with lateral margins almost straight. Basal half with fine, simple setae scattered over dorsal surface.

*Hypopharynx* (Fig. 28f). Lingua longer than superlingua. Lingua longer than broad; medial tuft of stout setae present; distal half laterally expanded. Superlingua rounded; lateral margin rounded; fine, long, simple setae along distal margin.

*Maxilla* (Fig. 28g). Galea-lacinia with two simple, robust apical setae under crown. Inner dorsal row of setae with three denti-setae, distal denti-seta tooth-like, middle and proximal denti-setae slender, bifid and pectinate. Medially with one bipectinate, spine-like seta and five long, simple setae. Maxillary palp 1.2× as long as length of galea-lacinia; two segmented. Palp segment II 1.4× length of segment I. Setae on maxillary palp fine and simple, scattered over surface of segments I and II. Apex of last segment rounded, with slight excavation at inner distolateral margin.

*Labium* (Fig. 28h). Glossa basally broad, narrowing toward apex; shorter than paraglossa; inner margin with six spine-like setae increasing in length distally; apex with two long and one short, robust, pectinate setae; outer margin with five long, spine-like setae increasing in length distally; ventral surface with few short, fine, simple setae. Paraglossa sub-rectangular, curved inward; apex rounded; with three rows of long, robust, apically pectinate setae; dorsally with 2–3 medium, simple setae; ventrally with three long, spine-like setae near inner margin. Labial palp with segment I 0.6× length of segments II and III combined. Segment I covered with micropores dorsally and ventrally with fine, simple setae along margins. Segment II with a compact, rounded distomedial protuberance; distomedial protuberance 0.3× width of base of segment III; inner and outer margin both with short, fine, simple setae; dorsally with a row of four long, spine-like, simple setae. Segment III conical; apex rounded; length 1.1× width; ventrally covered with medium spine-like, simple setae and short, fine, simple setae.

*Hind wing pads* absent.

*Foreleg* (Fig. 29a, b). Ratio of foreleg segments 1.2:1.0:0.5:0.2. *Femur*. Length ca. 3× maximum width. Dorsal margin with row of ca. 16 curved, spine-like setae and a row of stout, pointed setae near margin; length of setae 0.2× maximum width of femur. Apex rounded; with one curved, spine-like seta and many short, stout, pointed setae. Many stout, lanceolate setae and a few fine, simple setae along ventral margin; femoral patch poorly developed. *Tibia.* Dorsal margin with a row of short, curved, spine-like setae and a row of long, fine, simple setae, and a row of stout, lanceolate setae near margin. Ventral margin with a row of short, spine-like setae, on apex one stout, spine-like seta and a tuft of long, fine, simple setae. Anterior surface scattered with many stout, lanceolate setae. Tibio-patellar suture present on basal 1/2. *Tarsus.* Dorsal margin with a row of short, spine-like setae and long, simple setae. Ventral margin with a row of curved, spine-like setae. Tarsal claw with one row of nine denticles; distally pointed; with three stripes; subapical setae absent.

*Tergum* (Fig. 29c). Surface with irregular rows of U-shaped scale bases and scattered fine, simple setae, scales egg-shaped. Posterior margin of tergum IV with rounded spines, wider than long.

*Gills* (Fig. 29d). Present on segments II–VII. Margin with alternating smaller and bigger denticles intercalating long, fine, simple setae. Tracheae extending from main trunk to inner and outer margins. Gill IV as long as length of segments V and 1/2 VI combined. Gill VII as long as length of segments VIII and 2/3 IX combined.

*Paraproct* (Fig. 29e, f). Distally expanded, with many marginal, stout spines. Surface with U-shaped scale bases, scattered micropores and fine, simple setae and three robust, lanceolate setae near lateral margin. Postero-lateral extension (cercotractor) with small marginal spines.

###### Etymology.

Dedicated to Marion Podolak (Museum for Zoology Lausanne, MZL) for her valuable support during our study.

###### Distribution.

New Guinea.

###### Type-material.

**Holotype.** Nymph (on slide, GBIFCH 00465227), Papua New Guinea, Eastern Highlands, Aiyura, 1710 m, 15 Jan 2003, 06°21.41'S, 145°54.34'E, grassland stream, K. Sagata leg. Deposited in ZSM. **Paratypes.** 2 nymphs (1 on slide, GBIFCH 00465194, 1 in alcohol, GBIFCH 00515276, deposited in MZL), same data as holotype.

##### 16. Labiobaetis rutschmannae sp. n.

Taxon classificationAnimaliaEphemeropteraBaetidae

http://zoobank.org/38BEAA38-8CF9-4AA9-B221-AF7CC5436773

**Figure 30.**
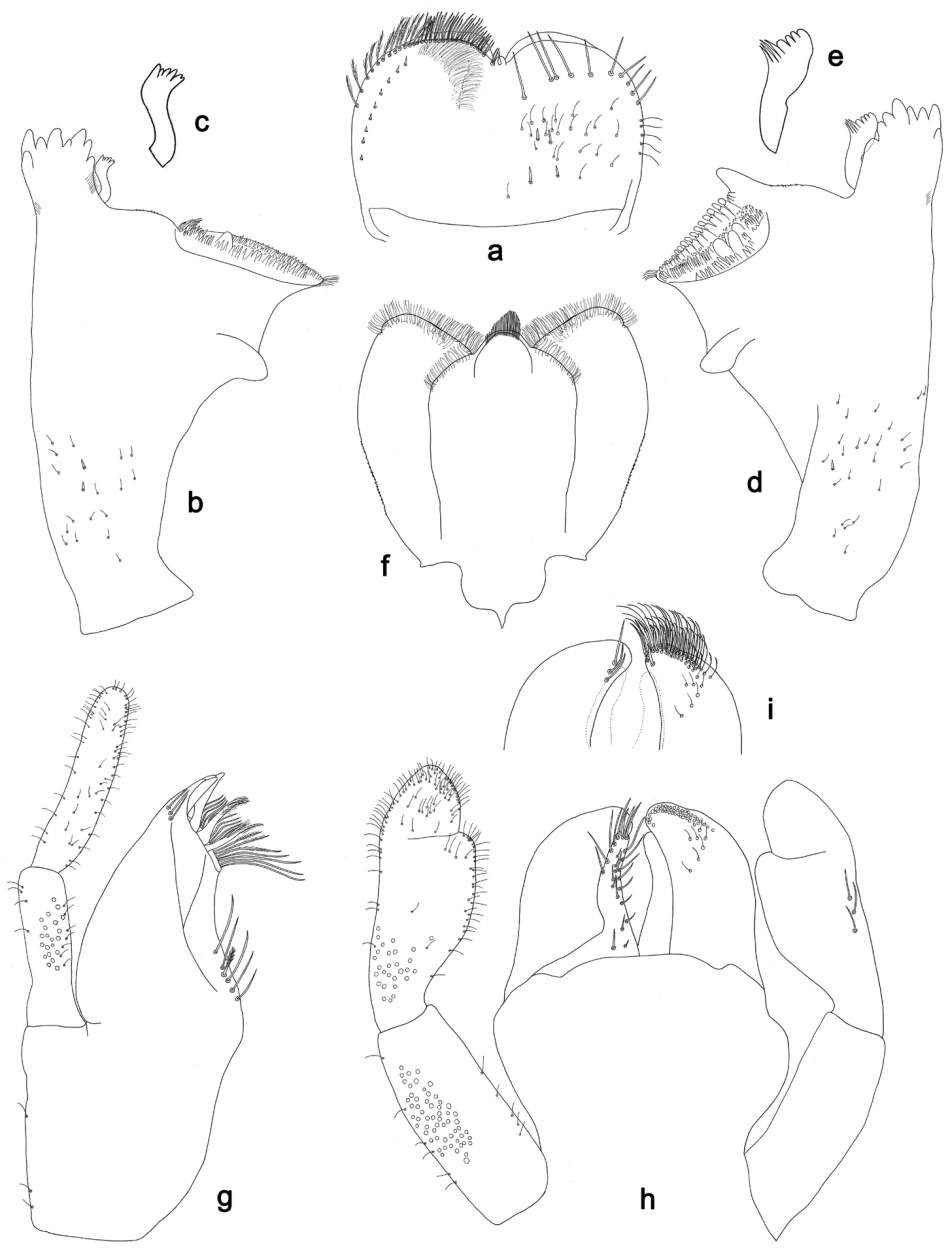
*Labiobaetisrutschmannae* sp. n., larva morphology: **a** Labrum **b** Right mandible **c** Right prostheca **d** Left mandible **e** Left prostheca **f**Hypopharynx**g** Maxilla **h** Labium.

**Figure 31.**
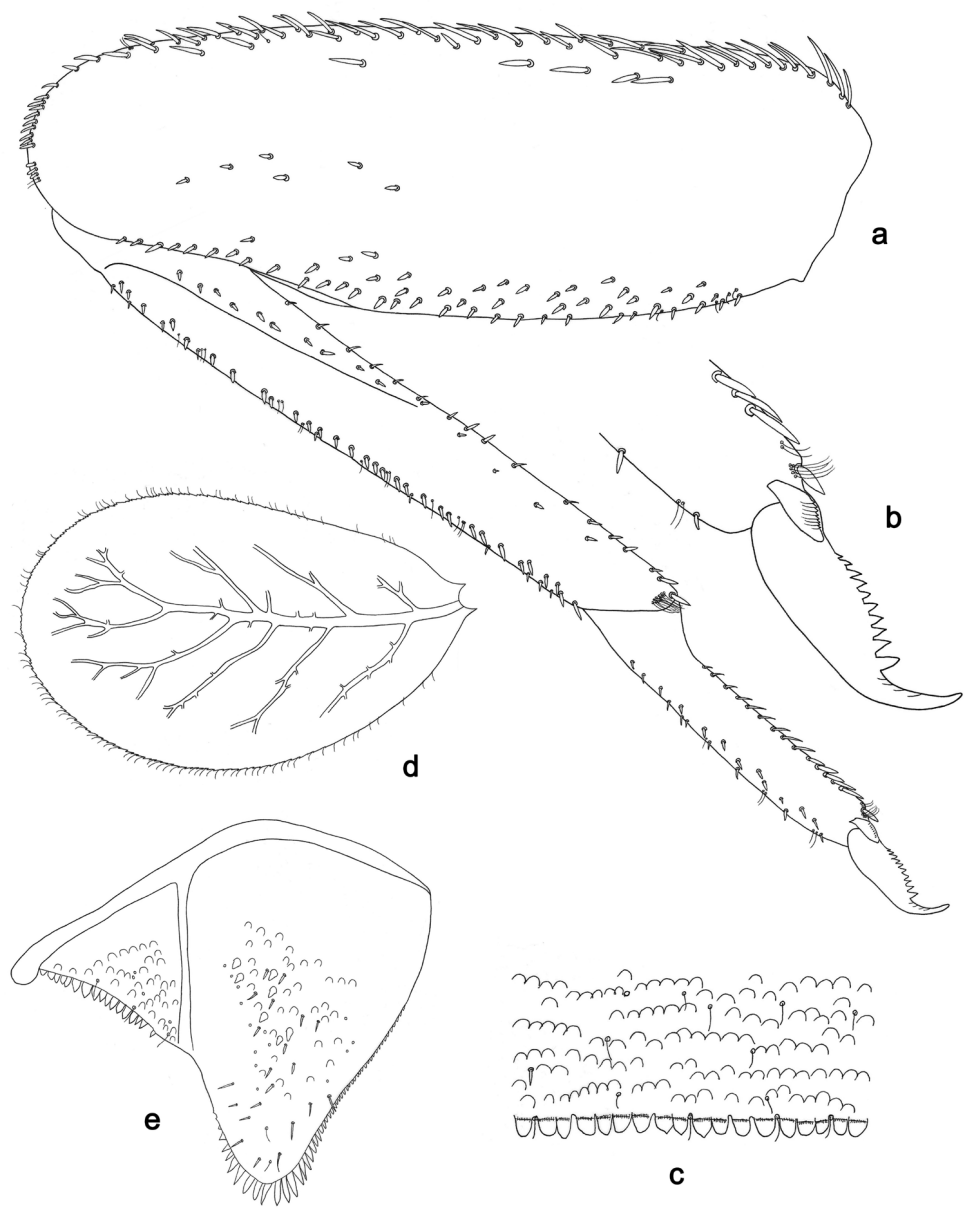
*Labiobaetisrutschmannae* sp. n., larva morphology: **a**Foreleg**b** Fore claw **c**Tergum IV **d** Gill IV **e**Paraproct.

Figures 30 , 31 , 61a , 65a

###### Diagnosis.

**Larva.** Following combination of characters: A) labrum dorsal submarginal arc of setae composed of one plus nine long, simple setae ; B) maxillary palp longer than length of galea-lacinia, apically rounded, without excavation at inner distolateral margin; C) labial palp segment II with a compact, rounded distomedial protuberance; D) fore femur rather broad, length ca. 3× maximum width, dorsal margin with a row of ca. 26 curved, spine-like setae and many stout, pointed setae near margin; E) paraproct distally expanded, surface with slightly spatulate setae.

###### Description.

**Larva** (Figs 30, 31, 61a). Body length 5.2 mm.

*Colouration.* Head, thorax and abdomen dorsally brown, head and thorax with bright median, dorsal suture. Head, thorax and abdomen ventrally light brown. Femur dorsal margin light brown, legs otherwise colourless, caudal filaments basally light brown, otherwise colourless.

*Antenna* with scape and pedicel sub-cylindrical, without distolateral process at scape; flagellum with broad, lanceolate spines and fine, simple setae on apex of each segment.

*Labrum* (Fig. 30a). Rectangular, length 0.7× maximum width. Distal margin with medial emargination and a small process. Dorsally with medium, fine, simple setae and a few slightly spatulate setae; submarginal arc of setae composed of one plus nine long, simple setae. Ventrally with marginal row of setae composed of lateral and anterolateral long, feathered setae and medial long, bifid setae; ventral surface with nine short, spine-like setae near lateral and anterolateral margin.

*Right mandible* (Fig. 30b, c). Incisors fused. Outer and inner sets of denticles with 4 + 3 denticles plus one small intermediate denticle. Inner margin of innermost denticle with a row of thin setae. Prostheca robust, apically denticulate. Margin between prostheca and mola slightly convex, with minute denticles. Tuft of setae at apex of mola present.

*Left mandible* (Fig. 30d, e). Incisors fused. Outer and inner sets of denticles with 4 + 4 denticles. Prostheca robust, apically with small denticles and comb-shape structure. Margin between prostheca and mola slightly convex, with minute denticles toward subtriangular process. Subtriangular process long and slender, above level of area between prostheca and mola. Denticles of mola apically constricted. Tuft of setae at apex of mola present.

Both mandibles with lateral margins almost straight. Basal half with fine, simple setae scattered over dorsal surface.

*Hypopharynx* (Fig. 30f). Lingua shorter than superlingua. Lingua longer than broad; medial tuft of stout setae present; distal half not expanded. Superlingua rounded; lateral margin rounded; fine, long, simple setae along distal margin.

*Maxilla* (Fig. 30g). Galea-lacinia with three simple, robust apical setae under crown. Inner dorsal row of setae with three denti-setae, distal denti-seta tooth-like, middle and proximal denti-setae slender, bifid and pectinate. Medially with one bipectinate, spine-like seta and five long, simple setae. Maxillary palp 1.2× as long as length of galea-lacinia; two segmented. Palp segment II 1.2× length of segment I. Setae on maxillary palp fine and simple, scattered over surface of segments I and II. Apex of last segment rounded, without excavation at inner distolateral margin.

*Labium* (Fig. 30h, i). Glossa basally broad, narrowing toward apex; shorter than paraglossa; inner margin with 7–8 spine-like setae increasing in length distally; apex with three long, robust setae; outer margin with five long, spine-like setae; ventral surface with short, simple setae. Paraglossa sub-rectangular, curved inward; apex rounded; with three rows of long, robust, apically pectinate setae; dorsally with seven simple setae; ventrally with three long, spine-like setae near inner margin. Labial palp with segment I 0.9× length of segments II and III combined. Segment I covered with micropores dorsally and ventrally with fine, simple setae along margins. Segment II with a compact, rounded distomedial protuberance; distomedial protuberance 0.2× width of base of segment III; inner and outer margin both with short, fine, simple setae; dorsally with row of three long, spine-like setae. Segment III conical; apex rounded; length 0.9× width; ventrally covered with short, fine, simple setae.

*Hind wing pads* absent.

*Foreleg* (Fig. 31a, b). Ratio of foreleg segments 1.2:1.0:0.5:0.2. *Femur*. Length ca. 3× maximum width. Dorsal margin with a row of ca. 26 curved, spine-like setae and with many stout, pointed setae near margin; length of setae 0.16× maximum width of femur. Apex rounded; with some spine-like setae and many short, stout, pointed or apically rounded setae. Many stout, lanceolate setae and a few fine, simple setae along ventral margin; femoral patch poorly developed. *Tibia.* Dorsal margin with a row of stout, lanceolate setae and very fine, simple setae. Ventral margin with a row of curved, spine-like setae, on apex one stout, spine-like seta and a tuft of long, fine, simple setae. Anterior surface scattered with stout, lanceolate setae. Tibio-patellar suture present on basal 1/2. *Tarsus.* Dorsal margin with a row of short, spine-like setae and long, simple setae. Ventral margin with a row of curved, spine-like setae. Tarsal claw with one row of 8–10 denticles; distally pointed; with four stripes; subapical setae absent.

*Tergum* (Fig. 31c). Surface with irregular rows of U-shaped scale bases and scattered fine, simple setae. Posterior margin of tergum IV with rounded or triangular spines, wider than long.

*Gills* (Fig. 31d). Present on segments II - VII. Margin with small denticles intercalating long, fine, simple setae. Tracheae extending from main trunk to inner and outer margins. Gill IV as long as length of segments V and VI combined. Gill VII as long as length of segments VIII and 1/2 IX combined.

*Paraproct* (Fig. 31e). Distally expanded, with many marginal, stout spines. Surface with U-shaped scale bases and scattered fine, slightly lanceolate setae, fine, simple setae and micropores. Postero-lateral extension (cercotractor) with small marginal spines.

###### Etymology.

Dedicated to Sereina Rutschmann (University of Vigo, Spain) for her long-lasting support of our group in genetics.

###### Distribution.

New Guinea.

###### Biological aspects.

The specimens were collected in altitudes of 1800 m–2000 m a.s.l.

###### Type-material.

**Holotype.** Nymph (on slide, GBIFCH 00465195), Papua New Guinea, Western Highlands, Simbai, 1800–2000 m, 01 Mar 2007, 05°14.28'S, 144°28.74'E, Kinibel (PNG 138). Deposited in ZSM. **Paratype.** Nymph (on slide, GBIFCH 00465196, deposited in MZL), Papua New Guinea, Western Highlands, Simbai, 2000 m, 28 Feb 2007, 05°15.17'S, 144°32.81'E, Kinibel (PNG 136).

##### 17. Labiobaetis schwanderae sp. n.

Taxon classificationAnimaliaEphemeropteraBaetidae

http://zoobank.org/C745A8D1-2E6C-4F35-BD62-3C0E1DF94168

**Figure 32.**
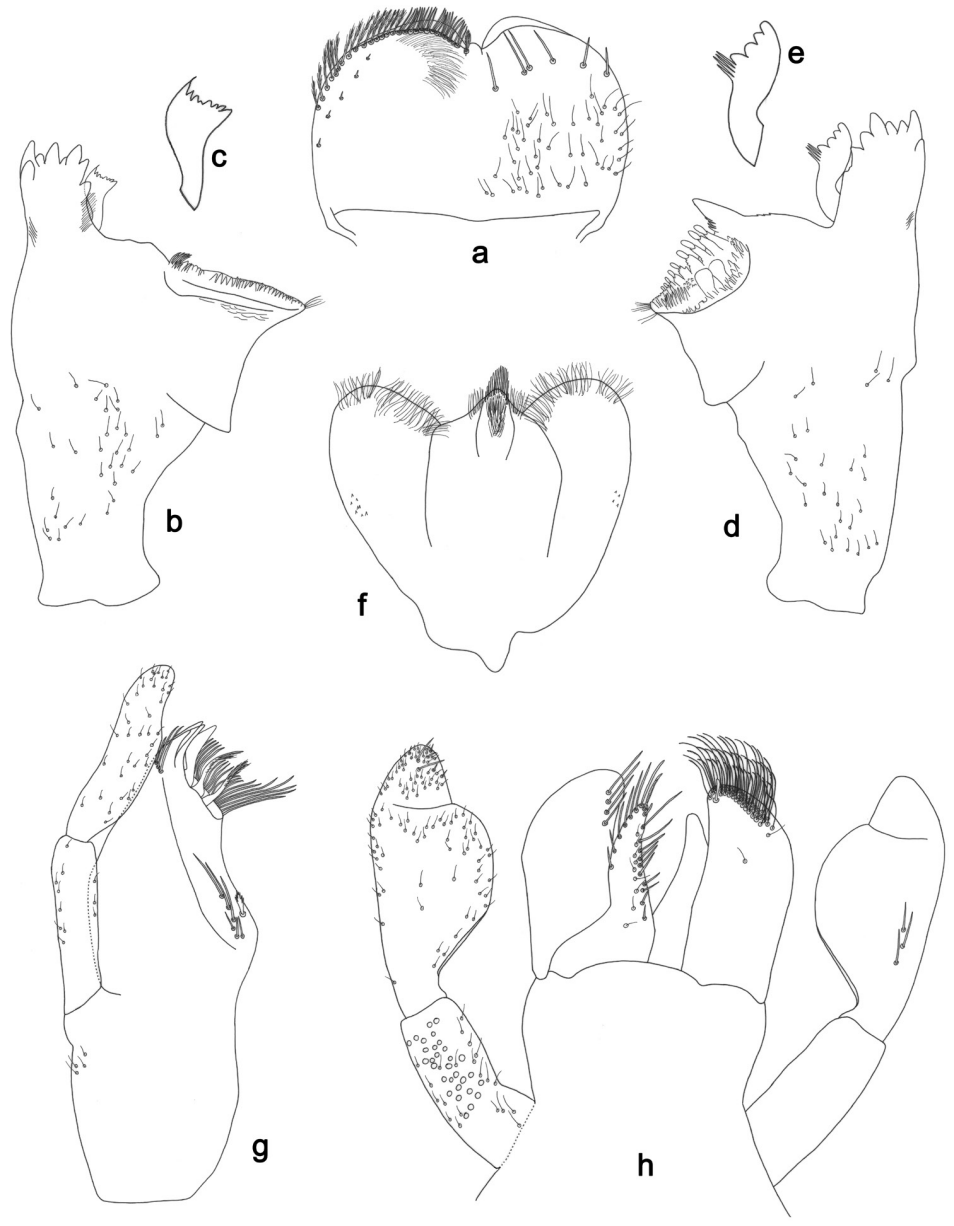
*Labiobaetisschwanderae* sp. n., larva morphology: **a** Labrum **b** Right mandible **c** Right prostheca **d** Left mandible **e** Left prostheca **f**Hypopharynx**g** Maxilla **h** Labium.

**Figure 33.**
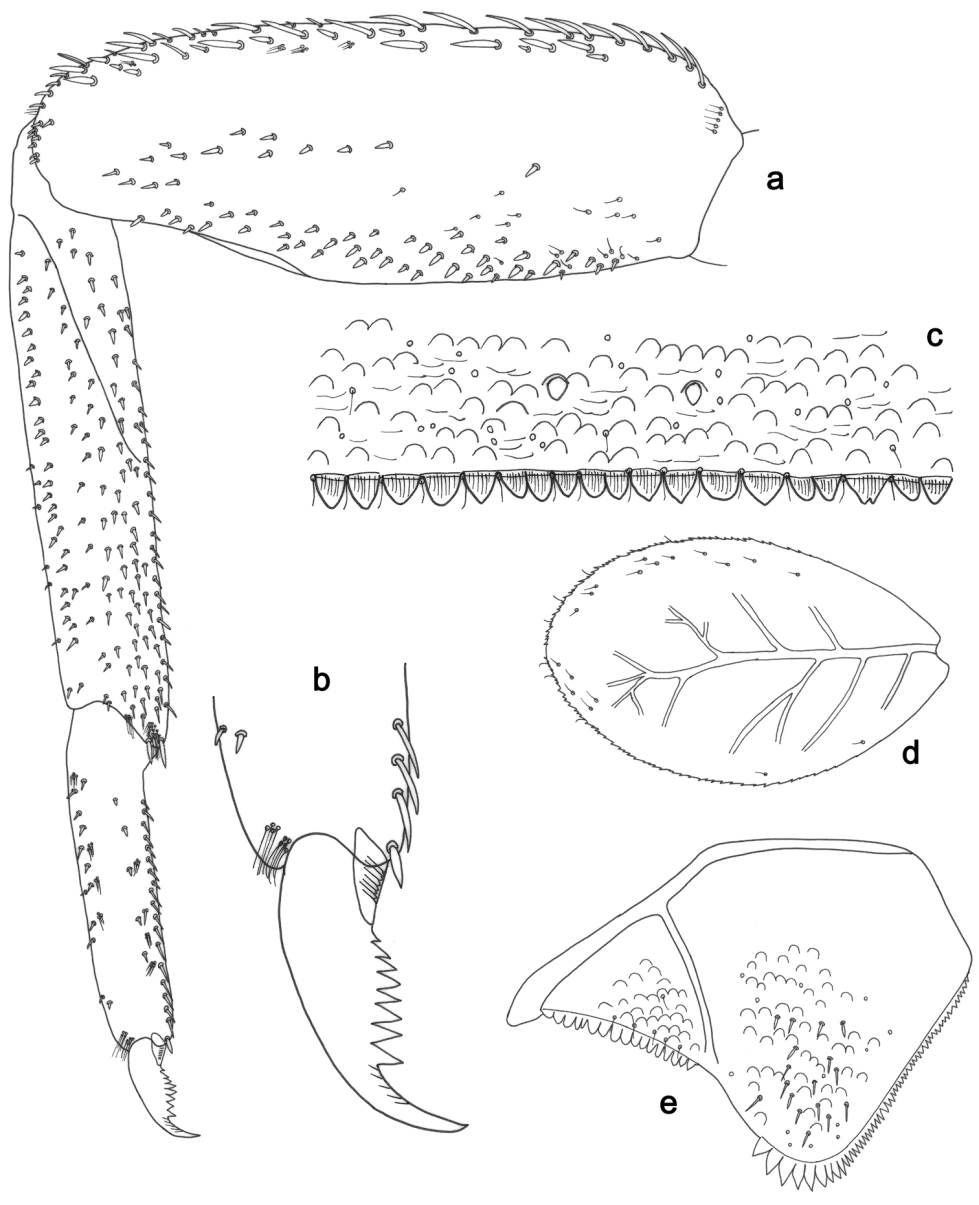
*Labiobaetisschwanderae* sp. n., larva morphology: **a**Foreleg**b** Fore claw **c**Tergum IV **d** Gill IV **e**Paraproct.

Figures 32 , 33 , 61b , 65a

###### Diagnosis.

**Larva.** Following combination of characters: A) labrum dorsal arc of setae composed of one plus five long, simple setae; B) maxillary palp 1.3× as long as length of galea-lacinia; segment II apically rounded, with an excavation at inner lateral margin; C) labial palp segment II with a compact, rounded distomedial protuberance, segment III conical; D) fore femur rather broad, length 2.7× maximum width, dorsal margin with a row of ca. 21 curved, spine-like setae and many stout, pointed setae near margin; E) fore claw with a row of 8 denticles; F) spines at posterior margin of tergum IV mostly rounded, about as long as wide; G) paraproct surface with U-shaped scale bases and scattered fine, slightly spatulate setae and micropores.

###### Description.

**Larva** (Figs 32, 33, 61b). Body length 5 mm; antenna: approximately twice as long as head length.

*Colouration.* Head, thorax and abdomen dorsally brown, head and thorax with bright median, dorsal suture. Head, thorax and abdomen ventrally colourless. Legs colourless with some brown spots, femur with brown dorsal margin, caudal filaments brown.

*Antenna* with scape and pedicel sub-cylindrical, without distolateral process at scape; flagellum with broad, lanceolate spines and fine, simple setae on apex of each segment.

*Labrum* (Fig. 32a). Rectangular, length 0.7× maximum width. Distal margin with medial emargination and a small process. Dorsally with medium, fine, simple setae scattered over surface; submarginal arc of setae composed of one plus five long, simple setae. Ventrally with marginal row of setae composed of lateral and anterolateral long, feathered setae and medial long, bifid, pectinate setae; ventral surface with five short, spine-like setae near lateral and anterolateral margin.

*Right mandible* (Fig. 32b, c). Incisors fused. Outer and inner sets of denticles with 4 + 3 denticles plus one small intermediate denticle. Inner margin of innermost denticle with a row of thin setae. Prostheca robust, apically denticulate. Margin between prostheca and mola slightly convex. Tuft of setae at apex of mola present.

*Left mandible* (Fig. 32d, e). Incisors fused. Outer and inner sets of denticles with 4 + 4 denticles. Prostheca robust, apically with small denticles and comb-shape structure. Margin between prostheca and mola straight, with minute denticles towards subtriangular process. Subtriangular process long and slender, above level of area between prostheca and mola. Denticles of mola apically constricted. Tuft of setae at apex of mola present.

Both mandibles with lateral margins almost straight. Basal half with fine, simple setae scattered over dorsal surface.

*Hypopharynx* (Fig. 32f). Lingua shorter than superlingua. Lingua longer than broad; medial tuft of stout setae present; distal half laterally expanded. Superlingua rounded; lateral margin rounded; fine, long, simple setae along distal margin.

*Maxilla* (Fig. 32g). Galea-lacinia with three simple, robust apical setae under crown. Inner dorsal row of setae with three denti-setae, distal denti-seta tooth-like, middle and proximal denti-setae slender, bifid and pectinate. Medially with one spine-like seta and six long, simple setae. Maxillary palp 1.3× as long as length of galea-lacinia; two segmented. Palp segment II 1.2× length of segment I. Setae on maxillary palp fine and simple, scattered over surface of segments I and II. Apex of last segment rounded, with excavation at inner distolateral margin.

*Labium* (Fig. 32h). Glossa basally broad, narrowing toward apex; shorter than paraglossa; inner margin with ten spine-like setae increasing in length distally; apex with two long and one short, robust, pectinate setae; outer margin with six long spine-like setae increasing in length distally; ventral surface with few short, fine, simple setae. Paraglossa sub-rectangular, curved inward; apex rounded; with three rows of long, robust, apically pectinate setae; dorsally with two medium, simple setae; ventrally with four long, spine-like setae near inner margin. Labial palp with segment I 0.6× length of segments II and III combined. Segment I covered with short and medium, fine, simple setae ventrally and with micropores dorsally. Segment II with a compact, rounded distomedial protuberance; distomedial protuberance 0.4× width of base of segment III; inner and outer margin both with short, fine, simple setae; dorsally with row of three long, spine-like setae. Segment III conical; apex rounded; length 0.8× width; ventrally covered with short and medium spine-like, simple setae and short, fine, simple setae.

*Hind wing pads* absent.

*Foreleg* (Fig. 33a, b). Ratio of foreleg segments 1.2:1.0:0.6:0.2. *Femur*. Length ca. 3× maximum width. Dorsal margin with a row of ca. 21 curved, spine-like setae and with many stout, pointed setae near margin; length of setae 0.2× maximum width of femur. Apex rounded; with one pair of curved, spine-like setae and many short, stout, pointed setae. Many stout, lanceolate setae and a few fine, simple setae scattered along ventral margin; femoral patch poorly developed. *Tibia.* Dorsal margin with a few curved, spine-like setae. Ventral margin with a row of curved, spine-like setae and some longer, spine-like, bipectinate setae and a tuft of long, fine, simple setae on apex. Anterior surface scattered with many stout, lanceolate setae. Tibio-patellar suture present on basal 1/2. *Tarsus.* Dorsal margin with a row of short, curved, spine-like setae and long, simple setae. Ventral margin with a row of curved, spine-like setae. Tarsal claw with one row of eight denticles; distally pointed; with five stripes; subapical setae absent.

*Tergum* (Fig. 33c). Surface with irregular rows of U-shaped scale bases and scattered fine, simple setae and micropores, scales egg-shaped. Posterior margin of tergum IV with rounded or triangular spines, about as long as wide.

*Gills* (Fig. 33d). Present on segments II–VII. Margin with small denticles intercalating long, fine, simple setae. Tracheae extending from main trunk to inner and outer margins. Gill IV as long as length of segments V and 1/2 VI combined. Gill VII as long as length of segments VIII and 1/3 IX combined.

*Paraproct* (Fig. 33e). Distally slightly expanded, with many marginal, stout spines. Surface with U-shaped scale bases and scattered fine, slightly lanceolate setae and micropores. Postero-lateral extension (cercotractor) with small marginal spines.

###### Etymology.

Dedicated to Tanja Schwander (University of Lausanne, UNIL) for her constant support during a master project of one of the authors (TK) in her lab.

###### Distribution.

New Guinea.

###### Biological aspects.

The specimens were collected at an altitude of 1400 m a.s.l.

###### Type-material.

**Holotype.** Nymph (on slide, GBIFCH 00465197), Papua New Guinea, Gulf, Marawaka, 1400 m, 11 Nov 2006, 07°05.66'S, 145°44.47'E, Balke & Kinibel (PNG 90). Deposited in ZSM. **Paratypes.** 2 nymphs (1 on slide, GBIFCH 00465198, 5 in alcohol, GBIFCH 00515234, deposited in MZL; 3 in alcohol, GBIFCH 00515235, deposited in ZSM), same data as holotype.

#### *L.vitilis* group of species

The group is characterized by a short, thumb-like protuberance of labial palp segment II and a dorsal, submarginal arc of setae composed of simple setae. Additionally, there is no distolateral excavation at maxillary palp segment II.

##### 18. Labiobaetis vitilis

Taxon classificationAnimaliaEphemeropteraBaetidae

(Lugo-Ortiz & McCafferty, 1999)

Figures 34 , 65b

###### Diagnosis.

**Figure 34.**
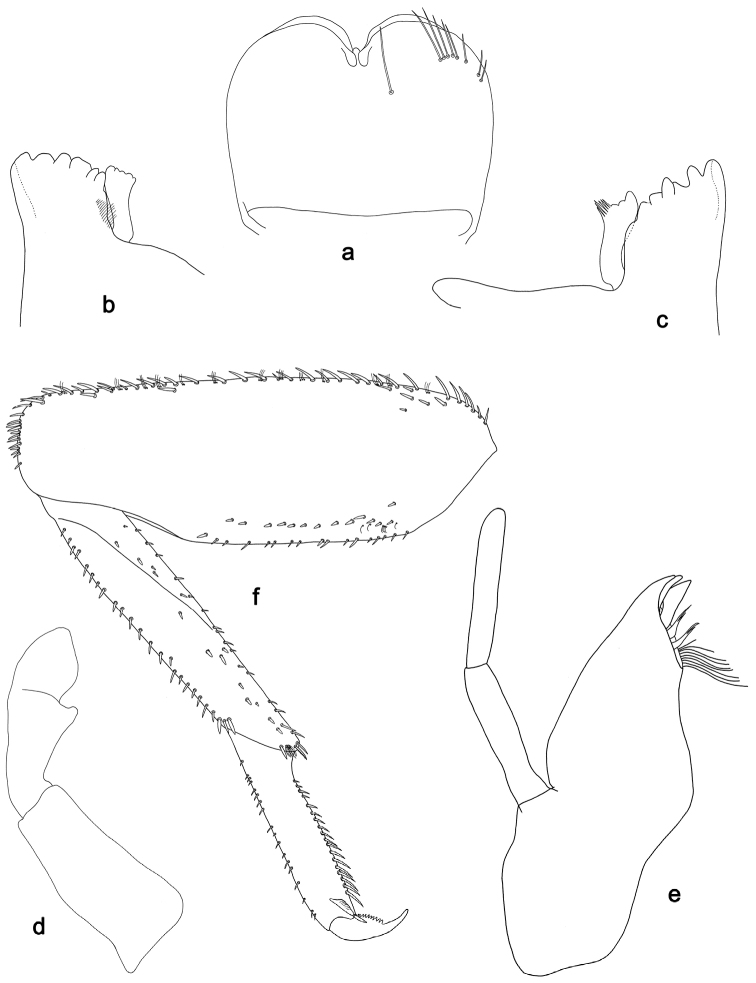
*Labiobaetisvitilis*, larva morphology: **a** Labrum **b** Right mandible **c** Left mandible **d** Labial palp **e** Maxilla **f**Foreleg.

**Larva.** Following combination of characters: A) labrum dorsal arc of setae composed of one plus eight long, simple setae, 5 setae standing closely together; B) right mandible with 3+3 denticles; C) left mandible with 3+3 denticles; D) maxillary palp longer than galea-lacinia, without excavation at inner distolateral margin; E) labial palp segment II with short thumb-like protuberance, segment III slightly pentagonal; F) fore femur rather broad, length ca. 3× as long as maximum width; G) fore leg setation as Fig. 34f.

###### Examined material.

**Paratype.** 1 nymph (on slide, PERC 0 012 576), Papua New Guinea, Western Highlands Prov., Kaugel R, nr Alkena, 07 Sept 1983, J.T. and D.A. Polhemus.

##### 19. Labiobaetis altus sp. n.

Taxon classificationAnimaliaEphemeropteraBaetidae

http://zoobank.org/6765D747-5D2E-4959-964C-E88E837867DF

**Figure 35.**
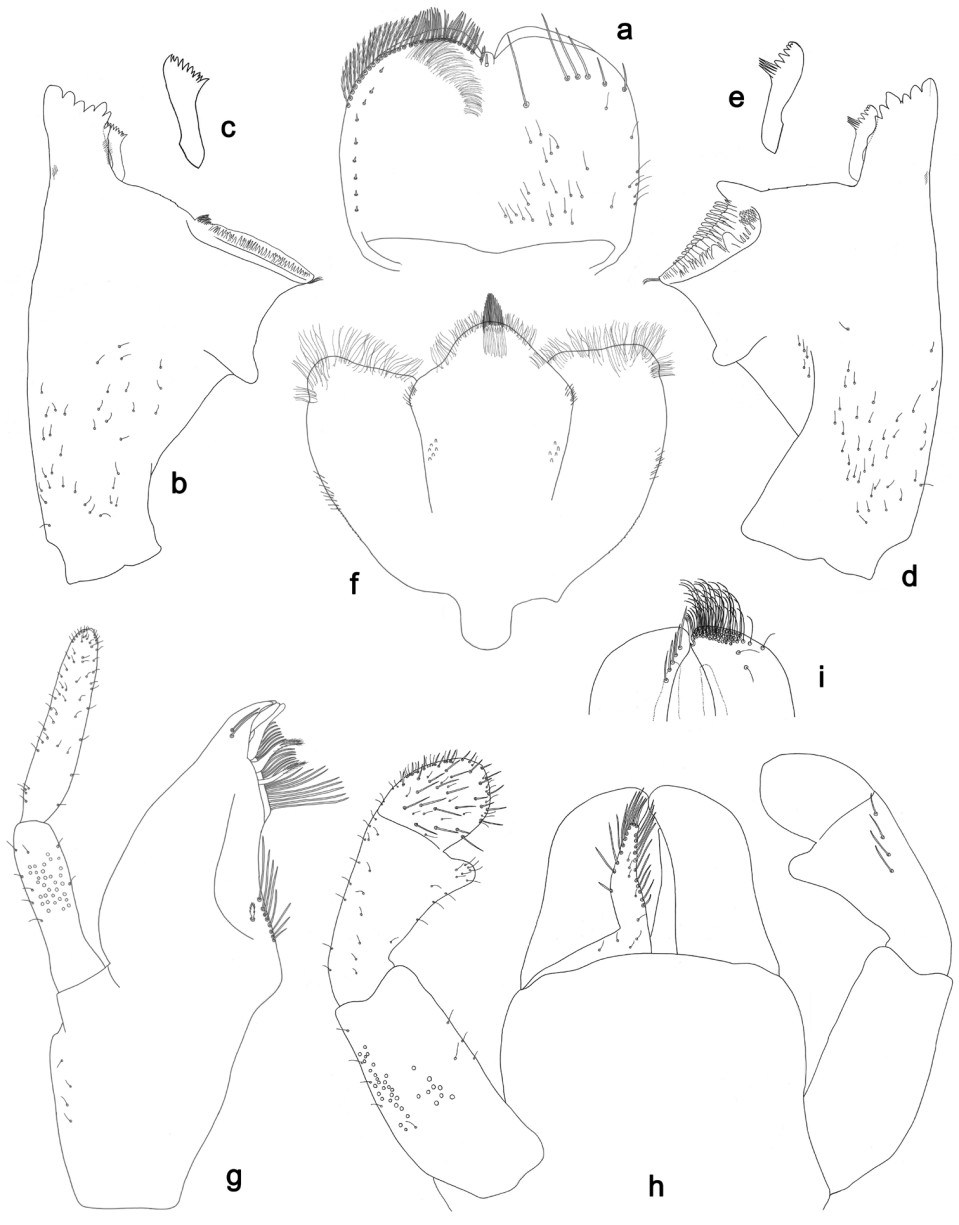
*Labiobaetisaltus* sp. n., larva morphology: **a** Labrum **b** Right mandible **c** Right prostheca **d** Left mandible **e** Left prostheca **f**Hypopharynx**g** Maxilla **h** Labium.

**Figure 36.**
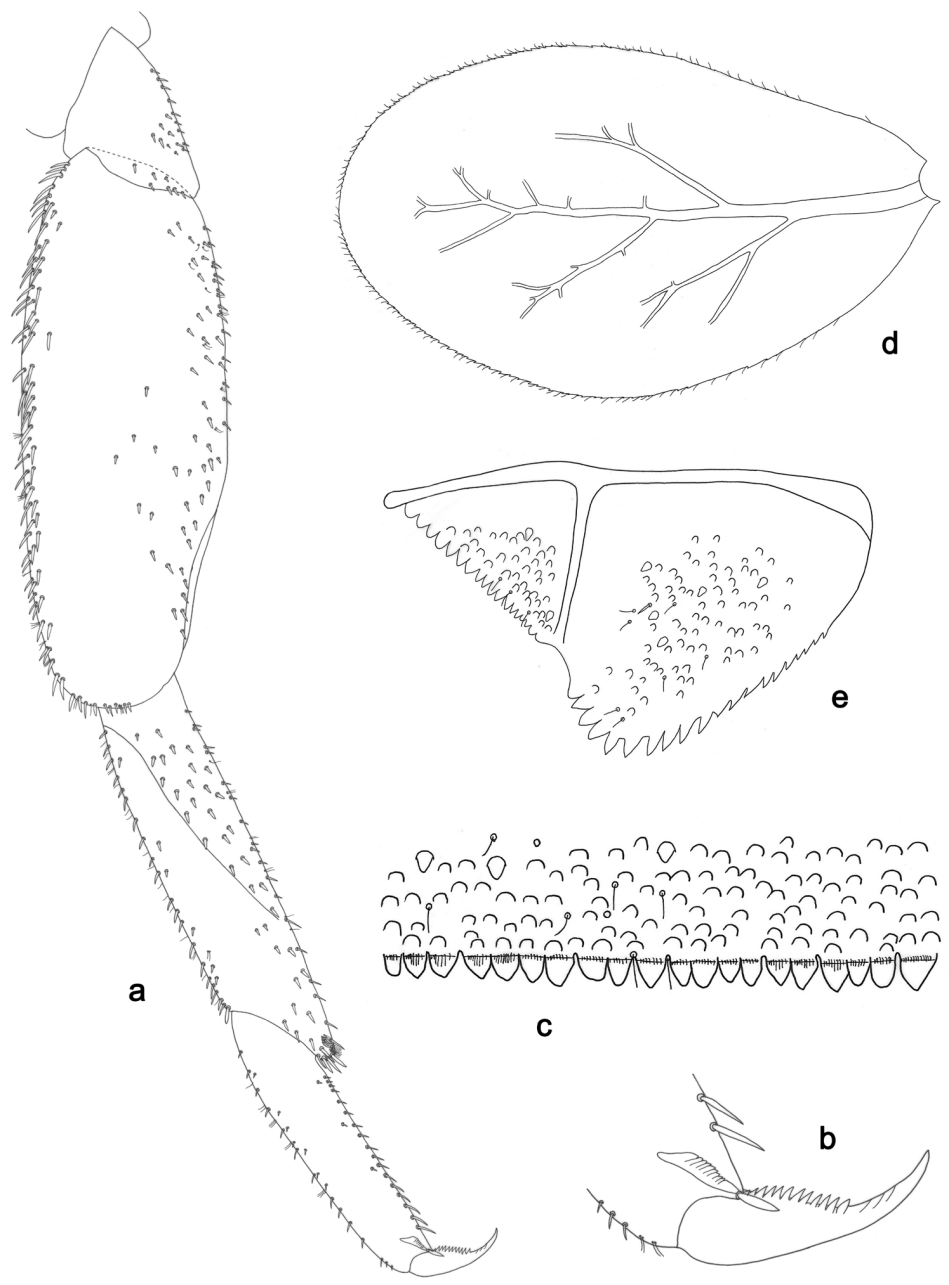
*Labiobaetisaltus* sp. n., larva morphology: **a**Foreleg**b** Fore claw **c**Tergum IV **d** Gill IV **e**Paraproct.

Figures 35 , 36 , 61c , 65b

###### Diagnosis.

**Larva.** Following combination of characters: A) labrum dorsal submarginal arc of setae composed of one plus 5–6 long, simple setae; B) maxillary palp somewhat longer as length of galea-lacinia, apically constricted, without excavation at inner distolateral margin; C) labium paraglossa apically with four rows of long, robust, apically pectinate setae; D) labial palp segment II with a short, thumb-like distomedial protuberance; E) fore femur rather broad, length ca. 3× maximum width, dorsal margin with a row of ca. 30 curved, spine-like setae and many stout, pointed setae near margin; F) fore claw with one row of 12–13 denticles.

###### Description.

**Larva** (Figs 35, 36, 61c). Body length 9.2 mm.

*Colouration.* Head, thorax and abdomen dorsally brown, head and thorax with bright median, dorsal suture, thorax and abdomen with bright pattern as in Fig. 61c, forewing pads with bright striation. Head, thorax and abdomen ventrally colourless, femur with distomedial brown spot, legs otherwise colourless.

*Antenna* with scape and pedicel sub-cylindrical, without distolateral process at scape; flagellum with lanceolate spines and fine, simple setae on apex of each segment.

*Labrum* (Fig. 35a). Rectangular, length 0.7× maximum width. Distal margin with medial emargination and a small process. Dorsally with medium, fine, simple setae scattered over surface; submarginal arc of setae composed of one plus 5–6 long, simple setae. Ventrally with marginal row of setae composed of lateral and anterolateral long, feathered setae and medial long, bifid setae; ventral surface with 9–11 short, spine-like setae near lateral and anterolateral margin.

*Right mandible* (Fig. 35b, c). Incisors fused. Outer and inner sets of denticles with 3 + 4 denticles. Inner margin of innermost denticle with a row of thin setae. Prostheca robust, apically denticulate. Margin between prostheca and mola straight. Tuft of setae at apex of mola present.

*Left mandible* (Fig. 35d, e). Incisors fused. Outer and inner sets of denticles with 3 + 3 denticles and one minute intermediate denticle. Prostheca robust, apically with small denticles and comb-shape structure. Margin between prostheca and mola straight, with minute denticles towards subtriangular process. Subtriangular process long and slender, above level of area between prostheca and mola. Denticles of mola apically constricted. Tuft of setae at apex of mola present.

Both mandibles with lateral margins almost straight. Basal half with fine, simple setae scattered over dorsal surface.

*Hypopharynx* (Fig. 35f). Lingua longer than superlingua. Lingua longer than broad; medial tuft of stout setae present; distal half laterally expanded. Superlingua straight; lateral margin rounded; fine, long, simple setae along distal margin.

*Maxilla* (Fig. 35g). Galea-lacinia with two simple, robust apical setae under crown. Inner dorsal row of setae with three denti-setae, distal denti-seta tooth-like, middle and proximal denti-setae slender, bifid and pectinate. Medially with one bipectinate, spine-like seta and 7–8 long, simple setae. Maxillary palp slightly longer than length of galea-lacinia; two segmented. Palp segment II 1.2× length of segment I. Setae on maxillary palp fine and simple, scattered over surface of segments I and II. Apex of last segment constricted, without excavation at inner distolateral margin.

*Labium* (Fig. 35h, i). Glossa basally broad, narrowing toward apex; shorter than paraglossa; inner margin with 10–11 spine-like setae increasing in length distally; apex with three long, robust setae; outer margin with 6–8 spine-like setae; ventral surface with short, fine, simple, scattered setae. Paraglossa sub-rectangular, curved inward; apex rounded; with four rows of long, apically pectinate setae; dorsally with three medium, simple setae; ventrally with five long, spine-like setae near inner margin. Labial palp with segment I 0.9× length of segments II and III combined. Segment I covered with short, fine, simple setae ventrally and micropores dorsally. Segment II with a short, thumb-like distomedial protuberance; distomedial protuberance 0.5× width of base of segment III; inner margin with short, fine, simple setae, more numerous at apex; outer margin with short, fine, simple setae; dorsally with row of 3–4 long, spine-like, simple setae. Segment III oblong; apex slightly pointed; length 1.2× width; ventrally covered with short and medium spine-like, simple setae and short, fine, simple setae.

*Hind wing pads* absent.

*Foreleg* (Fig. 36a, b). Ratio of foreleg segments 1.6:1.0:0.8:0.3. *Femur*. Length ca. 3× maximum width. Dorsal margin with a row of ca. 30 curved, spine-like setae on margin, and with many stout, pointed setae near margin; length of setae 0.13× maximum width of femur. Apex rounded; with one pair of curved, spine-like setae and some short, stout, pointed setae. Many stout, lanceolate setae and a few fine, simple setae scattered along ventral margin; femoral patch poorly developed. *Tibia.* Dorsal margin with a row of curved, spine-like setae and long, fine, simple setae. Ventral margin with a row of curved, spine-like setae and some longer, stout, pointed setae and a tuft of long, fine, simple setae on apex. Anterior surface scattered with many stout, lanceolate setae. Tibio-patellar suture present on basal 2/3. *Tarsus.* Dorsal margin with a row of short, curved, spine-like setae and long, simple setae. Ventral margin with a row of curved, spine-like setae. Tarsal claw with one row of 12–13 denticles; distally pointed; with 3–4 stripes; subapical setae absent.

*Tergum* (Fig. 36c). Surface with irregular rows of U-shaped scale bases and scattered fine, simple setae and micropores, scales slightly triangular. Posterior margin of tergum IV with rounded or triangular spines, about as long as wide.

*Gills* (Fig. 36d). Present on segments II–VII. Margin with small denticles intercalating long, fine, simple setae. Tracheae extending from main trunk to inner and outer margins. Gill IV as long as length of segments V and VI combined. Gill VII as long as length of segments VIII and 1/2 IX combined.

*Paraproct* (Fig. 36e). Distally not expanded, with ca. 20 marginal, stout spines. Surface with U-shaped scale bases and scattered fine, simple setae. Postero-lateral extension (cercotractor) with small marginal spines.

###### Etymology.

Latin word for high, refers to the altitude of the type locality (2700 m a.s.l.).

###### Distribution.

New Guinea.

###### Biological aspects.

The specimens were collected in altitudes of 2700 m a.s.l. and 2900 m a.s.l.

###### Type-material.

**Holotype.** Nymph (on slide, GBIFCH 00465199), Papua New Guinea, Enga, Kumul Lodge at foot of Mt. Hagen, 2700 m, 05 Dec 2006, 05°47.55'S, 143°58.76'E, Balke & Kinibel (PNG 124). Deposited in ZSM. **Paratypes.** 4 nymphs (1 on slide, GBIFCH 00465200, 3 in alcohol, GBIFCH 00515273, GBIFCH 00508131, deposited in MZL), same data as holotype; 1 nymph (on slide, GBIFCH 00465201, deposited in MZL), Papua New Guinea, Simbu Prov., 05°49'S, 145°04.50'E, Mt. Wilhelm, Pindaunde Creek, 2900 m a.s.l. (in forest), S3 (oria.4), 18 Aug 1999, leg. L. Čížek.

##### 20. Labiobaetis gindroi sp. n.

Taxon classificationAnimaliaEphemeropteraBaetidae

http://zoobank.org/78B8FC52-E792-4A4B-9733-2AC80BACA419

**Figure 37.**
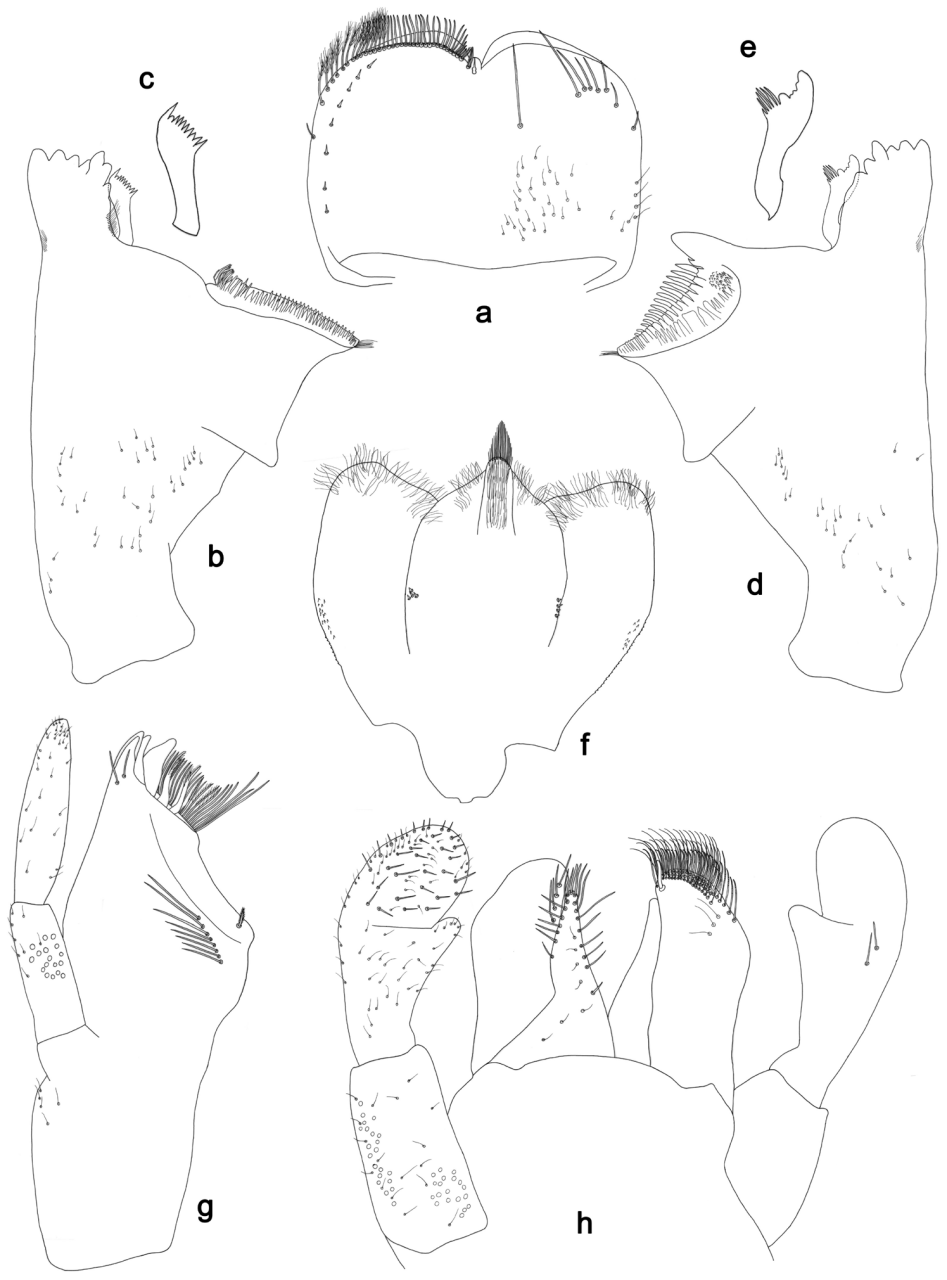
*Labiobaetisgindroi* sp. n., larva morphology: **a** Labrum **b** Right mandible **c** Right prostheca **d** Left mandible **e** Left prostheca **f**Hypopharynx**g** Maxilla **h** Labium.

**Figure 38.**
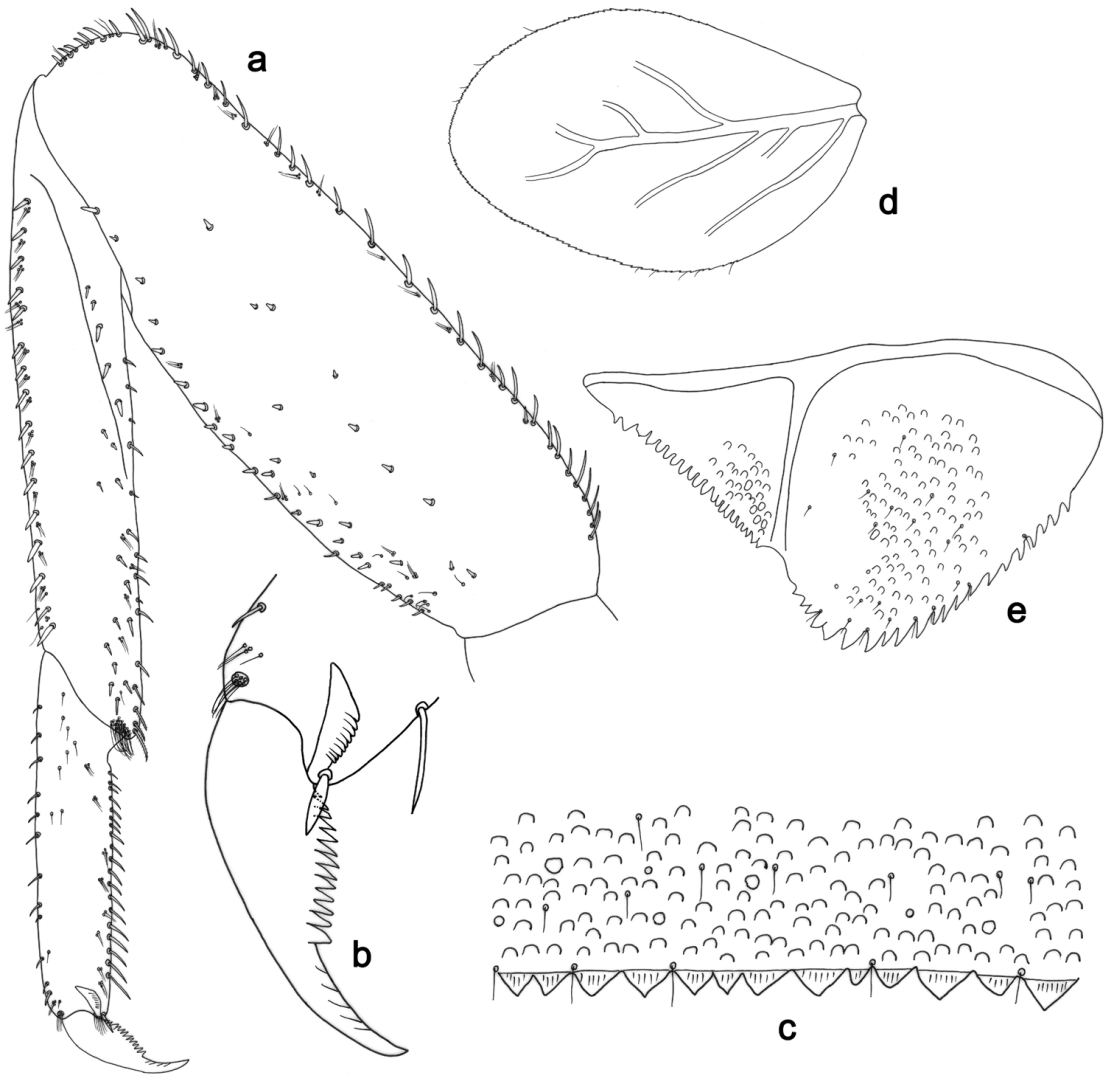
*Labiobaetisgindroi* sp. n., larva morphology: **a**Foreleg**b** Fore claw **c**Tergum IV **d** Gill IV **e**Paraproct.

Figures 37 , 38 , 61d , 65b

###### Diagnosis.

**Larva.** Following combination of characters: A) labrum dorsal arc of submarginal setae composed of one plus 6–7 long simple setae; B) maxillary palp about as long as galea-lacinia, apically slightly pointed and without excavation at inner lateral margin; C) labial palp segment II with an elongated, thumb-like distomedial protuberance; D) fore femur rather broad, length ca. 3× maximum width, dorsal margin with a row of ca. 26 curved, spine-like setae; E) fore claw with 13–15 denticles.

###### Description.

**Larva** (Figs 37, 38, 61d). Body length 6.9 mm; antenna: approximately 2.5× as long as head length.

*Colouration.* Head, thorax and abdomen dorsally brown, head and thorax with bright median, dorsal suture, forewing pads with bright striation, thorax and abdomen with bright pattern as in Fig. 61d. Legs and caudal filaments light brown, femur dorsal margin brown.

*Antenna* with scape and pedicel sub-cylindrical, without distolateral process at scape; flagellum with lanceolate spines and fine, simple setae on apex of each segment.

*Labrum* (Fig. 37a). Rectangular, length 0.7× maximum width. Distal margin with medial emargination and a small process. Dorsally with medium, fine, simple setae scattered over surface; submarginal arc of setae composed of one plus 6–7 long, simple setae. Ventrally with marginal row of setae composed of lateral and anterolateral long, feathered setae and medial long, bifid, pectinate setae; ventral surface with 8–9 short, spine-like setae near lateral and anterolateral margin.

*Right mandible* (Fig. 37b, c). Incisors fused. Outer and inner sets of denticles with 4 + 4 (sometimes three) denticles. Inner margin of innermost denticle with a row of thin setae. Prostheca robust, apically denticulate. Margin between prostheca and mola straight. Tuft of setae at apex of mola present.

*Left mandible* (Fig. 37d, e). Incisors fused. Outer and inner sets of denticles with 3 + 3 (sometimes four) denticles, sometimes with one minute, intermediate denticle. Prostheca robust, apically with small denticles and comb-shape structure. Margin between prostheca and mola straight. Subtriangular process long and slender, above level of area between prostheca and mola. Denticles of mola apically constricted. Tuft of setae at apex of mola present.

Both mandibles with lateral margins almost straight. Basal half with fine, simple setae scattered over dorsal surface.

*Hypopharynx* (Fig. 37f). Lingua about as long as superlingua. Lingua longer than broad; medial tuft of stout setae present; distal half not expanded. Superlingua rounded; lateral margin rounded; fine, long, simple setae along distal margin.

*Maxilla* (Fig. 37g). Galea-lacinia with two simple, robust apical setae under crown. Inner dorsal row of setae with three denti-setae, distal denti-seta tooth-like, middle and proximal denti-setae slender, bifid and pectinate. Medially with one bipectinate, spine-like seta and 8–9 long, simple setae. Maxillary palp about as long as length of galea-lacinia; two segmented. Palp segment II 1.4× length of segment I. Setae on maxillary palp fine and simple, scattered over surface of segments I and II. Apex of last segment slightly pointed, without excavation at inner distolateral margin.

*Labium* (Fig. 37h). Glossa basally broad, narrowing toward apex; shorter than paraglossa; inner margin with 8–9 spine-like setae increasing in length distally; apex with three long, robust, pectinate setae; outer margin with 6–7 spine-like setae increasing in length distally; ventral surface with fine, simple, scattered setae. Paraglossa sub-rectangular, curved inward; apex rounded; with three rows of long, robust, apically pectinate setae; dorsally with row of three medium, simple setae; ventrally with four long, spine-like setae near inner margin. Labial palp with segment I 0.8× length of segments II and III combined. Segment I covered with short and medium, fine, simple setae ventrally and with micropores dorsally. Segment II with an elongated, thumb-like distomedial protuberance; distomedial protuberance 0.4× width of base of segment III; inner and outer margin both with short, fine, simple setae; dorsally with row of 2–5 long, spine-like, simple setae. Segment III oblong; apex rounded; length 1.4× width; ventrally covered with short and medium spine-like, simple setae and short, fine, simple setae.

*Hind wing pads* absent.

*Foreleg* (Fig. 38a, b). Ratio of foreleg segments 1.2:1.0:0.5:0.2. *Femur*. Length ca. 3× maximum width. Dorsal margin with a row of ca. 26 curved, spine-like setae; length of setae 0.16× maximum width of femur. Apex rounded; with one pair of curved, spine-like setae and some short, stout, pointed setae. Many stout, lanceolate setae and a few fine, simple setae scattered along ventral margin; femoral patch poorly developed. *Tibia.* Dorsal margin with a row of stout, lanceolate setae and very fine, simple setae. Ventral margin with a row of curved, spine-like setae and some longer, spine-like, bipectinate setae and a tuft of long, fine, simple setae on apex. Anterior surface scattered with many stout, lanceolate setae and fine, simple setae. Tibio-patellar suture present on basal 1/2. *Tarsus.* Dorsal margin with a row of short, curved, spine-like setae. Ventral margin with a row of curved, spine-like setae. Tarsal claw with one row of 13–15 denticles; distally pointed; with 5–7 stripes; subapical setae absent.

*Tergum* (Fig. 38c). Surface with irregular rows of U-shaped scale bases and scattered fine, simple setae and micropores, scales short, apically rounded. Posterior margin of tergum IV with triangular spines, wider than long.

*Gills* (Fig. 38d). Present on segments II - VII. Margin with small denticles intercalating long, fine, simple setae. Tracheae partly extending from main trunk towards outer and inner margins. Gill IV as long as length of segments V and VI combined. Gill VII as long as length of segments VIII, IX and 1/4 X combined.

*Paraproct* (Fig. 38e). Distally not expanded, with ca. 21 marginal, stout spines. Surface with U-shaped scale bases and scattered fine, simple setae and micropores. Postero-lateral extension (cercotractor) with small marginal spines.

###### Etymology.

Dedicated to the late friend of one of the authors (JLG), the biologist Cédric Gindro.

###### Distribution.

New Guinea.

###### Biological aspects.

The specimens were collected in altitudes of 1620 m a.s.l. and 2000 m a.s.l.

###### Type-material.

**Holotype.** Nymph (on slide, GBIFCH 00465202), Indonesia, Papua, Wamena, 20 mins towd Jiwika, limestone creek, 1620 m, 18 Oct 2011, 03°56.95'S, 138°54.38'E, Balke (PAP07). Temporary deposited in MZL before definitely housed in MZB. **Paratypes.** 63 nymphs (3 on slides, GBIFCH 00465203, GBIFCH 00465204, GBIFCH 00465205, 42 in alcohol, GBIFCH 00515254, GBIFCH 00515256, deposited in MZL; 18 in alcohol, GBIFCH 00515255, GBIFCH00515257, deposited in ZSM), same data as holotype.

###### Additional material.

5 nymphs (1 on slide, GBIFCH 00465206, 4 in alcohol, GBIFCH 00515291, deposited in MZL), Papua New Guinea, Western Highlands, Simbai, 2000 m, 28 Feb 2007, 05°15.17'S, 144°32.81'E, Kinibel (PNG 136).

##### 21. Labiobaetis paravitilis sp. n.

Taxon classificationAnimaliaEphemeropteraBaetidae

http://zoobank.org/1C21C5E7-497F-4B35-9D10-CD119B22DE01

**Figure 39.**
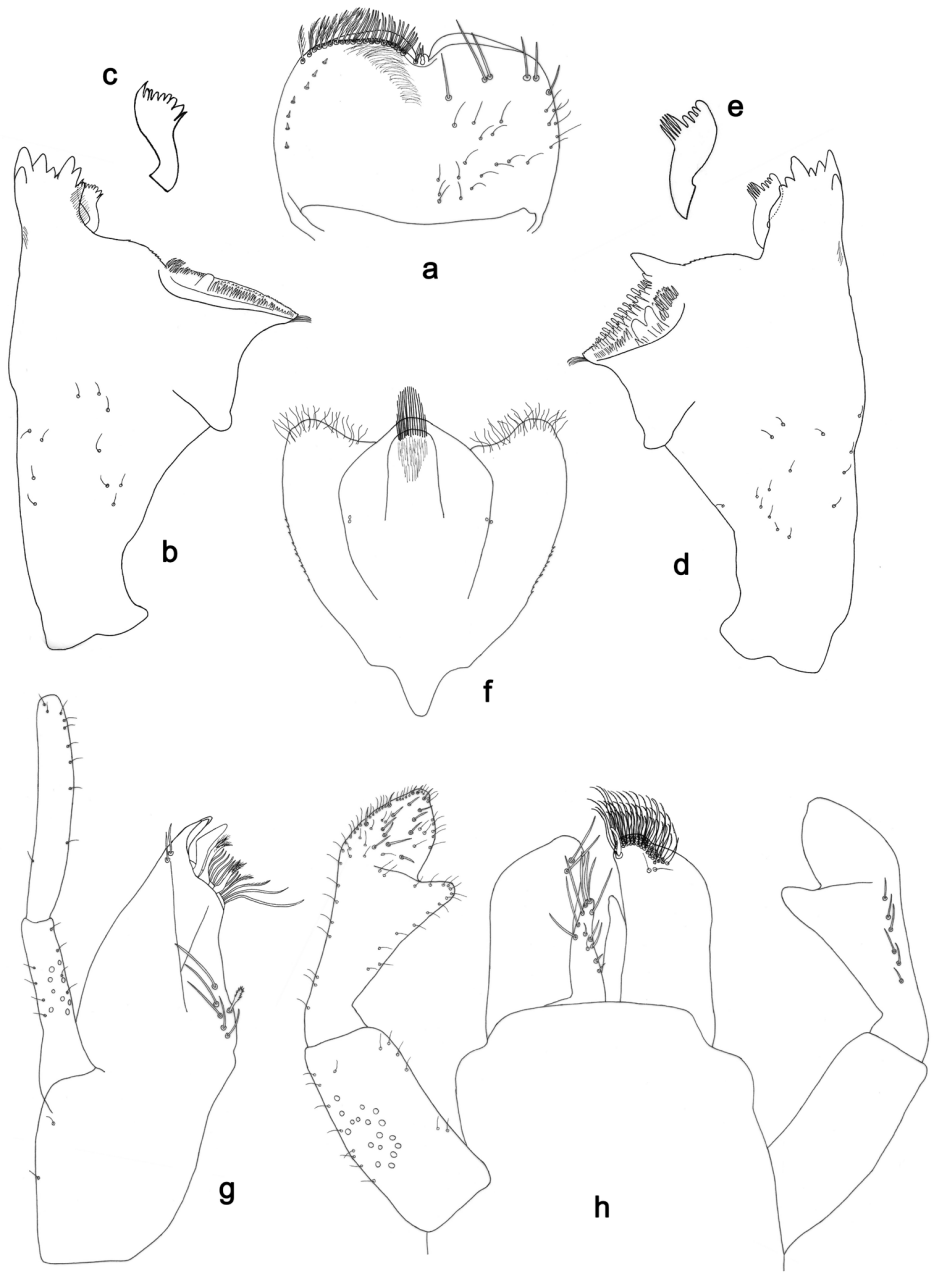
*Labiobaetisparavitilis* sp. n., larva morphology: **a** Labrum **b** Right mandible **c** Right prostheca **d** Left mandible **e** Left prostheca **f**Hypopharynx**g** Maxilla **h** Labium.

**Figure 40.**
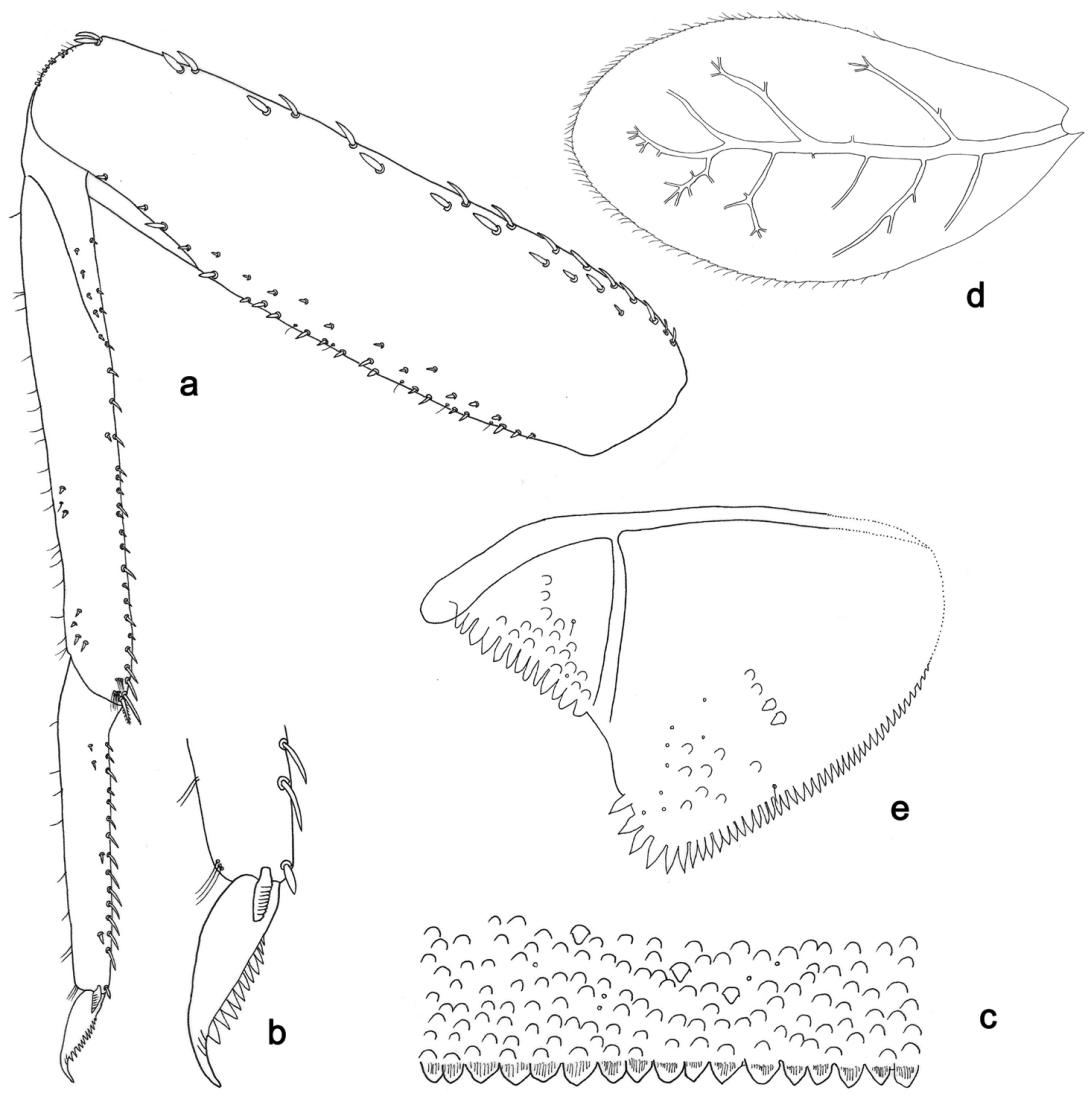
*Labiobaetisparavitilis* sp. n., larva morphology: **a**Foreleg**b** Fore claw **c**Tergum IV **d** Gill IV **e**Paraproct.

Figures 39 , 40 , 62a , 65b

###### Diagnosis.

**Larva.** Following combination of characters: A) labrum dorsal submarginal arc of setae composed of one plus 5–6 long, simple setae; B) maxillary palp longer as length of galea-lacinia, apically rounded, without excavation at inner distolateral margin; C) labial glossae much shorter than paraglossae; D) labial palp segment II with an elongated, thumb-like distomedial protuberance, segment III conical, apically slightly truncate; E) fore femur slender, length 3.6× maximum width, dorsal margin with a row of ca. 12 curved, spine-like setae; F) fore claw with one row of eleven denticles.

###### Description.

**Larva** (Figs 39, 40, 62a). Body length 3.7 mm.

*Colouration.* Head, thorax and abdomen dorsally brown, head and thorax with bright median, dorsal suture. Head, thorax and abdomen ventrally light brown, femur dorsal margin light brown, legs otherwise colourless, caudal filaments colourless.

*Antenna* with scape and pedicel sub-cylindrical, without distolateral process at scape; flagellum with lanceolate spines and fine, simple setae on apex of each segment.

*Labrum* (Fig. 39a). Rectangular, length 0.7× maximum width. Distal margin with medial emargination and a small process. Dorsally with medium, fine, simple setae scattered over surface; submarginal arc of setae composed of one plus 5–6 long, simple setae. Ventrally with marginal row of setae composed of lateral and anterolateral long, feathered setae and medial long, bifid setae; ventral surface with seven short, spine-like setae near lateral and anterolateral margin.

*Right mandible* (Fig. 39b, c). Incisors fused. Outer and inner sets of denticles with 4 + 3 denticles, partly plus one small intermediate denticle. Inner margin of innermost denticle with a row of thin setae. Prostheca robust, apically denticulate. Margin between prostheca and mola slightly convex, with minute denticles. Tuft of setae at apex of mola present.

*Left mandible* (Fig. 39d, e). Incisors fused. Outer and inner sets of denticles with 4 + 3 denticles. Prostheca robust, apically with small denticles and comb-shape structure. Margin between prostheca and mola slightly convex, with minute denticles toward subtriangular process. Subtriangular process long and slender, above level of area between prostheca and mola. Denticles of mola apically constricted. Tuft of setae at apex of mola present.

Both mandibles with lateral margins almost straight. Basal half with fine, simple setae scattered over dorsal surface.

*Hypopharynx* (Fig. 39f). Lingua about as long as superlingua. Lingua longer than broad; medial tuft of stout setae present; distal half laterally expanded. Superlingua rounded; lateral margin rounded; fine, long, simple setae along distal margin.

*Maxilla* (Fig. 39g). Galea-lacinia with two simple, robust apical setae under crown. Inner dorsal row of setae with three denti-setae, distal denti-seta tooth-like, middle and proximal denti-setae slender, bifid and pectinate. Medially with one bipectinate, spine-like seta and five long, simple setae. Maxillary palp 1.4× as long as length of galea-lacinia; two segmented. Palp segment II 1.4× length of segment I. Setae on maxillary palp fine and simple, scattered over surface of segments I and II. Apex of last segment rounded, without excavation at inner distolateral margin.

*Labium* (Fig. 39h). Glossa basally broad, narrowing toward apex; much shorter than paraglossa; inner margin with five spine-like setae increasing in length distally; apex with two long, robust setae; outer margin with 3–4 long, spine-like setae; ventral surface with few short, fine, simple setae. Paraglossa sub-rectangular, curved inward; apex rounded; with three rows of long, robust, apically pectinate setae; dorsally with 2–3 medium, simple setae; ventrally with two long, spine-like setae near inner margin. Labial palp with segment I 0.8× length of segments II and III combined. Segment I covered with short, fine, simple setae ventrally and micropores dorsally. Segment II with an elongated, thumb-like distomedial protuberance; distomedial protuberance 0.5× width of base of segment III; inner and outer margin both with short, fine, simple setae; dorsally with row of six medium, spine-like, simple setae. Segment III conical; apex truncate; length 0.9× width; ventrally covered with short and medium spine-like, simple setae and short, fine, simple setae.

*Hind wing pads* absent.

*Foreleg* (Fig. 40a, b). Ratio of foreleg segments 1.2:1.0:0.5:0.2. *Femur*. Length ca. 4× maximum width. Dorsal margin with a row of ca. 12 curved, spine-like setae; length of setae 0.16× maximum width of femur. Apex rounded; with one pair of curved, spine-like setae and some very short, stout setae. Many stout, lanceolate setae and a few fine, simple setae scattered along ventral margin; femoral patch absent. *Tibia.* Dorsal margin with a row of fine, simple setae. Ventral margin with a row of curved, spine-like setae, one seta on apex much longer; one long, bipectinate seta and a tuft of fine, long, simple setae on apex. Anterior surface scattered with stout, lanceolate setae. Tibio-patellar suture present on basal 1/3. *Tarsus.* Dorsal margin with row of fine, simple setae. Ventral margin with a row of curved, spine-like setae. Tarsal claw with one row of eleven denticles; tapering distally; with three stripes; subapical setae absent.

*Tergum* (Fig. 40c). Surface with irregular rows of U-shaped scale bases and scattered micropores, scales slightly triangular. Posterior margin of tergum IV with rounded or triangular spines, wider than long.

*Gills* (Fig. 40d). Present on segments II–VII. Margin with small denticles intercalating long, fine, simple setae. Tracheae extending from main trunk to inner and outer margins. Gill IV as long as length of segments V and VI combined. Gill VII as long as length of segments VIII and IX combined.

*Paraproct* (Fig. 40e). Distally not expanded, with many marginal, stout spines. Surface with U-shaped scale bases and scattered fine, simple setae and micropores. Postero-lateral extension (cercotractor) with small marginal spines.

###### Etymology.

Refers to the morphological similarity with *L.vitilis*.

###### Distribution.

New Guinea.

###### Biological aspects.

The specimens were collected at an altitude of 30 m a.s.l.

###### Type-material.

**Holotype.** Nymph (on slide, GBIFCH 00465207), Papua New Guinea, Madang, Trans Gogol, 30 m, 02.2008, 05°18.09'S, 145°36.45'E, BRC leg. (PNG 179). Deposited in ZSM. **Paratypes.** 17 nymphs (1 on slide, GBIFCH 00465208, 11 in alcohol, GBIFCH 00515271, GBIFCH 00508148, deposited in MZL; 5 in alcohol, GBIFCH00515272, deposited in ZSM), same data as holotype.

##### 22. Labiobaetis wilhelmensis sp. n.

Taxon classificationAnimaliaEphemeropteraBaetidae

http://zoobank.org/D6A28AD5-23FA-42D7-9407-22215C86A0C9

**Figure 41.**
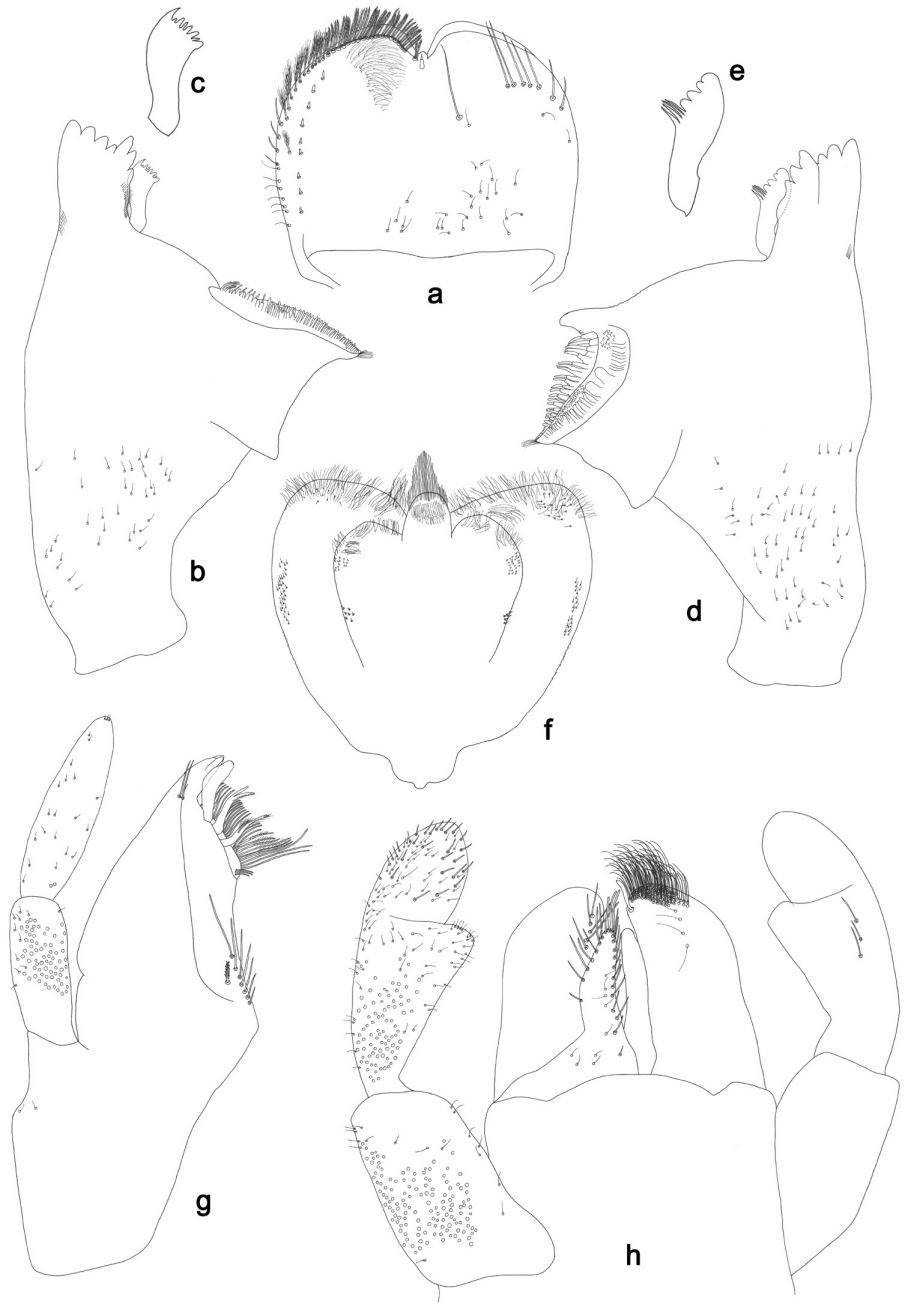
*Labiobaetiswilhelmensis* sp. n., larva morphology: **a** Labrum **b** Right mandible **c** Right prostheca **d** Left mandible **e** Left prostheca **f**Hypopharynx**g** Maxilla **h** Labium.

**Figure 42.**
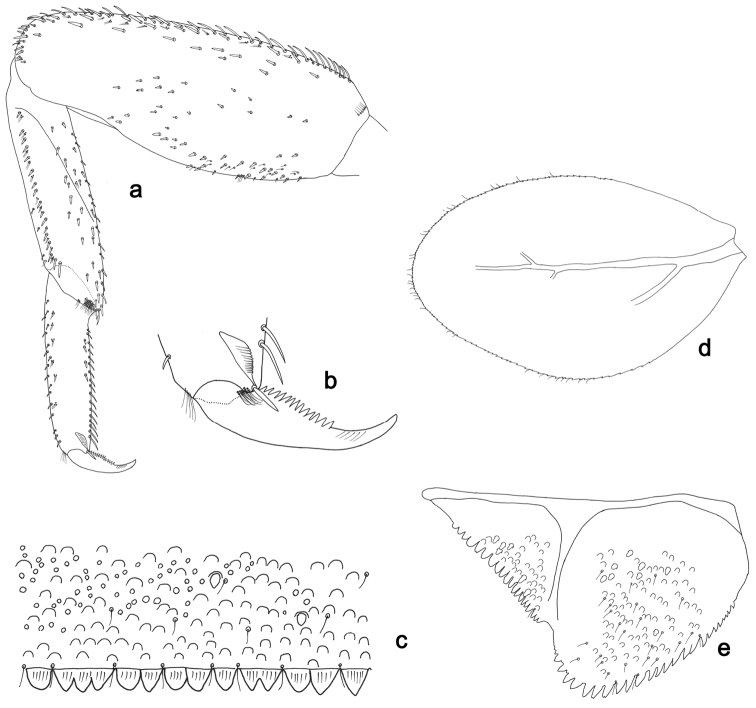
*Labiobaetiswilhelmensis* sp. n., larva morphology: **a**Foreleg**b** Fore claw **c**Tergum IV **d** Gill IV **e**Paraproct.

Figures 41 , 42 , 62b , 65b

###### Diagnosis.

**Larva.** Following combination of characters: A) labrum dorsal submarginal arc of setae composed of one plus 7–10 long, simple setae; B) maxillary palp somewhat longer than length of galea-lacinia, segment II apically slightly pointed and without excavation at inner lateral margin; C) labial palp segment III oblong, apically slightly pointed; D) labium paraglossa apically with five rows of long, robust, apically pectinate setae; E) fore femur rather broad, length ca. 3× maximum width, dorsal margin with a row of ca. 29 curved, spine-like setae and many stout, pointed setae near margin; F) fore claw with one row of 13–15 denticles; G) tracheae of gills restricted to main trunk.

###### Description.

**Larva** (Figs 41, 42, 62b). Body length 6 mm; antenna approximately twice as long as head length.

*Colouration.* Head, thorax and abdomen dorsally brown, head and thorax with bright median, dorsal suture. Head, thorax and abdomen ventrally light brown, legs colourless with light brown pattern as in Fig. 62b, caudal filaments light brown.

*Antenna* with scape and pedicel sub-cylindrical, without distolateral process at scape; flagellum with lanceolate spines and fine, simple setae on apex of each segment.

*Labrum* (Fig. 41a). Rectangular, length 0.8× maximum width. Distal margin with medial emargination and small process. Dorsally with medium, fine, simple setae scattered over surface; submarginal arc of setae composed of one plus 7–10 long, simple setae. Ventrally with marginal row of setae composed of lateral and anterolateral long, feathered setae and medial long, bifid, pectinate setae; ventral surface with nine short, spine-like setae near lateral and anterolateral margin.

*Right mandible* (Fig. 41b, c). Incisors fused. Outer and inner sets of denticles with 4 + 4 (sometimes three) denticles. Inner margin of innermost denticle with a row of thin setae. Prostheca robust, apically denticulate. Margin between prostheca and mola straight. Tuft of setae at apex of mola present.

*Left mandible* (Fig. 41d, e). Incisors fused. Outer and inner sets of denticles with 3 + 3 denticles and one minute intermediate denticle. Prostheca robust, apically with small denticles and comb-shape structure. Margin between prostheca and mola straight. Subtriangular process long and slender, at the same level as area between prostheca and mola. Denticles of mola apically constricted. Tuft of setae at apex of mola present.

Both mandibles with lateral margins almost straight. Basal half with fine, simple setae scattered over dorsal surface.

*Hypopharynx* (Fig. 41f). Lingua shorter than superlingua. Lingua broader than long; medial tuft of stout setae present; distal half laterally expanded. Superlingua rounded; lateral margin rounded; fine, long, simple setae along distal margin.

*Maxilla* (Fig. 41g). Galea-lacinia with two simple, robust apical setae under crown. Inner dorsal row of setae with three denti-setae, distal denti-seta tooth-like, middle and proximal denti-setae slender, bifid and pectinate. Medially with one bipectinate, spine-like seta and 7–8 long, simple setae. Maxillary palp slightly longer than length of galea-lacinia; two segmented. Palp segment II 1.3× length of segment I. Setae on maxillary palp fine and simple, scattered over surface of segments I and II. Apex of last segment slightly pointed, without excavation at inner distolateral margin.

*Labium* (Fig. 41h). Glossa basally broad, narrowing toward apex; shorter than paraglossa; inner margin with eleven spine-like setae; apex with three long, robust, pectinate setae; outer margin with 7–8 long, spine-like setae; ventral surface with short, fine, simple, scattered setae. Paraglossa sub-rectangular, curved inward; apex rounded; with five rows of long, robust, apically pectinate setae; dorsally with row of four medium, simple setae; ventrally with five long, spine-like setae near inner margin. Labial palp with segment I 0.8× length of segments II and III combined. Segment I covered with short, fine, simple setae ventrally and micropores dorsally. Segment II with a short, thumb-like distomedial protuberance; distomedial protuberance 0.4× width of base of segment III; inner and outer margin both with short, fine, simple setae; dorsally with row of three long, spine-like setae. Segment III oblong; apex rounded; length 1.4× width; ventrally covered with short and medium spine-like, simple setae and short, fine, simple setae.

*Hind wing pads* absent.

*Foreleg* (Fig. 42a, b). Ratio of foreleg segments 1.5:1.0:0.6:0.3. *Fore femur*. Length 2.6× maximum width. Dorsal margin with a row of ca. 29 curved, spine-like setae and with many stout, pointed setae near margin; length of setae 0.14× maximum width of femur. Apex rounded; with one pair of curved, spine-like setae and many short, stout, pointed setae. Many stout, lanceolate setae and a few fine, simple setae along ventral margin; femoral patch poorly developed. *Tibia.* Dorsal margin with a row of stout, lanceolate setae and very fine, simple setae. Ventral margin with a row of curved, spine-like setae and some longer, stout, pointed setae and a tuft of long, fine, simple setae on apex. Anterior surface scattered with many stout, lanceolate setae. Tibio-patellar suture present on basal 2/3. *Tarsus.* Dorsal margin with a row of short, curved, spine-like setae. Ventral margin with a row of curved, spine-like setae and some stout, pointed setae near margin. Tarsal claw with one row of 13–15 denticles; distally pointed; with five stripes; subapical setae absent.

*Tergum* (Fig. 42c). Surface with irregular rows of U-shaped scale bases and scattered fine, simple setae, micropores and egg-shaped scales. Posterior margin of tergum IV with rounded or triangular spines, about as long as wide.

*Gills* (Fig. 42d). Present on segments II–VII. Margin with small denticles intercalating long, fine, simple setae. Tracheae restricted to main trunk. Gill IV as long as length of segments V and 1/2 VI combined. Gill VII as long as length of segments VIII and 1/3 IX combined.

*Paraproct* (Fig. 42e). Distally not expanded, with ca. 26 marginal, stout spines. Surface with U-shaped scale bases and scattered fine, simple setae. Postero-lateral extension (cercotractor) with small marginal spines.

###### Etymology.

Refers to the type locality at Mt. Wilhelm.

###### Distribution.

New Guinea.

###### Biological aspects.

The specimens were collected in altitudes of 2900 m a.s.l. and 3210 m a.s.l.

###### Type-material.

**Holotype.** Nymph (on slide, GBIFCH 00465209), Papua New Guinea, Simbu Prov., 05°48.050'S, 145°04.15'E, (GPS)’, Mt. Wilhelm, Pindaunde Creek, 3210 m a.s.l., (10895 ft GPS), S32 (oria.3), 17 Aug 1999, L. Čížek leg. Deposited in MZL. **Paratypes.** 14 nymphs (10 in alcohol, GBIFCH 00515247, deposited in MZL; 4 in alcohol, GBIFCH 00515248, deposited in ZSM), same data as holotype; 108 nymphs (3 on slides, GBIFCH 00465210, GBIFCH 00465211, GBIFCH 00465212, 61 in alcohol, GBIFCH 00515249, GBIFCH 00508127, deposited in MZL; 44 in alcohol, GBIFCH 00515250, deposited in ZSM), Papua New Guinea, Simbu Prov., 05°49’ 145°04.5’, Mt. Wilhelm, Pindaunde Creek, 2900 m a.s.l., (in forest), S3 (oria.4), 18 Aug 1999, leg. L. Čížek .

#### *L.vultuosus* group of species

The group is characterised by a hook-like protuberance of labial palp segment II and the labrum with a dorsal, submarginal arc of setae composed of simple setae.

##### 23. Labiobaetis vultuosus

Taxon classificationAnimaliaEphemeropteraBaetidae

(Lugo-Ortiz & McCafferty, 1999)

Figures 43 , 64b

###### Diagnosis.

**Figure 43.**
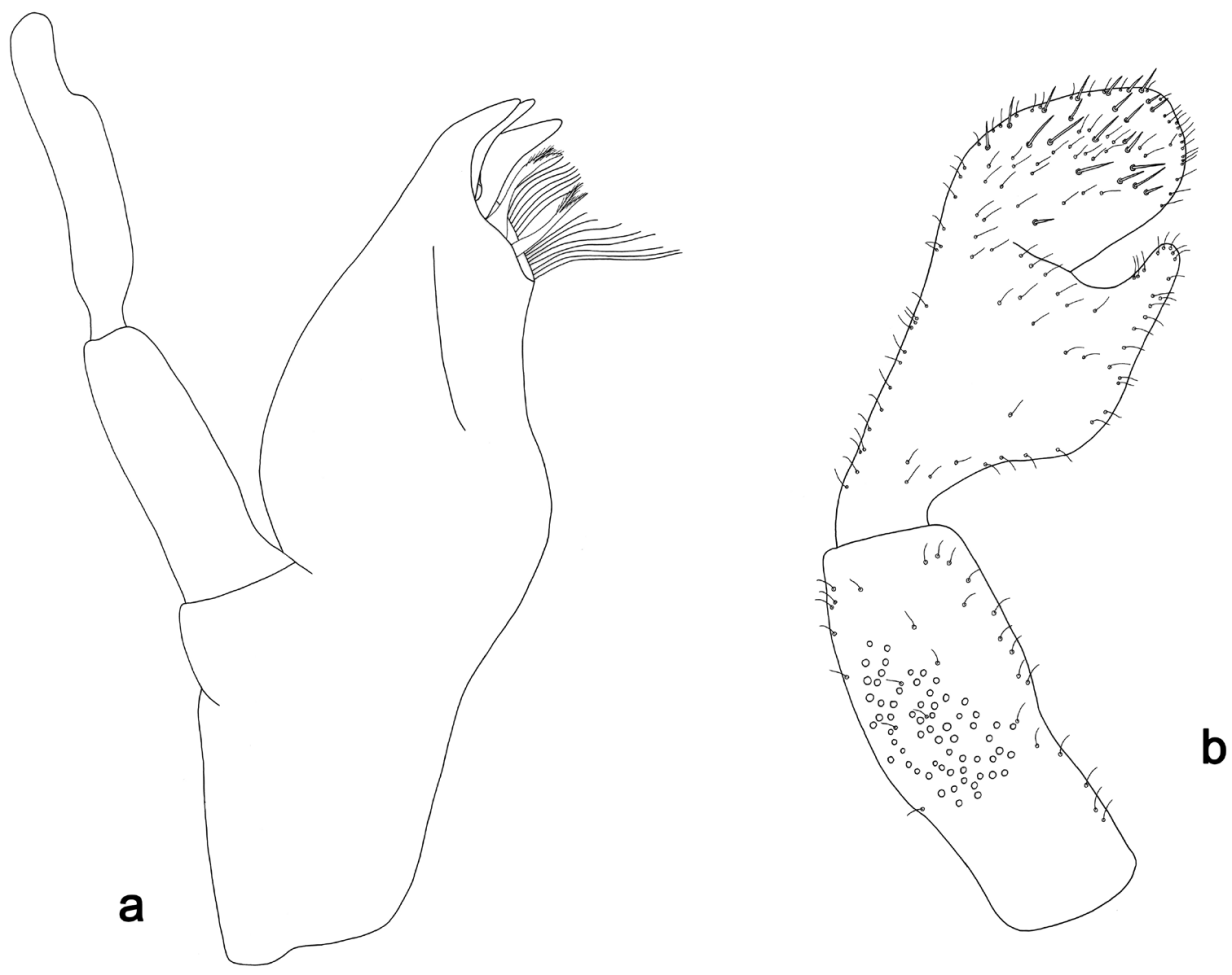
*Labiobaetisvultuosus*, larva morphology: **a** Maxilla **b** Labial palp.

**Larva.** Following combination of characters: A) labrum dorsal submarginal arc of setae composed of one plus 8–9 long, simple setae; B) maxillary palp longer than galea-lacinia, with well-developed excavation at inner distolateral margin; C) labial palp segment II with hook-like distomedial protuberance, as Fig. 43b, segment III conical; D) fore claw with a row of 8–10 denticles.

###### Examined material.

**Paratype.** 1 nymph (on slide, PERC 0 012 577), Papua New Guinea, Western Highlands Prov., 17 km N of Mt. Hagen, 06 Sept 1983, J.T. and D.A. Polhemus leg.

##### 24. Labiobaetis paravultuosus sp. n.

Taxon classificationAnimaliaEphemeropteraBaetidae

http://zoobank.org/0E6E506F-6BDD-4119-9FDF-D58B955054E7

**Figure 44.**
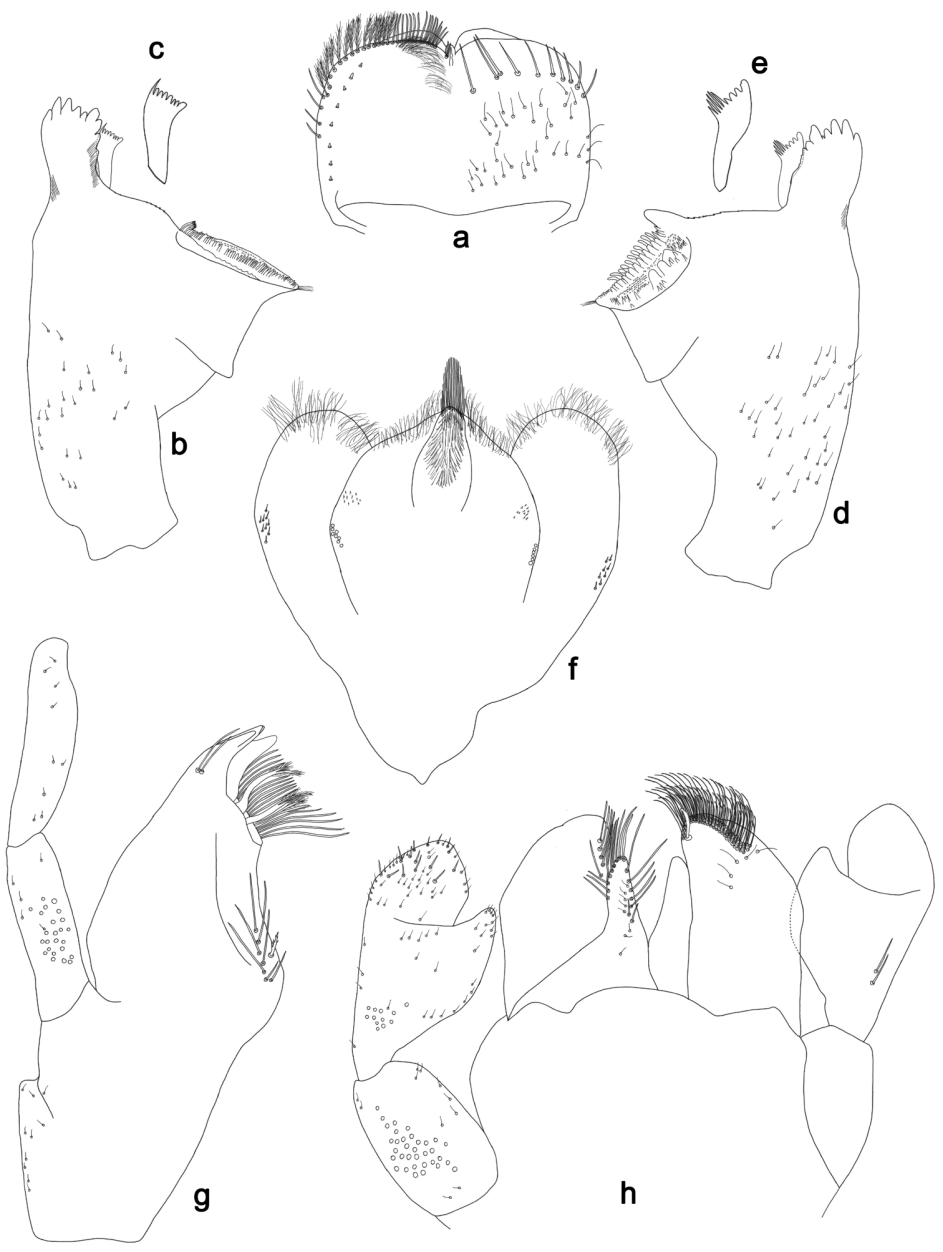
*Labiobaetisparavultuosus* sp. n., larva morphology: **a** Labrum **b** Right mandible **c** Right prostheca **d** Left mandible **e** Left prostheca **f**Hypopharynx**g** Maxilla **h** Labium.

**Figure 45.**
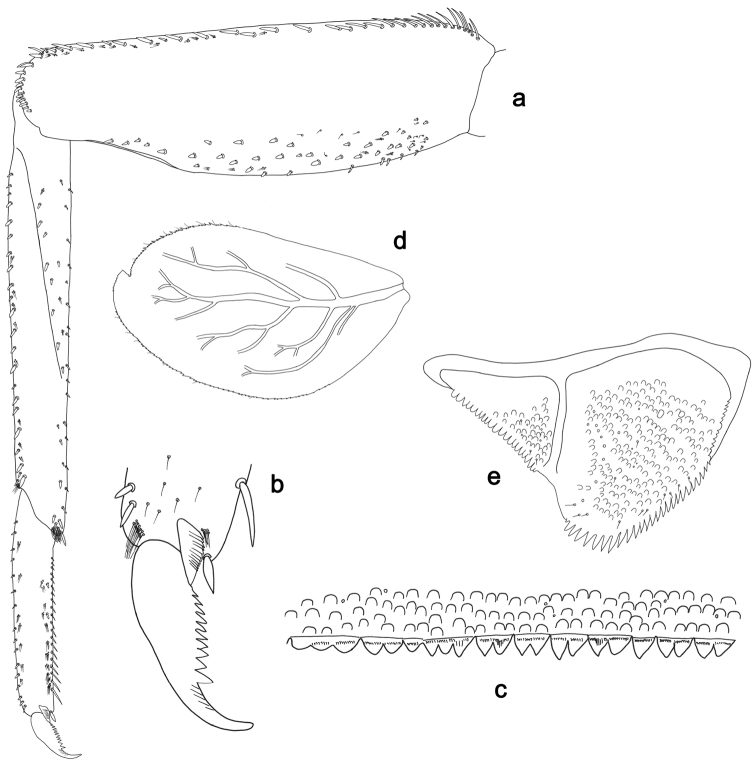
*Labiobaetisparavultuosus* sp. n., larva morphology: **a**Foreleg**b** Fore claw **c**Tergum IV **d** Gill IV **e**Paraproct.

Figures 44 , 45 , 62c , 64b

###### Diagnosis.

**Larva.** Following combination of characters: A) labrum dorsal submarginal arc of setae composed of one plus nine long, simple setae; B) maxillary palp with segment II longer than length of segment I, segment II with excavation at inner distolateral margin; C) labial palp segment II with a hook-like distomedial protuberance; D) fore femur rather broad, length ca. 3× maximum width, dorsal margin with a row of ca. 28 curved, spine-like setae; E) fore claw with 10 denticles; F) gills margin serrated with alternating bigger and smaller spines.

###### Description.

**Larva** (Figs 44, 45, 62c). Body length 7.8 mm; antenna approximately twice as long as head length.

*Colouration.* Head, thorax and abdomen dorsally brown, head and thorax with bright median, dorsal suture, forewing pads with bright striation. Head, thorax and abdomen ventrally light brown, femur colourless with a medial brown spot and a brown dorsal margin, tibia and tarsus brown, caudal filaments brown.

*Antenna* with scape and pedicel sub-cylindrical, without distolateral process at scape; flagellum with lanceolate spines and fine, simple setae on apex of each segment.

*Labrum* (Fig. 44a). Rectangular, length 0.7× maximum width. Distal margin with medial emargination and a small process. Dorsally with many medium to long, fine, simple setae; submarginal arc of setae composed of one plus nine long, simple setae. Lateral margin with few long, spine-like setae and few long, fine, simple setae. Ventrally with marginal row of setae composed of lateral and anterolateral long, feathered setae and medial long, bifid, pectinate setae; ventral surface with eight short, spine-like setae near lateral and anterolateral margin.

*Right mandible* (Fig. 44b, c). Incisors fused. Outer and inner sets of denticles with 5 + 4 denticles. Inner margin of innermost denticle with a row of thin setae. Prostheca robust, apically denticulate. Margin between prostheca and mola slightly convex, with minute denticles. Tuft of setae at apex of mola present.

*Left mandible* (Fig. 44d, e). Incisors fused. Outer and inner sets of denticles with 4 + 3 denticles and one minute intermediate denticle. Prostheca robust, apically with small denticles and comb-shape structure. Margin between prostheca and mola straight, with minute denticles towards subtriangular process. Subtriangular process long and slender, above level of area between prostheca and mola. Denticles of mola apically constricted. Tuft of setae at apex of mola present.

Both mandibles with lateral margins slightly convex. Basal half with fine, simple setae scattered over dorsal surface.

*Hypopharynx* (Fig. 44f). Lingua about as long as superlingua. Lingua about as broad as long; medial tuft of stout setae present; distal half laterally expanded. Superlingua rounded; lateral margin rounded; fine, long, simple setae along distal margin.

*Maxilla* (Fig. 44g). Galea-lacinia with two simple, robust apical setae under crown. Inner dorsal row of setae with three denti-setae, distal denti-seta tooth-like, middle and proximal denti-setae slender, bifid and pectinate. Medially with one bipectinate, spine-like seta and 6–7 long, simple setae. Maxillary palp 1.2× as long as length of galea-lacinia; two segmented. Palp segment II 1.2× length of segment I. Setae on maxillary palp fine and simple, scattered over surface of segments I and II. Apex of last segment rounded, with excavation at inner distolateral margin.

*Labium* (Fig. 44h). Glossa basally broad, narrowing toward apex; shorter than paraglossa; inner margin with 7–9 spine-like setae increasing in length distally; apex with three long, robust, pectinate setae; outer margin with six long spine-like setae increasing in length distally; ventral surface with short, fine, simple, scattered setae. Paraglossa sub-rectangular, curved inward; apex rounded; with three rows of long, robust, apically pectinate setae; dorsally with five medium, simple setae; ventrally with five long, spine-like setae near inner margin. Labial palp with segment I 0.7× length of segments II and III combined. Segment I covered with short, fine, simple setae ventrally and micropores dorsally. Segment II with a hook-like distomedial protuberance; distomedial protuberance 0.5× width of base of segment III; inner and outer margin both with short, fine, simple setae; dorsally with row of 2–3 long, spine-like, simple setae. Segment III slightly pentagonal; apex rounded; length 1.0× width; ventrally covered with short and medium spine-like, simple setae and short, fine, simple setae.

*Hind wing pads* absent.

*Foreleg* (Fig. 45a, b). Ratio of foreleg segments 1.1:1.0:0.5:0.1. *Femur*. Length ca. 3× maximum width. Dorsal margin with a row of ca. 28 curved, spine-like setae; length of setae 0.16× maximum width of femur. Apex rounded; with two pairs of curved, spine-like setae and many short, stout, pointed setae. Many stout, lanceolate setae and a few fine, simple setae scattered along ventral margin; femoral patch poorly developed. *Tibia.* Dorsal margin with a row of curved, spine-like setae and long, fine, simple setae. Ventral margin with a row of short, curved, spine-like setae and some longer, spine-like, bipectinate setae and a tuft of long, fine, simple setae on apex. Anterior surface scattered with stout, lanceolate setae. Tibio-patellar suture present on basal 2/3. *Tarsus.* Dorsal margin with a row of short, curved, spine-like setae and long, simple setae. Ventral margin with a row of curved, spine-like setae. Tarsal claw with one row of ten denticles; tapering distally; with five stripes; subapical setae absent.

*Tergum* (Fig. 45c). Surface with irregular rows of U-shaped scale bases and scattered micropores. Posterior margin of tergum IV with rounded or triangular spines, about as long as wide.

*Gills* (Fig. 45d). Present on segments II–VII. Margin with alternating smaller and bigger denticles intercalating long, fine, simple setae. Tracheae extending from main trunk to inner and outer margins. Gill IV as long as length of segments V and 1/2 VI combined. Gill VII as long as length of segments VIII and 1/2 IX combined.

*Paraproct* (Fig. 45e). Distally not expanded, with many marginal, stout spines. Surface with U-shaped scale bases and scattered fine, simple setae and micropores. Postero-lateral extension (cercotractor) with small marginal spines.

###### Etymology.

Refers to the similarity and close relationship to *L.vultuosus*.

###### Distribution.

New Guinea.

###### Biological aspects.

The specimens were collected at an altitude of 1500 m a.s.l.

###### Type-material.

**Holotype.** Nymph (on slide, GBIFCH 00465213), Papua New Guinea, Enga, Wapanamanda, 1500 m, 06 Dec 2006, 05°38.11'S, 143°55.34'E, Balke & Kinibel (PNG 128). Deposited in ZSM. **Paratypes.** 30 nymphs (1 on slide, GBIFCH 00465214, 16 in alcohol, GBIFCH 00515223, deposited in MZL; 13 in alcohol, GBIFCH 00515224, deposited in ZSM), same data as holotype.

#### Species not assigned to a group

##### 25. Labiobaetis centralensis sp. n.

Taxon classificationAnimaliaEphemeropteraBaetidae

http://zoobank.org/41833252-4CDE-408E-BE81-803010BF58EC

**Figure 46.**
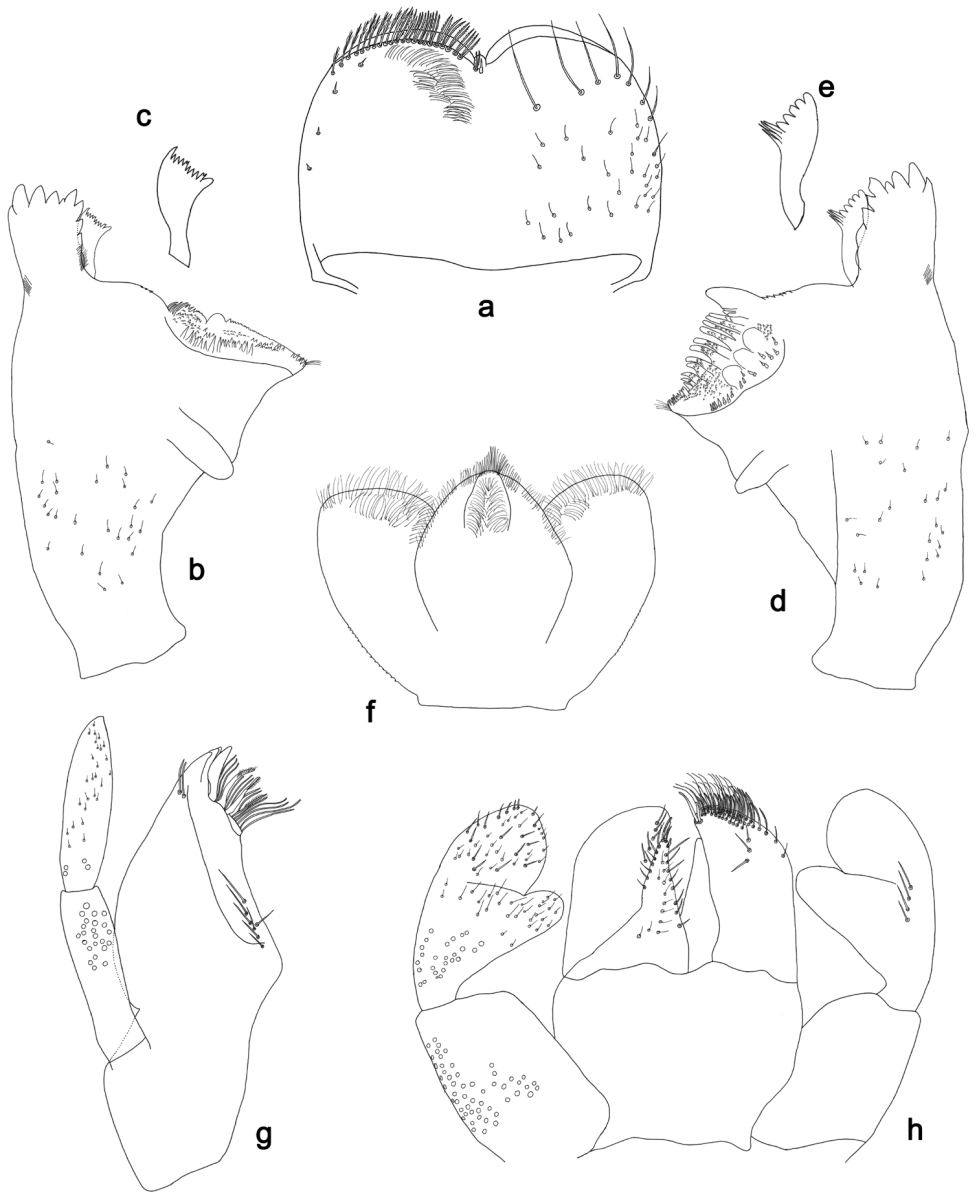
*Labiobaetiscentralensis* sp. n., larva morphology: **a** Labrum **b** Right mandible **c** Right prostheca **d** Left mandible **e** Left prostheca **f**Hypopharynx**g** Maxilla **h** Labium.

**Figure 47.**
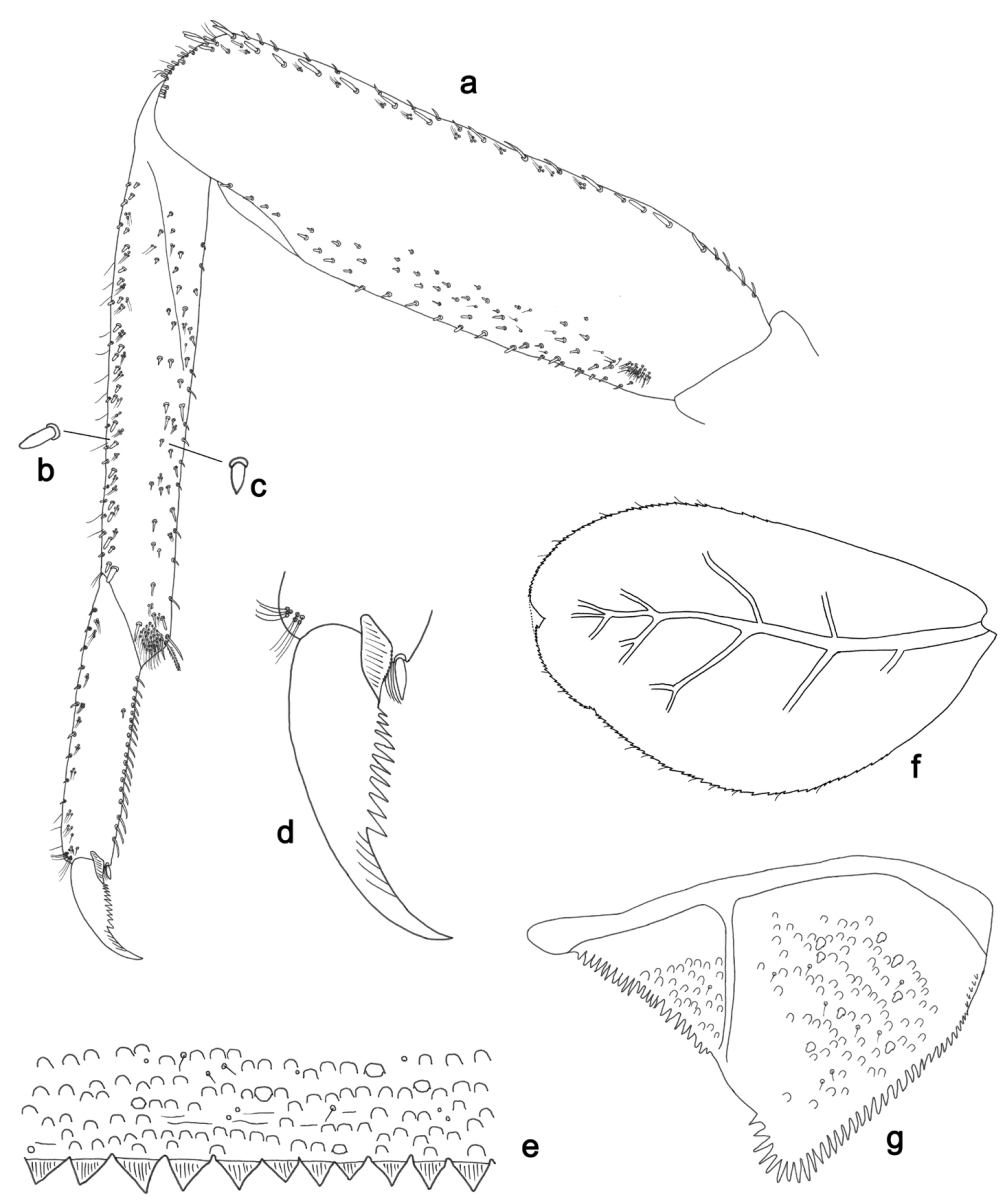
*Labiobaetiscentralensis* sp. n., larva morphology: **a**Foreleg**b** Tibia dorsal seta **c** Tibia ventral seta **d** Fore claw **e**Tergum IV **f** Gill IV **g**Paraproct.

Figures 46 , 47 , 63a , 65a

###### Diagnosis.

**Larva.** Following combination of characters: A) labrum with dorsal submarginal arc of setae composed of one plus six long, simple setae; B) labial palp segment II with an elongated, thumb-like distomedial protuberance; C) maxillary palp slightly longer than length of galea-lacinia, segment II apically slightly pointed and without excavation at inner lateral margin; D) right mandible outer and inner sets of denticles with 4 + 4 denticles respectively plus one small intermediate denticle; E) left mandible outer and inner sets of denticles with 4 + 4 denticles respectively; F) fore femur slender, length ca. 4× maximum width, dorsal margin with a row of ca. 21 curved, spine-like setae and distally a row of robust, spine-like setae close to margin.

###### Description.

**Larva** (Figs 46, 47, 63a). Body length 4.8 mm.

*Colouration.* Head, thorax and abdomen dorsally brown, with bright pattern as in Fig. 63a. Head and thorax with bright median, dorsal suture, forewing pads with bright striation. Head, thorax, and abdomen ventrally colourless, femur, tibia, and tarsus with brown dorsal margin, legs otherwise colourless, caudal filaments brown.

*Antenna* with scape and pedicel sub-cylindrical, without distolateral process at scape; flagellum with broad spines on apex of each segment.

*Labrum* (Fig. 46a). Rectangular, length 0.6× maximum width. Distal margin with medial emargination and a small process. Dorsally with medium, fine, simple setae scattered over surface; submarginal arc of setae composed of one plus six long, simple setae. Ventrally with marginal row of setae composed of anterolateral long, feathered setae and medial long, bifid, pectinate setae; ventral surface with four short, spine-like setae near lateral and anterolateral margin.

*Right mandible* (Fig. 46b, c). Incisors fused. Outer and inner sets of denticles with 4 + 4 denticles plus one small intermediate denticle. Inner margin of innermost denticle with a row of thin setae. Prostheca robust, apically denticulate. Margin between prostheca and mola slightly convex, with minute denticles. Tuft of setae at apex of mola present.

*Left mandible* (Fig. 46d, e). Incisors fused. Outer and inner sets of denticles with 4 + 4 denticles. Prostheca robust, apically with small denticles and comb-shape structure. Margin between prostheca and mola slightly convex, with minute denticles toward subtriangular process. Subtriangular process long and slender, above level of area between prostheca and mola. Denticles of mola not constricted. Tuft of setae at apex of mola present.

Both mandibles with lateral margins almost straight. Basal half with fine, simple setae scattered over dorsal surface.

*Hypopharynx* (Fig. 46f). Lingua longer than superlingua. Lingua about as broad as long; medial tuft of stout setae present; distal half laterally expanded. Superlingua rounded; lateral margin rounded; fine, long, simple setae along distal margin.

*Maxilla* (Fig. 46g). Galea-lacinia with two simple, robust apical setae under crown. Inner dorsal row of setae with three denti-setae, distal denti-seta tooth-like, middle and proximal denti-setae slender, bifid and pectinate. Medially with one spine-like seta and six long, simple setae. Maxillary palp slightly longer than length of galea-lacinia; two segmented. Palp segment II about as long as segment I. Fine and simple setae scattered over surface of segment II. Apex of segment II slightly pointed, without excavation at inner distolateral margin.

*Labium* (Fig. 46h). Glossa basally broad, narrowing toward apex; shorter than paraglossa; inner margin with eight spine-like setae increasing in length distally; apex with two long and one short, robust, pectinate setae; outer margin with seven spine-like setae increasing in length distally; ventral surface with fine, simple, scattered setae. Paraglossa sub-rectangular, curved inward; apex rounded; with three rows of long, robust, apically pectinate setae; dorsally with row of three medium, simple setae; ventrally with five medium, spine-like setae near inner margin. Labial palp with segment I 0.7× length of segments II and III combined. Segment I dorsally covered with micropores. Segment II with an elongated, thumb-like distomedial protuberance; distomedial protuberance 0.5× width of base of segment III; inner margin with few fine, simple setae; outer margin bare; dorsally with row of four long, spine-like, simple setae. Segment III conical; apex rounded; ventrally covered with medium spine-like, simple setae and short, fine, simple setae.

*Hind wing pads* absent.

*Foreleg* (Fig. 47a–d). Ratio of foreleg segments 1.2:1.0:0.5:0.2. *Femur*. Length ca. 4× maximum width. Dorsal margin with a row of ca. 21 curved, spine-like setae, distally a row of robust, spine-like setae close to margin, and a row of fine simple setae along margin; length of setae 0.1× maximum width of femur. Apex rounded; with two pairs of spine-like setae, many short, stout, blunt setae and some fine, long, simple setae. Many stout, lanceolate setae and a few fine, simple setae along ventral margin; femoral patch well developed. *Tibia.* Dorsal margin with stout, lanceolate, apically rounded setae and very fine, simple setae along margin. Ventral margin with a row of curved, spine-like setae, one seta on apex much longer; one long, bipectinate seta and a tuft of fine, long, simple setae on apex. Anterior surface scattered with many stout, lanceolate setae. Tibio-patellar suture present on basal 1/2. *Tarsus.* Dorsal margin with a row of short, spine-like setae and fine, simple setae along margin, especially on apex. Ventral margin with a row of curved, spine-like setae. Tarsal claw with one row of 10–11 denticles; distally pointed; with five stripes; subapical setae absent.

*Tergum* (Fig. 47e). Surface with irregular rows of U-shaped scale bases and scattered fine, simple setae and micropores, scales short, apically rounded. Posterior margin of tergum IV with triangular spines, about as long as wide.

*Gills* (Fig. 47f). Present on segments II–VII. Margin with small denticles intercalating long, fine, simple setae. Tracheae extending from main trunk to inner and outer margins, pigmentation limited to part of main trunk and one or two extensions to inner margin only. Gill IV as long as length of segments V and 1/2 VI combined. Gill VII as long as length of segments VIII and 1/2 IX combined.

*Paraproct* (Fig. 47g). Distally slightly expanded, with many marginal, stout spines. Surface with U-shaped scale bases and scattered fine, simple setae. Postero-lateral extension (cercotractor) with small marginal spines.

###### Etymology.

Refers to the type locality in the Central Province of Papua New Guinea.

###### Distribution.

New Guinea.

###### Biological aspects.

The specimens were collected at an altitude of 590 m a.s.l.

###### Type-material.

**Holotype**. Nymph (on slide, GBIFCH 00465215), Papua New Guinea, Central, Kokoda Trek, 590 m, 01.2008, 09°14.34'S, 147°36.92'E, Posman (PNG170). Deposited in ZSM. **Paratypes**. 16 nymphs (1 on slide, GBIFCH 00465216, 8 in alcohol, GBIFCH 00515217, deposited in MZL; 7 in alcohol, GBIFCH 00515218, deposited in ZSM), same data as holotype.

##### 26. Labiobaetis dendrisetis sp. n.

Taxon classificationAnimaliaEphemeropteraBaetidae

http://zoobank.org/E7CFB714-938F-4291-B046-F085CC363D63

**Figure 48.**
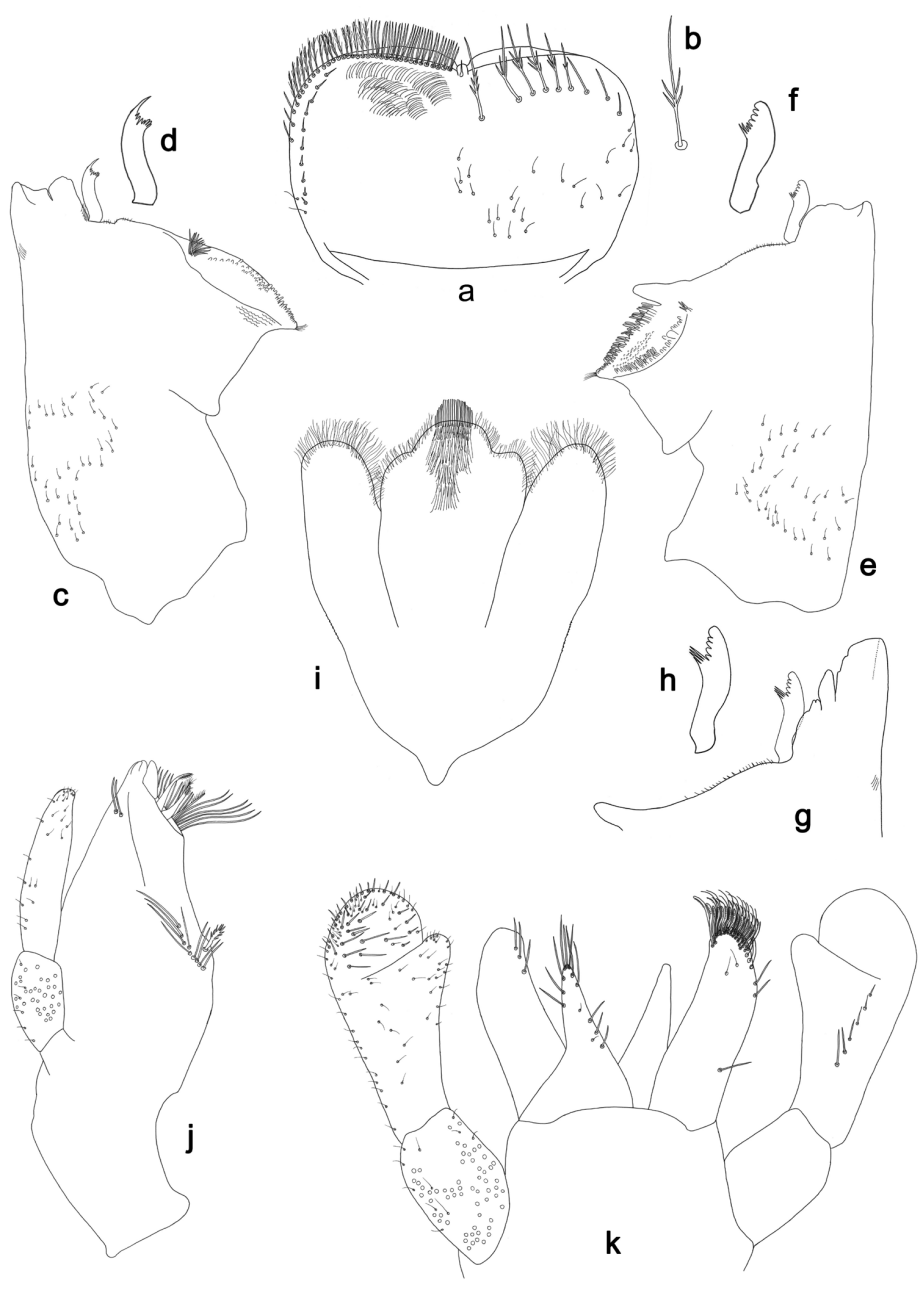
*Labiobaetisdendrisetis* sp. n., larva morphology: **a** Labrum **b** Labrum dorsal, submarginal seta **c** Right mandible, denticles outworn **d** Right prostheca **e** Left mandible, denticles outworn **f** Left prostheca **g** Left mandible, denticles unused **h** Left prostheca **i**Hypopharynx**j** Maxilla **k** Labium.

**Figure 49.**
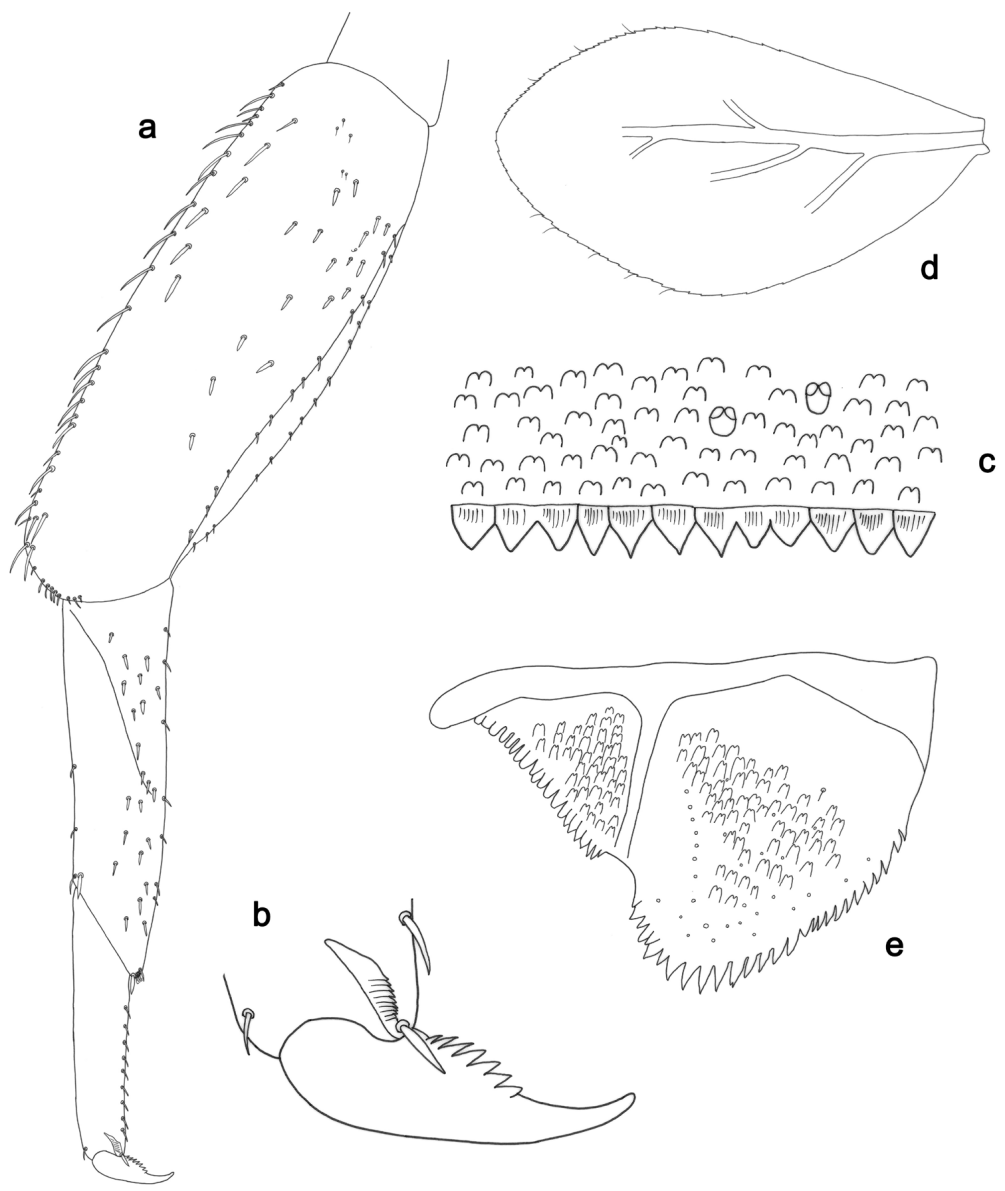
*Labiobaetisdendrisetis* sp. n., larva morphology: **a**Foreleg**b** Fore claw **c**Tergum IV **d** Gill IV **e**Paraproct.

Figures 48 , 49 , 65b

###### Diagnosis.

**Larva.** Following combination of characters: A) labrum dorsal submarginal arc of setae composed of one plus 5 long, dendritic, apically pointed setae and 3 middle to long, simple setae; B) both mandibles with outermost denticles blade-like; C) maxillary palp somewhat shorter than length of galea-lacinia; D) distomedial protuberance of labial palp segment II short, thumb-like; E) fore femur rather broad, length ca. 3× maximum width, dorsal margin with a row of ca. 20 curved, spine-like setae and basally a row of robust, spine-like setae near margin; F) tarsal claw with one row of seven denticles and without striation.

###### Description.

**Larva** (Figs 48, 49). Antenna approximately twice as long as head length.

*Colouration.* Unknown.

*Antenna* with scape and pedicel sub-cylindrical, without distolateral process at scape; flagellum with lanceolate spines on apex of each segment.

*Labrum* (Fig. 48a, b). Rectangular, length 0.5× maximum width. Distal margin with medial emargination and a small process. Dorsally with medium, fine, simple setae scattered over surface; submarginal dorsal arc of setae composed of one plus five long, dendritic, apically pointed setae and three middle to long, simple setae. Ventrally with marginal row of setae composed of lateral and anterolateral long, feathered setae and medial long, bifid setae; ventral surface with ten short, spine-like setae near lateral and anterolateral margin.

*Right mandible* (Fig. 48c, d). Incisors fused. Outer and inner sets of denticles unknown, outermost denticle blade-like. Inner margin of innermost denticle with a row of thin setae. Prostheca robust, apically denticulate. Margin between prostheca and mola straight, with minute setae. Tuft of setae at apex of mola present.

*Left mandible* (Fig. 48e–h). Incisors fused. Outer and inner sets of denticles with 3 + 3 denticles, outermost denticle blade-like. Prostheca robust, apically with small denticles and comb-shape structure. Margin between prostheca and mola straight, with minute setae. Subtriangular process long and slender, above level of area between prostheca and mola. Denticles of mola apically constricted. Tuft of setae at apex of mola present.

Both mandibles with lateral margins almost straight. Basal half with fine, simple setae scattered over dorsal surface.

*Hypopharynx* (Fig. 48i). Lingua longer than superlingua. Lingua longer than broad; medial tuft of stout setae present; distal half laterally expanded. Superlingua rounded; lateral margin straight; fine, long, simple setae along distal margin.

*Maxilla* (Fig. 48j). Galea-lacinia with two simple, robust apical setae under crown. Inner dorsal row of setae with three denti-setae, distal denti-seta tooth-like, middle and proximal denti-setae slender, bifid and pectinate. Medially with one bipectinate, spine-like seta and 9–10 long, simple setae. Maxillary palp somewhat shorter than length of galea-lacinia; two segmented. Palp segment II 1.8× length of segment I. Setae on maxillary palp fine and simple, scattered over surface of segment II and along outer margin of segment I. Apex of last segment rounded, with slight excavation at inner distolateral margin.

*Labium* (Fig. 48k). Glossa basally broad, narrowing toward apex; shorter than paraglossa; inner margin with five spine-like setae increasing in length distally; apex with three long, robust setae; outer margin with 3–4 long, spine-like setae; ventral surface with few short, fine, simple setae. Paraglossa sub-rectangular, curved inward; apex rounded; with three rows of long, robust, apically pectinate setae; dorsally with two medium, simple setae; ventrally with three long, spine-like setae near inner margin. Labial palp with segment I 0.6× length of segments II and III combined. Segment I dorsally covered with micropores and ventrally with fine, simple setae along margins. Segment II with a short, thumb-like distomedial protuberance; distomedial protuberance 0.2× width of base of segment III; inner and outer margin both with short, fine, simple setae; dorsally with row of six spine-like, simple setae, decreasing in length distally. Segment III semicircular; apex rounded; length 0.8× width; covered with long and medium spine-like, simple setae and short, fine, simple setae.

*Hind wing pads* absent.

*Foreleg* (Fig. 49a, b). Ratio of foreleg segments 1.8:1.0:0.6:0.2. *Femur*. Length ca. 3× maximum width. Dorsal margin with a row of ca. 20 curved, spine-like setae, basally a row of robust, spine-like setae near margin; length of setae 0.18× maximum width of femur. Apex rounded; with one pair of curved, spine-like setae and many short, stout, pointed setae. Ventral margin with a row of short, spine-like setae, some stout, lanceolate, pointed setae and a few fine, simple setae scattered along ventral margin; femoral patch absent. *Tibia.* Dorsal margin with a few short, curved, spine-like setae and a pair of longer, curved, spine-like setae on apex. Ventral margin with a row of curved, spine-like setae, on apex one stout, spine-like seta and a tuft of long, fine, simple setae. Anterior surface scattered with many stout, lanceolate setae. Tibio-patellar suture present on basal 1/2. *Tarsus.* Dorsal margin with one or two short, spine-like setae near apex. Ventral margin with a row of curved, spine-like setae. Tarsal claw with one row of seven denticles; tapering distally; striation absent.

*Tergum* (Fig. 49c). Surface with irregular rows of W-shaped scale bases, scales broad, apically rounded. Posterior margin of tergum IV with triangular or pentagonal spines, about as long as wide.

*Gills* (Fig. 49d). Present on segments I–VII. Margin with small denticles intercalating long, fine, simple setae. Tracheae partly extending from main trunk towards outer and inner margins. Gill I as long as length of segment II. Gill IV as long as length of segments V and VI combined. Gill VII as long as length of segments VIII and 1/3 IX combined.

*Paraproct* (Fig. 49e). Distally not expanded, with many marginal, stout spines. Surface with W-shaped scale bases and scattered fine, simple setae and micropores. Postero-lateral extension (cercotractor) with small marginal spines.

###### Etymology.

Refers to the dendritic submarginal setae on dorsal surface of labrum.

###### Distribution.

New Guinea.

###### Biological aspects.

The specimens were collected in forest at an altitude of 2900 m a.s.l.

###### Type-material.

**Holotype.** Nymph (on slide, GBIFCH 00465217), Papua New Guinea, Simbu Prov., 05°49.00'S, 145°04.50'E, Mt. Wilhelm, Pindaunde Creek, 2900 m a.s.l. (in forest), S3 (oria.4), 18 Aug 1999, leg. L. Čížek . Deposited in MZL. **Paratype.** 1 nymph (on 2 slides, GBIFCH 00465218, deposited in MZL), same data as holotype.

##### 27. Labiobaetis elisae sp. n.

Taxon classificationAnimaliaEphemeropteraBaetidae

http://zoobank.org/9C39EBAE-03F5-439E-8E14-839BC8DB51B5

**Figure 50.**
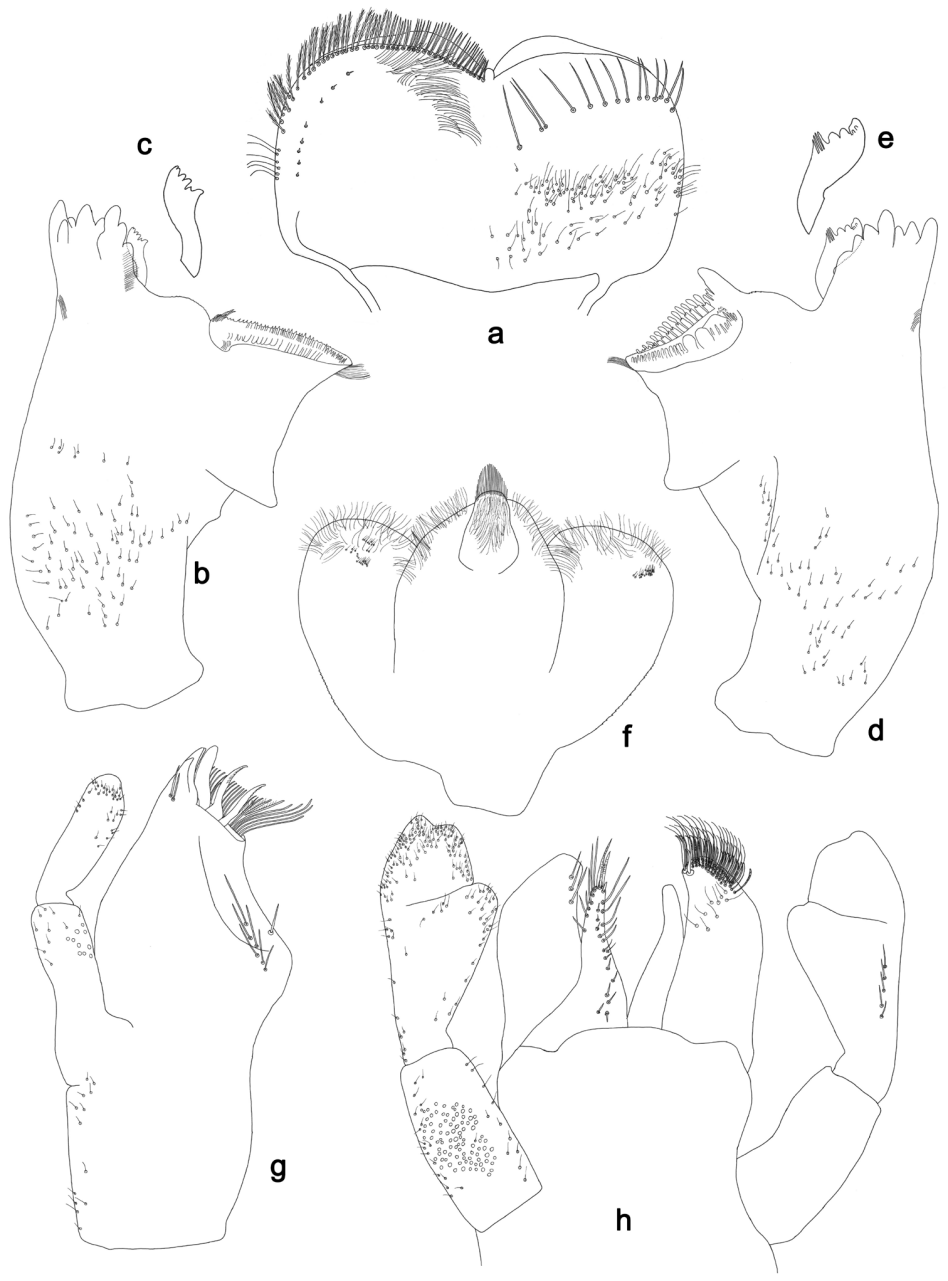
*Labiobaetiselisae* sp. n., larva morphology: **a** Labrum **b** Right mandible **c** Right prostheca **d** Left mandible **e** Left prostheca **f**Hypopharynx**g** Maxilla **h** Labium.

**Figure 51.**
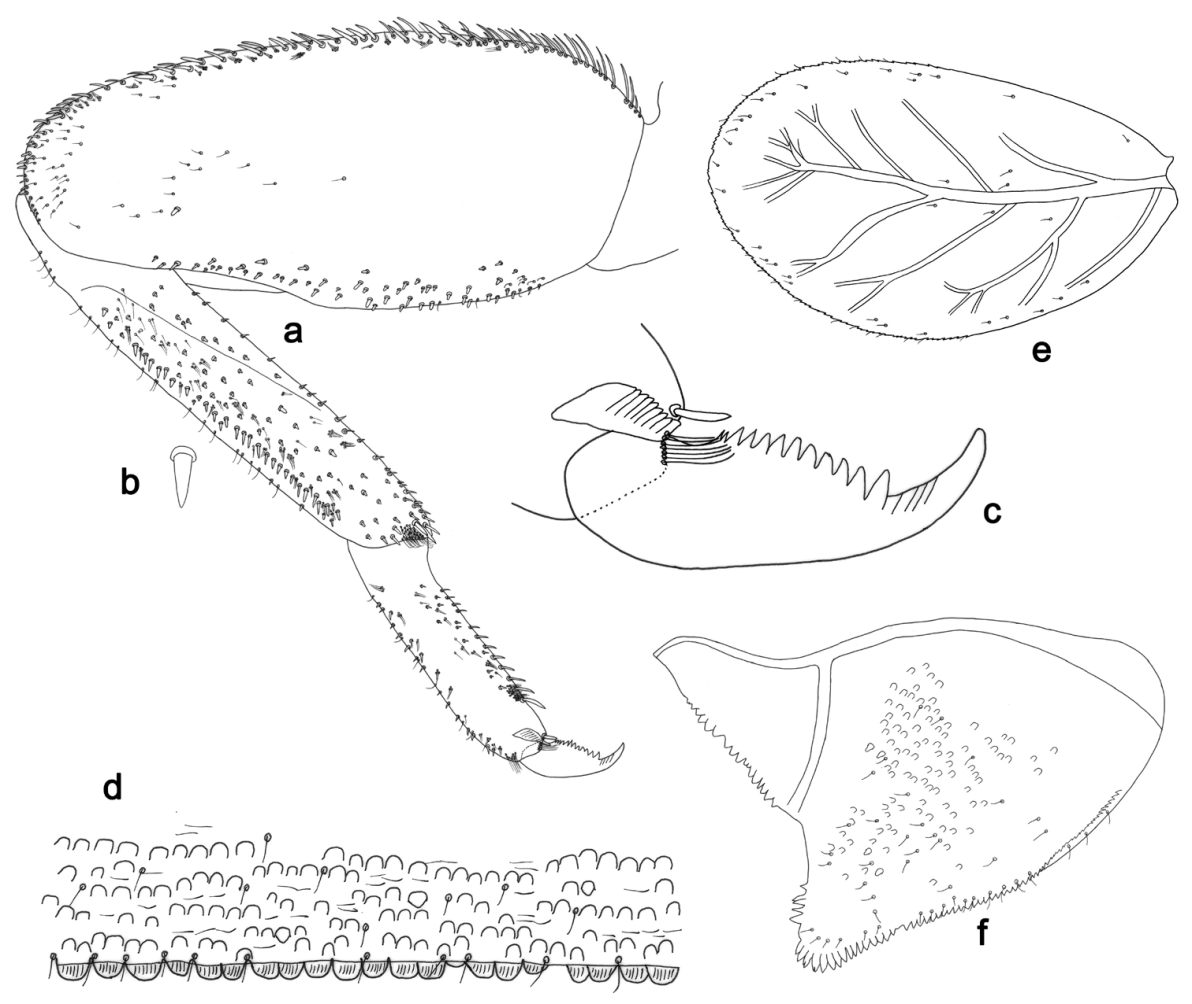
*Labiobaetiselisae* sp. n., larva morphology: **a**Foreleg**b** Tibia dorsal seta **c** Fore claw **d**Tergum IV **e** Gill IV **f**Paraproct.

Figures 50 , 51 , 63b , 64a

###### Diagnosis.

**Larva.** Following combination of characters: A) labrum dorsal submarginal arc of setae composed of one plus 11–13 long, simple setae; B) labrum dorsally with many medium, fine, simple setae, mainly arranged in one band; C) labial palp segment III with emargination at distal margin; D) maxillary palp shorter than galea-lacinia; E) fore femur broad, length ca. 2× maximum width, dorsal margin with an irregular row of more than 40 curved, spine-like setae and some curved, spine like setae near margin.

###### Description.

**Larva** (Figs 50, 51, 63b). Body length 9.6 mm.

*Colouration.* Head, thorax and abdomen dorsally brown, abdominal terga I and X lighter. Head and thorax with bright median, dorsal suture, forewing pads with bright striation. Thorax and abdomen ventrally light brown, femur with brown dorsal margin, legs otherwise light brown, caudal filaments light brown.

*Antenna* with scape and pedicel sub-cylindrical, without distolateral process at scape; flagellum with broad, apically blunt spines and fine, simple setae on apex of each segment.

*Labrum* (Fig. 50a). Rectangular, length 0.6× maximum width. Distal margin with medial emargination and a small process. Dorsally with many medium to long, fine, simple setae, mainly arranged in one band; submarginal arc of setae composed of one plus 11–13 long, simple setae. Lateral margin with few long, fine, simple setae. Ventrally with marginal row of setae composed of lateral and anterolateral long, feathered setae and medial long, bifid setae; ventral surface with eight short, spine-like setae near lateral and anterolateral margin.

*Right mandible* (Fig. 50b, c). Incisors fused. Outer and inner sets of denticles with 4 + 3 denticles plus one small intermediate denticle. Inner margin of innermost denticle with a row of thin setae. Prostheca robust, apically denticulate. Margin between prostheca and mola slightly convex. Tuft of setae at apex of mola present.

*Left mandible* (Fig. 50d, e). Incisors fused. Outer and inner sets of denticles with 3 + 4 denticles. Prostheca robust, apically with small denticles and comb-shape structure. Margin between prostheca and mola slightly convex, with minute denticles toward subtriangular process. Subtriangular process long and slender, above level of area between prostheca and mola. Denticles of mola apically constricted. Tuft of setae at apex of mola present.

Both mandibles with lateral margins slightly convex. Basal half with fine, simple setae scattered over dorsal surface.

*Hypopharynx* (Fig. 50f). Lingua longer than superlingua. Lingua about as broad as long; medial tuft of stout setae present; distal half not expanded. Superlingua rounded; lateral margin rounded; fine, long, simple setae along distal margin and short, simple setae in distal part.

*Maxilla* (Fig. 50g). Galea-lacinia with two simple, robust apical setae under crown. Inner dorsal row of setae with three denti-setae, distal denti-seta tooth-like, middle and proximal denti-setae slender, bifid and pectinate. Medially with one spine-like seta and six long, simple setae. Maxillary palp shorter than length of galea-lacinia; two segmented. Palp segment II somewhat shorter than segment I. Setae on maxillary palp fine and simple, scattered over surface of segments I and II. Palp segment III about as long as segment II. Apex of last segment rounded, without excavation at inner distolateral margin.

*Labium* (Fig. 50h). Glossa basally broad, narrowing toward apex; shorter than paraglossa; inner margin with six spine-like setae increasing in length distally; apex with three long, robust, pectinate setae and one short, robust seta; outer margin with six long spine-like setae increasing in length distally; ventral surface with short, simple setae. Paraglossa sub-rectangular, curved inward; apex rounded; with three rows of long, robust, apically pectinate setae; dorsally with nine medium, simple setae; ventrally with three long, spine-like setae near inner margin. Labial palp with segment I 0.8× length of segments II and III combined. Segment I dorsally covered with micropores and ventrally with fine, simple setae along margins. Segment II with a short, thumb-like distomedial protuberance; distomedial protuberance 0.2× width of base of segment III; inner and outer margin both with short, fine, simple setae; dorsally with row of five long, spine-like, simple setae. Segment III conical; apex with emargination; ventrally covered with short, fine, simple setae.

*Hind wing pads* absent.

*Foreleg* (Fig. 51a–c). Ratio of foreleg segments 1.2:1.0:0.5:0.2. *Femur*. Length ca. 2× maximum width. Dorsal margin with an irregular row of more than 40 curved, spine-like setae and some curved, spine-like setae and fine, simple setae near margin; length of setae 0.1× maximum width of femur. Apex rounded; with one pair of curved, spine-like setae and many short, stout, apically rounded setae. Many stout, lanceolate, setae and a few fine, simple setae along ventral margin; femoral patch poorly developed. *Tibia.* Dorsal margin with a row of fine, simple setae and a row of stout, lanceolate setae along margin. Ventral margin with a row of short, curved, spine-like setae and a tuft of long, fine, simple setae on apex. Anterior surface scattered with stout, lanceolate, apically rounded setae and fine, simple setae. Tibio-patellar suture present on basal 2/3. *Tarsus.* Dorsal margin with a row of short, spine-like setae and a row of long, fine, simple setae on and near margin, especially on apex. Ventral margin with a row of curved, spine-like setae. Tarsal claw with one row of 11–12 denticles; tapering distally; with 4–6 stripes; subapical setae absent.

*Tergum* (Fig. 51d). Surface with irregular rows of U-shaped scale bases and scattered fine, simple setae, scales short and apically rounded. Posterior margin of tergum IV with rounded spines, wider than long.

*Gills* (Fig. 51e). Present on segments II - VII. Margin with small denticles intercalating long, fine, simple setae. Tracheae extending from main trunk to inner and outer margins. Gill IV as long as length of segments V and 1/2 VI combined. Gill VII little longer than length of segment VIII.

*Paraproct* (Fig. 51f). Distally expanded, with many marginal, stout spines. Surface with U-shaped scale bases and scattered fine, simple setae. Postero-lateral extension (cercotractor) with small marginal spines.

###### Etymology.

Dedicated to Elisa Gattolliat, the daughter of one of the authors.

###### Distribution.

New Guinea.

###### Biological aspects.

The specimens were collected in altitudes of 1200 m and 1400 m a.s.l.

###### Type-material.

**Holotype.** Nymph (on slide, GBIFCH 00465219), Papua New Guinea, Western Highlands, Kundum, 1400 m, 03 Mar 2007, 05°16.10'S, 144°27.87'E, Kinibel (PNG142). Deposited in ZSM. **Paratypes.** 31 nymphs (1 on slide, GBIFCH 00465220, 20 in alcohol, GBIFCH 00515221; 10 in alcohol, GBIFCH 00515222, deposited in ZSM), same data as holotype; 1 nymph (on slide, GBIFCH 00465221, remaining parts in alcohol, GBIFCH 00515287, deposited in MZL), Papua New Guinea, Madang, Simbai area, 1200 m, 10 Mar 2007, 05°13.39'S, 144°37.29'E, Kinibel (PNG 152); 1 nymph (on slide, GBIFCH 00465231, deposited in MZL), Papua New Guinea, Eastern Highlands, Bena, 1393 m, 20 Oct 2002, 06°11.02'S, 145°26.41'E, grassland river, K. Sagata leg.

##### 28. Labiobaetis inopinatus sp. n.

Taxon classificationAnimaliaEphemeropteraBaetidae

http://zoobank.org/D3287F9C-B2BB-4DB6-8341-BAD6F11F1623

**Figure 52.**
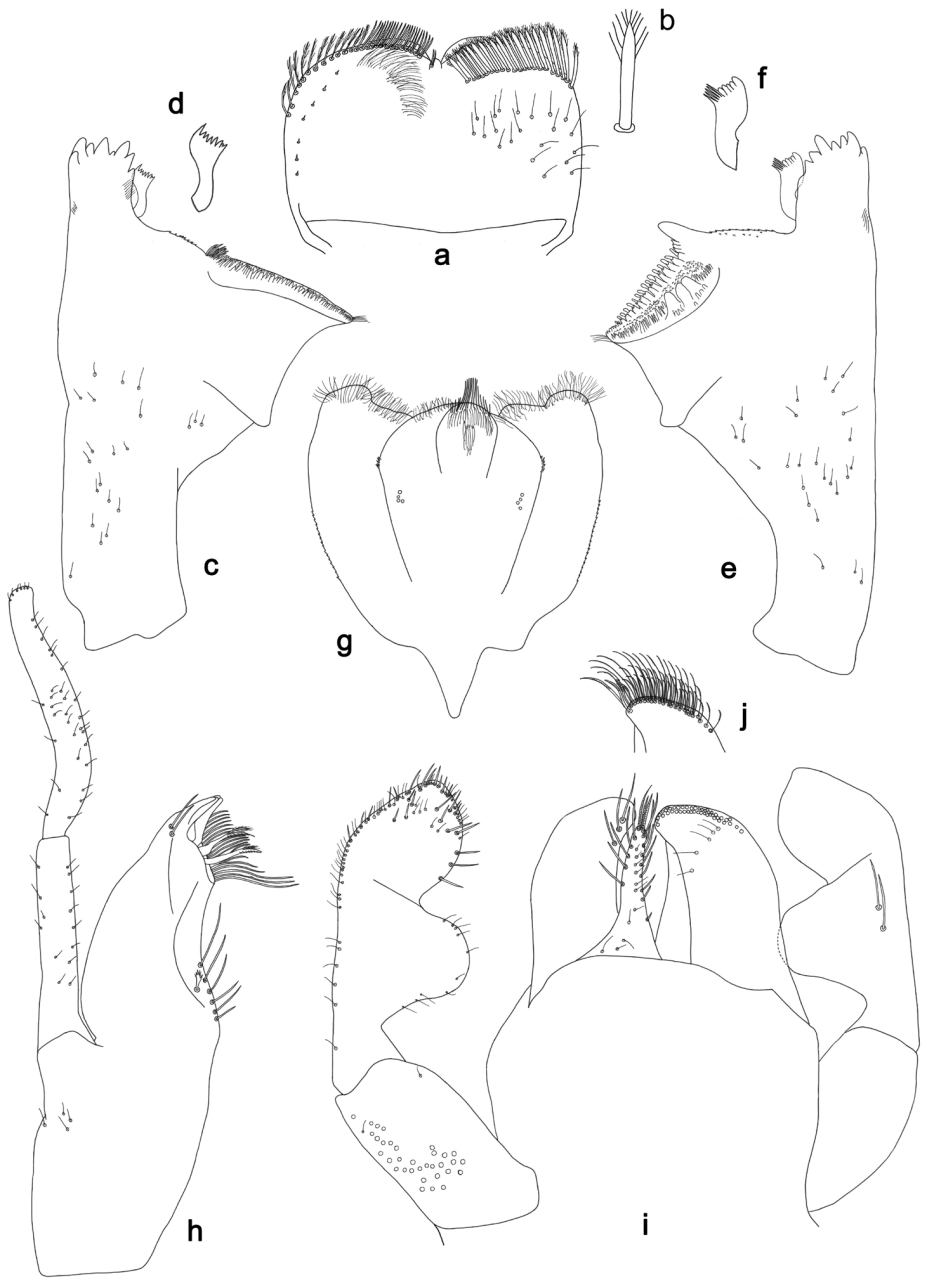
*Labiobaetisinopinatus* sp. n., larva morphology: **a** Labrum **b** Labrum dorsal, submarginal seta **c** Right mandible **d** Right prostheca **e** Left mandible **f** Left prostheca **g**Hypopharynx**h** Maxilla **i** Labium.

**Figure 53.**
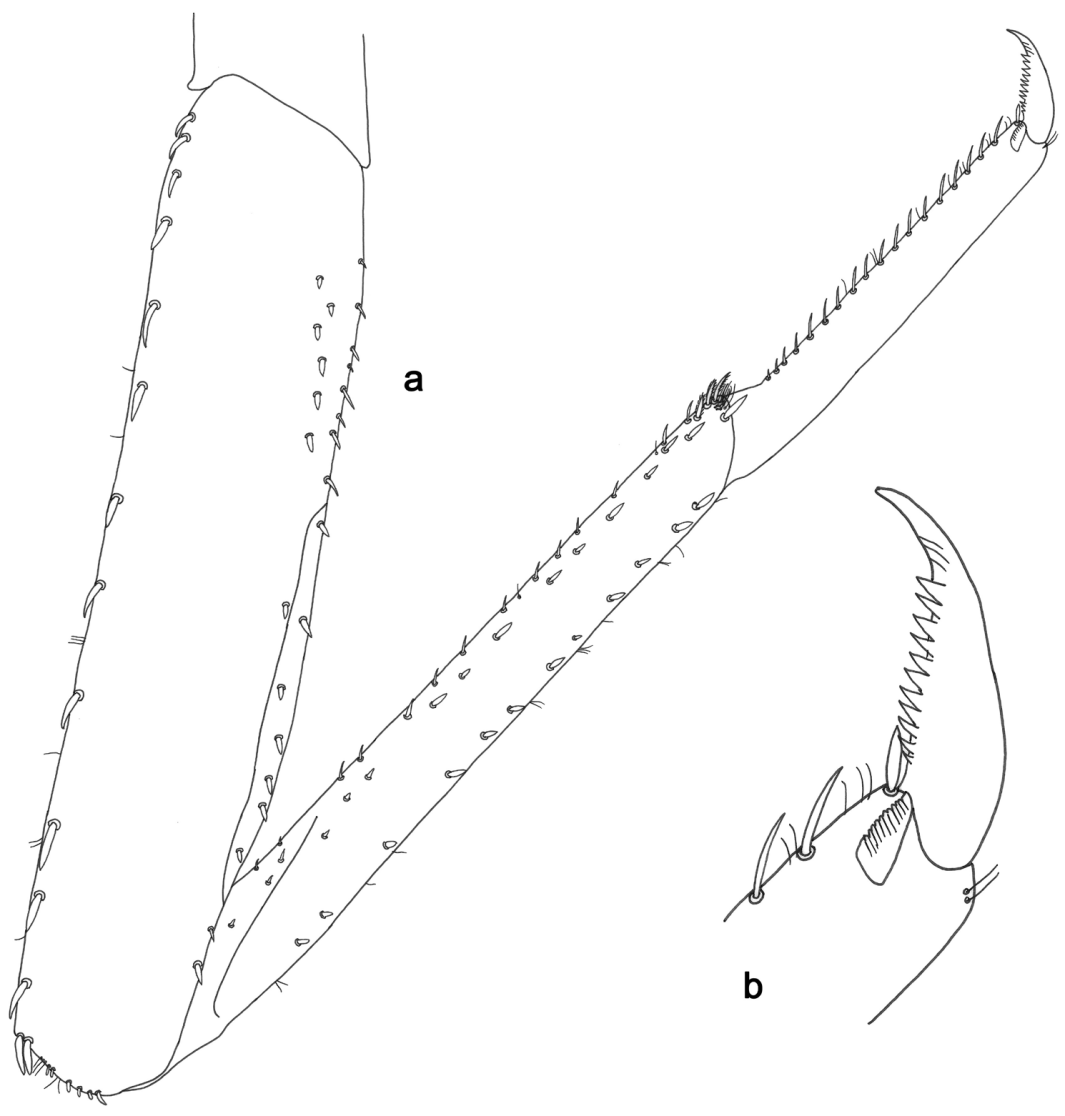
*Labiobaetisinopinatus* sp. n., larva morphology: **a**Foreleg**b** Fore claw.

Figures 52 , 53 , 64b

###### Diagnosis.

**Larva.** Following combination of characters: A) labrum dorsal submarginal arc of setae composed of 21 long, lanceolate, apically pectinate setae; B) maxillary palp much longer than length of galea-lacinia, apically rounded, with excavation at inner distolateral margin; C) labial palp segment II with a large, lobed distomedial protuberance, segment III slightly pentagonal, apically slightly pointed; D) fore femur very slender, length ca. 5× maximum width, dorsal margin with a row of ca. 12 curved, spine-like setae; E) fore claw with one row of eleven denticles.

###### Description.

**Larva** (Figs 52, 53).

*Colouration.* Unknown.

*Labrum* (Fig. 52a, b). Rectangular, length 0.7× maximum width. Distal margin with medial emargination and a small process. Dorsally with medium, fine, simple setae scattered over surface; submarginal arc of setae composed of 21 long, lanceolate, apically pectinate setae. Ventrally with marginal row of setae composed of lateral and anterolateral long, feathered setae and medial long, bifid setae; ventral surface with seven short, spine-like setae near lateral and anterolateral margin.

*Right mandible* (Fig. 52c, d). Incisors fused. Outer and inner sets of denticles with 4 + 3 denticles plus one small intermediate denticle. Inner margin of innermost denticle with a row of thin setae. Prostheca robust, apically denticulate. Margin between prostheca and mola straight, with minute denticles. Tuft of setae at apex of mola present.

*Left mandible* (Fig. 52e, f). Incisors fused. Outer and inner sets of denticles with 4 + 4 denticles. Prostheca robust, apically with small denticles and comb-shape structure. Margin between prostheca and mola straight, with minute denticles towards subtriangular process. Subtriangular process long and slender, above level of area between prostheca and mola. Denticles of mola apically constricted. Tuft of setae at apex of mola present.

Both mandibles with lateral margins almost straight. Basal half with fine, simple setae scattered over dorsal surface.

*Hypopharynx* (Fig. 52g). Lingua shorter than superlingua. Lingua longer than broad; medial tuft of stout setae present; distal half laterally expanded. Superlingua slightly concave; lateral margin rounded; fine, long, simple setae along distal margin.

*Maxilla* (Fig. 52h). Galea-lacinia with two simple, robust apical setae under crown. Inner dorsal row of setae with three denti-setae, distal denti-seta tooth-like, middle and proximal denti-setae slender, bifid and pectinate. Medially with one spine-like seta and six long, simple setae. Maxillary palp 1.7× as long as length of galea-lacinia; two segmented. Palp segment II 1.2× length of segment I. Setae on maxillary palp fine and simple, scattered over surface of segments I and II. Apex of last segment rounded, with excavation at inner distolateral margin.

*Labium* (Fig. 52i, j). Glossa basally broad, narrowing toward apex; shorter than paraglossa; inner margin with eight spine-like setae increasing in length distally; apex with three long, robust, pectinate setae; outer margin with five long, spine-like setae; ventral surface with short, fine, simple, scattered setae. Paraglossa sub-rectangular, curved inward; apex rounded; with three rows of long, robust, apically pectinate setae; dorsally with five medium, simple setae; ventrally with three long, spine-like setae near inner margin. Labial palp with segment I 0.7× length of segments II and III combined. Segment I covered with short, fine, simple setae ventrally and micropores dorsally. Segment II with a large, lobed distomedial protuberance; distomedial protuberance 0.7× width of base of segment III; inner and outer margin both with short, fine, simple setae; dorsally with two long, spine-like, simple setae. Segment III slightly pentagonal; apex slightly pointed; length 1.2× width; ventrally covered with short and medium spine-like, simple setae and short, fine, simple setae.

*Hind wing pads* unknown.

*Foreleg* (Fig. 53a, b). Ratio of foreleg segments 1.1:1.0:0.5:0.1. *Femur*. Length ca. 5× maximum width. Dorsal margin with a row of ca. 12 curved, spine-like setae; length of setae 0.2× maximum width of femur. Apex rounded; with one pair of curved, spine-like setae and some short, stout, pointed setae. Many stout, lanceolate setae along ventral margin; femoral patch absent. *Tibia.* Dorsal margin with a row of stout, lanceolate setae and very fine, simple setae. Ventral margin with a row of curved, spine-like setae and some longer, spine-like, bipectinate setae and a tuft of long, fine, simple setae on apex. Anterior surface scattered with stout, lanceolate setae. Tibio-patellar suture present on basal 1/3. *Tarsus.* Dorsal margin bare. Ventral margin with a row of curved, spine-like setae. Tarsal claw with one row of eleven denticles; tapering distally; with three stripes; subapical setae absent.

*Tergum*. Unknown.

*Gills*. Unknown.

*Paraproct*. Unknown.

###### Etymology.

Latin word for unexpected, refers to the unexpected finding of this species amongst other material.

###### Distribution.

New Guinea.

###### Type-material.

**Holotype.** Nymph (on slide, GBIFCH 00465230), Papua New Guinea, Gulf Prov., Supa-Hala, 1032 m, 10 Nov 2002, forest stream, K. Sagata leg. Deposited in ZSM.

##### 29. Labiobaetis involutus

Taxon classificationAnimaliaEphemeropteraBaetidae

(Lugo-Ortiz & McCafferty, 1999)

Figure 64b

###### Diagnosis.

**Larva.** Following combination of characters: A) labrum dorsal submarginal arc of setae composed of one plus 4–5 long, simple setae; B) labial palp segment II with elongated thumb-like distomadial protuberance, segment III about semicircular; C) maxillary palp shorter than length of galea-lacinia, without excavation at inner distolateral margin of segment II; D) fore femur rather broad, length ca. 3× maximum width; E) fore claw with a row of 10–12 denticles; F) spines at posterior margin of tergum IV triangular, pointed, longer than wide; G) paraproct distally not expanded.

###### Examined material.

**Paratypes.** 2 nymphs (on slides, PERC 0.012.560, PERC 0.012.561), Papua New Guinea, Bulolo River, East of Wau, 2950 ft, 15 Oct 1964, W.L. and J.G. Peters leg.

##### 30. Labiobaetis pindaundensis sp. n.

Taxon classificationAnimaliaEphemeropteraBaetidae

http://zoobank.org/4490390D-7D51-44BC-A303-E2339B8F8A68

**Figure 54.**
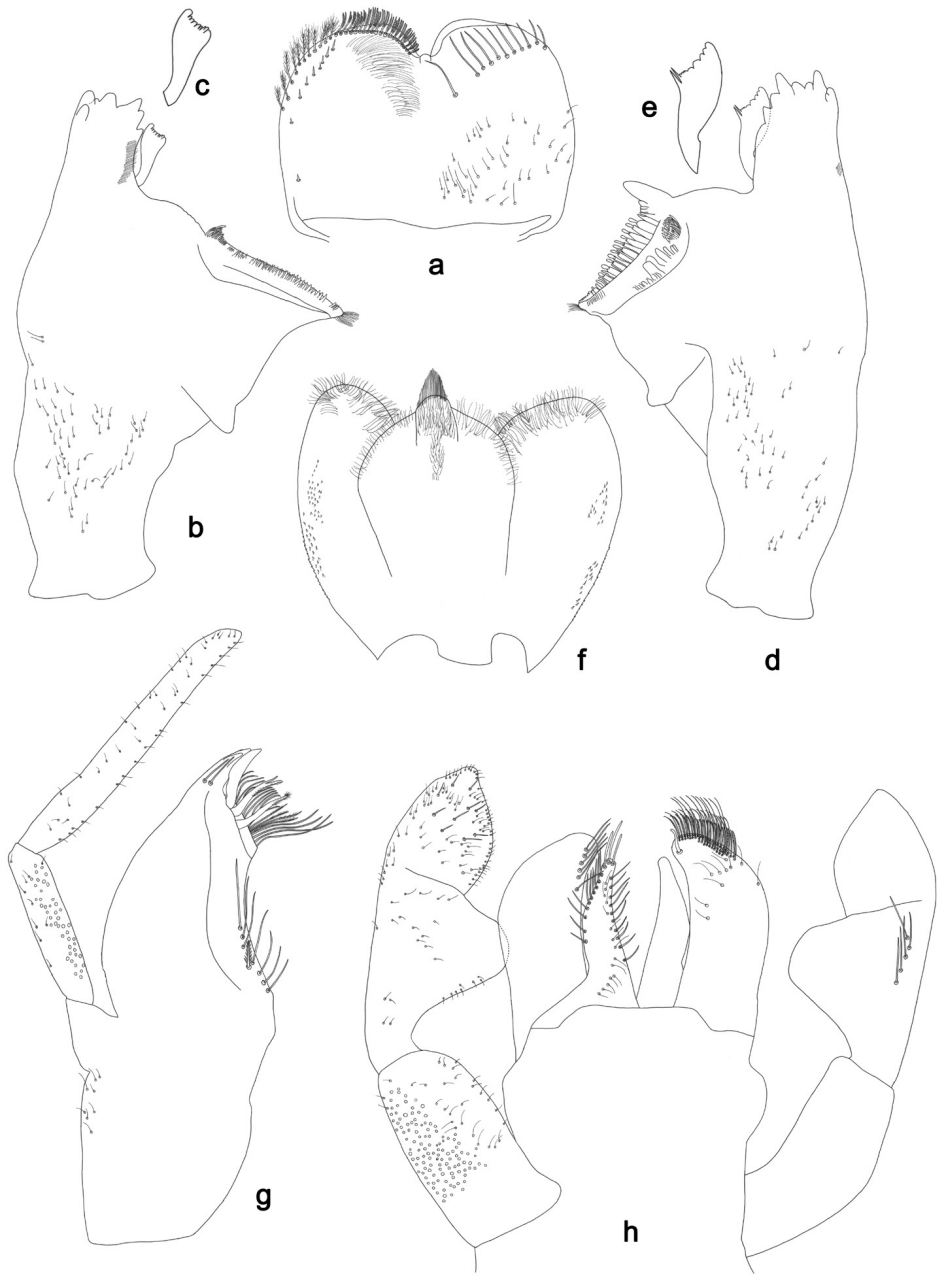
*Labiobaetispindaundensis* sp. n., larva morphology: **a** Labrum **b** Right mandible **c** Right prostheca **d** Left mandible **e** Left prostheca **f**Hypopharynx**g** Maxilla **h** Labium.

**Figure 55.**
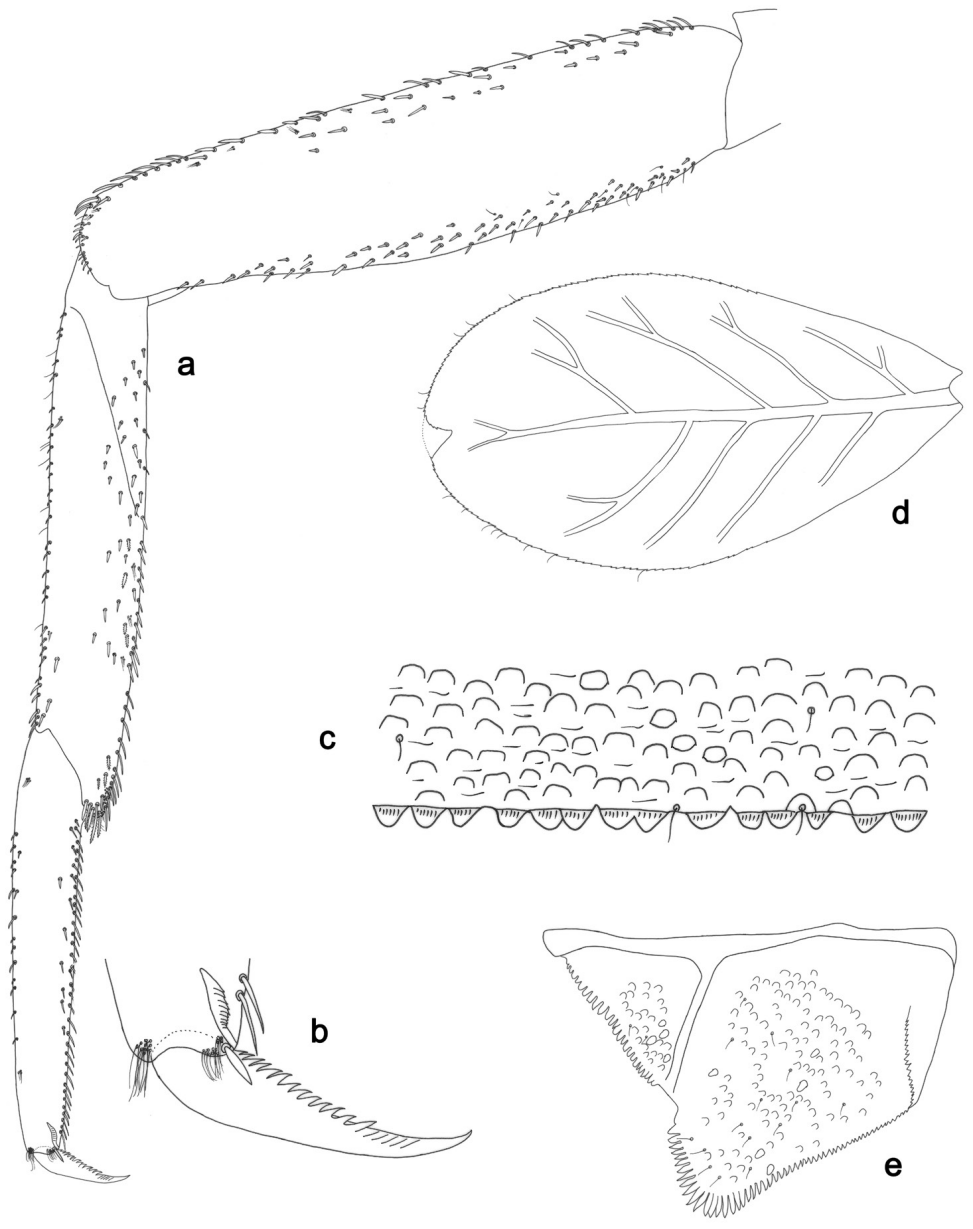
*Labiobaetispindaundensis* sp. n., larva morphology: **a**Foreleg**b** Fore claw **c**Tergum IV **d** Gill IV **e**Paraproct.

Figures 54 , 55 , 63c , 65b

###### Diagnosis.

**Larva.** Following combinations of characters: A) labrum dorsal submarginal arc of setae composed of one plus 10–12 long, simple setae; B) maxillary palp 1.6× as long as length of galea-lacinia; C) labial palp segment III conical, slightly pointed; D) labial palp segment II with a large, lobed distomedial protuberance; E) fore femur slender, length ca. 4× maximum width, dorsal margin with a row of ca. 27 curved, spine-like setae and with stout, pointed setae near margin.

###### Description.

**Larva** (Figs 54, 55, 63c). Body length 7.5 mm; antenna approximately 2.5× as long as head length.

*Colouration.* Head, thorax and abdomen dorsally brown, head and thorax with bright median, dorsal suture, forewing pads with bright striation. Head, thorax and abdomen ventrally light brown, legs colourless, caudal filaments light brown.

*Antenna* with scape and pedicel sub-cylindrical, without distolateral process at scape; flagellum with lanceolate spines on apex of each segment.

*Labrum* (Fig. 54a). Rectangular, length 0.7× maximum width. Distal margin with medial emargination and a small process. Dorsally with medium, fine, simple setae scattered over surface; submarginal arc of setae composed of one plus 10–12 long, simple setae. Ventrally with marginal row of setae composed of lateral and anterolateral long, feathered setae and medial long, bifid, pectinate setae; ventral surface with nine short, spine-like setae near lateral and anterolateral margin.

*Right mandible* (Fig. 54b, c). Incisors fused. Outer and inner sets of denticles with 4 + 3 denticles. Inner margin of innermost denticle with a row of thin setae. Prostheca robust, apically denticulate. Margin between prostheca and mola slightly convex. Tuft of setae at apex of mola present.

*Left mandible* (Fig. 54d, e). Incisors fused. Outer and inner sets of denticles with 4 + 3 denticles. Prostheca robust, apically with small denticles and comb-shape structure. Margin between prostheca and mola slightly convex. Subtriangular process long and slender, above level of area between prostheca and mola. Denticles of mola apically constricted. Tuft of setae at apex of mola present.

Both mandibles with lateral margins almost straight. Basal half with fine, simple setae scattered over dorsal surface.

*Hypopharynx* (Fig. 54f). Lingua shorter than superlingua. Lingua longer than broad; medial tuft of stout setae present; distal half laterally expanded. Superlingua rounded; lateral margin rounded; fine, long, simple setae along distal margin.

*Maxilla* (Fig. 54g). Galea-lacinia with two simple, robust apical setae under crown. Inner dorsal row of setae with three denti-setae, distal denti-seta tooth-like, middle and proximal denti-setae slender, bifid and pectinate. Medially with one bipectinate, spine-like seta and seven long, simple setae. Maxillary palp 1.6× as long as length of galea-lacinia; two segmented. Palp segment II 1.9× length of segment I. Setae on maxillary palp fine and simple, scattered over surface of segments I and II. Apex of last segment slightly pointed, without excavation at inner distolateral margin.

*Labium* (Fig. 54h). Glossa basally broad, narrowing toward apex; shorter than paraglossa; inner margin with ten spine-like setae increasing in length distally; apex with three long, robust setae; outer margin with ten long, spine-like setae; ventral surface with fine, simple setae. Paraglossa sub-rectangular, curved inward; apex rounded; with three rows of long, robust, apically pectinate setae; dorsally with ten medium, simple setae; ventrally with six long, spine-like setae near inner margin. Labial palp with segment I 0.7× length of segments II and III combined. Segment I covered with short, fine, simple setae ventrally and micropores dorsally. Segment II with a large, lobed distomedial protuberance; distomedial protuberance 0.8× width of base of segment III; inner and outer margin both with short, fine, simple setae; dorsally with row of five long, spine-like, simple setae. Segment III conical; apex slightly pointed; length 1.2× width; ventrally covered with medium spine-like, simple setae and short, fine, simple setae.

*Hind wing pads* absent.

*Foreleg* (Fig. 55a, b). Ratio of foreleg segments 1.4:1.0:0.8:0.2. *Femur*. Length ca. 4× maximum width. Dorsal margin with a row of ca. 27 curved, short, spine-like setae and with stout, pointed setae near margin; length of setae 0.14× maximum width of femur. Apex rounded; with two pairs of curved, spine-like setae and many short, stout, pointed setae. Many stout, lanceolate setae and a few fine, simple setae along ventral margin; femoral patch poorly developed. *Tibia.* Dorsal margin with a row of short, curved, spine-like setae and long, fine, simple setae. Ventral margin with a row of curved, spine-like setae, apically longer, dense and bipectinate and with a tuft of long, fine, simple setae. Anterior surface scattered with many stout, lanceolate setae, partly bipectinate (difficult to see). Tibio-patellar suture present on basal 1/2. *Tarsus.* Dorsal margin with a row of short, curved, spine-like setae. Ventral margin with a row of curved, spine-like setae. Tarsal claw with one row of 13–14 denticles; distally pointed; with 5–6 stripes; subapical setae absent.

*Tergum* (Fig. 55c). Surface with irregular rows of U-shaped scale bases and scattered fine, simple setae, scales short and apically rounded. Posterior margin of tergum IV with rounded spines, wider than long.

*Gills* (Fig. 55d). Present on segments II–VII. Margin with small denticles intercalating long, fine, simple setae. Tracheae extending from main trunk to inner and outer margins. Gill IV as long as length of segments V, VI and 1/2 VII combined. Gill VII as long as length of segments VIII and IX combined.

*Paraproct* (Fig. 55e). Distally slightly expanded, with many marginal, stout spines. Surface with U-shaped scale bases and scattered fine, simple setae. Postero-lateral extension (cercotractor) with small marginal spines.

###### Etymology.

Refers to Pindaunde Creek in Papua New Guinea, where the species has been collected.

###### Distribution.

New Guinea.

###### Biological aspects.

The specimens were collected at altitudes of 1700 m, 2600 m and 2900 m a.s.l., partly in forest.

###### Type-material.

**Holotype.** Nymph (on slide, GBIFCH 00465222), Papua New Guinea, Simbu Prov., 05°49.00'S, 145°04.50'E, (GPS), Mt. Wilhelm, Pindaunde Creek, 2900 m a.s.l. (in forest), S3 (oria.4),18 Aug 1999, leg. L. Čížek. Deposited in MZL. **Paratypes.** 13 nymphs (1 on slide, GBIFCH 00465223, 8 in alcohol, GBIFCH 00515236, GBIFCH 00508125, deposited in MZL; 4 in alcohol, GBIFCH 00515237, deposited in ZSM), same data as holotype; 2 nymphs (1 on slide, GBIFCH 00465224, 1 in alcohol, GBIFCH 00515238, deposited in MZL), Papua New Guinea, Simbu Prov., 05°49.03'S, 145°05.27'E, Mt. Wilhelm, Pindaunde Creek, 2600 m a.s.l. (near fish farm), (9181 ft GPS), S4 (oria.5), 18 Aug 1999, L. Čížek leg.

###### Additional material.

10 nymphs (1 on slide, GBIFCH 00465225, 9 in alcohol, GBIFCH 00515278, deposited in MZL), Papua New Guinea, Central, Woitape, 1700 m, 01.2008, 08°31.29'S, 147°13.68'E, Posman (PNG 166).

##### 31. Labiobaetis vallus sp. n.

Taxon classificationAnimaliaEphemeropteraBaetidae

http://zoobank.org/BBD0E979-C891-4605-97E0-AF015900E94E

**Figure 56.**
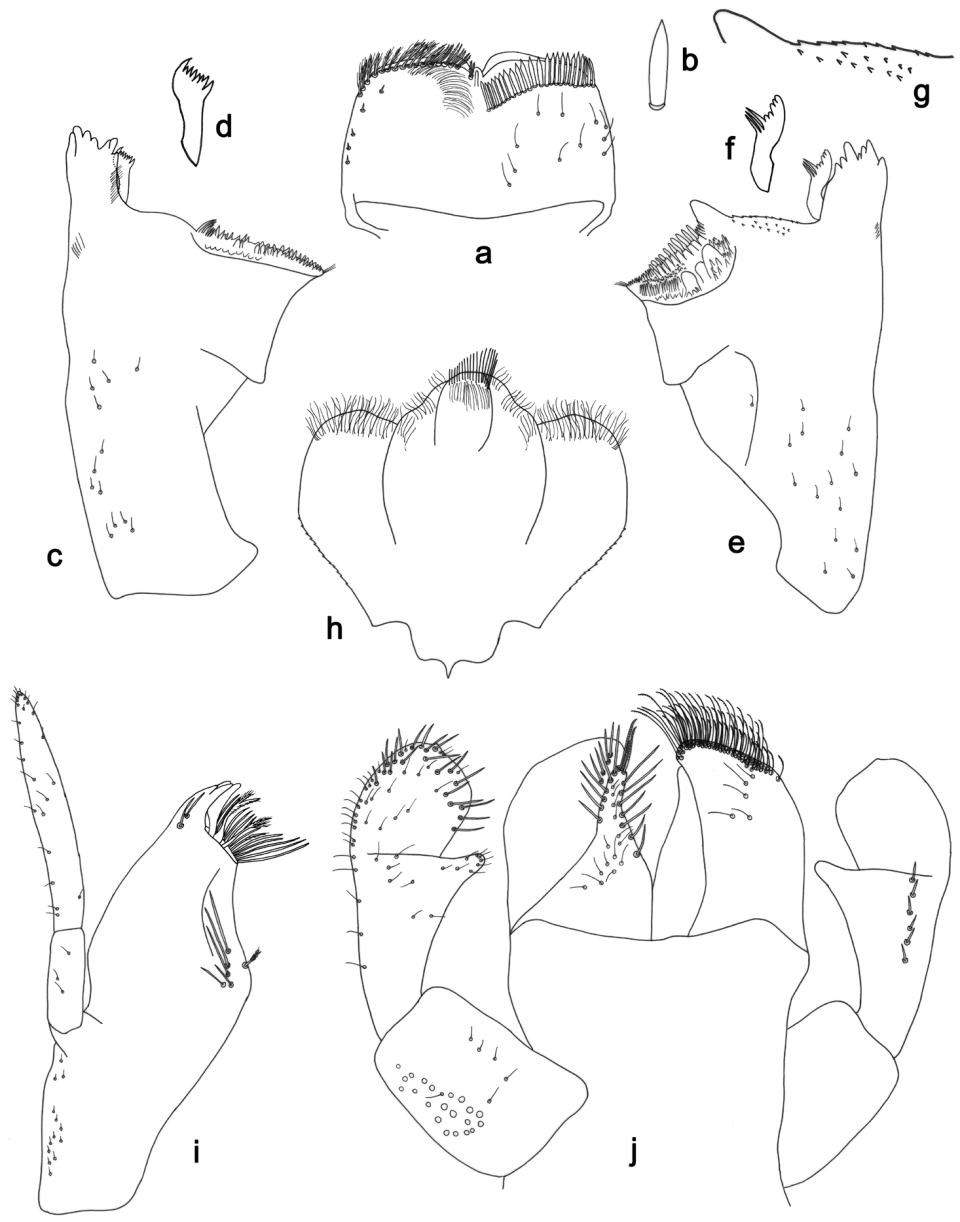
*Labiobaetisvallus* sp. n., larva morphology: **a** Labrum **b** Labrum dorsal, submarginal seta **c** Right mandible **d** Right prostheca **e** Left mandible **f** Left prostheca **g** Left mandible, area between prostheca and mola **h**Hypopharynx**i** Maxilla **j** Labium.

**Figure 57.**
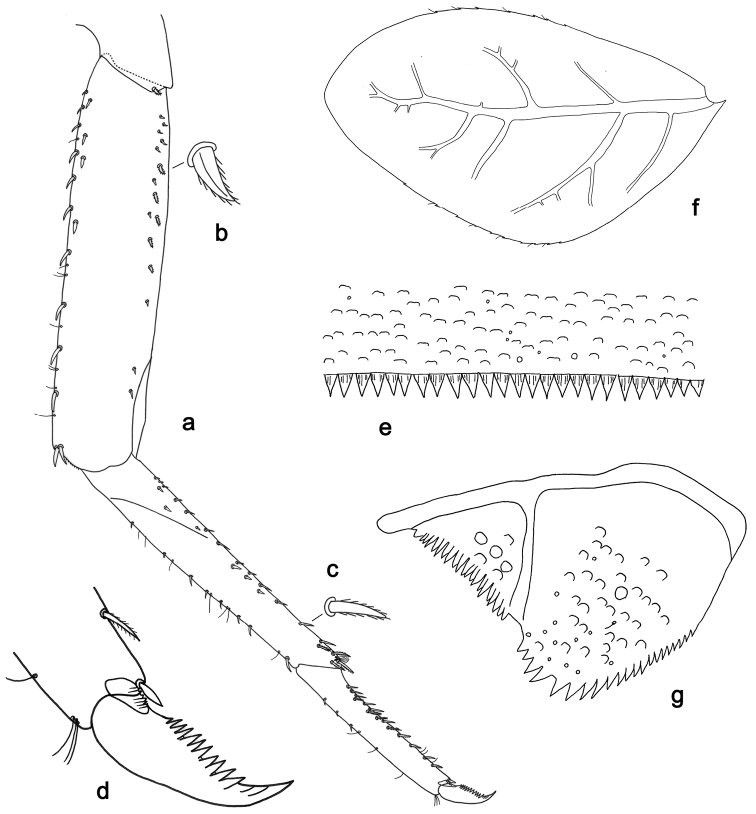
*Labiobaetisvallus* sp. n., larva morphology: **a**Foreleg**b** Femur ventral seta **c** Tibia bipectinate seta **d** Fore claw **e**Tergum IV **f** Gill III **g**Paraproct.

Figures 56 , 57 , 63d , 64b

###### Diagnosis.

**Larva.** Following combination of characters: A) labrum dorsal submarginal arc of setae composed of 23 long, lanceolate, apically pointed setae; B) maxillary palp with segment II 2.4× longer than length of segment I, slender, apically pointed, without excavation at inner lateral margin; C) labial palp segment III sub-rectangular, apically slightly pointed; D) labial palp segment II with an elongated, thumb-like distomedial protuberance; E) fore femur slender, length ca. 4× maximum width, dorsal margin with a row of ca. 10 curved, spine-like setae and some stout, pointed setae near margin.

###### Description.

**Larva** (Figs 56, 57, 63d). Body length 5.4 mm.

*Colouration.* Thorax and abdomen dorsally brown, with bright pattern as in Fig. 63d. Thorax with bright median, dorsal line. Thorax and abdomen ventrally colourless, abdomen light brown toward the end. Legs colourless, femur with proxomedial and distomedial brown spots, tibia with proximal and distal brown spots. Caudal filaments light brown.

*Antenna* with scape and pedicel sub-cylindrical, without distolateral process at scape.

*Labrum* (Fig. 56a, b). Rectangular, length 0.6× maximum width. Distal margin with medial emargination and a small process. Dorsally with medium, fine, simple setae scattered over surface; submarginal arc of setae composed of 23 long, lanceolate setae. Ventrally with marginal row of setae composed of lateral and anterolateral long, feathered setae and medial long, bifid, pectinate setae; ventral surface with five short, spine-like setae near lateral and anterolateral margin.

*Right mandible* (Fig. 56c, d). Incisors fused. Outer and inner sets of denticles with 4 + 3 denticles. Inner margin of innermost denticle with a row of thin setae. Prostheca robust, apically denticulate. Margin between prostheca and mola slightly convex. Tuft of setae at apex of mola present.

*Left mandible* (Fig. 56e–g). Incisors fused. Outer and inner sets of denticles with 3 + 4 denticles and one minute intermediate denticle. Prostheca robust, apically with small denticles and comb-shape structure. Margin between prostheca and mola straight, with minute denticles towards subtriangular process. Subtriangular process long and slender, above level of area between prostheca and mola. Denticles of mola apically constricted. Tuft of setae at apex of mola present.

Both mandibles with lateral margins almost straight. Basal half with fine, simple setae scattered over dorsal surface.

*Hypopharynx* (Fig. 56h). Lingua longer than superlingua. Lingua about as broad as long; medial tuft of stout setae present; distal half not expanded. Superlingua rounded; lateral margin angulate; fine, long, simple setae along distal margin.

*Maxilla* (Fig. 56i). Galea-lacinia with two simple, robust apical setae under crown. Inner dorsal row of setae with three denti-setae, distal denti-seta tooth-like, middle and proximal denti-setae slender, bifid and pectinate. Medially with one bipectinate, spine-like seta and five long, simple setae. Maxillary palp 1.2× as long as length of galea-lacinia; two segmented. Palp segment II 2.4× length of segment I. Setae on maxillary palp fine and simple, scattered over surface of segments I and II. Apex of last segment slightly pointed, without excavation at inner distolateral margin.

*Labium* (Fig. 56j). Glossa basally broad, narrowing toward apex; shorter than paraglossa; inner margin with seven spine-like setae increasing in length distally; apex with three long, robust, pectinate setae; outer margin with six long spine-like setae increasing in length distally; ventral surface with fine, simple setae. Paraglossa sub-rectangular, curved inward; apex rounded; with three rows of long, robust, apically pectinate setae; dorsally with five medium, simple setae; ventrally with four long, spine-like setae near inner margin. Labial palp with segment I 0.7× length of segments II and III combined. Segment I covered with short and medium, fine, simple setae ventrally and with micropores dorsally. Segment II with an elongated, thumb-like distomedial protuberance; distomedial protuberance 0.5× width of base of segment III; inner and outer margin both with short, fine, simple setae; dorsally with row of six medium, spine-like, simple setae. Segment III sub-rectangular; apex slightly pointed; length 1.0× width; ventrally covered with long and medium spine-like, simple setae and short, fine, simple setae.

*Hind wing pads* absent.

*Foreleg* (Fig. 57a–d). Ratio of foreleg segments 1.4:1.0:0.6:0.2. *Femur*. Length ca. 4× maximum width. Dorsal margin with a row of ca. 10 curved, spine-like setae and some stout, pointed setae near margin; length of setae 0.2× maximum width of femur. Apex rounded; with one pair of curved, spine-like setae and some minute setae. Stout, lanceolate, bipectinate setae along ventral margin; femoral patch absent. *Tibia.* Dorsal margin with a row of curved, spine-like setae and long, fine, simple setae. Ventral margin with a row of curved, spine-like setae, apically longer, dense and bipectinate and with a tuft of long, fine, simple setae. Anterior surface scattered with stout, lanceolate setae. Tibio-patellar suture present on basal 1/2. *Tarsus.* Dorsal margin with a row of short, spine-like setae and long, simple setae. Ventral margin with a row of curved, spine-like, bipectinate setae. Tarsal claw with one row of 11–13 denticles; distally pointed; with two stripes; subapical setae absent.

*Tergum* (Fig. 57e). Surface with irregular rows of shallow U-shaped scale bases and scattered micropores. Posterior margin of tergum IV with triangular spines, longer than wide.

*Gills* (Fig. 57f). Present on segments II–VII. Margin with small denticles intercalating fine simple setae. Tracheae extending from main trunk to inner and outer margins. Gill IV unknown, gill III as long as segments IV and 1/2 V combined. Gill VII unknown.

*Paraproct* (Fig. 57g). Distally not expanded, with ca. 21 marginal, stout spines. Surface with U-shaped scale bases and scattered fine, simple setae and micropores. Postero-lateral extension (cercotractor) with small marginal spines.

###### Etymology.

Refers to the remarkable, fence-like, dorsal, submarginal arc of setae of the labrum.

###### Distribution.

New Guinea.

###### Biological aspects.

The specimen was collected at an altitude of 400 m a.s.l.

###### Type-material.

**Holotype.** Nymph (on slide, GBIFCH 00465226), Papua New Guinea, Madang, Keki, Adalbert Mts, 400 m, 29 Nov 2006, 04°43.06'S, 145°24.44'E, Binatang Boys (PNG 119). Deposited in ZSM.

##### 32. Labiobaetis xeniolus

Taxon classificationAnimaliaEphemeropteraBaetidae

(Lugo-Ortiz & McCafferty, 1999)

Figure 65b

###### Diagnosis.

**Larva.** Following combination of characters: A) labrum dorsal submarginal arc of setae composed of one plus 7–9 long, simple setae; B) maxillary palp shorter than length of galea-lacinia, segment II without excavation at inner distolateral margin; C) labial palp segment II with elongated thumb-like distomedial protuberance, segment III about semicircular; D) femur rather broad, length ca. 2–3× maximum width, dorsal margin with >40 curved, spine-like setae; E) fore claw with a row of 8–10 denticles; F) Gills with strongly developed, dense, pigmented tracheation; G) spines at posterior margin of tergum IV irregular, mostly triangular, apically rounded, wider than long; H) paraproct with marginal spines poorly defined.

###### Examined material.

**Paratypes.** 2 nymphs (on slides, PERC 0.012.578, PERC 0.012.579), Papua New Guinea, Morobe Prov., Poverty Cr., Mt. Missim, 1600 m, 18.09. Sept 1983, J.T. and D.A. Polhemus leg.

### Key to the nymphs of *Labiobaetis* species from New Guinea

**Table:** 

1	Labrum submarginal dorsal arc of simple setae (Fig. 1a)	**2**
–	Labrum submarginal dorsal arc of lanceolate setae (Fig. 1e)	***L.vallus* sp. n.**
–	Labrum submarginal dorsal arc of lanceolate, apically pectinate setae (Fig. 1f)....	***L.inopinatus* sp. n.**
–	Labrum submarginal dorsal arc of spatulate, apically pectinate setae (Fig. 1d)	**17**
–	Labrum submarginal dorsal arc of feathered setae (Fig. 1b)	**19**
–	Labrum submarginal dorsal arc of dendritic setae (Fig. 1c)	***L.dendrisetis* sp. n.**
2(1)	Labial palp segment II enlargement elongated, thumb-like (Fig. 1j)	**3**
–	Labial palp segment II enlargement short, thumb-like (Fig. 1k)	**7**
–	Labial palp segment II enlargement compact, rounded (Fig. 1h)	**12**
–	Labial palp segment II enlargement large, lobed (Fig. 1g)	***L.pindaundensis* sp. n.**
–	Labial palp segment II enlargement hook-like (Fig. 1i)	**16**
3(2)	Labial palp segment III subrectangular (Fig. 8h)	***L.claudiae* sp. n.**
–	Labial palp segment III about semicircular (Fig. 1j)	**4**
–	Labial palp segment III conical (Fig. 1h)	**6**
–	Labial palp segment III oblong (Fig. 1k)	***L.gindroi* sp.n.**
4(3)	Mandibles with outermost denticle normally developed (Fig. 1m); maxillary palp segment II longer than segment I; spines at posterior margin of abdominal terga longer than wide; spines at posterior margin of abdominal terga mostly triangular, pointed	**5**
–	Mandibles with outermost denticle blade-like (Fig. 1l); maxillary palp segment II about as long or shorter than segment I; spines at posterior margin of abdominal terga wider than long; spines at posterior margin of abdominal terga mostly rounded; paraproct with marginal spines poorly defined (Lugo-Ortiz et al. 1999: figs 104–115)	*** L. xeniolus ***
5(4)	Maxillary palp shorter than length of galea-lacinia; gills margin serrate with alternating smaller and bigger denticles and with fine, simple setae; number of femur dorsal setae on margin > 40; paraproct distally not expanded (Lugo-Ortiz et al. 1999: figs 31–42)	*** L. involutus ***
–	Maxillary palp longer than length of galea-lacinia; gills margin serrate with small denticles and with fine simple setae (Fig. 11d); number of femur dorsal setae on margin 12–20; paraproct distally expanded (Fig. 11e)	***L.stagnum* sp. n.**
6(3)	Labial palp segment III distal margin rounded (Fig. 46h); maxillary palp segment II about as long or shorter than segment I; spines at posterior margin of abdominal terga about as long as wide; fore femur ca. 2–3× as long as wide	***L.centralensis* sp. n.**
–	Labial palp segment III distal margin truncate (Fig. 39h); maxillary palp segment II longer than segment I; spines at posterior margin of abdominal terga wider than long; fore femur ca. 4–5× as long as wide	***L.paravitilis* sp. n.**
7(2)	Labium paraglossae distally with three rows of setae	**8**
–	Labium paraglossae distally with four rows of setae	***L.altus* sp. n.**
–	Labium paraglossae distally with five rows of setae	***L.wilhelmensis* sp. n.**
8(7)	Mandibles with outermost denticle normally developed (Fig. 1m)	**9**
–	Mandibles with outermost denticle blade-like (Fig. 1l)	**10**
9(8)	Labial palp segment III conical, distal margin with emargination (Fig. 50h); maxillary palp shorter than length of galea-lacinia; spines at posterior margin of abdominal terga wider than long	***L.elisae* sp. n.**
–	Labial palp segment III slightly pentagonal, distal margin rounded (Fig. 34d); maxillary palp longer than length of galea-lacinia; spines at posterior margin of abdominal terga about as long as wide	*** L. vitilis ***
10(8)	Labial palp segment III distal margin slightly pointed (Fig. 1k)	***L.gladius* sp. n.**
–	Labial palp segment III distal margin rounded (Fig. 16c)	**11**
11(10)	Maxillary palp segment II longer than segment I; spines at posterior margin of abdominal terga wider than long; gills margin serrate with small denticles and with fine simple setae (Fig. 20d); number of femur dorsal setae on margin 31–40	***L.janae* sp. n.**
–	Maxillary palp segment II about as long or shorter than segment I; spines at posterior margin of abdominal terga longer than wide; gills margin serrate with alternating smaller and bigger denticles and with fine, simple setae (Lugo-Ortiz et al. 1999: fig. 54); number of femur dorsal setae on margin > 40	*** L. petersorum ***
12(2)	Maxillary palp about as long as length of galea-lacinia	***L.magnovaldus* sp. n.**
–	Maxillary palp longer (1.2–1.4×) than length of galea-lacinia	**13**
–	Maxillary palp much longer (> 1.4×) than length of galea-lacinia	***L.planus* sp. n.**
13(12)	Gills margin serrate with small denticles and with robust, lanceolate setae (Fig. 23d, e)	***L.branchiaesetis* sp. n.**
–	Gills margin serrate with alternating smaller and bigger denticles and with fine, simple setae (Fig. 29d); paraproct surface with several robust, lanceolate setae (Fig. 29e, f)	***L.podolakae* sp. n.**
–	Gills margin serrate with small denticles and with fine simple setae (Fig. 31d)	**14**
14(13)	Spines at posterior margin of abdominal terga about as long as wide; labrum submarginal dorsal arc of setae with 0–9 setae; maxillary palp segment II with well-developed distolateral excavation; paraproct distally not or slightly expanded (Fig. 1r, s)	**15**
–	Spines at posterior margin of abdominal terga wider than long; labrum submarginal dorsal arc of setae with 10–14 setae; maxillary palp segment II without distolateral excavation; paraproct distally expanded (Fig. 1q)	***L.rutschmannae* sp. n.**
15(14)	Spines at posterior margin of abdominal terga mostly rounded; paraproct with > 40 marginal spines; tibia as Fig. 33a	***L.schwanderae* sp. n.**
–	Spines at posterior margin of abdominal terga mostly triangular, pointed; paraproct with 31–40 marginal spines; tibia as Fig. 21c	*** L. tuberpalpus ***
16(2)	Maxillary palp segment II longer than segment I; gills margin serrate with alternating smaller and bigger denticles and with fine, simple setae (Fig. 45d); fore femur ca. 2–3× as long as wide; labial palp as Fig. 44h	***L.paravultuosus* sp. n.**
–	Maxillary palp segment II about as long or shorter than segment I; gills margin serrate with small denticles and with fine simple setae; fore femur ca. 4–5× as long as wide; labial palp as Fig. 43b..	*** L. vultuosus ***
17(1)	Maxillary palp longer (1.1–1.4×) than length of galea-lacinia; fore femur ca. 2–3× as long as wide	**18**
–	Maxillary palp much longer (> 1.4×) than length of galea-lacinia; fore femur ca. 4–5× as long as wide	***L.lobatus* sp. n.**
18(17)	Maxillary palp segment II longer than segment I; spines at posterior margin of abdominal terga mostly triangular, pointed; paraproct distally expanded (Fig. 3f)	***L.balkei* sp. n.**
–	Maxillary palp segment II about as long or shorter than segment I; spines at posterior margin of abdominal terga mostly rounded; paraproct distally slightly expanded (Fig. 7e)	***L.michaeli* sp. n.**
19(1)	Spines at posterior margin of abdominal terga mostly rounded; femur ventral without pectinate setae	***L.papuaensis* sp. n.**
–	Spines at posterior margin of abdominal terga mostly triangular, pointed; femur ventral with pectinate setae (difficult to see)	***L.orientis* sp. n.**

**Figure 58.**
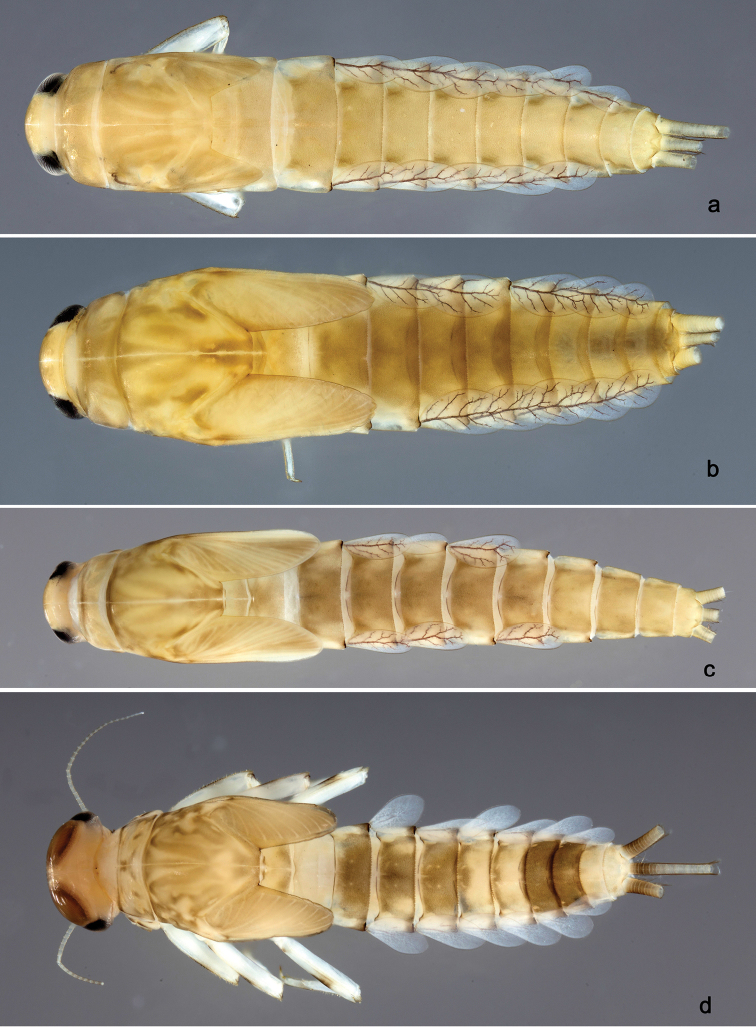
Habitus, larvae: **a***Labiobaetisbalkei* sp. n., dorsal view **b***Labiobaetislobatus* sp. n., dorsal view **c***Labiobaetismichaeli* sp. n., dorsal view **d***Labiobaetisclaudiae* sp. n., dorsal view.

**Figure 59.**
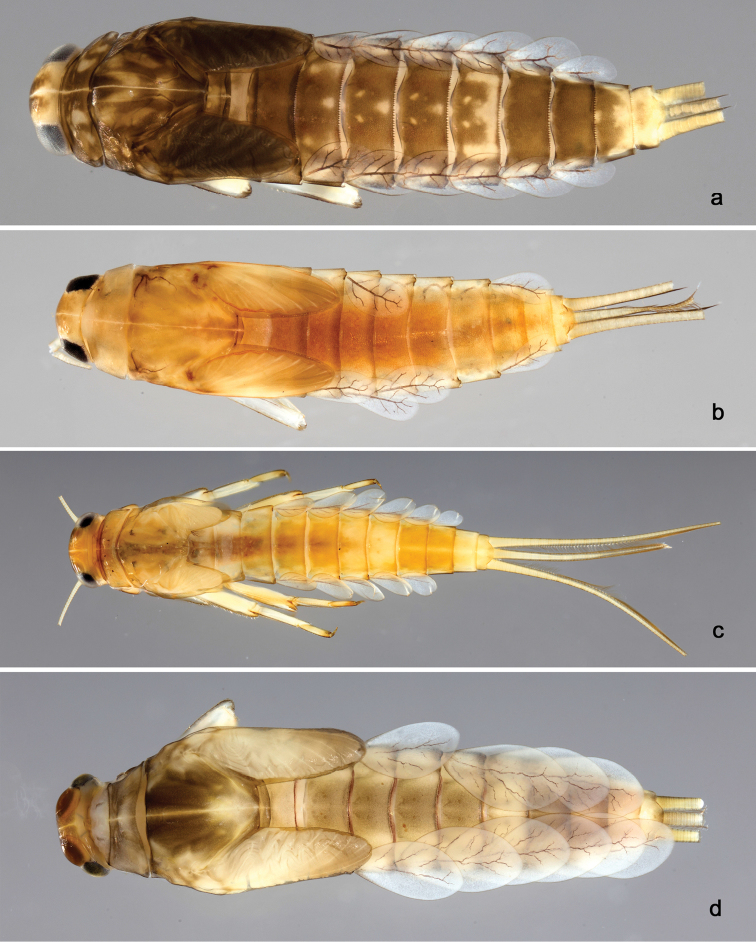
Habitus, larvae: **a***Labiobaetisstagnum* sp. n., dorsal view **b***Labiobaetispapuaensis* sp. n., dorsal view **c***Labiobaetisgladius* sp. n., dorsal view **d***Labiobaetisjanae* sp. n., dorsal view.

**Figure 60.**
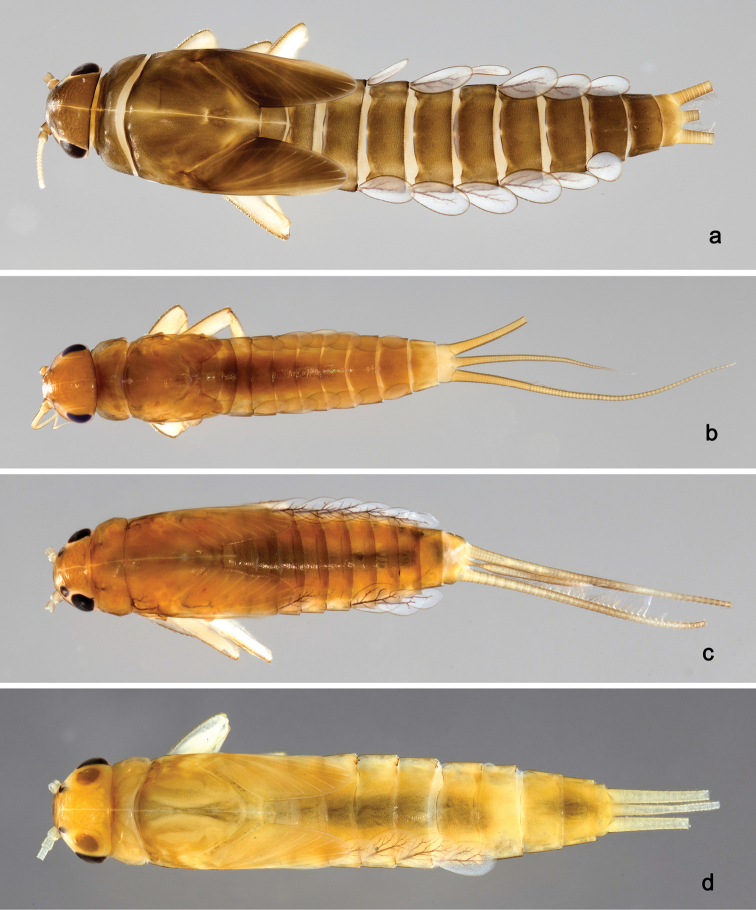
Habitus, larvae: **a***Labiobaetisbranchiaesetis* sp. n., dorsal view **b***Labiobaetismagnovaldus* sp. n., dorsal view **c***Labiobaetisplanus* sp. n., dorsal view **d***Labiobaetispodolakae* sp. n., dorsal view.

**Figure 61.**
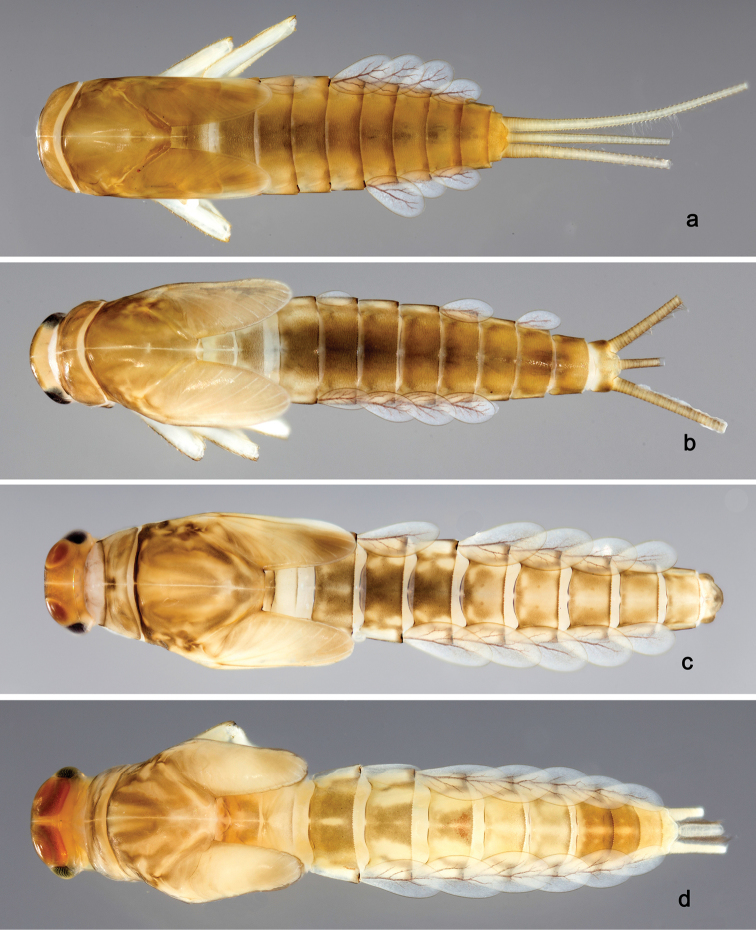
Habitus, larvae: **a***Labiobaetisrutschmannae* sp. n., dorsal view, without head **b***Labiobaetisschwanderae* sp. n., dorsal view **c***Labiobaetisaltus* sp. n., dorsal view **d***Labiobaetisgindroi* sp. n., dorsal view.

**Figure 62.**
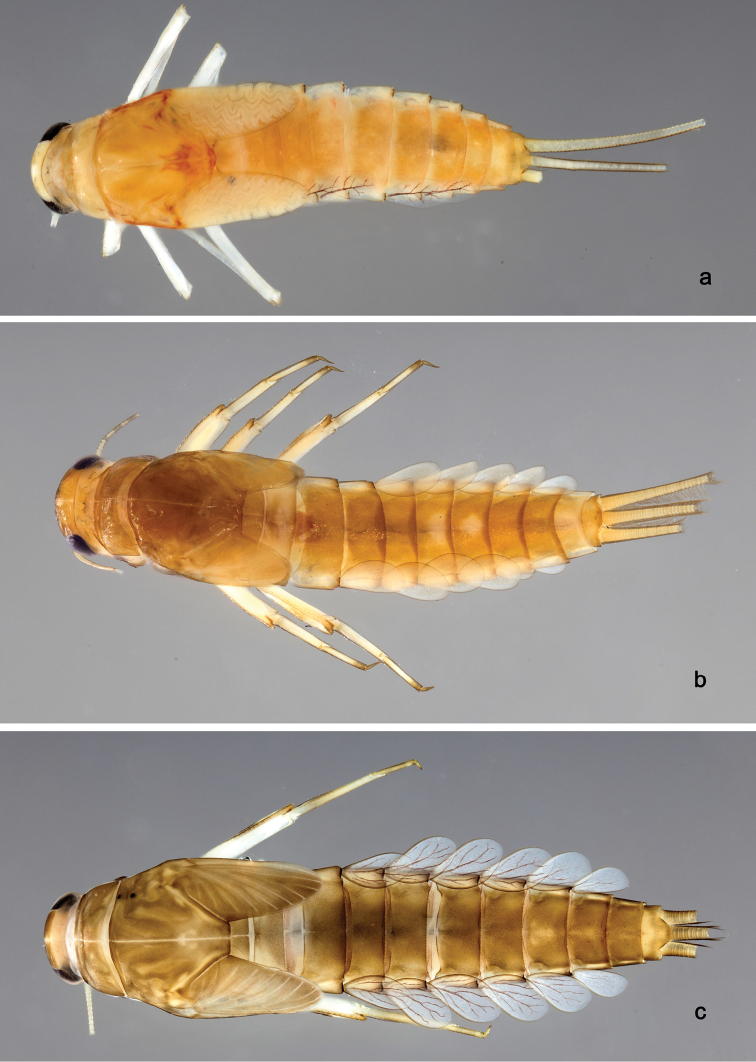
Habitus, larvae: **a***Labiobaetisparavitilis* sp. n., dorsal view **b***Labiobaetiswilhelmensis* sp. n., dorsal view **c***Labiobaetisparavultuosus* sp. n., dorsal view.

**Figure 63.**
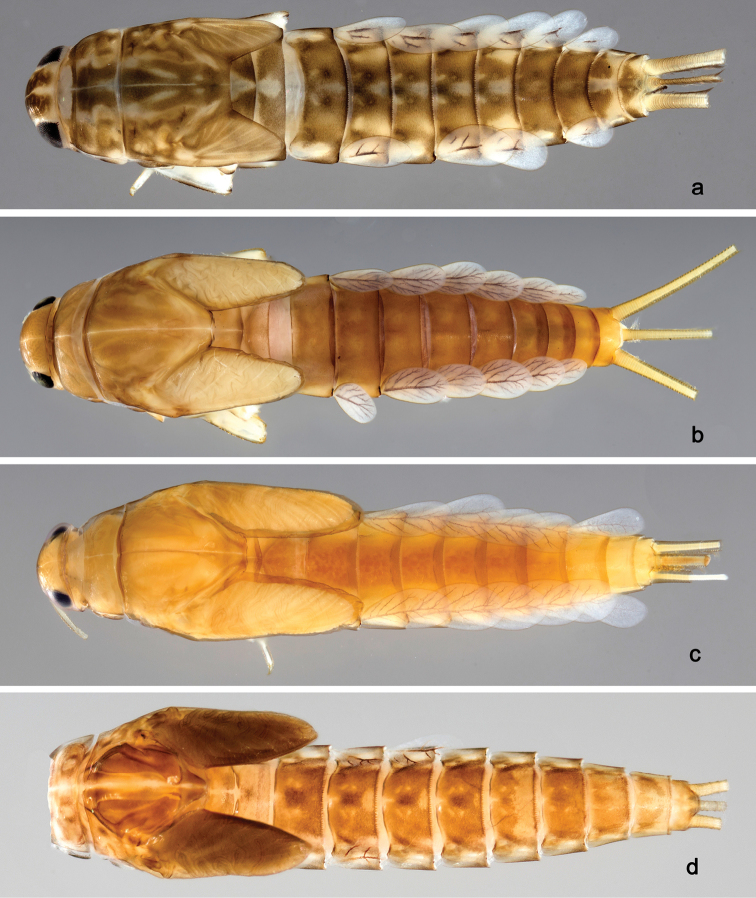
Habitus, larvae: **a***Labiobaetiscentralensis* sp. n., dorsal view **b***Labiobaetiselisae* sp. n., dorsal view **c***Labiobaetispindaundensis* sp. n., dorsal view **d***Labiobaetisvallus* sp. n., dorsal view, without head.

## Distributions

**Figure 64.**
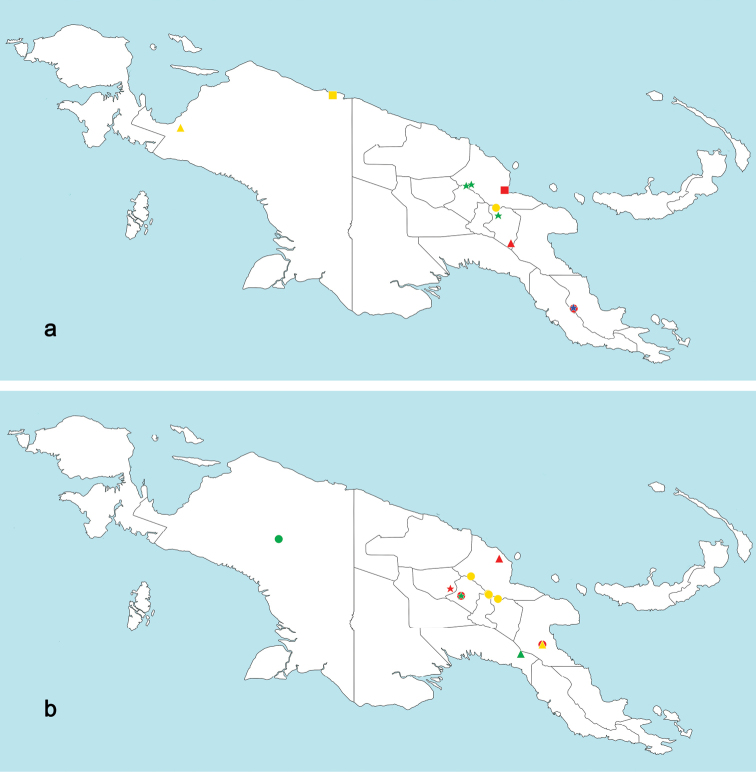
Distribution of *Labiobaetis* in New Guinea: **a** red circle, *L.balkei* sp. n.; blue star, *L.lobatus* sp. n.; yellow circle, *L.michaeli* sp. n.; red square, *L.claudiae* sp. n.; yellow square, *L.stagnum* sp. n.; red triangle, *L.orientis* sp. n.; yellow triangle, *L.papuaensis* sp. n.; green star, *L.elisae* sp. n. **b** red circle, *L.petersorum*; yellow circle, *L.gladius* sp. n.; green circle, *L.janae* sp. n.; green star, *L.vultuosus*; red star, *L.paravultuosus* sp. n.; yellow triangle, *L.involutus*; red triangle, *L.vallus* sp. n.; green triangle, *L.inopinatus* sp. n.

**Figure 65.**
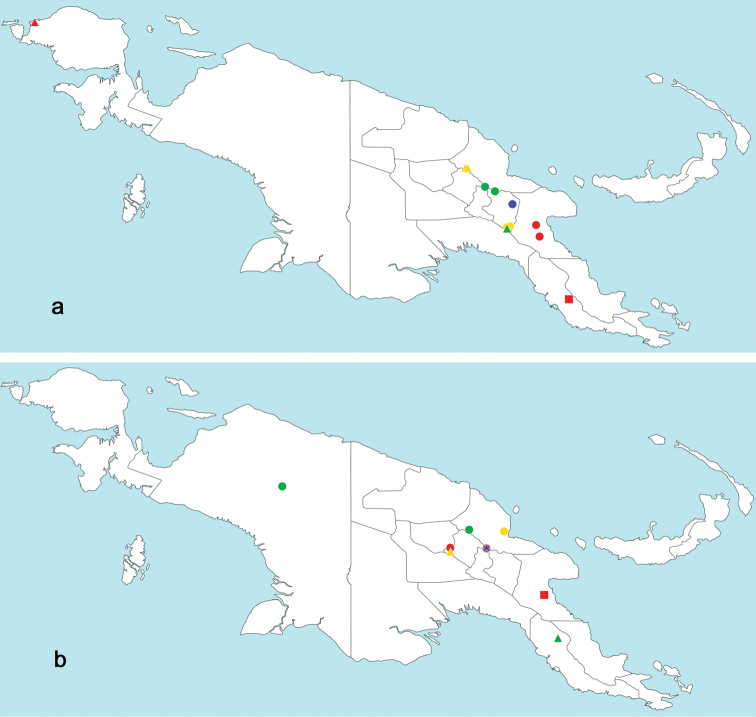
Distribution of *Labiobaetis* in New Guinea: **a** red circle, *L.tuberpalpus*; yellow circle, *L.branchiaesetis* sp. n.; green circle, *L.magnovaldus* sp. n.; red triangle, *L.planus* sp. n.; blue circle, *L.podolakae* sp. n.; yellow triangle, *L.rutschmannae* sp. n.; green triangle, *L.schwanderae* sp. n.; red square, *L.centralensis* sp. n. **b** yellow circle, *L.paravitilis* sp. n.; red circle, *L.altus* sp. n.; green circle, *L.gindroi* sp. n.; yellow triangle, *L.vitilis*; green triangle, *L.pindaundensis* sp. n.; red square, *L.xeniolus*; blue star, *L.wilhelmemsis* sp. n. / *L.dendrisetis* sp. n. / *L.pindaundensis* sp. n.

The distributions of all species known from New Guinea are shown in Figures 64, 65. The six species described in Lugo-Ortiz et al. (1999) were all collected in two restricted areas. The recent material treated in this study was collected in many locations across the island. However, there are still other regions in New Guinea where no sampling of mayflies has yet been done and many species are known from only one single population so far. In terms of altitude, the *Labiobaetis* species of New Guinea were found from close to sea level near the coast to high parts of the mountains till up to 3210 m a.s.l. The GPS coordinates of the locations in New Guinea are given in Table 2.

## Genetics

COI sequences were obtained from 20 of the new species (Table 1). The genetic distances between these species are between 13% and 32% and therefore always much higher than 3.5%, generally considered as a likely maximal value for intraspecific divergence (Hebert et al. 2003, Ball et al. 2005, Zhou et al. 2010) (Table 3). Only very limited genetic distances between 0% and 2% were found between specimens of the same species, as in *L.balkei* sp. n., *L.janae* sp. n., *L.planus* sp. n., *L.paravultuosus* sp. n. and *L.centralensis* sp. n. The exception is *L.michaeli* sp. n., where two specimens have no distance to each other (K2P), but the third specimen from the same location has a distance of 5% (K2P and *p*-distance) to both of them. However, sequences were obtained from only one to a few specimens per species (Table 1). In general, the genetic distances calculated with K2P are very similar to the *p*-distance values, but often slightly bigger, as expected (Srivathsan and Meier 2012). Both methods supported the same delimitation of species.

**Table 2.** GPS coordinates of locations of examined specimens.

Species	Locality	GPS coordinates	
*L.balkei* sp. n.	Papua New Guinea, Central Prov.	09°01.95'S, 147°44.46'E	
*L.lobatus* sp. n.	Papua New Guinea, Central Prov.	09°00.34'S, 147°44.25'E	
*L.michaeli* sp. n.	Papua New Guinea, Eastern Highlands	05°56.80'S, 145°22.24'E	
*L.claudiae* sp. n.	Papua New Guinea, Madang Prov.	05°24.41'S, 145°38.21'E	
*L.stagnum* sp. n.	Indonesia, Papua Prov.	02°30.51'S, 140°22.83'E	
*L.orientis* sp. n.	Papua New Guinea, Eastern Highlands	07°01.70'S, 145°49.81'E	
*L.papuaensis* sp. n.	Indonesia, Papua Prov.	03°29.80'S, 135°43.89'E	
* L. petersorum *	Papua New Guinea, Morobe Prov.	07°20.30'S, 146°43.72'E	
*L.gladius* sp. n.	Papua New Guinea, Simbu Prov.	05°49.96'S, 145°06.13'E	
		05°48.05'S, 145°04.15'E	
		05°49.03'S, 145°05.27'E	
	Papua New Guinea, Western Highlands	05°15.87'S, 144°32.72'E	
	Papua New Guinea, Eastern Highlands	05°56.80'S, 145°22.23'E	
*L.janae* sp. n.	Indonesia, Papua Prov.	04°07.77'S, 138°40.77'E	
* L. tuberpalpus *	Papua New Guinea, Morobe Prov.	07°20.30'S, 146°43.72'E	
		06°59.00'S, 146°37.00'E	
*L.branchiaesetis* sp. n.	Papua New Guinea, Eastern Highlands	07°01.70'S, 145°49.81'E	
	Papua New Guinea, Gulf Prov.	07°03.60'S, 145°44.38'E	
*L.magnovaldus* sp. n.	Papua New Guinea, Simbu Prov.	05°49.00'S, 145°04.50'E	
	Papua New Guinea, Eastern Highlands	05°56.80'S, 145°22.24'E	
*L.planus* sp. n.	Indonesia, Papua Prov.	00°49.35'S, 131°24.20'E	
*L.podolakae* sp. n.	Papua New Guinea, Eastern Highlands	06°21.41'S, 145°54.34'E	
*L.rutschmannae* sp. n.	PapuaNewGuinea,WesternHighlands	05°14.28'S, 144°28.74'E	
		05°15.17'S, 144°32.81'E	
*L.schwanderae* sp. n.	Papua New Guinea, Gulf Prov.	07°05.66'S, 145°44.47'E	
* L. vitilis *	Papua New Guinea, Western Highlands	05°54.91'S, 143°59.06'E	
*L.altus* sp. n.	Papua New Guinea, Enga Prov.	05°47.55'S, 143°58.76'E	
	Papua New Guinea, Simbu Prov.	05°49.00'S, 145°04.50'E	
*L.gindroi* sp. n.	Indonesia, Papua Prov.	03°56.95'S, 138°54.38'E	
	Papua New Guinea, Western Highlands	05°15.17'S, 144°32.81'E	
*L.paravitilis* sp. n.	Papua New Guinea, Madang Prov.	05°18.09'S, 145°36.45'E	
*L.wilhelmensis* sp. n	Papua New Guinea, Simbu Prov.	05°49.00'S, 145°04.50'E	
		05°48.05'S, 145°04.15'E	
* L. vultuosus *	Papua New Guinea, Western Highlands	05°51.00'S, 144°14.72'E	
*L.paravultuosus* sp. n.	Papua New Guinea, Enga Prov.	05°38.11'S, 143°55.34'E	
*L.centralensis* sp. n.	Papua New Guinea, Central Prov.	09°14.34'S, 147°36.92'E	
*L.dendrisetis* sp. n.	Papua New Guinea, Simbu Prov.	05°49.00'S, 145°04.50'E	
*L.elisae* sp. n.	Papua New Guinea, Western Highlands	05°16.10'S, 144°27.87'E	
	Papua New Guinea, Madang Prov.	05°13.39'S, 144°37.29'E	
	Papua New Guinea, Eastern Highlands	06°11.02'S, 145°26.41'E	
*L.inopinatus* sp. n.	Papua New Guinea, Gulf Prov.	07°37'S	146°04'E
*L.pindaundensis* sp. n.	Papua New Guinea, Simbu Prov.	05°49.00'S, 145°04.50'E	
		05°49.03'S, 145°05.27'E	
	Papua New Guinea, Central Prov.	08°31.29'S, 147°13.68'E	
*L.vallus* sp. n.	Papua New Guinea, Madang Prov.	04°43.06'S, 145°24.44'E	
* L. involutus *	Papua New Guinea, Morobe Prov.	07°20.30'S, 146°43.72'E	
* L. xeniolus *	Papua New Guinea, Morobe Prov.	07°13.00'S, 146°49.00'E	

**Table 3.** Genetic distances (COI) between sequenced specimens, using the Kimura 2-parameter.

		1	2	3	4	5	6	7	8	9	10	11	12	13	14	15	16	17	18	19	20	21	22	23	24	25	26
1	*L.balkei* sp. n.																										
2	*L.balkei* sp. n.	0,00																									
3	*L.michaeli* sp. n.	0,26	0,26																								
4	*L.michaeli* sp. n.	0,26	0,26	0,00																							
5	*L.michaeli* sp. n.	0,28	0,28	0,05	0,05																						
6	*L.lobatus* sp. n.	0,20	0,20	0,28	0,28	0,28																					
7	*L.claudiae* sp. n.	0,21	0,21	0,25	0,25	0,27	0,24																				
8	*L.stagnum* sp. n.	0,22	0,22	0,27	0,27	0,26	0,22	0,21																			
9	*L.orientis* sp. n.	0,26	0,26	0,27	0,27	0,26	0,24	0,18	0,22																		
10	*L.papuaensis* sp. n.	0,27	0,27	0,25	0,25	0,25	0,24	0,20	0,19	0,21																	
11	*L.gladius* sp. n.	0,22	0,22	0,25	0,25	0,27	0,24	0,19	0,23	0,24	0,20																
12	*L.janae* sp. n.	0,24	0,24	0,29	0,29	0,29	0,24	0,23	0,23	0,23	0,24	0,23															
13	*L.janae* sp. n.	0,24	0,24	0,29	0,29	0,29	0,24	0,23	0,23	0,23	0,24	0,23	0,00														
14	*L.branchiaesetis* sp. n.	0,20	0,20	0,26	0,26	0,25	0,24	0,18	0,21	0,22	0,23	0,19	0,19	0,19													
15	*L.planus* sp. n.	0,22	0,22	0,25	0,25	0,25	0,24	0,18	0,16	0,19	0,17	0,18	0,18	0,18	0,18												
16	*L.planus* sp. n.	0,22	0,22	0,25	0,25	0,25	0,24	0,18	0,16	0,19	0,17	0,18	0,18	0,18	0,18	0,00											
17	*L.podolakae* sp. n.	0,25	0,25	0,25	0,25	0,25	0,24	0,20	0,19	0,22	0,20	0,18	0,20	0,20	0,14	0,17	0,17										
18	*L.schwanderae* sp. n.	0,21	0,21	0,25	0,25	0,25	0,24	0,18	0,18	0,19	0,19	0,16	0,22	0,22	0,19	0,13	0,13	0,19									
19	*L.altus* sp. n.	0,22	0,22	0,29	0,29	0,30	0,24	0,20	0,18	0,15	0,20	0,22	0,24	0,24	0,22	0,18	0,18	0,20	0,20								
20	*L.gindroi* sp. n.	0,26	0,26	0,24	0,24	0,25	0,24	0,21	0,19	0,20	0,19	0,22	0,19	0,19	0,20	0,20	0,20	0,20	0,18	0,16							
21	*L.paravitilis* sp. n.	0,24	0,24	0,27	0,27	0,26	0,24	0,24	0,20	0,24	0,21	0,21	0,22	0,22	0,17	0,21	0,21	0,21	0,20	0,19	0,20						
22	*L.paravultuosus* sp. n.	0,22	0,22	0,24	0,24	0,24	0,24	0,20	0,18	0,21	0,18	0,21	0,21	0,21	0,19	0,15	0,15	0,19	0,19	0,19	0,23	0,20					
23	*L.paravultuosus* sp. n.	0,23	0,23	0,26	0,26	0,26	0,24	0,23	0,20	0,23	0,20	0,23	0,22	0,22	0,21	0,17	0,17	0,21	0,21	0,21	0,25	0,21	0,02				
24	*L.centralensis* sp. n.	0,22	0,22	0,31	0,31	0,29	0,24	0,21	0,22	0,25	0,23	0,23	0,20	0,20	0,24	0,22	0,22	0,21	0,22	0,23	0,22	0,26	0,26	0,27			
25	*L.centralensis* sp. n.	0,23	0,23	0,32	0,32	0,30	0,24	0,21	0,23	0,24	0,23	0,23	0,20	0,20	0,24	0,22	0,22	0,21	0,21	0,23	0,22	0,25	0,26	0,27	0,01		
26	*L.elisae* sp. n.	0,22	0,22	0,28	0,28	0,27	0,24	0,19	0,20	0,21	0,19	0,21	0,23	0,23	0,22	0,17	0,17	0,19	0,18	0,19	0,19	0,19	0,20	0,21	0,21	0,21	
27	*L.vallus* sp. n.	0,26	0,26	0,25	0,25	0,26	0,24	0,18	0,18	0,24	0,19	0,21	0,19	0,19	0,17	0,17	0,17	0,18	0,18	0,21	0,16	0,19	0,21	0,23	0,20	0,20	0,20

## Discussion

For the attribution of the new species to *Labiobaetis* we are referring to Kluge and Novikova (2014), Müller-Liebenau (1984a) and McCafferty and Waltz (1995). The plesiomorph *Labiobaetis* is characterised by a number of derived characters, some of which are not found in other taxa (Kluge and Novikova 2014): antennal scape sometimes with a distolateral process (not developed by any species in New Guinea); maxillary palp two segmented with excavation at inner distolateral margin of segment II, excavation may be poorly developed or absent (Fig. 1n–p); labium with paraglossae widened and glossae diminished; labial palp segment II with distomedial protuberance (Fig. 1g–k). All these characters vary and may be secondarily lost (Kluge and Novikova 2014). The concept of *Labiobaetis* is also based on additional characters (Müller-Liebenau 1984a, McCafferty and Waltz 1995, Lugo-Ortiz and McCafferty 1997, Lugo-Ortiz et al. 1999). The discovery of 26 new species allows us to slightly modify these characters: labrum dorsal, submarginal setae are arranged in one arc, the setae may belong to a simple, pointed type, a feathered type, a dendritic type, a spatulate type or a lanceolate, apically pectinate or not pectinate type (Fig. 1 a–f); mandibles with fused incisors, right prostheca apically denticulate, left prostheca apically denticulate and with comb-shape structure; hypopharynx with medial tuft of stout setae at apex of median lobe; paraglossae subrectangular, slightly curved inward; hindwing pads present, minute or absent (always absent in species from New Guinea); femoral patch well developed, rudimentary or absent; tibia at apical margin with a tuft of fine, simple setae; tarsal claw distally pointed with one row of denticles, striation present, subapical setae absent; abdominal terga with irregular rows of numerous U-shaped or rarely W-shaped scale bases, posterior margin with regular, triangular, pentagonal or rounded spines; gills on abdominal segment I present or absent (mostly absent in species from New Guinea); paraproct with ca. 18 to over 40 marginal spines, laterally always smaller, and distally expanded, slightly expanded or not expanded at all (Fig. 1q–s). The medial tuft of setae of the hypopharynx is an important character, as it is stable across the genus and quite rare in other genera. Another very important character is the femoral patch, which is considered to be a synapomorphy of the Baetini (Waltz et al. 1994), in *Labiobaetis* it is often rudimentary or absent, which seems to be a secondary loss. There are a few taxa outside *Labiobaetis* with a convergent excavation at the maxillary palp: one is *Indobaetiscostai* Müller-Liebenau & Morihara, 1982 (Müller-Liebenau and Morihara 1982: figs 1d, 2d, McCafferty and Waltz 1995, Kluge and Novikova 2014), other species of *Indobaetis* do not show this character; other two species belong to the Neotropical genus *Zelusia* Lugo-Ortiz & McCafferty, 1998 (Salles et al. 2016). Two species from New Guinea (*L.orientis* sp. n., *L.vallus* sp. n.) have bipectinate setae ventrally on tibia and femur similar to *Indocloeon* Müller-Liebenau, 1982, but not on the dorsal margin of the femur as *Indocloeon* (Kluge 2012, Kaltenbach and Gattolliat 2017).

The seven species groups proposed in this paper are mainly based on the combination of two characters: the kind of setae composing the dorsal, submarginal arc of setae on the labrum and the shape of the distomedial protuberance of labial palp segment II, sometimes together with other additional characters. An exception is the *petersorum* group, where the main character is the blade-like outermost denticle of the mandibles. Species sharing combinations of character states as the type of setae forming the dorsal, submarginal arc of setae or the shape of the labial palp segment II are often very similar in most other characters, which justify the formation of species groups. These morphological groups within *Labiobaetis* are primarily a working tool, but could also serve as a basis for future studies on the generic delimitation and phylogeny of this probably polyphyletic genus. The inclusion of nuclear gene sequences may prove that some of them may be natural groups. In other realms, especially the Oriental realm, some species share the main characters of some of the proposed species groups: *L.molawinensis* (Müller-Liebenau) from the Philippines and Taiwan and *L.atrebatinus* (Eaton) from Eurasia have spatulate, apically pectinate setae composing the dorsal, submarginal arc of setae of the labrum and a large, lobed distomedial protuberance of the labial palp segment II like the species of the *balkei* group. They also share other characters such as the distolateral excavation of maxillary palp segment II, the shape of labial palp segment III, which is slightly pentagonal and apically slightly pointed, and the arrangement of outer and inner sets of denticles of the mandibles, which are as usually fused, but have a small gap between them. Therefore both species should be considered as members of that group. They are both differentiated from the species in New Guinea by a distolateral process at the antennal scape and *L.atrebatinus* additionally by the presence of hindwing pads and seven pairs of gills (Müller-Liebenau 1982, Kang et al. 1994). Another Oriental species, *L.borneoensis* (Müller-Liebenau), has a lobed distomedial protuberance at labial palp segment II combined with feathered dorsal labrum arc setae as seen in the two species of the *orientis* group and therefore belongs to this group as well (Müller-Liebenau 1984b). It is easily differentiated by having seven pairs of gills, an antennal scape process and hindwing pads, which the two species from New Guinea don’t have. The Australian *L.inconspicuus* (Lugo-Ortiz & McCafferty) shares the compact, rounded protuberance of labial palp segment II, the simple dorsal labrum arc setae and the closely neighboured position of the two first labrum arc setae with the species of the *tuberpalpus* group in New Guinea and may therefore belong to this group as well (Lugo-Ortiz et al. 1999, Webb and Suter 2011). However, it is missing the pair of central, submedian setae dorsally on the labrum. The *L.vultuosus* group of species consists of two species in New Guinea and is characterised by a hook-like protuberance of the labial palp segment II in combination with simple labrum arc setae. *L.sumigarensis* (Müller-Liebenau) from the Philippines shares a very similar labial palp, but has spatulate, apically pectinate dorsal submarginal labrum arc setae (Müller-Liebenau 1982). It is therefore not considered to be part of that group. As a whole we see some remarkable differences between the morphology of species from New Guinea compared to other regions: in New Guinea there are no species with an antennal scape process, all but one species have only six pairs of gills, there are no species with hindwing pads and most species have simple dorsal labrum arc setae. All other regions have several species with or without antennal scape process, with six or seven pairs of gills, with or without hindwing pads and the balance between the main types of dorsal labrum arc setae (simple, feathered, spatulate) is more equalised. The latter is especially true in the Oriental realm, whereas the feathered type seems to be dominant in the Afrotropical region (Lugo-Ortiz and McCafferty 1997, Gattolliat 2001, Gattolliat et al. 2018). The tendency of secondary loss of certain structures seems to be particularly strong in New Guinea, alternatively it could also be due to the colonization history of the island and thus a phylogenetic signal. Additionally, there is more variability in some morphological characters in New Guinea compared to all other regions: Examples are the dendritic or lanceolate dorsal labrum arc setae of *L.dendrisetis* sp. n. and *L.vallus* sp. n. respectively, the robust, lanceolate setae at the margin of the gills of *L.branchiaesetis* sp. n., the robust, lanceolate setae on the surface of the paraproct of *L.podolakae* sp. n. and the blade-like outermost denticle of the mandibles of the *petersorum* group.

In general, the genetic distances between the different species of *Labiobaetis* are rather high in New Guinea, on average 22% (K2P, Table 3). Ball et al. (2005) reported a mean interspecific, congeneric distance of 18% for mayflies from the United States and Canada. The intraspecific distances are very low as expected, ranging from 0 % to 2% (K2P), based on the limited number of sequenced specimens per species, which were mostly from one single population. However, there is one exception, *L.michaeli* sp. n., where one specimen has an intraspecific distance of 5% (K2P and *p*-distance). Sequencing errors may have contributed to that, but also compared to the usual distances between different *Labiobaetis* species in that region we consider this distance to be still intraspecific. Ball et al. (2005) also reported a case with 6% intraspecific distance in a mayfly in North America and intraspecific K2P distances of more than 3.5% are also not uncommon within Plecoptera (Gill et al. 2015, Gattolliat et al. 2016).

In addition to the 26 new species described in this paper we obtained five other different COI sequences with clearly interspecific genetic distance to other species with similar morphology: two sequences L.cf.balkei sp. n. (K2P 16%–30%), two sequences L.cf.vultuosus (K2P 16%–21%) and one sequence L.cf.tuberpalpus (K2P 13%). Based on the existing material we are not in a position to distinguish these specimens morphologically from all species within their morphological group. Therefore, they have to remain species hypotheses for now without further treatment in this paper. Additional material will be necessary to confirm their status in the future.

The *Labiobaetis* species distribution seems to be restricted to New Guinea, as far as we know, which is fully in line with other Baetidae genera having a species endemicity of close to 100% in the Australasian realm (Gattoliat and Nieto 2009). In Madagascar there is a similar situation with eight species, which are all restricted to this island (Lugo-Ortiz and McCafferty 1997, Gattolliat 2001). On the other hand, there is the Afrotropical *L.glaucus* (Agnew) with a widespread distribution reaching from South Africa to Saudi Arabia, including even the Comoros (Lugo-Ortiz and McCafferty 1997, Gattolliat et al. 2018). Borneo is equally known for its enormous diversity. During an 85 km^2^ survey of the mayfly fauna of a lowland tropical forest more than 40 mayfly genera were collected and at least 10 new genera and many new species were discovered (Barber-James et al. 2008, Sartori et al. 2003). So far only one *Labiobaetis* species is known from this island, but several others were already identified and will be described later (Kaltenbach and Gattolliat, in preparation), and Borneo is less well sampled than Madagascar.

Our recent knowledge shows high levels of micro-endemism restricted to smaller areas in New Guinea. This indicates that allopatry could be a major driver of diversity in the genus. Large studies about the highly diversified genus *Exocelina* Balke, 1998 (Coleoptera, Dytiscidae) demonstrated allopatry to be the main mechanism of diversification in New Guinea and found strong evidence that recent environmental change in the extremely structured central highlands of New Guinea with its ongoing formation of rich aquatic resources and remote valleys and mountain blocks was the primary driver of diversification in that area (Toussaint et al. 2013, 2014). There is also evidence that species in running waters are weaker dispersers then species living in standing water, which has been suggested to promote allopatric speciation and micro-endemism in the first group and dispersal in the second group (Ribera et al. 2001, Monaghan et al. 2005). *Labiobaetis* species mainly live in running waters, but there are a few exceptions. On the other hand, their dispersal ability seems to be high enough to have reached remote islands like Vanuatu (Gattolliat and Staniczek 2011) and Fiji (Flowers 1990) in the past and bidirectional transoceanic dispersal between Madagascar and Africa has been shown as well (Monaghan et al. 2005). Additionally, parthenogenesis has been demonstrated in the genus, which may favor successful dispersal events as well (Sivaramakrishnan et al. 1991, Gattolliat and Staniczek 2011).

Despite covering an important part of New Guinea, the sampling effort and the number of localities and different habitats is still limited and there are huge areas without any collection activities so far. Additionally we have already five species hypotheses based on genetics only in the present material, which may be confirmed as separate species in the future. Therefore we may assume, that the number of *Labiobaetis* species in New Guinea will continue to increase substantially with further collections in the future.

## Supplementary Material

XML Treatment for Labiobaetis balkei

XML Treatment for Labiobaetis lobatus

XML Treatment for Labiobaetis michaeli

XML Treatment for Labiobaetis claudiae

XML Treatment for Labiobaetis stagnum

XML Treatment for Labiobaetis orientis

XML Treatment for Labiobaetis papuaensis

XML Treatment for Labiobaetis petersorum

XML Treatment for Labiobaetis gladius

XML Treatment for Labiobaetis janae

XML Treatment for Labiobaetis tuberpalpus

XML Treatment for Labiobaetis branchiaesetis

XML Treatment for Labiobaetis magnovaldus

XML Treatment for Labiobaetis planus

XML Treatment for Labiobaetis podolakae

XML Treatment for Labiobaetis rutschmannae

XML Treatment for Labiobaetis schwanderae

XML Treatment for Labiobaetis vitilis

XML Treatment for Labiobaetis altus

XML Treatment for Labiobaetis gindroi

XML Treatment for Labiobaetis paravitilis

XML Treatment for Labiobaetis wilhelmensis

XML Treatment for Labiobaetis vultuosus

XML Treatment for Labiobaetis paravultuosus

XML Treatment for Labiobaetis centralensis

XML Treatment for Labiobaetis dendrisetis

XML Treatment for Labiobaetis elisae

XML Treatment for Labiobaetis inopinatus

XML Treatment for Labiobaetis involutus

XML Treatment for Labiobaetis pindaundensis

XML Treatment for Labiobaetis vallus

XML Treatment for Labiobaetis xeniolus
